# Supplementary material for: Deaths and cardiopulmonary events following colorectal cancer screening—A systematic review with meta-analyses
Source: PLoS One. 2024 Mar 14;19(3):e0295900. doi: 10.1371/journal.pone.0295900 (PMC10939197; doi:10.1371/journal.pone.0295900)
Supplement: S1 File — (PDF) [file pone.0295900.s001.pdf]

S1 – Appendices

Content

Appendix 1 – Definitions & Eligibility criteria 3
Appendix 2 – Search strategy 7
Overall search strategy for all databases: 7
Pubmed MeSH 7
Pubmed keyword 8
Embase Emtree 8
Embase keyword 12
Cinahl 21
PsycInfo 26
The Cochrane database 30
Appendix 3 – Reasons for exclusion 31
Appendix 4 – Data extraction templates 32
Appendix 5 – GRADE approach 36
Appendix 6 – Subcategories of CPEs 42
Appendix 7 – Procedures per person for the four screening procedure groups 50
Appendix 8 - Study characteristics of included studies 51
sTable7a. Study characteristics of included studies 51
sTable7b. Characteristics of subpopulations with assessment of deaths 62
sTable7c. Characteristics of subpopulations with assessment of cardiopulmonary events 64
Appendix 9 – Ongoing RCTs on CRCSPs 68
Appendix 10 - Study characteristics of special case studies and studies with an unscreened control group 71
Deaths associated with CRCSPs using data from special case studies and studies with an unscreened control group 72
Cardiopulmonary events associated with CRCSPs using data from special case studies and studies with an unscreened control group 73
Appendix 11 – Studies from countries 74
Appendix 12 – Characteristics of studies with assessment of CPEs or death 78
Appendix 13 – Study characteristics related to screening procedure group 79

Appendix 14 – Conceptualization of physical harms across studies 87
Appendix 15 – Risk of bias in studies with assessments of death 92
Appendix 16 – Risk of bias in studies with assessments of CPEs 95
Appendix 17 – GRADE assessments of findings on deaths 99
Appendix 18 – GRADE assessments of findings on CPEs 109
Appendix 19 – Studies identified and harms assessed in other systematic reviews 138
Appendix 20 - Deaths associated with CRCSPs compared to other reviews 140
References 141

Appendix 1 – Definitions & Eligibility criteria

Screening

The term “screening” is used to describe the entire screening cascade; from the invitation to screening procedures to further diagnostic workup, surveillance, incidental findings etc. (Figure 2 in main article).

We limited our scope to physical harm due to sigmoidoscopy or colonoscopy performed during CRCSPs.

Conventional colorectal cancer screening methods

We define conventional colorectal cancer screening methods as the use of the following screening tools in any combination: Faecal occult blood test (FOBT)/faecal immunochemical test (FIT), sigmoidoscopy and colonoscopy. We also include bowel preparation/enema of any type as this accompanies sigmoidoscopy/colonoscopy.

Harm of screening

Harm is defined as the totality of adverse consequences of screening, being the direct opposite of benefits [1]. The term “harm” thereby comprises both complications, safety issues, adverse events, adverse effects and side effects occurring in relation to screening for colorectal cancer. Harms are not only medical issues but any negative effect perceived by the screening participant and/or their significant others to result from screening participation [2].

Causal inference between harm and screening

Harm might occur at any step of the screening cascade. Whether a harm occurring in relation to screening is a random correlation or a causal effect of screening can always be discussed. We therefore include any harm as defined above occurring during the screening cascade and include everything correlated to colorectal cancer screening we assess how study authors might have assessed causal inference between harm and screening.

Physical harm

Any type of harm of screening deemed somatic in nature, i.e. affecting the body of the individual. This includes:

- Major adverse events requiring medical assistance such as perforation or bleeding due to colonoscopy, anaesthesia complications, perioperative complications, infections, cardiopulmonary complications etc.
- Other somatic complaints not necessarily resulting in medical assistance such as discomfort due to bowel preparation, loss of sleep, physical symptoms due to diagnostic procedures, bloating, water-electrolyte disturbances etc.

Types of physical harm

General terms that might be physical: Adverse event/effect/outcome, complications, harm, safety, side effect, toxicity, perioperative complications (preoperative, operative, postoperative), burden, Iatrogenic, medical error, poisoning

Physical harms:

Infection, death, pain, symptomatic/physical complaint/symptom, discomfort, gastrointestinal event, cardiopulmonary event, hypovolemia, ischemia, shock, water-electrolyte imbalance,

Specific terms to CRC screening we define as physical:

Transmural burn syndrome, post-polypectomy syndrome, gas explosion, colon injury, perforation, bleeding/haemorrhage, milt rupture, abdominal injury,

Types of harm (From PRISMA harms statement paper)

Adverse event: An unfavourable outcome that occurs during or after the use of a drug or other intervention but is not necessarily caused by it

Adverse effect: An unfavourable outcome that occurs during or after the use of a drug or other intervention and the causal relation between the intervention and the event is at least a reasonable possibility

Complication: An adverse event or effect following surgical and other invasive intervention

Harm: The totality of possible adverse consequences (if single or multiple) of an intervention or therapy; harms are the direct opposite of benefits

Safety: Substantive evidence of an absence of harm. The term is often misused when there is simply absence of evidence of harm

Side effect: Any unintended effect, adverse or beneficial, of a drug that occurs at doses normally used for treatment

Toxicity: Drug related harm. The term may be most appropriate for laboratory determined measurements, although it is also used in relation to clinical events. The disadvantage of the term “toxicity” is that it implies causality. If authors cannot prove causality, the terms “abnormal laboratory measurements” or “laboratory abnormalities” are more appropriate

sTable 1. Eligibility criteria

	Inclusion	Exclusion
**Population**	40 years < Age < 80 years*<br><br>Mean risk of CRC and asymptomatic regarding signs of CRC (screening population)[1].	People at higher than average risk of CRC. People with family history of CRC, known genetic susceptibility to CRC or known IBD.<br>Non-screening populations (symptomatic, earlier or current diagnosis of CRC)
**Setting**	Settings corresponding to clinical screening settings in real life	Research or industrial based settings that diverge from normal screening setting to an extent that comparison with screening setting is not possible. Including general practice or specialized endoscopic treatment centers.
**Intervention**	Conventional CRC screening tools, defined as all of the following screening techniques in any type of combination: Faecal occult blood test (guaiac or immunochemical tests), sigmoidoscopy and colonoscopy.	Stool testing using in-office digital rectal exam (DRE). Genetic testing for increased risk of CRC. Blood tests such as septins. Stool DNA test. Capsule endoscopy or CT colonography.<br>Any interventions after the screening colonoscopy/sigmoidoscopy, i.e. people referred for further work-up/treatment or people participating in surveillance porammes due to former identification of cancer or cancer precursors, i.e. polyps.<br>Any interventions other than conventional screening methods designed to reduce harm, e.g. music therapy, pain medication etc.
**Outcomes**	Any type of physical harm no matter its severity, potential causality or consequences, thus comprising the totality of adverse consequences deemed physical in nature. Physical harms occurring due to the diagnostic work-up procedures of CRCSPs: sigmoidoscopy and/or colonoscopy with or without polypectomy. No restrictions on follow-up time.	Expected harms of screening from participants or harm of screening in the view of physicians or other health professionals. Including simulated numbers of harm in microsimulation studies. Any study not reporting about harms in title, abstract or full text.
**Study design**	All types of original research: RCTs, CCTs, cohort studies, case-control studies, cross-sectional studies. Case studies/case series**	Systematic reviews. Qualitative studies.

		Any article not related to original data/research: Journalism, editorials, narrative reviews, and opinions as letters or comments.<br>Studies reporting harm while comparing technical aspects of procedures such as type of colonoscope, different anaesthetic procedures, different bowel preparation schemes or different polypectomy techniques.

[1]Mixed populations were included if data was stratified corresponding to these eligibility criteria or when the majority of the study population corresponds to the eligibility criteria, i.e. small discrepancies are accepted. [2]Referencelists in reviews deemed relevant to the research question will be scrutinized for studies not found via the search strategy.
*We accepted studies that included people above and below the desired age interval as many screening programmes provide the opportunity for people outside the age group to opt-in.
**Case series/case reports were included to identify all potential types of physical harm due to colorectal cancer screening. However, these types of studies are not able to quantify the risk of harm and therefore these studies are not part of harm quantification in this publication.
Abbreviations: CRC = Colorectal cancer, IBD = Inflammatory Bowel Disease

Appendix 2 – Search strategy

Overall search strategy for all databases:

The search strategy was divided into an index word search and a keyword search to identify all relevant studies for each database searched.

CRC screening AND physical harm

CRC screening = ((Screening AND laxative) OR (Screening AND CRC) OR CRC screening technologies)

Search strategies were performed via use of index words in databases supplemented by searches via selected keywords in separate searches. The original search was developed for Medline/Pubmed and following translated to the terminology used in other databases. The search stratgeies are presented below for Medline, Pubmed and Embase. The Cochrane library use the same terminology as in Medline and is therefore not described separately below.

Pubmed MeSH

((("Colonoscopy"[3] OR "Sigmoidoscopy"[3] OR "Occult Blood"[3] OR "Colonic Neoplasms/surgery"[3] OR "Intestinal Polyps/surgery"[3] OR "Colorectal Neoplasms/prevention and control"[3])) OR (((("intestinal polyps"[MeSH Terms] OR "cecal neoplasms"[MeSH Terms] OR "colorectal neoplasms"[MeSH Terms]) OR ("adenoma"[MeSH Terms] AND ("Intestine, Large"[3] OR "Intestinal Neoplasms"[3])))) AND ("Early Diagnosis"[3] OR "Secondary Prevention"[3] OR "Early Detection of Cancer"[3] OR "Mass Screening"[3] OR "Preventive Health Services"[3] OR "Early Medical Intervention"[3]))) OR ((((("Colonoscopy"[3] OR "Sigmoidoscopy"[3] OR "Occult Blood"[3] OR "Colonic Neoplasms/surgery"[3] OR "Intestinal Polyps/surgery"[3] OR "Colorectal Neoplasms/prevention and control"[3])) OR ("Early Diagnosis"[3] OR "Secondary Prevention"[3] OR "Early Detection of Cancer"[3] OR "Mass Screening"[3] OR "Preventive Health Services"[3] OR "Early Medical Intervention"[3]))) AND ("Cathartics"[3] OR "Laxatives"[3]))

AND

"Postoperative Complications"[3] OR "Intraoperative Complications"[3] OR "Death"[3] OR "Pain"[3] OR "Pain Measurement"[3] OR "Signs and Symptoms, Digestive"[3] OR "Infection"[3] OR "Blood Loss, Surgical"[3] OR "Heart Diseases"[3] OR "Hypovolemia"[3] OR "Ischemia"[3] OR "Hypersensitivity"[3] OR "Inflammation"[3] OR "Shock"[3] OR "Ulcer"[3] OR "Intestinal Perforation"[3] OR "Abdominal Injuries"[3] OR "Iatrogenic Disease"[3] OR "Medical Errors"[3] OR "Safety"[3] OR "Long Term Adverse Effects"[3] OR "Water-Electrolyte Imbalance"[3] OR "Colon/injuries"[3] OR "Early Diagnosis/adverse effects"[3] OR "Early Diagnosis/mortality"[3] OR "Early Detection of Cancer/adverse effects"[3] OR "Early Detection of Cancer/mortality"[3] OR "Mass Screening/adverse effects"[3] OR "Mass Screening/mortality"[3] OR "Preventive Health Services/adverse effects"[3] OR "Preventive Health Services/complications"[3] OR "Preventive Health Services/mortality"[3] OR "Colonoscopy/adverse effects"[3] OR "Colonoscopy/complications"[3] OR "Colonoscopy/mortality"[3] OR "Sigmoidoscopy/adverse effects"[3] OR "Sigmoidoscopy/complications"[3] OR "Sigmoidoscopy/mortality"[3] OR "Occult Blood/adverse effects"[3] OR "Cathartics/adverse effects"[3]

OR "Cathartics/poisoning"[3] OR "Cathartics/toxicity"[3] OR "Laxatives/adverse effects"[3] OR "Laxatives/poisoning"[3] OR "Laxatives/toxicity"[3]

Pubmed keyword

(((((((fecal immunochemical test*[tiab] OR fobt[tiab] OR colonoscop*[tiab] OR sigmoidoscop*[tiab] OR occult blood[tiab] OR hemoccult*[tiab] OR polypect*[tiab])) OR (early detect*[tiab] OR early diagnos*[tiab] OR prevent*[tiab] OR screen*[tiab] OR early intervent*[tiab]))) AND (laxativ*[tiab] OR Cathartic*[tiab] OR purgativ*[tiab] OR bowel prepa*[tiab] OR colon cleans*[tiab]))) OR (((colorectal neoplasm*[tiab] OR colorectal adenoma*[tiab] OR colorectal carcinoma*[tiab] OR colorectal carcinogenesis[tiab] OR colorectal adenocarcinoma*[tiab] OR colorectal tumor*[tiab] OR colorectal polyp*[tiab] OR colorectal cancer*[tiab] OR colon adenoma*[tiab] OR colon carcinoma*[tiab] OR colon carcinogenesis[tiab] OR colon adenocarcinoma*[tiab] OR colon tumor*[tiab] OR colon polyp*[tiab] OR colon cancer*[tiab] OR colon neoplasm*[tiab] OR bowel cancer*[tiab] OR intestine cancer*[tiab] OR intestinal adenoma*[tiab] OR cecal neoplasm*[tiab] OR intestinal polyp*[tiab])) AND (early detect*[tiab] OR early diagnos*[tiab] OR prevent*[tiab] OR screen*[tiab] OR early intervent*[tiab]))) OR (fecal immunochemical test*[tiab] OR fobt[tiab] OR colonoscop*[tiab] OR sigmoidoscop*[tiab] OR occult blood[tiab] OR hemoccult*[tiab] OR polypect*[tiab])

AND

"intraoperative complication*"[tiab] OR "adverse outcome*"[tiab] OR "adverse event*"[tiab] OR "postoperative complication*"[tiab] OR post procedure infection*[tiab] OR "symptomatic complaint*"[tiab] OR "physical complaint*"[tiab] OR discomfort*[tiab] OR transmural burn syndrome[tiab] OR Postpolypectomy syndrome[tiab] OR "gastrointestinal event*"[tiab] OR "cardiopulmonary event*"[tiab] OR gas explosion[tiab] OR intracolonic explosion[tiab] OR perforation[tiab] OR perforations[tiab] OR "colon injury"[tiab] OR "physical symptom*"[tiab] OR "physical discomfort*"[tiab] OR "physical harm*"[tiab] OR "colonoscopy burden" OR "intraoperative bleeding"[tiab] OR "postoperative bleeding"[tiab] OR "intraoperative haemorrhage"[tiab] OR "postoperative haemorrhage"[tiab]

Embase Emtree

1. early diagnosis.sh.
2. early cancer diagnosis.sh.
3. cancer prevention.sh.
4. secondary prevention.sh.
5. prophylactic surgical procedure.sh.
6. primary prevention.sh.
7. screening test.sh.
8. early intervention.sh.

9. mass screening.sh.

10. screening.sh.

11. rescreening.sh.

12. cancer screening.sh.

13. 1 or 2 or 3 or 4 or 5 or 6 or 7 or 8 or 9 or 10 or 11 or 12

14. (colonoscopy or colonoscope or flexible colonoscope).sh.

15. (sigmoidoscope or flexible sigmoidoscope or rigid sigmoidoscope).sh.

16. (sigmoidoscopy or occult blood).sh.

17. exp occult blood test/

18. exp polypectomy/

19. exp endoscopic biopsy/

20. exp endoscopic mucosal resection/

21. exp endoscopic polypectomy/

22. gastrointestinal endoscopy/

23. gastrointestinal biopsy/

24. rectum biopsy/

25. intestine biopsy/

26. colon biopsy/

27. 14 or 15 or 16 or 17 or 18 or 19 or 20 or 21 or 22 or 23 or 24 or 25 or 26

28. 13 and 27

29. cecum tumor/

30. cecum cancer/

31. cecum carcinoma/

32. colon tumor/

33. colon cancer/

34. colon carcinoma/

35. colon adenoma/

36. colon polyp/

37. colon adenocarcinoma/

38. colorectal tumor/

39. colorectal cancer/

40. colorectal carcinoma/

41. colorectal polyp/

42. colorectal adenoma/

43. intestine tumor/

44. intestine cancer/

45. intestine carcinoma/

46. intestine polyp/

47. rectum tumor/

48. rectum cancer/

49. rectum carcinoma/

50. rectum polyp/

51. rectum adenoma/

52. large intestine tumor/

53. large intestine cancer/

54. sigmoid carcinoma/

55. gastrointestinal tumor/

56. gastrointestinal carcinoma/

57. digestive system tumor/

58. digestive system cancer/

59. 29 or 30 or 31 or 32 or 33 or 34 or 35 or 36 or 37 or 38 or 39 or 40 or 41 or 42 or 43 or 44 or 45 or 46 or 47 or 48 or 49 or 50 or 51 or 52 or 53 or 54 or 55 or 56 or 57 or 58

60. 13 and 59

61. laxative/

62. exp laxative/

63. exp intestine contraction stimulating agent/

64. intestine preparation/

65. 61 or 62 or 63 or 64

66. 13 and 65

67. 28 or 60 or 66

68. colonoscope/am [Adverse Device Effect]

69. gastrointestinal endoscopy/ae [Adverse Drug Reaction]

70. sigmoidoscope/am [Adverse Device Effect]

71. sigmoidoscopy/ae [Adverse Drug Reaction]

72. early diagnosis/ae [Adverse Drug Reaction]

73. prophylaxis/co [Complication]

74. mass screening/ae [Adverse Drug Reaction]

75. endoscopic surgery/ae [Adverse Drug Reaction]

76. anesthesia complication/

77. lung complication/

78. disease exacerbation/

79. infection complication/

80. infectious complication/

81. neurological complication/

82. hemoperitoneum/

83. mucosal bleeding/

84. operative blood loss/

85. postoperative hemorrhage/

86. peroperative complication/

87. postoperative complication/

88. postoperative cognitive dysfunction/

89. postoperative delirium/

90. postoperative ileus/

91. postoperative infection/

92. exp "postoperative nausea and vomiting"/

93. postoperative pain/

94. postoperative thrombosis/

95. iatrogenic disease/

96. colon perforation/

97. intestine perforation/

98. rectum perforation/

99. large intestine perforation/

100. digestive system perforation/

101. medical error/

102. surgical error/

103. therapeutic error/

104. abdominal discomfort/

105. abdominal cramp/

106. bloating/

107. epigastric discomfort/

108. abdominal bleeding/

109. patient harm/

110. patient risk/

111. harm reduction/

112. operative blood loss/

113. exp abdominal pain/

114. 68 or 69 or 70 or 71 or 72 or 73 or 74 or 75 or 76 or 77 or 78 or 79 or 80 or 81 or 82 or 83 or 84 or 85 or 86 or 87 or 88 or 89 or 90 or 91 or 92 or 93 or 94 or 95 or 96 or 97 or 98 or 99 or 100 or 101 or 102 or 103 or 104 or 105 or 106 or 107 or 108 or 109 or 110 or 111 or 112 or 113

115. laxative/ae, to [Adverse Drug Reaction, Drug Toxicity]

116. intestine contraction stimulating agent/ae [Adverse Drug Reaction]

117. 114 or 115 or 116

118. 67 and 117

119. limit 118 to yr="2017 -Current"

Embase keyword

1. early detection of cancer.tw.

2. screening.tw.

3. population screening.tw.

4. health screening.tw.

5. early diagnosis.tw.

6. early cancer diagnosis.tw.

7. early detection of cancer.tw.

8. cancer prevention*.tw.

9. secondary prevention.tw.

10. preventive therap*.tw.

11. preventive treatment*.tw.

12. prophylactic treatment*.tw.

13. prophylactic therap*.tw.

14. prophylactic surgical procedure*.tw.

15. primary prevention.tw.

16. cancer screening.tw.

17. screening test*.tw.

18. Early Medical Intervention*.tw.

19. mass screening.tw.

20. rescreening.tw.

21. 1 or 2 or 3 or 4 or 5 or 6 or 7 or 8 or 9 or 10 or 11 or 12 or 13 or 14 or 15 or 16 or 17 or 18 or 19 or 20

22. purgativ*.tw.

23. laxativ*.tw.

24. cathartic*.tw.

25. bowel prepar*.tw.

26. intestine prepar*.tw.

27. 22 or 23 or 24 or 25 or 26

28. colonoscop*.tw.

29. sigmoidoscop*.tw.

30. occult blood.tw.

31. bowel biopsy.tw.

32. colonic biopsy.tw.

33. intestinal biopsy.tw.

34. sigmoid biopsy.tw.

35. occult blood test.tw.

36. polypectomy.tw.

37. endoscopic biopsy.tw.

38. endoscopic mucosal resection.tw.

39. endoscopic polypectomy.tw.

40. gastrointestinal biopsy.tw.

41. rectum biopsy.tw.

42. intestine biopsy.tw.

43. colon biopsy.tw.

44. 28 or 29 or 30 or 31 or 32 or 33 or 34 or 35 or 36 or 37 or 38 or 39 or 40 or 41 or 42 or 43

45. 21 and 27

46. bowel tumor.tw.

47. bowel tumour.tw.

48. bowel cancer.tw.

49. bowel carcinoma.tw.

50. bowel polyp.tw.

51. bowel adenoma.tw.

52. bowel adenocarcinoma.tw.

53. bowel neoplasm.tw.

54. cecum tumour.tw.

55. cecum polyp.tw.

56. cecum adenoma.tw.

57. cecum adenocarcinoma.tw.

58. cecum neoplasm.tw.

59. caecal tumor.tw.

60. caecal tumour.tw.

61. caecal cancer.tw.

62. caecal carcinoma.tw.

63. caecal polyp.tw.

64. caecal adenoma.tw.

65. caecal adenocarcinoma.tw.

66. ceacal neoplasm.tw.

67. cecal tumor.tw.

68. cecal tumour.tw.

69. cecal cancer.tw.

70. cecal carcinoma.tw.

71. cecal polyp.tw.

72. cecal adenoma.tw.

73. cecal adenocarcinoma.tw.

74. cecal neoplasm.tw.

75. coecum tumor.tw.

76. coecum tumour.tw.

77. coecum cancer.tw.

78. coecum carcinoma.tw.

79. coecum polyp.tw.

80. coecum adenoma.tw.

81. coecum adenocarcinoma.tw.

82. coecum neoplasm.tw.

83. colon tumour.tw.

84. colon neoplasm.tw.

85. colonic tumor.tw.

86. colonic tumour.tw.

87. colonic cancer.tw.

88. colonic carcinoma.tw.

89. colonic polyp.tw.

90. colonic adenoma.tw.

91. colonic adenocarcinoma.tw.

92. colonic neoplasm.tw.

93. colorectal tumour.tw.

94. colorectal adenocarcinoma.tw.

95. colorectal neoplasm.tw.

96. intestine tumour.tw.

97. intestine adenoma.tw.

98. intestine neoplasm.tw.

99. intestinal tumor.tw.

100. intestinal tumour.tw.

101. intestinal cancer.tw.

102. intestinal carcinoma.tw.

103. intestinal polyp.tw.

104. intestinal adenoma.tw.

105. intestinal adenocarcinoma.tw.

106. intestinal neoplasm.tw.

107. rectum tumour.tw.

108. rectum adenocarcinoma.tw.

109. rectum neoplasm.tw.

110. rectal tumor.tw.

111. rectal tumour.tw.

112. rectal cancer.tw.

113. rectal carcinoma.tw.

114. rectal polyp.tw.

115. rectal adenoma.tw.

116. rectal adenocarcinoma.tw.

117. rectal neoplasm.tw.

118. large intestine tumour.tw.

119. large intestine carcinoma.tw.

120. large intestine polyp.tw.

121. large intestine adenoma.tw.

122. large intestine adenocarcinoma.tw.

123. large intestine neoplasm.tw.

124. large bowel tumor.tw.

125. large bowel tumour.tw.

126. large bowel cancer.tw.

127. large bowel carcinoma.tw.

128. large bowel polyp.tw.

129. large bowel adenoma.tw.

130. large bowel adenocarcinoma.tw.

131. large bowel neoplasm.tw.

132. sigmoid tumor.tw.

133. sigmoid tumour.tw.

134. sigmoid cancer.tw.

135. sigmoid polyp.tw.

136. sigmoid adenoma.tw.

137. sigmoid adenocarcinoma.tw.

138. sigmoid neoplasm.tw.

139. gastrointestinal tumour.tw.

140. gastrointestinal cancer.tw.

141. gastrointestinal polyp.tw.

142. gastrointestinal adenoma.tw.

143. gastrointestinal adenocarcinoma.tw.

144. gastrointestinal neoplasm.tw.

145. digestive system tumour.tw.

146. digestive system carcinoma.tw.

147. digestive system polyp.tw.

148. digestive system adenoma.tw.

149. digestive system adenocarcinoma.tw.

150. digestive system neoplasm.tw.

151. cecum tumor*.tw.

152. cecum cancer*.tw.

153. cecum carcinoma*.tw.

154. colon tumor*.tw.

155. colon cancer*.tw.

156. colon carcinoma*.tw.

157. colon adenoma*.tw.

158. colon polyp*.tw.

159. colon adenocarcinoma*.tw.

160. colorectal tumor*.tw.

161. colorectal cancer*.tw.

162. colorectal carcinoma*.tw.

163. colorectal polyp*.tw.

164. colorectal adenoma*.tw.

165. intestine tumor*.tw.

166. intestine cancer*.tw.

167. intestine carcinoma*.tw.

168. intestine polyp*.tw.

169. rectum tumor*.tw.

170. rectum cancer*.tw.

171. rectum carcinoma*.tw.

172. rectum polyp*.tw.

173. rectum adenoma*.tw.

174. large intestine tumor*.tw.

175. large intestine cancer*.tw.

176. sigmoid carcinoma*.tw.

177. gastrointestinal tumor*.tw.

178. gastrointestinal carcinoma*.tw.

179. digestive system tumor*.tw.

180. digestive system cancer*.tw.

181. 46 or 47 or 48 or 49 or 50 or 51 or 52 or 53 or 54 or 55 or 56 or 57 or 58 or 59 or 60 or 61 or 62 or 63 or 64 or 65 or 66 or 67 or 68 or 69 or 70 or 71 or 72 or 73 or 74 or 75 or 76 or 77 or 78 or 79 or 80 or 81 or 82 or 83 or 84 or 85 or 86 or 87 or 88 or 89 or 90 or 91 or 92 or 93 or 94 or 95 or 96 or 97 or 98 or 99 or 100 or 101 or 102 or 103 or 104 or 105 or 106 or 107 or 108 or 109 or 110 or 111 or 112 or 113 or 114 or 115 or 116 or 117 or 118 or 119 or 120 or 121 or 122 or 123 or 124 or 125 or 126 or 127 or 128 or 129 or 130 or 131 or 132 or 133 or 134 or 135 or 136 or 137 or 138 or 139 or 140 or 141 or 142 or 143 or 144 or 145 or 146 or 147 or 148 or 149 or 150 or 151 or 152 or 153 or 154 or 155 or 156 or 157 or 158 or 159 or 160 or 161 or 162 or 163 or 164 or 165 or 166 or 167 or 168 or 169 or 170 or 171 or 172 or 173 or 174 or 175 or 176 or 177 or 178 or 179 or 180

182. 21 and 181

183. 44 or 45 or 182

184. intraoperative complication*.tw.

185. (postoperative nausea and vomiting).tw.

186. bowel perforation.tw.

187. cecum perforation.tw.

188. colonic perforation.tw.

189. intestinal perforation.tw.

190. sigmoid perforation.tw.

191. gastrointestinal perforation.tw.

192. extracolonic finding*.tw.

193. Postpolypectomy syndrome.tw.

194. transmural burn syndrome.tw.

195. Harms of colonoscopy.tw.

196. Harms of sigmoidoscopy.tw.

197. Harms of bowel preparation.tw.

198. physical harm*.tw.

199. procedure related harm*.tw.

200. "anesthesia complication".tw.

201. lung complication*.tw.

202. infectious complication*.tw.

203. neurological complication*.tw.

204. hemoperitoneum.tw.

205. mucosal bleeding.tw.

206. operative blood loss.tw.

207. postoperative hemorrhage.tw.

208. peroperative complication*.tw.

209. postoperative complication*.tw.

210. postoperative cognitive dysfunction.tw.

211. postoperative delirium.tw.

212. postoperative ileus.tw.

213. postoperative infection*.tw.

214. postoperative pain.tw.

215. postoperative thrombosis.tw.

216. iatrogenic disease.tw.

217. colon perforation.tw.

218. intestine perforation.tw.

219. rectum perforation.tw.

220. large intestine perforation.tw.

221. digestive system perforation.tw.

222. medical error.tw.

223. surgical error.tw.

224. therapeutic error.tw.

225. abdominal cramp.tw.

226. epigastric discomfort.tw.

227. abdominal bleeding.tw.

228. patient harm.tw.

229. patient risk.tw.

230. harm reduction.tw.

231. surgical blood loss.tw.

232. 184 or 185 or 186 or 187 or 188 or 189 or 190 or 191 or 192 or 193 or 194 or 195 or 196 or 197 or 198 or 199 or 200 or 201 or 202 or 203 or 204 or 205 or 206 or 207 or 208 or 209 or 210 or 211 or 212 or 213 or 214 or 215 or 216 or 217 or 218 or 219 or 220 or 221 or 222 or 223 or 224 or 225 or 226 or 227 or 228 or 229 or 230 or 231

233. 183 and 232

234. limit 233 to yr="2017 - 2022"

Cinahl

<u>Index search:</u>

S127 S76 AND S126

S126 S98 AND S125

S125 S99 OR S100 OR S101 OR S102 OR S103 OR S104 OR S105 OR S106 OR S107 OR S108 OR S109 OR S110 OR S111 OR S112 OR S113 OR S114 OR S115 OR S116 OR S117 OR S118 OR S119 OR S120 OR S121 OR S122 OR S123 OR S124

S124 (MH "Fluid-Electrolyte Imbalance")

S123 (MH "Treatment Complications, Delayed") OR (MH "Adverse Health Care Event")

S122 (MH "Safety")

S121 (MH "Health Care Errors") OR (MH "Treatment Errors") OR (MH "Medication Errors")

S120 (MH "Iatrogenic Disease")

S119 (MH "Abdominal Injuries")

S118 (MH "Intestinal Perforation")

S117 (MH "Ulcer")

S116 (MH "Shock") OR (MH "Toxic Shock Syndrome") OR (MH "Shock, Septic") OR (MH "Shock, Hemorrhagic") OR (MH "Shock, Traumatic") OR (MH "Shock, Surgical") OR (MH "Shock, Cardiogenic")

S115 (MH "Inflammation")

S114 (MH "Hypersensitivity")

S113 (MH "Myocardial Ischemia")

S112 (MH "Heart Diseases")

S111	(MH "Gastrointestinal Hemorrhage")
S110	(MH "Blood Loss, Surgical")
S109	(MH "Surgical Wound Infection")
S108	(MH "Infection")
S107	(MH "Signs and Symptoms, Digestive")
S106	(MH "Pain")
S105	(MH "Death, Sudden, Cardiac")
S104	(MH "Death")
S103	(MH "Intraoperative Complications")
S102	(MH "Postoperative Pain")
S101	(MH "Postoperative Hemorrhage")
S100	(MH "Postoperative Complications")
S99	(MH "Treatment Complications, Delayed")
S98	S80 OR S96 OR S97
S97	S86 AND S92
S96	(S93 OR S94) AND (S80 AND S95)
S95	S93 OR S94
S94	laxatives
S93	cathartic
S92	S87 OR S88 OR S89 OR S90 OR S91
S91	intestinal neoplasm
S90	adenoma
S89	colorectal neoplasms
S88	cecal neoplasms
S87	intestinal polyps
S86	S81 OR S82 OR S83 OR S84 OR S85
S85	Preventive Health Services
S84	mass screening or screening
S83	early detection or early diagnosis or early identification

S82 secondary prevention

S81 early diagnosis or early intervention

S80 S77 OR S78 OR S79

S79 Occult Blood

S78 sigmoidoscopy

S77 colonoscopy

Keyword search:

S76 S46 AND S74

S75 S46 AND S74

S74 S47 OR S48 OR S49 OR S50 OR S51 OR S52 OR S53 OR S54 OR S55 OR S56 OR S57 OR S58 OR S59 OR S60 OR S61 OR S62 OR S63 OR S64 OR S65 OR S66 OR S67 OR S68 OR S69 OR S70 OR S71 OR S72 OR S73

S73 postoperative haemorrhage

S72 intraoperative haemorrhage

S71 postoperative bleeding

S70 intraoperative bleeding

S69 colonoscopy burden

S68 physical harm*

S67 physical discomfort*

S66 physical symptom*

S65 colon injury

S64 perforations

S63 perforation

S62 intracolonic explosion

S61 intracolonic explosion

S60 gas explosion

S59 cardiopulmonary event*

S58 gastrointestinal event*

S57 Postpolypectomy syndrome

S56 transmural burn syndrome

S55 transmural burn syndrome

S54 discomfort*

S53 physical complaint*

S52 symptomatic complaint*

S51 post procedure infection*

S50 postoperative complication*

S49 adverse event*

S48 adverse outcome*

S47 intraoperative complication*

S46 S8 OR S21 OR S45

S45 S43 AND S44

S44 S8 OR S14

S43 S22 OR S23 OR S24 OR S25 OR S26 OR S27 OR S28 OR S29 OR S30 OR S31 OR S32 OR S33 OR S34 OR S35 OR S36 OR S37 OR S38 OR S39 OR S40 OR S41 OR S42

S42 intestinal polyp*

S41 cecal neoplasm*

S40 intestinal adenoma*

S39 intestine cancer*

S38 bowel cancer*

S37 colon neoplasm*

S36 colon cancer*

S35 colon polyp*

S34 colon tumor*

S33 colon adenocarcinoma*

S32 colon carcinogenesis

S31 colon carcinoma*

S30 colon adenoma*

S29	colorectal cancer*
S28	colorectal polyp*
S27	colorectal tumor*
S26	colorectal adenocarcinoma*
S25	colorectal carcinogenesis
S24	colorectal carcinoma*
S23	colorectal adenoma*
S22	colorectal neoplasm*
S21	S14 AND S20
S20	S15 OR S16 OR S17 OR S18 OR S19
S19	colon cleans*
S18	bowel prepa*
S17	purgativ*
S16	Cathartic*
S15	laxativ*
S14	S9 OR S10 OR S11 OR S12 OR S13
S13	early intervent*
S12	screen*
S11	prevent*
S10	early diagnos*
S9	early detect*
S8	S1 OR S2 OR S3 OR S4 OR S5 OR S6 OR S7
S7	polypect*
S6	hemoccult*
S5	occult blood
S4	sigmoidoscop*
S3	colonoscop*
S2	fobt
S1	fecal immunochemical test*

PsycInfo

Index search:

S110S97 AND S108Limiters - Publication Year: 2017-2022

S109S97 AND S108

S108S98 OR S99 OR S100 OR S101 OR S102 OR S103 OR S104 OR S105 OR S106 OR S107

S107DE "Patient Safety"

S106DE "Shock"

S105DE "Inflammation"

S104DE "Ischemia"

S103DE "Infectious Disorders"

S102DE "Digestive System Disorders"

S101DE "Heart Disorders"

S100DE "Death and Dying"

S99DE "Pain"

S98DE "Postsurgical Complications"

S97S79 OR S95 OR S96

S96S85 AND S91

S95(S92 OR S93) AND (S79 AND S94)

S94S92 OR S93

S93laxatives

S92cathartic

S91S86 OR S87 OR S88 OR S89 OR S90

S90intestinal neoplasm

S89adenoma

S88colorectal neoplasms

S87cecal neoplasms

S86intestinal polyps

S85S80 OR S81 OR S82 OR S83 OR S84

S84Preventive Health Services

S83mass screening or screening

S82early detection or early diagnosis or early identification

S81secondary prevention

S80early diagnosis or early intervention

S79S76 OR S77 OR S78

S78Occult Blood

S77sigmoidoscopy

S76colonoscopy

Keyword search:

S75 S46 AND S74 Limiters - Published Date: 20170401-20220131

S74 S47 OR S48 OR S49 OR S50 OR S51 OR S52 OR S53 OR S54 OR S55 OR S56 OR S57 OR S58 OR S59 OR S60 OR S61 OR S62 OR S63 OR S64 OR S65 OR S66 OR S67 OR S68 OR S69 OR S70 OR S71 OR S72 OR S73

S73postoperative haemorrhage

S72intraoperative haemorrhage

S71postoperative bleeding

S70intraoperative bleeding

S69colonoscopy burden

S68physical harm*

S67physical discomfort*

S66physical symptom*

S65colon injury

S64perforations

S63perforation

S62intracolonic explosion

S61intracolonic explosion

S60gas explosion

S59cardiopulmonary event*

S58gastrointestinal event*

S57Postpolypectomy syndrome

S56transmural burn syndrome

S55transmural burn syndrome

S54discomfort*

S53physical complaint*

S52symptomatic complaint*

S51post procedure infection*

S50postoperative complication*

S49adverse event*

S48adverse outcome*

S47intraoperative complication*

S46 S8 OR S21 OR S45

S45 S43 AND S44

S44 S8 OR S14

S43 S22 OR S23 OR S24 OR S25 OR S26 OR S27 OR S28 OR S29 OR S30 OR S31 OR S32 OR S33 OR S34 OR S35 OR S36 OR S37 OR S38 OR S39 OR S40 OR S41 OR S42

S42intestinal polyp*

S41cecal neoplasm*

S40intestinal adenoma*

S39intestine cancer*

S38bowel cancer*

S37colon neoplasm*

S36colon cancer*

S35colon polyp*

S34colon tumor*

S33colon adenocarcinoma*

S32colon carcinogenesis

S31colon carcinoma*

S30colon adenoma*

S29colorectal cancer*

S28colorectal polyp*

S27colorectal tumor*

S26colorectal adenocarcinoma*

S25colorectal carcinogenesis

S24colorectal carcinoma*

S23colorectal adenoma*

S22colorectal neoplasm*

S21S14 AND S20

S20 S15 OR S16 OR S17 OR S18 OR S19

S19colon cleans*

S18bowel prepa*

S17purgativ*

S16Cathartic*

S15laxativ*

S14 S9 OR S10 OR S11 OR S12 OR S13

S13early intervent*

S12screen*

S11prevent*

S10early diagnos*

S9early detect*

S8 S1 OR S2 OR S3 OR S4 OR S5 OR S6 OR S7

S7polypect*

S6hemoccult*

S5occult blood

S4sigmoidoscop*

S3colonoscop*

S2fobt

S1fecal immunochemical test*

The Cochrane database

Identical to the Pubmed MeSh and Pubmed keyword search

Appendix 3 – Reasons for exclusion

All studies excluded on full-text level are available here with reasons for exclusion:

https://osf.io/89zxq/?view_only=90247dddb272492bb6af138b5deb3084

Appendix 4 – Data extraction templates

Rules for data extraction, simplifications and interpretations

In case a study did not report a given type of harm, that either the study protocol or the methods section of the publication mentioned would be reported, it was deemed selective reporting bias. Reporting of zero occurrences of a given type of harm was noted as no occurrences in data extraction tables, “0”.

Data on any type of physical harm reported in studies was categorized during data extraction into one of the following categories: Death, perforation, bleeding, CPE, pain, discomfort and other. If authors did not explicitly state that a given type of physical harm was assessed, e.g. death or bleeding, we marked that outcome as “not assessed” in data extraction tables.

Template 1 – Study characteristics

- Study Number (assigned by reviewer)
- Study ID (First author - year)
- Referring to study protocol?
  - YES/NO
- Important deviations from protocol?
  - Free text
- Study limitations concerning harms noted by study authors
  - Free text
- Conclusions concerning harms noted by study authors
  - Free text
- Authors
- Title of publication
- Year of publication
- Funding
  - NR/Industry/Non-Industry/Mixed/Unclear
- Funding - details
  - Free text
- Conflicts of interest
  - Yes/No/Not Reported
- Conflicts of interest - details

    - Free text
- Study design
    - RCTs/Non-Randomized Study
- Study period
    - dd-mm-year - dd-mm-year
- Country/Countries
    - Free text
- Setting - Other
    - Free text
- Population
    - Screening/Mixed
- Exclusion criteria
    - Reported/Not Reported/Noted no exclusion criteria
- Population details
    - Free text
- People invited, N
- People attending, N
- People performing FOBT, N
- FOBT+, N
- People receiving procedure, N
- Procedures performed, N
- Age interval
    - year-year
- Sex distribution
    - % women
- Mean age
- Median age
- Standard deviation
- Sociodemography
    - Reported/Not Reported
- Sociodemography - details
    - Free text

- **CRC screening methods applied**
- FIT/FOBT test
  - Reported/Not Reported/ Not Performed
  - Type, dose, timing
- Bowel prep/Enema
  - Reported/Not Reported/ Not Performed
  - Type, dose, timing
- Anaestehesia (All types of medications before, during and after)
  - Reported/Not Reported/ Not Performed
  - Type, dose, timing
- Procedure
  - Sigmoidoscopy/Colonoscopy/Sigmoidoscopy to colonoscopy/Both/Control group
  - Expertise
- Polypectomy
  - Reported/Not Reported/ Not Performed
  - Details
- Overall definition of the harm domain physical harm
  - Not Reported/Definition

Template 2 – Outcome data

For each unique population of people reviewers noted if the following outcomes were assessed:

- Death
- Perforation
- Bleeding
- Pain
- Discomfort
- Cardiopulmonary
- Other

Any outcome that did not fit into the first six categories were categorized as other. When outcomes were reported as composite outcomes like "complications", serious adverse events etc. these were categorized as other.

<u>The following characteristics were noted by reviewers for all assessed outcomes:</u>

- Definition
  - Free text including any information supplied regarding thresholds, causality limits, severity limits etc.
- Time point(s) of assessment, i.e. when was the outcome assessed
  - Not Reported when outcome was assessed
  - Single time point
  - Composite > 1 time point of assessment, reporting of data summed up
  - Multiple > 1 time point of assessment, data reported per time point
- Follow-up
  - Longest duration of follow up in the event of multiple time points
  - Free text for details
- Outcome assessor
  - Person Performing Procedure, Self Report, Nurse, Research Assistant, Other, Not Reported
  - Free text for details
- Measurement tool
  - Not Reported, Interview, Questionnaire, Register, Other
  - Free text for details
- Analysis
  - Any changes between outcomes assessed and reported
  - Imputations for missing data, simplifications by merging categories or summing up scores, translations or interpretations from narrative material to numbers
- Number of people analyzed
- Number of procedures analyzed
- Effect size
- Consequences of harms
  - Reported/Not Reported
  - Details about consequences of harms (if reported)
- Modifiers assessed
  - Any covariate assessed for modifying the risk of the outcome
- Modifiers with significant association to outcome
  - Any covariate associated with the outcome, having a P-value < 0,05
- Size of effect
  - Any effetcs size estimate like OR, RR etc.

Appendix 5 – GRADE approach

General considerations

In case of downgrading due to other factors than those described below, these are elaborated via footnotes as recommended in the GRADE Handbook [4].

Design

All studies included for the collective outcomes analyses, i.e. excluding the studies presented under *Results: Special cases*, were effectively one-armed studies. In view of this, and the fact that NRSs are likely equal if not better than RCTs to assess the harms of interventions, we judged that results both from NRSs and from RCTs were of low quality as a starting point for assessments. Below, we outline how we assessed the five factors that determines downgrading of the evidence and the three factors used for upgrading the evidence.

Downgrading factors

Risk of bias (1)

Studies with critical risk of bias were not part of the studies assessed via the GRADE approach.
We downgraded the evidence using the following cut-off values for the collective risk of bias across subpopulations that contributed with data for the outcome:

- If there was serious risk of bias in one or more bias domains <25% of study participants
  - 0
- If there was serious risk of bias in one or more bias domains 25-50 % of study participants
  - -1
- If there was serious risk of bias in one or more bias domains >50 % of study participants
  - -2

We used the weighted bias distributions tables for this assessment, thereby attributing more weight to larger studies.

Inconsistency of results (2)

In case of inconsistent results across subpopulations, we looked for plausible explanations using data about screening procedure delivery, study population characteristics and outcome measurement. In case we could not account for inconsistencies, we downgraded the evidence 1 or 2 levels, depending on the size of the inconsistencies. Below, we present the variables used to find plausible explanations for observed inconsistency in results, divided according to the PICOT format.

Population

Country and setting
Sex distribution
Age interval, mean age, median age, sociodemographic information.

Intervention

Expertise of endoscopists
With/without provision of anaesthesia and any details provided about anaesthetics used

Whether polypectomies were done and if so at which rate.

Comparator
Not applicable

Outcome measures
Any differences in outcome definition, measurement method, who measured the outcome and the follow-up time included.

Indirectness of evidence (3)
The eligibility criteria for the review defines the population of interest, the target population. To assess indirectness of evidence, we compared the review's eligibility criteria to characteristics of screening procedure delivery, study population and outcome measurement in included studies. We only accepted small deviations between eligibility criteria and characteristic of included studies; hence, we expected the evidence to be very direct in terms of answering the research question. **Therefore, this GRADE domain was not meaningful to assess**. Eligibility criteria for the review are available in appendix 2. Below, we outline our considerations about the core components of eligible studies that determine their directness/indirectness related to the research question of interest, in the PICOT format.

Population
We had strict criteria for the population of interest. We excluded any study outside of these criteria. We accepted minor deviations, e.g. a small percentage of the study population being above or below the age interval or in case a small part of the study population that was not in mean risk of CRC due to symptoms of CRC or due to any other reason for increased risk of CRC. In case a study population received one or more different screening procedure, e.g. some receiving flexible sigmoidoscopy and other receiving FOBT and colonoscopy, we divided the study population according of the 4 screening procedure groups. As a result, we view all included studies as direct evidence concerning the interventions provided.

Intervention
We exclusively included studies that provided any combination of FOBT, colonoscopy and sigmoidoscopy and no other co-interventions other than those normally associated with these interventions, e.g. bowel preparation, anaesthesia and polypectomy. Following data extraction, we divided all studies according to the 4 types of screening procedures to facilitate homogenous groups. As a result, we view all included studies as direct evidence concerning the interventions provided.

Comparator
Not applicable

Outcome measures
We did not include surrogate outcome measures, e.g. changes in blood pressure, saturation etc. We only included outcomes that were measured systematically. We extracted data concerning how the outcome was defined, how it was measured, by whom and the follow-up time. Following data extraction, we subcategorized the outcomes to create more homogenous outcome groups. As a result, we view all included studies as direct evidence concerning the outcomes analysed.

Time

We did not downgrade the evidence due to the year the studies were conducted, e.g. studies conducted in the 90'ies versus the 2010's, as we expected the rate of adverse events to be constant over time. This is in line with a recent systematic review that found a small decline in the risk of post-colonoscopy bleeding and a stable rate of perforation and mortality due to screening of studies conducted between 2001 to 2015 [5].

Imprecise results (4)
We used the optimal information size (OIS) to assess whether the evidence should be downgraded.

We established the OIS as the number of people needed for 1 event to occur of a given outcome. To establish this estimate, we calculated the weighted mean risk of the outcome across those identified systematic reviews that quantified the outcome with the potential case risk estimate from this review added to the analysis. We added the potential case risk estimate from this review due to the hypothesis that physical harms have been underestimated in former reviews. Because of adding our estimate to the OIS calculation, the OIS becomes smaller, leading to a less strict downgrade criterion than if our results had not been added to the analysis.

For example, if the weighted mean risk of death across other review and the current review was 5/100.000 people, then the optimal information size would be 20.000 people.

For OIS on CPE (CPE) there were no risk estimates in other identified reviews. Therefore, we calculated OIS for each subgroup of CPE as the inverse of the weighted mean for each subgroup of CPE with data collected for the potential case analysis.

Calculations of OIS for death and CPE are available below.

We downgraded the evidence with the following degrees using the criteria outlined below:

1. Do not rate down
    a. < 50 % of studies have a size below the OIS and/or
    b. The 95% confidence interval for the collective outcomes excludes no effect
2. Rate down 1 level
    a. >50 % of studies have a sample size below the OIS and/or
    b. The 95% confidence interval for the collective outcomes overlaps no effect
3. Rate down 2 levels
    a. Very few events, in general < 5 AND
    b. The 95% confidence interval for the collective outcomes overlaps no effect

sTable 2. Optimal Information Size (OIS) for death and CPE

Risk of death								
**Procedure**	**FS**		**TConly**		**FOBT**		**TCfollowup**	
**Procedures per person**	1,222		1,006		1,134		1,029	
**Systematic review**	**Fitzpatrick-Lewis 2016***	**Martiny et al. 2020**	**Fitzpatrick-Lewis 2016**	**Martiny et al. 2020**	**Fitzpatrick-Lewis 2016**	**Martiny et al. 2020**	**Fitzpatrick-Lewis 2016**	**Martiny et al. 2020**
Reporting outcome (0=NO)	1	1	1	1	1	1	0	1
Events	6	6	14	1	10	2		1
Sample	40332	40674	109076	327037	39561	58900		5665
Average/100.000	15	15	13	0,3	25	3	0	18
Total events	12		15		12		1	
Total sample	81006		436113		98461		5665	
Unweighted Average/100.000	15		7		14		18	
Weighted Average/100.000	15		3		12		18	
OIS unweighted	6750		15220		6975		5665	
OIS weighted	**6751**		**29074**		**8205**		**5665**	

*Number reported per procedure were converted to per person via the conversion-factors that were calculated from included studies in this review.

Cardiopulmonary events												
**Procedure**	**FS**			**TConly**			**FOBT**			**TCfollowup**		
**Outcome**	Events	People	OIS*	Events	People	OIS*	Events	People	OIS*	Events	People	OIS*
Acute Coronary Syndrome	2	40674	**20337**	151	89919	**595**	2	13261	**6631**	0	0	**NA**
arrhythmia	0	0	**NA**	649	81261	**125**	2	10277	**5139**	0	0	**NA**
CPE arrest after 30 days	0	0	**NA**	56	78065	**1394**	0	0	**NA**	0	0	**NA**
heart failure	0	0	**NA**	284	78065	**275**	0	0	**NA**	0	0	**NA**
Immediate CPE arrest	0	0	**NA**	0	0	**NA**	0	0	**NA**	0	0	**NA**
stroke	0	0	**NA**	7	11854	**1693**	4	2984	**746**	0	0	**NA**
TE	1	40674	**40674**	1	3196	**3196**	5	23538	**4708**	0	0	**NA**
Vasovagal reaction*	236	58859	**249**	474	93173	**197**	0	0	**NA**	7	775	**111**

Publication bias

Publication bias, i.e. selective publication of studies, is different to selective outcome reporting, which is considered in ROBINS-I, domain 6: "Reporting bias".

Even though we had a comprehensive search strategy, we consider it likely that there is considerable publication bias concerning results both from RCTs and for NRS. This bias could both lead to over- or underestimates of the risk of harm. The following factors likely affects our estimates of harm, given the empirical evidence compiled in the GRADE handbook [4]:

- The event rate for most types of harm is low, leading to an increased risk of insignificant findings, which is often more difficult to publish.
- When the event rate is zero, which is common for rare events in small studies, such as death or serious cardiopulmonary events like cardiac arrest, authors might fail to report this, exclusively reporting those harms that did occur. If this is the case for many studies, our estimates are overestimated.
- Publication bias is more likely in systematic reviews of NRS. With our 75 NRS (77%) included for review, publication bias is likely for all outcomes.
- We include studies that are small and/or investigate a negative health effect, which are both difficult to publish.

For all 4 screening procedure groups, death was assessed in less than 50% of studies (in 41% of studies for once-only colonoscopy). For CPE, all types of events were analysed in less than 30% of included studies. Since there are no formal demands to assess all types of adverse events and no prior published protocols for NRS, it is not possible to know whether any of the outcomes of interest to this review have been investigated, but not published.

In view of these characteristics of the evidence at hand, and the many pitfalls of using funnel plots and other statistics to assess publication bias [6, 7], we chose a pragmatic solution and downgraded all analyses 1 level due to probable publication bias.

Upgrading factors

Large magnitude of effect

We choose to upgrade the evidence grade either 1 or 2 levels in case the following criteria were satisfied:

1. large magnitude of effect
2. adequate precision of the effect size
3. reason to believe the outcome was caused by screening and no other factors (small risk of confounding)

Large magnitude of effect and adequate precision of the effect size

To our knowledge, there is no agreed-upon threshold to judge when the effect size concerning physical harms related to screening is large, neither when the precision of the effects size estimate is adequate. Therefore, we took a pragmatic approach towards setting thresholds.

To judge whether the evidence should be upgraded due to large effects, we used the table below.

sTable 3. Upgrading factor level

	Effect size > 1 event / 100 people	Effect size > 1 event / 1000 people
CI ≥ 50% of the effects estimate	+2	+1
CI ≥ 33% of the effects estimate	+1	0
CI: Confidence interval		

Small risk of confounding

We did not consider this criterion, because the trials were effectively one-armed and thus the risk of confounding will always be considerable.

Effect of plausible residual confounding

Due to trials being one-armed, we did not consider this domain.

Dose-response gradient

Due to poor reporting in trials we could not assess the dose of screening, i.e. how many times an individual was screened e.g. due to inadequate bowel cleansing, pain etc. In effect, we have to treat screening as a one-time event in all trials with a constant dose. Therefore, it was not possible to assess any dose-response relationships between the dose of screening and the risk of harm.

Appendix 6 – Subcategories of CPEs

We used the following information from studies to subcategorise each type of harm: The definition of harm associated with CRCSPs in the publication, any defining information about the specific type of harm, e.g. details about “what counted” as a perforation, the follow-up time and any information about the consequences of harm. If any type of harm led to fatal consequences, we added the number of deaths as a separate outcome for that study.

sTable 4: Assessments of CPEs across the four screening interventions

	FS	TConly	TCfobt	TCfollowup	All procedures	Proportion, %
ACS short-term	0	3	1	0	4	4%
arrhythmia short-term	0	1	1	0	2	2%
heart failure short-term	0	0	1	0	1	1%
Pulmonary event short-term	0	4	1	0	5	4%
stroke short-term	0	2	1	0	3	3%
TE short-term	0	1	1	0	2	2%
Vasovagal reaction short-term	2	2	0	1	5	4%
ACS long-term	2	8	3	0	13	12%
arrhythmia long-term	0	6	2	1	9	8%
heart failure long-term	0	4	0	0	4	4%
Pulmonary event long-term	0	2	1	0	3	3%
stroke long-term	0	5	1	0	6	5%
TE long-term	0	2	4	0	6	5%
Vasovagal reaction long-term	0	3	1	0	4	4%
Follow-up time NR	6	3	3	2	14	12%

other	1	10	1	1	13	12%
NDCPE	2	11	5	1	19	17%
TOTAL	13	67	27	6	113	100%

sTable 5: Subcategories of CPEs

Study ID	Design	Procedure	Outcome	Definition	Analysis category	Followup	Combined analysis category
Levin 2002	NRS	FS	Cardiopulmonary	acute myocardial infarction	ACS	28 days	ACS long-term
Nelson 2002	NRS	TConly	Cardiopulmonary	Myocardial infarction	ACS	30 days	ACS long-term
Denis 2013	NRS	TCfobt	Cardiopulmonary	myocardial infarction	ACS	30 days	ACS long-term
Saraste 2016	NRS	TCfobt	Cardiopulmonary	unstable angina pectoris	ACS	30 days	ACS long-term
Berhane 2009	NRS	TConly	Cardiopulmonary	postop myocardial infarction	ACS	30 days	ACS long-term
Stoop 2012	RCT	TConly	Cardiopulmonary	Acute coronary syndrome or MI	ACS	30 days	ACS long-term
Stock 2013	NRS	TConly	Cardiopulmonary	Myocardial infarction	ACS	30 days	ACS long-term
Atkin 2002	RCT	FS	Cardiopulmonary	acute myocardial infarction	ACS	30 days	ACS long-term
Garcia-Albeniz 2017	NRS	TConly	Cardiopulmonary	Myocardial infarction or angina	ACS	30 days	ACS long-term
Garcia-Albeniz 2017	NRS	TConly	Cardiopulmonary	Myocardial infarction or angina	ACS	30 days	ACS long-term
Wang 2018a	NRS	TConly	Cardiopulmonary	Acute myocardial infarction	ACS	30 day'	ACS long-term
Causada-Calo 2020	NRS	TConly	Cardiopulmonary	myocardial infarction	ACS	30 days	ACS long-term

Denis 2021	NRS	TCfobt	Cardiopulmonary	myocardial infarction	ACS	30 days	ACS long-term
Pedersen 2020	NRS	TConly	Cardiopulmonary	Myocardial infarction	ACS	8 days	ACS short-term
Taleban 2018	NRS	TConly	Cardiopulmonary	Myocardial infarction during or immediately after the procedure	ACS	Until discharge	ACS short-term
Tomaszewski 2021	NRS	TCfobt	Cardiopulmonary	acute coronary syndrome	ACS	0-14 days	ACS short-term
Lieberman 2000	NRS	TConly	Cardiopulmonary	myocardial infarction	ACS	During	ACS short-term
Nelson 2002	NRS	TConly	Cardiopulmonary	Atrial fibrillation	arrhythmia	30 days	arrhythmia long-term
Denis 2013	NRS	TCfobt	Cardiopulmonary	dysrhythmia	arrhythmia	30 days	arrhythmia long-term
Castro 2013	NRS	TCfollowup	Cardiopulmonary	selflimited supraventricular tachycardia.	arrhythmia	30 days	arrhythmia long-term
Stoop 2012	RCT	TConly	Cardiopulmonary	Atrial fibrillation	arrhythmia	30 days	arrhythmia long-term
Garcia-Albeniz 2017	NRS	TConly	Cardiopulmonary	Arrhythmia	arrhythmia	30 days	arrhythmia long-term
Garcia-Albeniz 2017	NRS	TConly	Cardiopulmonary	Arrhythmia	arrhythmia	30 days	arrhythmia long-term
Wang 2018a	NRS	TConly	Cardiopulmonary	Cardiac dysrhytmia	arrhythmia	30 day'	arrhythmia long-term
Causada-Calo 2020	NRS	TConly	Cardiopulmonary	Cardiac arrhythmia	arrhythmia	30 days	arrhythmia long-term
Denis 2021	NRS	TCfobt	Cardiopulmonary	cardiac dysrhythmia secondary to hypokalemia caused by the bowel preperation	arrhythmia	30 days	arrhythmia long-term
Pedersen 2020	NRS	TConly	Cardiopulmonary	Arrhythmia	arrhythmia	8 days	arrhythmia short-term
Tomaszewski 2021	NRS	TCfobt	Cardiopulmonary	arrythmia	arrhythmia	0-14 days	arrhythmia short-term
Gupta 2012	NRS	TCfobt	Cardiopulmonary	MI after postpolypectomy bleeding	ACS	Not Reported	Follow-up time NR
Gupta 2012	NRS	TCfobt	Cardiopulmonary	Hypokalaemia-induced atrial fibrillation	arrhythmia	Not Reported	Follow-up time NR
Taupin 2006	NRS	TConly	Cardiopulmonary	Vasovagal episode	Vasovagal reaction	Not Reported	Follow-up time NR

Duku 2014	NRS	FS	Cardiopulmonary	Vasovagal episode	Vasovagal reaction	Not Reported	Follow-up time NR
Van Dam 2013	RCT	TConly	Cardiopulmonary	Vasovagal syncope	Vasovagal reaction	Not Reported	Follow-up time NR
Atkin 1998	RCT	FS	Cardiopulmonary	Vasovagal attack	Vasovagal reaction	Not Reported	Follow-up time NR
Atkin 1998	RCT	FS	Cardiopulmonary	Vasovagal attack	Vasovagal reaction	Not Reported	Follow-up time NR
Lee 2012	NRS	TCfobt	Cardiopulmonary	stroke	stroke	Not Reported	Follow-up time NR
Atkin 2002	RCT	FS	Cardiopulmonary	Fainted or had a vasovagal episode	Vasovagal reaction	Not Reported	Follow-up time NR
Segnan 2005	RCT	FS	Cardiopulmonary	Mild vagal reaction	Vasovagal reaction	Not Reported	Follow-up time NR
Segnan 2005	RCT	TCfollowup	Cardiopulmonary	Mild vagal reaction	Vasovagal reaction	Not Reported	Follow-up time NR
Gondal 2003	RCT	FS	Cardiopulmonary	Vasovagal events	Vasovagal reaction	Not Reported	Follow-up time NR
Gondal 2003	RCT	TCfollowup	Cardiopulmonary	Vasovagal events	Vasovagal reaction	Not Reported	Follow-up time NR
Xirasagar 2020	NRS	TConly	Cardiopulmonary	aspiration	Pulmonary event	Not Reported	Follow-up time NR
Garcia-Albeniz 2017	NRS	TConly	Cardiopulmonary	Congestive heart failure	heart failure	30 days	heart failure long-term
Garcia-Albeniz 2017	NRS	TConly	Cardiopulmonary	Congestive heart failure	heart failure	30 days	heart failure long-term
Wang 2018a	NRS	TConly	Cardiopulmonary	Congestive heart failure	heart failure	30 day'	heart failure long-term
Causada-Calo 2020	NRS	TConly	Cardiopulmonary	heart failure	heart failure	30 days	heart failure long-term
Hughes 2005	NRS	TCfobt	Cardiopulmonary	heart failure from colonoscopy preparation;	heart failure	14 days	heart failure short-term
Khalid-de Bakker 2011	NRS	TConly	Cardiopulmonary	Major cardiopulmonary complications (e.g. symptomatic myocardial ischaemia or dysrhythmias)	NDCPE	30 days	NDCPE
Khalid-de Bakker 2011b	NRS	TConly	Cardiopulmonary	ND	NDCPE	Not Reported	NDCPE

Bokemeyer 2009	NRS	TConly	Cardiopulmonary	recorded but left to the investigator s decision, which he assumed to be a complication.	NDCPE	Not Reported	NDCPE
Crispin 2009	NRS	TConly	Cardiopulmonary	ND	NDCPE	Not Reported	NDCPE
Shroff 2015	NRS	TCfollowup	Cardiopulmonary	ND	NDCPE	30 days	NDCPE
Sieg 2006	NRS	TConly	Cardiopulmonary	ND	NDCPE	During	NDCPE
Pox 2012	NRS	TConly	Cardiopulmonary	cardiopulmonary events controlled at time of endoscopy not requiring hospitalization	NDCPE	Not Reported	NDCPE
Pox 2012	NRS	TConly	Cardiopulmonary	cardiopulmonary events requiring hospitalization	NDCPE	Not Reported	NDCPE
Tepes 2017	NRS	TCfobt	Cardiopulmonary	ND	NDCPE	Not Reported	NDCPE
Atkin 2002	RCT	FS	Cardiopulmonary	probable pulmonary embolism 5 days after stopping warfarin treatment so that a rectal polyp could be removed	NDCPE	30 days	NDCPE
Waldmann 2016	NRS	TConly	Cardiopulmonary	ND	NDCPE	Not Reported	NDCPE
Kozbial 2015	NRS	TConly	Cardiopulmonary	ND	NDCPE	Not Reported	NDCPE
Ferlitsch 2011	NRS	TConly	Cardiopulmonary	ND	NDCPE	Not Reported	NDCPE
Senore 2011	RCT	FS	Cardiopulmonary	ND	NDCPE	30 days	NDCPE
Senore 2011	RCT	TConly	Cardiopulmonary	ND	NDCPE	30 days	NDCPE
Garcia-Albeniz 2017	NRS	TConly	Cardiopulmonary	Cardiac or respiratory arrest	NDCPE	30 days	NDCPE
Garcia-Albeniz 2017	NRS	TConly	Cardiopulmonary	Cardiac or respiratory arrest	NDCPE	30 days	NDCPE
Benazzato 2020	NRS	TCfobt	Cardiopulmonary	cardiovascular events (not further defined).	NDCPE	30 days	NDCPE
Robertson 2019	RCT	TCfobt	Cardiopulmonary	ND	NDCPE	30-45 days	NDCPE
Causada-Calo 2020	NRS	TConly	Cardiopulmonary	Miscellanea	NDCPE	30 days	NDCPE
Dominitz 2019	RCT	TCfobt	Cardiopulmonary	NR	NDCPE	30 days	NDCPE

Hsu 2020	NRS	TCfobt	Cardiopulmonary	cardiopulmonary events such as hypoxia, aspiration pneumonia, and cardiac arrhythmias.	NDCPE	14 days	NDCPE
Nelson 2002	NRS	TConly	Cardiopulmonary	Thrombophlebitis requiring hospitlization (Major)	other	30 days	other
Castro 2013	NRS	TCfollowup	Cardiopulmonary	symptoms of chest pain and shortness of breath.	other	30 days	other
Segnan 2005	RCT	FS	Cardiopulmonary	Severe vagal reaction and cardiac arrest	other	During	other
Pedersen 2020	NRS	TConly	Cardiopulmonary	heart arrest	other	8 days	other
Tomaszewski 2021	NRS	TCfobt	Cardiopulmonary	abdominal aortic dissection	other	0-14 days	other
Wang 2018a	NRS	TConly	Cardiopulmonary	Cardiac structural	other	30 day'	other
Causada-Calo 2020	NRS	TConly	Cardiopulmonary	acute kidney injury	other	30 days	other
Causada-Calo 2020	NRS	TConly	Cardiopulmonary	Endocarditis	other	30 days	other
Causada-Calo 2020	NRS	TConly	Cardiopulmonary	Valvular disease	other	30 days	other
Lieberman 2000	NRS	TConly	Cardiopulmonary	thrombophlebitis	other	During	other
Stoop 2012	RCT	TConly	Cardiopulmonary	Pneumonia	Pulmonary event	30 days	Pulmonary event long-term
Wang 2018a	NRS	TConly	Cardiopulmonary	Bronchitis and pneumonia	Pulmonary event	30 day'	Pulmonary event long-term
Denis 2021	NRS	TCfobt	Cardiopulmonary	aspiration pneumonia	Pulmonary event	30 days	Pulmonary event long-term
Pedersen 2020	NRS	TConly	Cardiopulmonary	Pneumonia	Pulmonary event	8 days	Pulmonary event short-term
Tomaszewski 2021	NRS	TCfobt	Cardiopulmonary	Pneumonia	Pulmonary event	0-14 days	Pulmonary event short-term
Wang 2018b	NRS	TConly	Cardiopulmonary	Aspiration pneumonia	Pulmonary event	7 days	Pulmonary event short-term

Wang 2018b	NRS	TConly	Cardiopulmonary	Infections of respiratory system	Pulmonary event	7 days	Pulmonary event short-term
Wang 2018b	NRS	TConly	Cardiopulmonary	Pneumonia	Pulmonary event	7 days	Pulmonary event short-term
Nelson 2002	NRS	TConly	Cardiopulmonary	Stroke	stroke	30 days	stroke long-term
Saraste 2016	NRS	TCfobt	Cardiopulmonary	Stroke	stroke	30 days	stroke long-term
Stoop 2012	RCT	TConly	Cardiopulmonary	Cerebrovascular accident	stroke	30 days	stroke long-term
Stock 2013	NRS	TConly	Cardiopulmonary	Stroke	stroke	30 days	stroke long-term
Wang 2018a	NRS	TConly	Cardiopulmonary	Ischaemic or hemorrhagic stroke	stroke	30 day'	stroke long-term
Wang 2018a	NRS	TConly	Cardiopulmonary	transient ischaemic attack	stroke	30 day'	stroke long-term
Pedersen 2020	NRS	TConly	Cardiopulmonary	Cerebrovascular event	stroke	8 days	stroke short-term
Tomaszewski 2021	NRS	TCfobt	Cardiopulmonary	cerebrovascular event	stroke	0-14 days	stroke short-term
Lieberman 2000	NRS	TConly	Cardiopulmonary	cerebrovascular accident	stroke	During	stroke short-term
Nelson 2002	NRS	TConly	Cardiopulmonary	Thrombosed carotid-subclavian bypass (Major)	TE	30 days	TE long-term
Denis 2013	NRS	TCfobt	Cardiopulmonary	Deep vein thrombosis	TE	30 days	TE long-term
Denis 2013	NRS	TCfobt	Cardiopulmonary	pulmonary embolism	TE	30 days	TE long-term
Saraste 2016	NRS	TCfobt	Cardiopulmonary	Pulmonary embolism	TE	30 days	TE long-term
Causada-Calo 2020	NRS	TConly	Cardiopulmonary	thromboembolic disease	TE	30 days	TE long-term
Denis 2021	NRS	TCfobt	Cardiopulmonary	pulmonary embolism	TE	30 days	TE long-term
Pedersen 2020	NRS	TConly	Cardiopulmonary	1 deep venous thrombosis and 4 pulmonary embolus	TE	8 days	TE short-term
Tomaszewski 2021	NRS	TCfobt	Cardiopulmonary	thromboembolic event	TE	0-14 days	TE short-term

Nelson 2002	NRS	TConly	Cardiopulmonary	Vasovagal events	Vasovagal reaction	30 days	Vasovagal reaction long-term
Garcia-Albeniz 2017	NRS	TConly	Cardiopulmonary	Syncope: Hypotension/shock	Vasovagal reaction	30 days	Vasovagal reaction long-term
Garcia-Albeniz 2017	NRS	TConly	Cardiopulmonary	Syncope: Hypotension/shock	Vasovagal reaction	30 days	Vasovagal reaction long-term
Ibánez 2018	NRS	TCfobt	Cardiopulmonary	severe vasovagal comlications	Vasovagal reaction	30 day	Vasovagal reaction long-term
Bretthauer 2016	RCT	TConly	Cardiopulmonary	Vasovagal reaction	Vasovagal reaction	During	Vasovagal reaction short-term
Pabby 2005	NRS	FS	Cardiopulmonary	Vasovagal episode	Vasovagal reaction	During	Vasovagal reaction short-term
Segnan 2002	RCT	TCfollowup	Cardiopulmonary	Mild vagal reaction	Vasovagal reaction	During	Vasovagal reaction short-term
Segnan 2002	RCT	FS	Cardiopulmonary	Mild vagal reaction	Vasovagal reaction	During	Vasovagal reaction short-term
Pedersen 2020	NRS	TConly	Cardiopulmonary	Syncope	Vasovagal reaction	8 days	Vasovagal reaction short-term

Appendix 7 – Procedures per person for the four screening procedure groups

The table below illustrates the relationship between people screened and procedures performed for each of the four screening procedure groups. Studies included for these analyses are presented below.

sTable 6: Procedures performed per person

FS		TCfobt		TCfollowup		TConly	
People	225563	People	676802	People	24561	People	788341
Procedure	267597	Procedure	800430	Procedure	25185	Procedure	801872
People/procedure	0,8429205	People/procedure	0,8455484	People/procedure	0,9752233	People/procedure	0,9831257
Procedures/people	1,1863515	Procedures/people	1,1826644	Procedures/people	1,0254061	Procedures/people	1,0171639

Appendix 8 - Study characteristics of included studies

sTable7a. Study characteristics of included studies

Abbreviations: Flexible sigmoidoscopy (FS), colonoscopy without prior screening (TConly), colonoscopy following FIT/FOBT (TCfobt), colonoscopy following other types of screening tests (TCfollowup), Cardiopulmonary events assessed (CPE, A), Deaths assessed (Death, A), people screened (people), sociodemography (socdem), Not Reported (NR), Reported (R), Not Performed (NP), Performed (P), Polypectomy (PPT), Follow up time (FUT).

Study ID	Procedure	CPE, A	Death, A	Design	Country	Study period	People	Sex, W%	Age	Mean Age	Socdem	Anaesthesia	Expertise	PPT	Rate
Atkin 1998 [8]	FS	YES	YES	RCT	UK	?? to May 1996	770		55-64		NR	NP	R	P	24%
Atkin 1998 [8]	FS	YES	YES	RCT	UK	?? to May 1996	536		55-64		NR	NP	R	P	21%
Atkin 2002 [9]	FS	YES	YES	RCT	UK	1994-1999	40.674	50%	55-64		NR	P	R	P	24%
Blom 2004 [10]	FS	NO	NO	RCT	Sweden	1996-?	469	52%	59-61		NR	NR	R	NP	
Collett 2000 [11]	FS	NO	NO	NRS	Australia	July 1995 to November 1999	2.605	41%	55-64		NR	NP	R	P	
Duku 2014 [12]	FS	YES	NO	NRS	US	February 2013	453		60		NR	P	R	P	
Eloubeidi 2003 [13]	FS	NO	NO	NRS	US	March, 1998 through February, 2000	3.980	52%	50+	59	R	NP	R	NR	
Forbes 2006 [14]	FS	NO	NO	RCT	Australia	1 February and 31 October 2004.	39		50-69		R	NP	R	P	
Gondal 2003 [15]	FS	YES	NO	RCT	Norway	1999-2000	12.960	51%	50-64		NR	NP	R	P	
Hoff 2009 [16]	FS	NO	NO	RCT	Norway	January 1999 and December 2000.	8.846	51%	55-64	58	NR	NR	NR	P	

Hol 2010a [17]	FS	NO	NO	RCT	Netherlands	November 2006- November 2007.	1.522		50-74		R	NP	R	P	
Holme 2014 [18]	FS	NO	YES	RCT	Norway	January 1, 1999 to December 31 2011	12.955	50%	50-64	57	R	NR	NR	P	
Jain 2002 [19]	FS	NO	YES	NRS	US	November 1995 to February 2001	4.229		50-75+		NR	NR	R	P	
Kewenter 1996 [20]	FS	NO	NO	RCT	Sweden	1990	2.108		60-64		NR	NR	NR	P	
Larsen 2002 [21]	FS	NO	NO	RCT	Norway	3rd January 1999 and 8th February 2000	4.956		55-64		NR	NR	NR	NR	
Levin 2002 [22]	FS	YES	YES	NRS	US	January 1, 1994, and December 31, 1996	107.704	49%	50-79	61	NR	NR	R	P	
Olynyk 1996 [23]	FS	NO	NO	NRS	Australia	July to December 1995	342	41%	55-59		NR	NP	R	NR	
Pabby 2005 [24]	FS	YES	NO	NRS	US	1996-2000	6.968	53%	50-74	58	NR	NR	R	P	
Randel 2021 [25]	FS	NO	YES	RCT	Norway	March 2012- May 2019	36.065	51%	50-74		NR	NR	R	P	
Rasmussen 1999 [26]	FS	NO	NO	RCT	Denmark	September 1992 and November 1995	2.235		50-75		NR	NR	R	NP	
Robb 2012 [27]	FS	NO	NO	NRS	UK	November 2006 and May 2008	1.020		58-59		NR	NP	R	P	
Santavirta 2002 [28]	FS	NO	NO	NRS	Finland	March 1994-May 2000	896	52%	60	60	NR	NR	R	P	
Schoen 2000 [29]	FS	NO	NO	NRS	US	May 30, 1996, and August 30, 1997	1.221	46%	40-70+	62	R	NR	R	NP	
Schoen 2012 [30]	FS	NO	NO	RCT	US	1993 through 2001	67.071		55-74		NR	NR	R	NP	
Segnan 2002 [31]	FS	YES	NO	RCT	Italy	October 1995 - April 1999	9.911	47%	55-64		NR	P	R	P	

Segnan 2005 [32]	FS	YES	NO	RCT	Italy	November 1999 through June 2001	4.523	50%	55-64		NR	P	R	P	
Senore 2011 [33]	FS	YES	NO	RCT	Italy	October/November 2002 and January 2004	1.696	49%	55-64		NR	NP	R	P	
Viiala 2008 [34]	FS	NO	NO	NRS	Australia	July 1995 to July 2005	2.868	41%	52,8–69,6	60	NR	NP	R	P	
Zubarik 2002 [35]	FS	NO	NO	NRS	US	December, 2000 to October, 2001	185	46%	49-84	61	NR	NP	R	NR	
Arana-Arri 2018 [36]	TCfobt	NO	YES	NRS	Spain	NR	36.346		50-69		NR	P	R	P	
Benazzato 2020 [37]	TCfobt	YES	YES	NRS	Italy	01-01-2004 - 15-10-2014	99.674	41%	50-69		NR	P	NR	P	57%
Binefa 2013 [38]	TCfobt	NO	NO	NRS	Spain	June 2006 to July 2013	1.691		50-69		NR	P	R	P	
Blanks 2015 [38]	TCfobt	NO	NO	NRS	UK	August 2006 to January 2012	112.024	39%	59-93	66	NR	NR	R	P	53%
Cheng 2002 [39]	TCfobt	NO	YES	NRS	Taiwan	January 1997 and December 2000	6.266	45%	20-81	47	NR	P	R	NR	
Dancourt 2008 [40]	TCfobt	NO	NO	NRS	France	2005	1.205		50-74		NR	P	R	NR	
Denis 2007 [41]	TCfobt	NO	NO	NRS	France	September 2003 to September 2006	2.303		50-74		NR	NR	R	NR	
Denis 2013 [42]	TCfobt	YES	YES	NRS	France	September 2003 and February 2010	10.024	45%	50-74	63	NR	P	R	P	49%
Denis 2021 [43]	TCfobt	NO	NO	NRS	France	September 2003 to july 2014	17.152	45%	50-74	63	NR	P	R	P	49%
Denis 2021 [43]	TCfobt	YES	YES	NRS	France	May 2015 to january 2018	9.061	40%	50-74	63	NR	P	R	P	65%
Denters 2012 [44]	TCfobt	NO	NO	NRS	Netherlands	2006-2008	491	57%	50-75	61	NR	P	R	NR	

Denters 2013 [45]	TCfobt	NO	NO	NRS	Netherlands	August 2008 until June 2009	373	48%	50-75	63	R	P	R	P	82%
Derbyshire 2018 [46]	TCfobt	NO	YES	NRS	UK	2 august 2006 to 13 march 2014	222.488	40%	60-74	66	NR	NR	R	NR	
Din 2015 [47]	TCfobt	NO	NO	NRS	UK	January 2010-December 2012	38.242	31%	59-93	66	NR	NR	R	P	
Din 2010 [48]	TCfobt	NO	YES	NRS	UK	NR	780	31%	60-69		NR	NR	NR	P	
Dominitz 2019 [49]	TCfobt	YES	NO	RCT	US	NR	14.662		50-75		R	NR	R	NR	
Dyson 2014 [50]	TCfobt	NO	NO	NRS	UK	February 2007 and March 2012	1.098	71%	60-74	66	NR	P	R	NR	
Ellul 2010 [51]	TCfobt	NO	NO	NRS	UK	Start July 2006 and 2 years	1.039		60-69		NR	NR	NR	NR	
Faivre 2004 [52]	TCfobt	NO	NO	NRS	France	1988-1998	1.339		45-75		NR	NR	R	NR	
Florido 2017 [53]	TCfobt	NO	NO	NRS	Spain	January 2015 through December 2016	842		50-69		NR	NR	NR	NR	
Fritzell 2020 [54]	TCfobt	NO	NO	RCT	Sweden	2014-2016	2.417	49%	59-60		NR	P	R	NR	
Garcia 2012 [55]	TCfobt	YES	NO	NRS	Spain	2000-2010	989	43%	50-69		NR	NR	NR	NR	
Ghanouni 2016 [56]	TCfobt	NO	NO	NRS	UK	1. January 2011-31. December 2012	64.152	41%	59-93		R	P	R	P	
Graser 2009 [57]	TCfobt	NO	NO	NRS	Germany	Unclear	311	45%	50-81	61	NR	P	R	P	
Gupta 2012 [58]	TCfobt	YES	YES	NRS	UK	October 2006 to September 2009	1.057		60-75		NR	NR	R	P	
Hsu 2020 [59]	TCfobt	YES	NO	NRS	Taiwan	January 2010 to December 2014	214.955	46%	50–75	60	NR	P	R	P	30%
Hughes 2005 [60]	TCfobt	YES	NO	NRS	Australia	november 2000	92		50-74		NR	NR	NR	P	

Ibánez 2018 [61]	TCfobt	YES	YES	NRS	Spain	1 December 2005 to 31 December 2012	7.467	42%	50-69		NR	NP	NR	NR	
Karlijn 2021 [62]	TCfobt	NO	NO	NRS	Netherlands	01–01– 2016 and 01–01–2019	47.270	41%	61-71		NR	NR	R	P	NR
Kooyker 2021 [63]	TCfobt	NO	YES	NRS	Netherlands	October 2013 and December 2017	158.949	41%	63-70		NR	P	NR	NR	
Lee 2012 [64]	TCfobt	YES	YES	NRS	UK	August 2006 and August 2009	30.829	38%	60-92	66	NR	P	R	P	
Marino 2012 [65]	TCfobt	NO	YES	NRS	Italy	June 2008 to October 2010	1.000	38%	50-69	61	NR	NR	NR	P	
Meulen 2021 [66]	TCfobt	NO	NO	NRS	Netherlands	February 2014 until August 2015	225		NR		NR	NR	R	P	NR
Mikkelsen 2018 [67]	TCfobt	NO	YES	NRS	Denmark	March 3, 2014 to December 31, 2014	14.671	44%	50-74	64	NR	NR	NR	P	55%
Monteiro 2021 [68]	TCfobt	NO	NO	NRS	Portugal	2018- june 2019	682		50-70		NR	NR	NR	NR	
Neely 2013 [69]	TCfobt	NO	NO	NRS	UK	May 2010 and May 2011	178	37%	60-69		NR	NR	R	P	
Parente 2013 [70]	TCfobt	NO	NO	NRS	Italy	September 2005 to December 2009.	3.698		50-69		NR	P	R	P	
Paszat 2020 [71]	TCfobt	NO	NO	NRS	Canada	2008-2017	121.626	46%	50-74		R	P	R	P	42%
Portillo 2018 [72]	TCfobt	NO	YES	NRS	Spain	January 2009 and September 2016	48.759	40%	49-69		NR	P	R	NR	
Quintero 2012 [73]	TCfobt	NO	NO	RCT	Spain	The recruitment period was initiated in June 2009, and the first round finished in June 2011.	663	54%	50-69	59	NR	P	R	NR	
Quyn 2018 [74]	TCfobt	NO	YES	NRS	UK	2007-2014	53.332	42%	50-74		NR	P	R	P	46%

Randel 2021 [25]	TCfobt	NO	NO	RCT	Norway	March 2012-January 2017	6.945	51%	50-74		NR	P	R	P	NA
Robertson 2019 [75]	TCfobt	YES	NO	RCT	NR	NR	17.485		50-75		R	NR	R	P	62%
Robinson 1999 [76]	TCfobt	NO	YES	RCT	UK	January, 1991 to February 1995	1.246		45-74		NR	NR	NR	P	
Rutter 2014 [77]	TCfobt	NO	NO	NRS	UK	August 2006 and January 2012	112.024	39%	59-92	66	NR	NR	R	P	
Saraste 2016 [78]	TCfobt	YES	YES	NRS	Sweden	1 January 2008 to 30 June 2012	2.984		60-69		NR	NR	NR	P	40%
Steele 2004 [79]	TCfobt	NO	NO	NRS	UK	March 2000 - May 2003	4.116		50-69		NR	NR	R	NR	
Steele 2009 [80]	TCfobt	NO	YES	NRS	UK	March 2000 - July 2007	5.265	39%	50-69		NR	NR	R	NR	
Sung 2003 [81]	TCfobt	NO	NO	NRS	China	NR	505	56%	50-79	57	R	P	R	P	29%
Tepes 2017 [82]	TCfobt	YES	NO	NRS	Slovenia	April 2009-March 2011	13.674	44%	50-69		NR	NP	R	P	
Tomaszewski 2021 [83]	TCfobt	YES	YES	NRS	Canada	Nov. 15, 2013, to Dec. 31, 2017	78.831	44%	50-74		NR	NR	R	P	65%
Vanaclocha-Espi 2018 [84]	TCfobt	NO	YES	NRS	Spain	2000-2013	41.204	72%	50-70		NR	P	NR	P	
Yamada 2017 [85]	TCfobt	NO	NO	NRS	Japan	March-October 2015	107	26%	35-84	65	R	NP	R	NR	
Zorzi 2009 [86]	TCfobt	NO	NO	NRS	Italy	2007	29.276		50-69		NR	NR	NR	NR	
Atkin 2002 [9]	TCfollowup	NO	YES	RCT	UK	September 1994.-July 1999.	2.131	50%	55-64		NR	P	R	P	
Castro 2013 [87]	TCfollowup	YES	YES	NRS	US	2005-?	3.215	74%	50-64	56	NR	NR	NR	NR	
Dellon 2009 [88]	TCfollowup	NO	YES	NRS	US	August 2003 and August 2005	3.614	58%	21-90	58	R	P	R	P	44%
Forbes 2006 [14]	TCfollowup	NO	NO	RCT	Australia	1 February and 31 October 2004.	112		50-69		R	NR	R	P	

Gondal 2003 [15]	TCfollowup	YES	NO	RCT	Norway	1999-2000	2.524		50-64		NR	P	NR	P	
Hol 2010a [17]	TCfollowup	NO	NO	RCT	Netherlands	November 2006-November 2007.	332		50-74		R	NR	NR	P	
Holme 2014 [18]	TCfollowup	NO	YES	RCT	Norway	January 1, 1999 to December 31 2011	2.746	50%	50-64	57	NR	NR	NR	P	
Kewenter 1996 [20]	TCfollowup	NO	NO	RCT	Sweden	1990	185		60-64		NR	NR	NR	P	59%
Ko 2010 [89]	TCfollowup	NO	NO	NRS	US	NR	11.692		40+		NR	NR	NR	P	
Mandel 1993 [90]	TCfollowup	NO	NO	RCT	US	1975-1992	11.943		50-80		NR	NR	NR	P	
Naumann 2021 [91]	TCfollowup	NO	NO	NRS	UK	2007 -2019	5.219	40%	NR	66	NR	NR	R	NR	
Polter 2015 [92]	TCfollowup	NO	NO	NRS	US	January 1, 2011, through December 31, 2012	3.059		18-79		NR	NR	R	NR	
Rajendran 2017 [3]	TCfollowup	NO	NO	NRS	UK	NR	4.953		60-74		NR	NR	NR	P	100%
Randel 2021 [25]	TCfollowup	NO	YES	RCT	Norway	March 2012- May 2019	3.297	37%	50-74		NR	P	R	P	
Rasmussen 1999 [26]	TCfollowup	NO	NO	RCT	Denmark	September 1992 and November 1995	490		50-75		NR	NR	NR	P	
Rutter 2012 [93]	TCfollowup	NO	YES	NRS	US	1994-2009	4.984	51%	40-86		NR	NR	NR	NR	
Schoen 2012 [30]	TCfollowup	NO	NO	RCT	US	1993 through 2001	17.234		55-74		NR	NR	NR	NR	
Segnan 2002 [31]	TCfollowup	YES	NO	RCT	Italy	October 1995-April 1999	775	47%	55-64		NR	P	R	P	
Segnan 2005 [32]	TCfollowup	YES	NO	RCT	Italy	November 1999 through June 2001	341		55-64		NR	P	R	P	
Shroff 2015 [94]	TCfollowup	YES	YES	NRS	US	1. july 2011-30. January 2015	6.152	63%	NR	58	R	P	NR	NR	

Ahmed 2016 [4]	TConly	NO	NO	NRS	UK	2007-2014	1.049	43%	60-74	66	NR	P	R	P	56%
Berhane 2009 [95]	TConly	YES	YES	NRS	US	January 2000-December 2006	11.808		50-90	64	NR	P	R	P	
Bielawska 2014 [96]	TConly	NO	NO	NRS	US	January 2000 through March 2011	535.286		18+		NR	P	NR	P	
Bokemeyer 2009 [97]	TConly	YES	YES	NRS	Germany	1 october 2003 to 31 december 2006	264.602	56%	55-99		NR	P	R	P	21%
Bretthauer 2016 [98]	TConly	YES	YES	RCT	Poland, Norway, the Netherlands, and Sweden	08-06-2009-23-06-2014	11.912	48%	55-64		NR	P	R	P	
Bugajski 2017 [99]	TConly	NO	NO	NRS	Poland	2014-2015	22.725		55-64	60	NR	P	R	NR	
Causada-Calo 2020 [100]	TConly	YES	YES	NRS	Canada	April 2008 - September 2017	30.443	50%	50-74	61	NR	P	NR	NP	
Chiu 2013 [101]	TConly	NO	NO	NRS	Taiwan	september 2005 - september 2010	18.296	42%	50+	60	NR	NR	R	P	
Crispin 2009 [102]	TConly	YES	NO	NRS	Germany	2006	55.993	56%	55-97		NR	P	NR	P	29%
Dae 2007 [103]	TConly	NO	NO	NRS	South Korea		4.629	47%	20-70+	48	NR	NR	R	NR	
Ferlitsch 2011 [104]	TConly	YES	YES	NRS	Austria	November 2007 and December 2010	44.350	51%	30-97		NR	P	R	P	95%
Fritzell 2020 [54]	TConly	NO	NO	RCT	Sweden	2014-2016	4.107	49%	59-60		NR	P	R	NR	
Garcia-Albeniz 2017 [105]	TConly	YES	NO	NRS	US	2004-2012	46.872	51%	70-74		R	NR	NR	P	
Garcia-Albeniz 2017 [105]	TConly	YES	NO	NRS	US	2004-2012	31.193	50%	75-79		R	NR	NR	P	

Hamdani 2013 [106]	TConly	NO	NO	NRS	US	January 1, 2002 to August 25, 2010	29.396		18+		NR	NR	NR	NR	
Huppe 2004 [107]	TConly	NO	NO	NRS	Germany	October 2002 and June 2003	1.117	70%	55+	64	NR	P	R	P	12%
Imperiale 2000 [108]	TConly	NO	YES	NRS	US	September 1995 and December 1998.	1.994	41%	50+	60	NR	NR	R	P	
Ionescu 2015 [109]	TConly	NO	NO	NRS	Romania	January 1, 2007 and June 30, 2008<br><br>January 1, 2012 and June 30, 2013.	1.351		50-75		NR	NR	R	NR	
Kaminski 2017 [110]	TConly	NO	NO	NRS	Poland	January 1, 2004, through December 31, 2008.	144.382	63%	50-66	56	R	P	R	P	17%
Khalid-de Bakker 2011 [111]	TConly	YES	NO	NRS	Netherlands	NR	214	61%	50-65	54	R	P	R	P	50%
Khalid-de Bakker 2011b [112]	TConly	YES	NO	NRS	Netherlands	November 2006 and May 2008.	447	61%	50-65	55	NR	P	R	NR	
Kobiela 2020 [113]	TConly	NO	YES	RCT	Poland	2012-2015	55.390	54%	55-64	59	NR	NR	NR	NR	
Kozbial 2015 [114]	TConly	YES	NO	NRS	Austria	2007 through September 2011	59.901	51%	50+	61	NR	P	R	P	34%
Leventi 2021 [115]	TConly	NO	NO	NRS	Germany	01.01.2018 to 01.01.2020	2.298		NR		NR	NR	NR	P	27%
Lieberman 2000 [116]	TConly	YES	YES	NRS	US	February 1994 and January 1997	3.121	3%	NR	63	R	P	R	NP	
Nelson 2002 [117]	TConly	YES	YES	NRS	US	February 1994 and January 1997	3.196	3%	50-75	63	R	P	R	P	54%
Pedersen 2020 [118]	TConly	YES	YES	NRS	Denmark	1 January 2015 to 31 December 2018	11.163	35%	NR	66	NR	P	NR	P	51%

Pox 2012 [119]	TConly	YES	YES	NRS	Germany	January 2003 to December 2008	2.773.783	56%	55 +	65	NR	P	R	P	
Quintero 2012 [73]	TConly	NO	NO	RCT	Spain	June 2009-??	5.059	53%	50-69	59	NR	P	R	NR	
Regula 2006 [120]	TConly	NO	NO	NRS	Poland	October 2000 to December 2004	50.148	3%	40-66	55	NR	P	NR	P	16%
Rutter 2012 [93]	TConly	NO	YES	NRS	US	1994-2009	38.472	51%	40-85		R	NR	NR	P	47%
Schoenfeld 2005 [121]	TConly	NO	NO	NRS	US	July 1999-December 2002	1.483	100%	40-79	59	R	P	R	P	
Senore 2011 [33]	TConly	YES	NO	RCT	Italy	October/November 2002 and January 2004	1.382	49%	55-64		NR	P	R	NR	
Sieg 2006 [122]	TConly	YES	YES	NRS	Germany	October 2003 to July 2005.	108.133	57%	55-99	64	NR	P	R	P	
Stock 2013 [123]	TConly	YES	YES	NRS	Germany	January 1 2000-December 31 2008	8.658	55%	55-80	66	R	NR	R	P	
Stoop 2012 [124]	TConly	YES	YES	RCT	Netherlands	Invitations were sent between June 8, 2009 and Aug 16, 2010.	1.276	49%	50-74		R	P	R	P	
Strul 2006 [125]	TConly	NO	YES	NRS	Israel	January 1996-July 2003	1.177	53%	40-80	60	NR	NR	NR	NR	
Taleban 2018 [126]	TConly	YES	NO	NRS	US	Maj - July 2017	99	52%	>=50	58	NR	P	R	NR	
Taupin 2006 [127]	TConly	YES	NO	NRS	Australia	May 2002-January 2004.	231	48%	55-74	62	NR	P	R	P	45%
Van Dam 2013 [128]	TConly	YES	NO	RCT	Netherlands	Invitation: 16 February-27 May 2010	322	48%	50-74		R	P	R	NR	
Waldmann 2016 [129]	TConly	YES	YES	NRS	Austria	January 2007 and December 2014,	159.246	49%	50+	61	NR	P	R	P	

Wang 2018a [130]	TConly	YES	YES	NRS	US	from January 1, 2005 through December 31, 2011	645.095	51%	50-80		R	NR	NR	NP	
Wang 2018b [131]	TConly	YES	YES	NRS	US	2014	454.271	57%	40-100		R	P	NR	P	
Wijkerslooth 2012 [132]	TConly	NO	NO	RCT	Netherlands	June 2009 and August 2010,	1.276	50%	50-74		R	P	R	P	
Wong 2017 [133]	TConly	NO	NO	NRS	US	May 2016 to September 2016	833	52%		62	NR	NR	NR	NR	
Xirasagar 2020 [134]	TConly	YES	YES	NRS	US	October 2001 to December 2014	25.008	54%	40-89	58	R	P	R	P	70%
Zubarik 2002 [35]	TConly	NO	NO	NRS	US	December, 2000 to October, 2001	281	56%	28-81	55	NR	P	R	NR	
Zwink 2017 [135]	TConly	NO	YES	NRS	Germany	2010-2013	5.252	52%	NR		NR	NR	NR	NP	

sTable7b. Characteristics of subpopulations with assessment of deaths

Abbreviations: Flexible sigmoidoscopy (FS), colonoscopy without prior screening (TConly), colonoscopy following FIT/FOBT (TCfobt), colonoscopy following other types of screening tests (TCfollowup), Death without reported follow-up time (Death-NRFU), death with follow-up time reported (Death-FUR), people screened (people), Not Reported (NR).

Study ID	Procedure	People	Effect size	Analysis category	Assessed?	Followup
Atkin 1998	FS	770	0	Death-NRFU	Assessed	NR
Atkin 1998	FS	335	1	Death-NRFU	Assessed	NR
Atkin 2002	FS	40674	6	Death-FUR	Assessed	30 days
Holme 2014	FS	12955	0	Death-NRFU	Assessed	NR
Jain 2002	FS	4229	0	Death-NRFU	Assessed	NR
Levin 2002	FS	107704	10	Death-FUR	Assessed	28 days
Randel 2021	FS	36065	0	Death-FUR	Assessed	30 days
Arana-Arri 2018	TCfobt	36346	0	Death-FUR	Assessed	10 days
Benazzato 2020	TCfobt	99674	15	Death-FUR	Assessed	30 days
Cheng 2002	TCfobt	6266	0	Death-NRFU	Assessed	NR
Denis 2013	TCfobt	10024	0	Death-FUR	Assessed	30 days
Denis 2021	TCfobt	9061	0	Death-FUR	Assessed	30 days
Denis 2021	TCfobt	17152	1	Death-FUR	Assessed	30 days
Derbyshire 2018	TCfobt	222488	1	Death-FUR	Assessed	30 days
Din 2017	TCfobt	780	0	Death-NRFU	Assessed	NR
Gupta 2012	TCfobt	1057	1	Death-NRFU	Assessed	NR
Ibánez 2018	TCfobt	7467	0	Death-FUR	Assessed	30 day
Kooyker 2021	TCfobt	158949	48	Death-FUR	Assessed	30 days
Lee 2012	TCfobt	32148	0	Death-NRFU	Assessed	NR
Marino 2012	TCfobt	1000	0	Death-FUR	Assessed	30 days
Mikkelsen 2018	TCfobt	14671	11	Death-FUR	Assessed	>30 days
Portillo 2018	TCfobt	48759	1	Death-FUR	Assessed	30 days
Quyn 2018	TCfobt	53332	1	Death-NRFU	Assessed	NR
Randel 2021	TCfobt	6945	1	Death-FUR	Assessed	30 days
Robinson 1999	TCfobt	1249	0	Death-FUR	Assessed	30 days
Saraste 2016	TCfobt	2984	1	Death-FUR	Assessed	30 days
Steele 2009	TCfobt	5265	0	Death-NRFU	Assessed	NR
Tomaszewski 2021	TCfobt	78831	2	Death-FUR	Assessed	0-14 days
Vanaclocha-Espi 2018	TCfobt	41204	1	Death-FUR	Assessed	30 days

Atkin 2002	TCfollowup	39274	1	Death-FUR	Assessed	30 days
Castro 2013	TCfollowup	3215	0	Death-FUR	Assessed	30 days
Dellon 2009	TCfollowup	3614	0	Death-FUR	Assessed	During
Holme 2014	TCfollowup	2746	0	Death-NRFU	Assessed	NR
Randel 2021	TCfollowup	3297	0	Death-FUR	Assessed	30 days
Rutter 2012	TCfollowup	38472	3	Death-FUR	Assessed	30 days
Shroff 2015	TCfollowup	6152	0	Death-FUR	Assessed	30 days
Berhane 2009	TConly	11808	0	Death-FUR	Assessed	30 days
Bokemeyer 2009	TConly	267579	0	Death-NRFU	Assessed	NR
Bretthauer 2016	TConly	11912	0	Death-FUR	Assessed	30 days
Causada-Calo 2020	TConly	30443	19	Death-FUR	Assessed	30 days
Ferlitsch 2011	TConly	44350	0	Death-NRFU	Assessed	NR
Imperiale 2000	TConly	1994	0	Death-NRFU	Assessed	NR
Kobiela 2020	TConly	55390	11	Death-FUR	Assessed	30 days
Lieberman 2000	TConly	3121	0	Death-FUR	Assessed	30 days
Nelson 2002	TConly	3196	3	Death-FUR	Assessed	30 days
Pedersen 2020	TConly	11163	10	Death-FUR	Assessed	30 days
Pox 2012	TConly	2804983	7	Death-NRFU	Assessed	NR
Rutter 2012	TConly	4984	15	Death-FUR	Assessed	30 days
Sieg 2006	TConly	109349	0	Death-FUR	Assessed	During
Stock 2013	TConly	8658	0	Death-FUR	Assessed	30 days
Stoop 2012	TConly	1276	1	Death-FUR	Assessed	30 days
Strul 2006	TConly	1177	0	Death-NRFU	Assessed	NR
Waldmann 2016	TConly	159246	0	Death-NRFU	Assessed	NR
Wang 2018a	TConly	645095	133	Death-FUR	Assessed	30 days
Wang 2018b	TConly	454271	21	Death-FUR	Assessed	7 days
Xirasagar 2020	TConly	25008	0	Death-NRFU	Assessed	NR
Zwink 2017	TConly	5252	0	Death-FUR	Assessed	3 months

sTable7c. Characteristics of subpopulations with assessment of cardiopulmonary events

Study ID	Procedure	People	Effect size	Analysis category	Assessed?	Followup
Atkin 2002	FS	2051	2	ACS long-term	Assessed	30 days
Levin 2002	FS	107704	33	ACS long-term	Assessed	28 days
Atkin 1998	FS	536	0	Follow-up time NR	Assessed	NR
Atkin 1998	FS	734	1	Follow-up time NR	Assessed	NR
Atkin 2002	FS	40674	95	Follow-up time NR	Assessed	NR
Duku 2014	FS	453	2	Follow-up time NR	Assessed	NR
Gondal 2003	FS	12960	26	Follow-up time NR	Assessed	NR
Segnan 2005	FS	4466	16	Follow-up time NR	Assessed	NR
Atkin 2002	FS	40674	1	NDCPE	Assessed	30 days
Senore 2011	FS	1198	2	NDCPE	Assessed	30 days
Segnan 2005	FS	4466	1	other	Assessed	During
Pabby 2005	FS	6968	98	Vasovagal reaction short-term	Assessed	During
Segnan 2002	FS	9911	42	Vasovagal reaction short-term	Assessed	During
Denis 2013	TCfobt	10277	1	ACS long-term	Assessed	30 days
Denis 2021	TCfobt	9061	1	ACS long-term	Assessed	30 days
Saraste 2016	TCfobt	2984	1	ACS long-term	Assessed	30 days
Tomaszewski 2021	TCfobt	78831	7	ACS short-term	Assessed	0-14 days
Denis 2013	TCfobt	10277	2	arrhythmia long-term	Assessed	30 days
Denis 2021	TCfobt	9061	2	arrhythmia long-term	Assessed	30 days
Tomaszewski 2021	TCfobt	78831	4	arrhythmia short-term	Assessed	0-14 days
Gupta 2012	TCfobt	1057	1	Follow-up time NR	Assessed	NR
Gupta 2012	TCfobt	1057	1	Follow-up time NR	Assessed	NR
Lee 2012	TCfobt	32148	1	Follow-up time NR	Assessed	NR
Hughes 2005	TCfobt	92	1	heart failure short-term	Assessed	14 days
Benazzato 2020	TCfobt	99674	49	NDCPE	Assessed	30 days
Dominitz 2019	TCfobt	14662	31	NDCPE	Assessed	30 days
Hsu 2020	TCfobt	214955	10	NDCPE	Assessed	14 days
Robertson 2019	TCfobt	17485	31	NDCPE	Assessed	30-45 days
Tepes 2017	TCfobt	13674	0	NDCPE	Assessed	NR
Tomaszewski 2021	TCfobt	78831	1	other	Assessed	0-14 days

Denis 2021	TCfobt	9061	1	Pulmonary event long-term	Assessed	30 days
Tomaszewski 2021	TCfobt	78831	1	Pulmonary event short-term	Assessed	0-14 days
Saraste 2016	TCfobt	2984	4	stroke long-term	Assessed	30 days
Tomaszewski 2021	TCfobt	78831	2	stroke short-term	Assessed	0-14 days
Denis 2013	TCfobt	10277	3	TE long-term	Assessed	30 days
Denis 2013	TCfobt	10277	1	TE long-term	Assessed	30 days
Denis 2021	TCfobt	9061	1	TE long-term	Assessed	30 days
Saraste 2016	TCfobt	2984	1	TE long-term	Assessed	30 days
Tomaszewski 2021	TCfobt	78831	2	TE short-term	Assessed	0-14 days
Ibánez 2018	TCfobt	7467	0	Vasovagal reaction long-term	Assessed	30 day
Castro 2013	TCfollowup	3215	1	arrhythmia long-term	Assessed	30 days
Gondal 2003	TCfollowup	2524	24	Follow-up time NR	Assessed	NR
Segnan 2005	TCfollowup	332	5	Follow-up time NR	Assessed	NR
Shroff 2015	TCfollowup	6152	0	NDCPE	Assessed	30 days
Castro 2013	TCfollowup	3215	2	other	Assessed	30 days
Segnan 2002	TCfollowup	775	7	Vasovagal reaction short-term	Assessed	During
Berhane 2009	TConly	11808	1	ACS long-term	Assessed	30 days
Causada-Calo 2020	TConly	30443	35	ACS long-term	Assessed	30 days
Garcia-Albeniz 2017	TConly	46872	66	ACS long-term	Assessed	30 days
Garcia-Albeniz 2017	TConly	31193	82	ACS long-term	Assessed	30 days
Nelson 2002	TConly	3196	1	ACS long-term	Assessed	30 days
Stock 2013	TConly	8658	2	ACS long-term	Assessed	30 days
Stoop 2012	TConly	1276	0	ACS long-term	Assessed	30 days
Wang 2018a	TConly	645095	160	ACS long-term	Assessed	30 day'
Lieberman 2000	TConly	3121	1	ACS short-term	Assessed	During
Pedersen 2020	TConly	11163	0	ACS short-term	Assessed	8 days
Taleban 2018	TConly	99	1	ACS short-term	Assessed	Until discharge
Causada-Calo 2020	TConly	30443	14	arrhythmia long-term	Assessed	30 days
Garcia-Albeniz 2017	TConly	46872	297	arrhythmia long-term	Assessed	30 days
Garcia-Albeniz 2017	TConly	31193	351	arrhythmia long-term	Assessed	30 days
Nelson 2002	TConly	3196	1	arrhythmia long-term	Assessed	30 days

Stoop 2012	TConly	1276	1	arrhythmia long-term	Assessed	30 days
Wang 2018a	TConly	645095	1473	arrhythmia long-term	Assessed	30 day'
Pedersen 2020	TConly	11163	21	arrhythmia short-term	Assessed	8 days
Taupin 2006	TConly	231	1	Follow-up time NR	Assessed	NR
Van Dam 2013	TConly	241	1	Follow-up time NR	Assessed	NR
Xirasagar 2020	TConly	25008	3	Follow-up time NR	Assessed	
Causada-Calo 2020	TConly	30443	38	heart failure long-term	Assessed	30 days
Garcia-Albeniz 2017	TConly	46872	124	heart failure long-term	Assessed	30 days
Garcia-Albeniz 2017	TConly	31193	160	heart failure long-term	Assessed	30 days
Wang 2018a	TConly	645095	624	heart failure long-term	Assessed	30 day'
Bokemeyer 2009	TConly	267579	222	NDCPE	Assessed	NR
Crispin 2009	TConly	55993	39	NDCPE	Assessed	NR
Ferlitsch 2011	TConly	44350	46	NDCPE	Assessed	NR
Khalid-de Bakker 2011	TConly	214	0	NDCPE	Assessed	30 days
Khalid-de Bakker 2011b	TConly	447	0	NDCPE	Assessed	NR
Kozbial 2015	TConly	59901	66	NDCPE	Assessed	NR
Pox 2012	TConly	2804983	1153	NDCPE	Assessed	NR
Pox 2012	TConly	2804983	83	NDCPE	Assessed	NR
Senore 2011	TConly	1502	3	NDCPE	Assessed	30 days
Sieg 2006	TConly	109349	105	NDCPE	Assessed	During
Waldmann 2016	TConly	159246	134	NDCPE	Assessed	NR
Causada-Calo 2020	TConly	30443	14	other	Assessed	30 days
Causada-Calo 2020	TConly	30443	6	other	Assessed	30 days
Causada-Calo 2020	TConly	30443	5	other	Assessed	30 days
Causada-Calo 2020	TConly	30443	3	other	Assessed	30 days
Garcia-Albeniz 2017	TConly	46872	30	other	Assessed	30 days
Garcia-Albeniz 2017	TConly	31193	26	other	Assessed	30 days
Lieberman 2000	TConly	3121	1	other	Assessed	During
Nelson 2002	TConly	3196	1	other	Assessed	30 days

Pedersen 2020	TConly	11163	5	other	Assessed	8 days
Wang 2018a	TConly	645095	299	other	Assessed	30 day'
Stoop 2012	TConly	1276	1	Pulmonary event long-term	Assessed	30 days
Wang 2018a	TConly	645095	1317	Pulmonary event long-term	Assessed	30 day'
Pedersen 2020	TConly	11163	15	Pulmonary event short-term	Assessed	8 days
Wang 2018b	TConly	454271	87	Pulmonary event short-term	Assessed	7 days
Wang 2018b	TConly	454271	524	Pulmonary event short-term	Assessed	7 days
Wang 2018b	TConly	454271	299	Pulmonary event short-term	Assessed	7 days
Nelson 2002	TConly	3196	4	stroke long-term	Assessed	30 days
Stock 2013	TConly	8658	3	stroke long-term	Assessed	30 days
Stoop 2012	TConly	1276	0	stroke long-term	Assessed	30 days
Wang 2018a	TConly	645095	328	stroke long-term	Assessed	30 day'
Wang 2018a	TConly	645095	429	stroke long-term	Assessed	30 day'
Lieberman 2000	TConly	3121	1	stroke short-term	Assessed	During
Pedersen 2020	TConly	11163	5	stroke short-term	Assessed	8 days
Causada-Calo 2020	TConly	30443	25	TE long-term	Assessed	30 days
Nelson 2002	TConly	3196	1	TE long-term	Assessed	30 days
Pedersen 2020	TConly	11163	5	TE short-term	Assessed	8 days
Garcia-Albeniz 2017	TConly	46872	117	Vasovagal reaction long-term	Assessed	30 days
Garcia-Albeniz 2017	TConly	31193	118	Vasovagal reaction long-term	Assessed	30 days
Nelson 2002	TConly	3196	188	Vasovagal reaction long-term	Assessed	30 days
Bretthauer 2016	TConly	11912	51	Vasovagal reaction short-term	Assessed	During
Pedersen 2020	TConly	11163	8	Vasovagal reaction short-term	Assessed	8 days

Appendix 9 – Ongoing RCTs on CRCSPs

Searching the WHO ICTRP for trials on colorectal cancer screening, we found 346 trials. Most were small trials, and trials investigating various methods to increase participation in CRCSPs. Other trials were already identified via the search strategy. We judged that 11 were relevant to the aim of this review. These are briefly outlined in the table below with link to the relevant trial register for more information on the trials. Many protocols did not describe whether harms of screening were assessed or not. However, in many cases, results on harm were reported in publications included in this review.

sTable 8. Ongoing RCTs on CRCSPs

Study name	Protocol	Registration	Methods	Size, N	Country	Status	Estimated completion	Adverse events
Colorectal Cancer Screening in Average-risk Population: a Multicenter, Randomized Control Trial Comparing Immunochemical Fecal Occult Blood Testing Versus Colonoscopy.COLON PREV	https://clinicaltrials.gov/ct2/show/record/NCT00906997?term=NCT00906997&rank=1	NCT00906997	Multicenter RCT	55498	Spain	Finished	November 2021 (Final data collection date for primary outcome measure)	No results published concenring adverse events
Colonoscopy Versus Fecal Immunochemical Test in Reducing Mortality From Colorectal Cancer (CONFIRM) (CONFIRM)	https://clinicaltrials.gov/ct2/show/NCT01239082	NCT01239082 .	Multicenter RCT	50126	USA	Active	September 29, 2028 (Final data collection date for primary outcome measure)	
Screening for Colorectal Cancer With FOBT, Virtual Colonoscopy and Optical Colonoscopy. A Randomized Clinical Trial in the Florence District (SAVE)	https://clinicaltrials.gov/ct2/show/NCT01651624	NCT01651624	RCT	16087	Italy	Finished	november 25, 2018	No results published concenring adverse events

Randomized Controlled trial to evaluate the effectiveness of total colonoscopy in colorectal cancer screening	https://upload.umin.ac.jp/cgi-open-bin/ctr_e/ctr_view.cgi?recptno=R000002416	UMIN000001980	RCT	10000	Japan	Finished	Last follow-up date 2020 Year 03 Month 01 Day	No results published concenring adverse events
Screening for Colorectal Cancer in Older Patients (PLCO Screening Trial)	https://clinicaltrials.gov/ct2/show/NCT01696981	NCT01696981	RCT	154900	USA	Finished	May 21, 2012	No results published concenring adverse events
The Moroccan Colorectal Cancer Screening, Pilot Demonstration Project in Rabat Region	https://pactr.samrc.ac.za/TrialDisplay.aspx?TrialID=5847	PACTR201902735175581	CCT	10000	Morocco	Finished	01/08/2019	No results published concenring adverse events
Trial of colorectal cancer screening in a Thai population	http://www.clinicaltrials.in.th/index.php?tp=regtrials&menu=trialsearch&smenu=fulltext&task=search&task2=view1&id=1787	TCTR20160410001	RCT	20000	Thailand	Active	28 september 2023	
Colonoscopy or Fecal Occult Blood Test in Screening Healthy Participants for Colorectal Cancer (00-046)	https://clinicaltrials.gov/ct2/show/NCT00102011	NCT00102011	RCT	4952	US	Active	-	
Care for Colon 2015 (CFC2015)	https://clinicaltrials.gov/ct2/show/NCT04049357	NCT04049357	RCT	2015	Denmark	Active	October 1, 2024	
Towards Painless Colonoscopy	https://clinicaltrials.gov/ct2/show/record/NCT04093687	NCT04093687	RCT	64	Poland	Finished	July 2021	No results published concenring adverse events
Comparative evaluation of novel screening strategies for colorectal cancer	https://www.cochranelibrary.com/central/doi/10.1002/central/CN-01937407/full	ChiCTR1800015506	RCT	20000	China	Finished	december 2021	No results published concenring adverse events

screening in China (TARGET-C)								
Screening colonoscopy findings and complications in a multi-center study in the United States	https://clinicaltrials.gov/ct2/show/NCT01239082	NCT01239082 .	RCT	50126	Puerto Rico, United States	Active	september 29, 2028	major complications of colonoscopy

Appendix 10 - Study characteristics of special case studies and studies with an unscreened control group

sTable 9. Study characteristics of special case studies and studies with an unscreened control group

Study ID	Study design	Procedure	People, N	Age interval	Compared to other studies?	Control group?	Cause for separate analysis	CPE assessed	Death assessed
Adler 2013 **[136]**	NRS	TConly	12134	50-85	NO	NO	Complex study design	YES	NO
Hol 2010b **[137]**	RCT	FS	1124	50-74	NO	YES	Complex study design	NO	NO
Hol 2010b **[137]**	RCT	Control	932	50-74	NO	YES	Complex study design	NO	NO
Ladabaum 2021 **[138]**	NRS	TConly	4482598	45-75	NO	NO	Complex study design	YES	YES
Stock 2013 **[123]**	NRS	TConly	8658	55-80	YES	YES	Control group	YES	YES
Stock 2013 **[123]**	NRS	Control	8658	55-80	NO	YES	Control group	YES	YES
Garcia-Albeniz 2017a **[105]**	NRS	TConly	46872	70-74	YES	YES	Control group	YES	NO
Garcia-Albeniz 2017b **[105]**	NRS	Control	1762816	70-74	NO	YES	Control group	YES	NO
Garcia-Albeniz 2017c **[105]**	NRS	TConly	31193	75-79	YES	YES	Control group	YES	NO
Garcia-Albeniz 2017d **[105]**	NRS	Control	1628020	75-79	NO	YES	Control group	YES	NO
Kobiela 2020 **[113]**	RCT	TConly	55390	55-64	YES	YES	Control group	NO	YES
Kobiela 2020 **[113]**	RCT	Control	338557	55-64	NO	YES	Control group	NO	YES
Wang 2018a **[130]**	NRS	TConly	645095	50-80	YES	YES	Control group	YES	YES
Wang 2018a **[130]**	NRS	Control	2015550	50-80	NO	YES	Control group	YES	YES

Deaths associated with CRCSPs using data from special case studies and studies with an unscreened control group

One of the three special case studies from the United States of America (US) assessed deaths [138]. Here, 4,482,598 people aged between 45 to 75 years were screened with once-only colonoscopy in the states of California from 2005–2011 and Florida and New York from 2009–2015. In this study, the rate of adverse events 180 days before and after the colonoscopy was recorded using in-hospital registries with unclear validity, leading to a serious risk of measurement bias. The 360-day follow-up time was used to establish a background rate of adverse events, which was then used to calculate whether there was an excess of adverse events. No excess in-hospital deaths were observed. Out-of-hospital deaths were not ascertained. Three of the five studies with an unscreened control group assessed deaths [113, 123, 130]. In all three studies, the screened group received once-only colonoscopy with 30 days of follow-up and outcome assessment via registries with unclear validity leading to a serious risk of missing data bias. One NRS from Germany screened 8,658 people and found zero deaths. However, the measurement method was so poor that we rated the risk of measurement bias as critical [123]. Another study from Poland assessed the number of deaths occurring during three time periods, a) 42 days before colonoscopy to 30 days after, b) 42 days before colonoscopy to the day before the colonoscopy, and c) the day of the colonoscopy to 30 days after the colonoscopy. The deaths were compared between people invited to screening and those not invited to screening. They found no significant differences in mortality rates between the control and screening groups for any of the three observation periods. Of the people invited to screening, 55390 attended screening (16.4%). These people were compared to a matched control group (per-protocol analysis). However, the number of deaths in the matched control group was ten times higher for the observation period, likely indicating issues with matching. The third study was from the US and compared the 30-day in-hospital and emergency department mortality of people undergoing screening colonoscopy without polypectomy to three comparator groups, including people undergoing various common and minimally invasive procedures not expected to have major systemic complications [131]. Analyses were adjusted demographics and comorbidities. Here, 645,095 people were screened, and 133 deaths occurred, corresponding to 2.1 deaths per 10,000 people screened, 95% CI [1.7-2.4]. For the three comparator groups, the number of deaths per 10.000 people was 1.1, 4.2 and to 6.3. In adjusted analyses, the odds for deaths occurring during screening were a little lower than in the three comparator groups, OR: 0.66 to 0.85.

Cardiopulmonary events associated with CRCSPs using data from special case studies and studies with an unscreened control group

Two of the special case studies assessed CPEs (67%) [136, 138]. The study mentioned above from the US, with 4,482,598 people screened with once-only colonoscopy, found an excess rate of many CPEs after screening colonoscopy, including acute myocardial infarction, heart failure, arrhythmia, ischemic stroke, transient ischemic attack and pneumonia with an odds ratio between observed event rate to an expected event rate of about 1 to 1.9 [138]. The other study was an NRS from Germany with an assessment of harm using case report forms given to the person performing the endoscopic procedure and to patients, which was compared to data in registries. Here, CPEs were reported as a composite outcome without event rates for each distinct type of CPE. In total, 26 events were reported, seven of which led to hospitalisation [136]. Three of the five studies with an unscreened control group assessed CPEs [105, 123, 130]. In all three studies, the screened group received a once-only colonoscopy with 30 days of follow-up. The worst bias score was serious due to both classifications, missing data and measurement bias. The study above from Germany provided insignificant findings due to small event rates [123]. The US study found lower odds of CPEs after screening colonoscopy compared to the three comparator groups in adjusted analyses [130]. In contrast, the third study, also from the US, found that people aged 70-74 and 75-79 had a significantly higher risk than the unscreened group of suffering arrhythmias, heart failure and vasovagal events [105]. Also, people aged 75-79 had 1.4 times higher risk of heart failure and 1.31 times higher risk of acute coronary syndrome than the unscreened group [105].

Appendix 11 – Studies from countries

sTable 10. Distribution of non-randomised studies (NRS) across countries

	For all outcomes		Studies assessing "CPE"		Studies assessing "Death"		Studies assessing "CPE" or "Death"	
Country	Subpop	%	Subpop	%	Subpop	%	Subpop	%
Netherlands	7	6%	2	5%	1	2%	3	5%
UK	18	17%	2	5%	6	14%	6	11%
Romania	1	1%	0	0%	0	0%	0	0%
Germany	9	8%	5	13%	5	12%	6	11%
South Korea	1	1%	0	0%	0	0%	0	0%
Finland	1	1%	0	0%	0	0%	0	0%
China	1	1%	0	0%	0	0%	0	0%
US	29	27%	14	36%	14	33%	19	33%
Japan	1	1%	0	0%	0	0%	0	0%
Australia	5	5%	2	5%	0	0%	2	4%
France	6	6%	2	5%	2	5%	2	4%
Sweden	1	1%	1	3%	1	2%	1	2%
Spain	7	6%	2	5%	4	9%	5	9%
Italy	4	4%	1	3%	2	5%	2	4%
Denmark	2	2%	1	3%	2	5%	2	4%
Taiwan	3	3%	1	3%	1	2%	2	4%
Israel	1	1%	0	0%	1	2%	1	2%
Slovenia	1	1%	1	3%	0	0%	1	2%
Austria	3	3%	3	8%	2	5%	3	5%
Canada	3	3%	2	5%	2	5%	2	4%
Portugal	1	1%	0	0%	0	0%	0	0%
Poland	3	3%	0	0%	0	0%	0	0%
Total	108	100%	39	100%	43	100%	57	100%

sFigure 1: Distribution of NRS that assess either CPE or death across countries

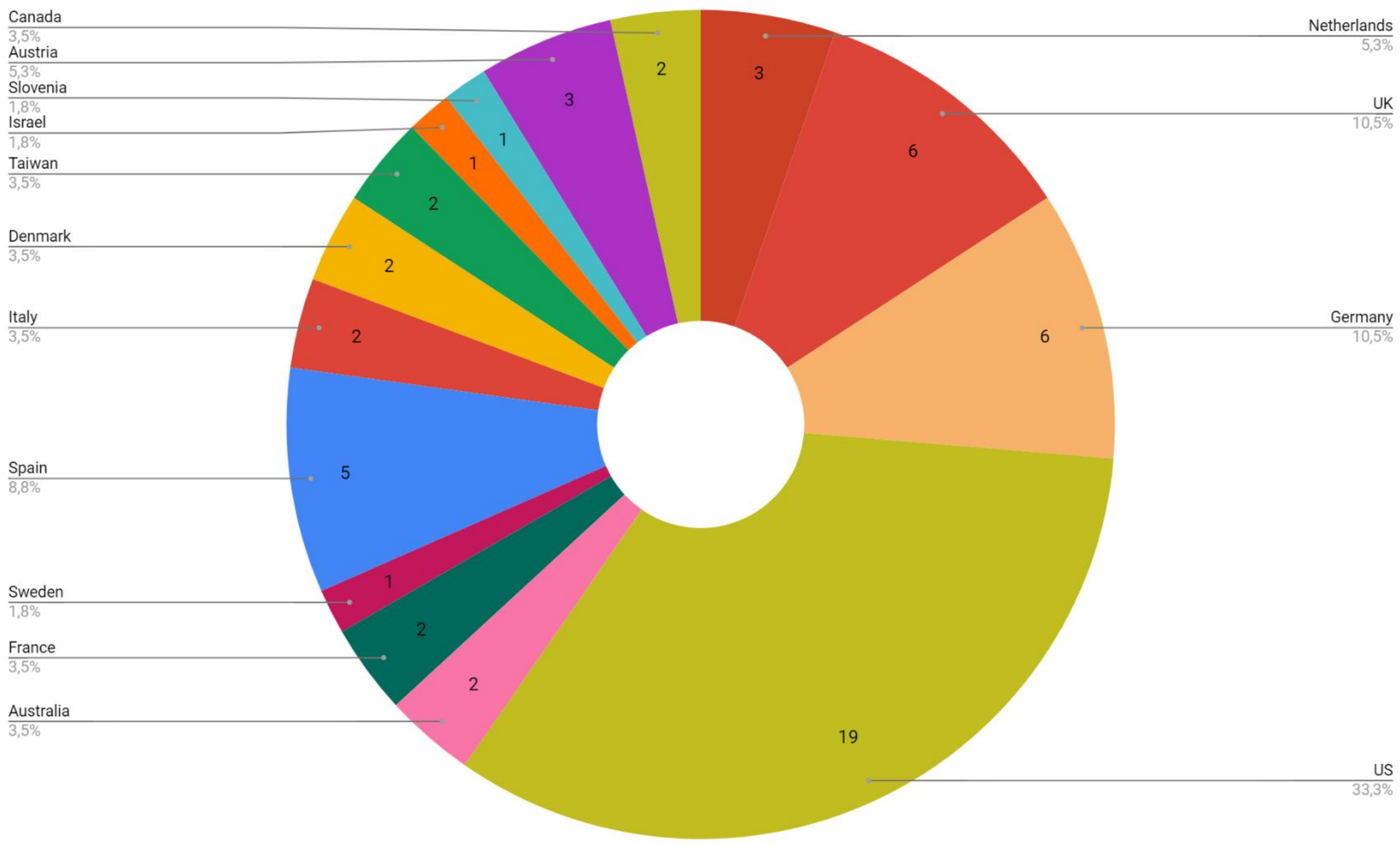

sTable 11. Distribution of randomized controlled trials (RCTs across countries

	For all outcomes		Studies assessing "CPE"		Studies assessing "Death"		Studies assessing "CPE" or "Death"	
Country	Subpop	%	Subpop	%	Subpop	%	Subpop	%
Multinational (1)	1	2%	1	6%	1	8%	1	4%
UK	5	12%	3	19%	5	42%	5	22%
Sweden	5	12%	0	0%	0	0%	0	0%
Norway	9	21%	2	13%	4	33%	6	26%
US	4	9%	1	6%	0	0%	1	4%
Netherlands	5	12%	2	13%	1	8%	2	9%
Spain	2	5%	0	0%	0	0%	0	0%
Italy	6	14%	6	38%	0	0%	6	26%
Australia	2	5%	0	0%	0	0%	0	0%
Denmark	2	5%	0	0%	0	0%	0	0%
Poland	1	2%	0	0%	1	8%	1	4%
NR	1	2%	1	6%	0	0%	1	4%
Total	43	100%	16	100%	12	100%	23	100%
(1) Multinational, multicentre RCT conducted in Poland, Norway, the Netherlands, and Sweden								

sFigure 2: Distribution of RCTs that assess either CPE or death across countries

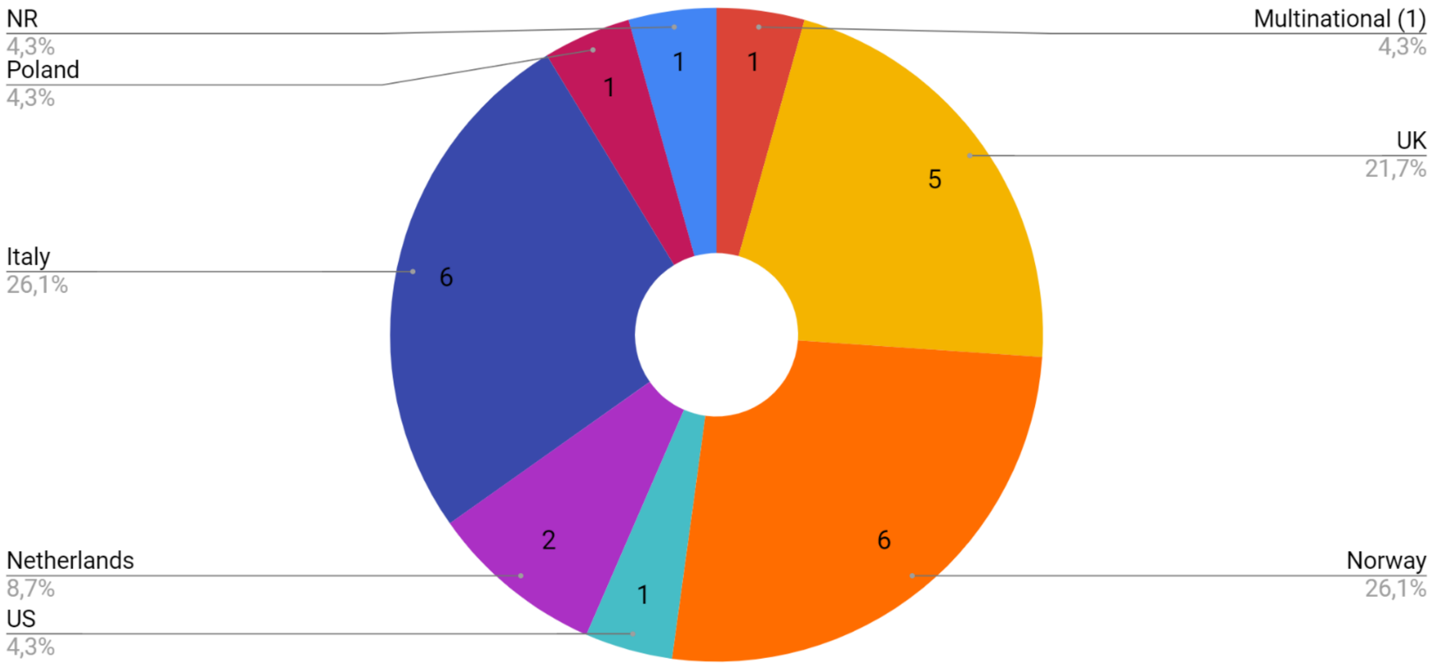

Appendix 12 – Characteristics of studies with assessment of CPEs or death

sTable 12. Characteristics of RCTs with assessment of CPEs or death

	Result	Publications	Percentage
No. publications		23	100%
Age range across publications	45-75	23	100%
Mean age range across publications	56,9-59,3	3	13%
Median age range across publications	60,2-63,3	2	9%
Not reporting mean or median age		18	78%
Range of sex distribution across publications, %women	0,374–0,535	16	70%
Sociodemographic information		5	22%
Explicit definition of physical harms		9	39%

sTable 13. Characteristics of NRS with assessment of CPEs or death

	Result	Publications	Percentage
No. publications		57	100%
Age range across publications	20-100	53	93%
Mean age range across publications	46,8-66,1	30	53%
Median age range across publications	60,7-67	5	9%
Not reporting mean or median age		22	39%
Range of sex distribution across publications, %women	0,032–0,74	50	88%
Sociodemographic information		12	21%
Explicit definition of physical harms		34	60%

Appendix 13 – Study characteristics related to screening procedure group

In the following analyses, we judged that endoscopists were experienced using information available in publications, including the number of endoscopies performed per endoscopists and or being certified/accredited.

sTable 14. Characteristics of subpopulations screened with FS with assessment of deaths

Procedure	FS								
Outcome	Death								
	All			RCTs			NRS		
Subpopulations in total	151	100%		43	28%		108	72%	
Subpopulations, procedure	29	19%		17	40%		12	11%	
Subpopulations, procedure & Outcome	7	24%		5	12%		2	2%	
People	202.933			91.000			111.933		
Procedures	240.108			107.670			132.438		
Procedures per person	1,1831900138								
		N	Report %		N	Report %		N	Report %
Women%, weighted mean	49,41%	4	57%	50,13%	3	60%	48,80%	1	50%
Women%, range	0,488-0,508	4	57%	0,4955-0,508	3	60%	0,488-0,488	1	50%
Age Range	50-79	7	100%	50-74	5	100%	50-79	2	100%
Mean age, weighted mean	61	2	29%	57	1	20%	61	1	50%
Polypectomy provided in studies, N	7	7	100%	5	5	100%	2	2	100%
Polypectomy rate known in studies, N	3	3	43%	3	3	60%	0	0	0%
Polypectomy rate, weighted mean	23,59%	3	43%	23,59%	3	60%	NA	0	0%

sTable 15. Characteristics of subpopulations screened with FS with assessment of CPE

Procedure	FS								
Outcome	CPE								
	All			RCTs			NRS		
Subpopulations in total	151	100%		43	28%		108	72%	
Subpopulations, procedure	29	19%		17	40%		12	11%	
Subpopulations, procedure & Outcome	10	34%		7	16%		3	3%	
People	186.195			71.070			115.125		
Procedures	220.304			84.089			136.215		
Procedures per person	1,1831900138								
		N	Report %		N	Report %		N	Report %
Women%, weighted mean	49,22%	7	70%	49,52%	5	71%	49,04%	2	67%
Women%, range	0,468-0,528	7	70%	0,468-0,514	5	71%	0,488-0,528	2	67%
Age Range	50-79	10	100%	50-64	7	100%	50-79	3	100%
Mean age, weighted mean	61	2	20%	NA	0	0%	61	2	67%
Polypectomy provided in studies, N	10	10	100%	7	7	100%	3	3	100%
Polypectomy rate known in studies, N	3	3	30%	3	3	43%	0	0	0%
Polypectomy rate, weighted mean	23,59%	3	30%	23,59%	3	43%	NA	0	0%

sTable 16. Characteristics of subpopulations screened with TCfobt with assessment of deaths

Procedure	TCfobt								
Outcome	Death								
	All			RCTs			NRS		
Subpopulations in total	151	100%		43	28%		108	72%	
Subpopulations, procedure	54	36%		6	14%		48	44%	
Subpopulations, procedure & Outcome	20	37%		1	2%		19	18%	
People	830.233			1.246			828.987		
Procedures	736.659			1.106			735.554		
Procedures per person	0,8872919056								
		N	Report %		N	Report %		N	Report %
Women%, weighted mean	42,43%	16	80%	NA	0	0%	42,43%	16	84%
Women%, range	0,314-0,715	16	80%	0-0	0	0%	0,314-0,715	16	84%
Age Range	20-92	20	100%	0-0	1	100%	20-92	19	100%
Mean age, weighted mean	65	7	35%	NA	0	0%	65	7	37%
Polypectomy provided in studies, N	14	14	70%	1	1	100%	13	13	68%
Polypectomy rate known in studies, N	7	7	35%	0	0	0%	7	7	37%
Polypectomy rate, weighted mean	56,57%	7	35%	NA	0	0%	56,57%	7	37%

sTable 17. Characteristics of subpopulations screened with TCfobt with assessment of CPE

Procedure	TCfobt								
Outcome	CPE								
	All			RCTs			NRS		
Subpopulations in total	151	100%		43	28%		108	72%	
Subpopulations, procedure	54	36%		6	14%		48	44%	
Subpopulations, procedure & Outcome	14	26%		2	5%		12	11%	
People	501.784			32.147			469.637		
Procedures	445.229			28.524			416.705		
Procedures per person	0,8872919056								
		N	Report %		N	Report %		N	Report %
Women%, weighted mean	43,94%	9	64%	NA	0	0%	43,94%	9	75%
Women%, range	0,384-0,463	9	64%	0-0	0	0%	0,384-0,463	9	75%
Age Range	50-92	14	100%	0-0	2	100%	50-92	12	100%
Mean age, weighted mean	61	4	29%	NA	0	0%	61	4	33%
Polypectomy provided in studies, N	11	11	79%	1	1	50%	10	10	83%
Polypectomy rate known in studies, N	7	7	50%	1	1	50%	6	6	50%
Polypectomy rate, weighted mean	44,75%	7	50%	61,80%	1	50%	44,03%	6	50%

sTable 18. Characteristics of subpopulations screened with TConly with assessment of deaths

Procedure	TConly								
Outcome	Death								
	All			RCTs			NRS		
Subpopulations in total	151	100%		43	28%		108	72%	
Subpopulations, procedure	48	32%		8	19%		40	37%	
Subpopulations, procedure & Outcome	21	44%		3	7%		18	17%	
People	4.658.351			68.578			4.589.773		
Procedures	5.242.810			77.182			5.165.627		
Procedures per person	1,1254647346								
		N	Report %		N	Report %		N	Report %
Women%, weighted mean	54,69%	20	95%	52,53%	3	100%	54,72%	17	94%
Women%, range	0,032-0,574	20	95%	0,484-0,535	3	100%	0,032-0,574	17	94%
Age Range	30-100	18	86%	50-74	3	100%	30-100	15	83%
Mean age, weighted mean	64	13	62%	59	1	33%	64	12	67%
Polypectomy provided in studies, N	15	15	71%	2	2	67%	13	13	72%
Polypectomy rate known in studies, N	6	6	29%	0	0	0%	6	6	33%
Polypectomy rate, weighted mean	36,28%	6	29%	NA	0	0%	36,28%	6	33%

sTable 19. Characteristics of subpopulations screened with TConly with assessment of CPE

Procedure	TConly								
Outcome	CPE								
	All			RCTs			NRS		
Subpopulations in total	151	100%		43	28%		108	72%	
Subpopulations, procedure	48	32%		8	19%		40	37%	
Subpopulations, procedure & Outcome	26	54%		4	9%		22	20%	
People	4.752.720			14.892			4.737.828		
Procedures	5.349.019			16.760			5.332.258		
Procedures per person	1,1254647346								
		N	Report %		N	Report %		N	Report %
Women%, weighted mean	54,64%	25	96%	48,53%	4	100%	54,66%	21	95%
Women%, range	0,032-0,607	25	96%	0,48-0,493	4	100%	0,032-0,607	21	95%
Age Range	30-100	24	92%	50-74	4	100%	30-100	20	91%
Mean age, weighted mean	64	15	58%	NA	0	0%	64	15	68%
Polypectomy provided in studies, N	19	19	73%	2	2	50%	17	17	77%
Polypectomy rate known in studies, N	9	9	35%	0	0	0%	9	9	41%
Polypectomy rate, weighted mean	34,29%	9	35%	NA	0	0%	34,29%	9	41%

sTable 20. Characteristics of subpopulations screened with TCfollowup with assessment of deaths

**Procedure**	**TCfollowup**								
**Outcome**	**Death**								
	**All**			**RCTs**			**NRS**		
Subpopulations in total	151	100%		43	28%		108	72%	
Subpopulations, procedure	20	13%		12	28%		8	7%	
Subpopulations, procedure & Outcome	7	35%		3	7%		4	4%	
People	26.139			8.174			17.965		
Procedures	32.666			10.215			22.451		
Procedures per person	1,2496909137								
		N	Report %		N	Report %		N	Report %
Women%, weighted mean	58,30%	6	86%	49,86%	2	67%	60,59%	4	100%
Women%, range	0,4955-0,74	6	86%	0,4955-0,501	2	67%	0,51-0,74	4	100%
Age Range	21-91	6	86%	50-64	3	100%	21-91	3	75%
Mean age, weighted mean	58	4	57%	57	1	33%	58	3	75%
Polypectomy provided in studies, N	4	4	57%	3	3	100%	1	1	25%
Polypectomy rate known in studies, N	1	1	14%	0	0	0%	1	1	25%
Polypectomy rate, weighted mean	44,10%	1	14%	NA	0	0%	44,10%	1	25%

sTable 21. Characteristics of subpopulations screened with TCfollowup with assessment of CPE

Procedure	TCfollowup								
Outcome	CPE								
	All			RCTs			NRS		
Subpopulations in total	151	100%		43	28%		108	72%	
Subpopulations, procedure	20	13%		12	28%		8	7%	
Subpopulations, procedure & Outcome	5	25%		3	7%		2	2%	
People	13.007			3.640			9.367		
Procedures	16.255			4.549			11.706		
Procedures per person	1,2496909137								
		N	Report %		N	Report %		N	Report %
Women%, weighted mean	65,25%	3	60%	46,80%	1	33%	66,78%	2	100%
Women%, range	0,468-0,74	3	60%	0,468-0,468	1	33%	0,63-0,74	2	100%
Age Range	50-64	4	80%	55-64	3	100%	50-64	1	50%
Mean age, weighted mean	57	2	40%	NA	0	0%	57	2	100%
Polypectomy provided in studies, N	3	3	60%	3	3	100%	0	0	0%
Polypectomy rate known in studies, N	0	0	0%	0	0	0%	0	0	0%
Polypectomy rate, weighted mean	NA	0	0%	NA	0	0%	NA	0	0%

Appendix 14 – Conceptualization of physical harms across studies

sTable 22. Conceptualization of physical harms across studies

Study ID	Design	Harm definition
Arana-Arri 2018	NRS	It was considered a case when a complication was identified as defined in the European guide on CRC screening: hospitalization within 30 days for serious hemorrhage involving transfusion, or for perforation, vagal syndrome or peritonitis-like syndrome and death attributed to complications of a screening colonoscopy.
Benazzato 2020	NRS	Major adverse events and mortality. Defined as requiring hospital admission within 30 days of a screening colonoscopy.
Bielawska 2014	NRS	the term “early perforation” refers to a perforation discovered before the procedure report is signed off at the end of the colonoscopy. Early perforations are known to make up approximately one quarter of all perforation events (1).
Binefa 2013	NRS	Colonoscopy complications = N<br>Total number of severe complications such as perforation and post-polypectomy bleeding (involving transfusion or hospitalisation of at least 24 hours) and death (within 30 days)
Blanks 2015	NRS	An adverse event is defined by the NHSBCSP as an event that prevents the completion of the procedure (excluding technical failure or poor bowel preparation) and/or results in: (i) admission to hospital or prolonged hospital stay; (ii) another intervention (endoscopic, radiological, or surgical); or (iii) subsequent medical consultation.
Castro 2013	NRS	major complications were defined as any conditions or symptoms that resulted in hospital admission within 30 days of the procedure and included perforation, gastrointestinal bleeding requiring or not requiring blood transfusion, cardiopulmonary events, postpolypectomy syndrome, excessive abdominal pain, and death. All complications occurring within 30 days of any program test were included, regardless of whether the medical records associated the complication with the procedure
Causada-Calo 2020	NRS	defined as the composite of unplanned hospital admissions or emergency department visits at 30 days after outpatient colonoscopy.
Crispin 2009	NRS	Acute complications
Dellon 2009	NRS	Complications were defined as the following: respiratory depression unresponsive to supplemental oxygen, with or without the use of reversal agents; hypotension that required pharmacologic support or a fluid bolus; cardiac arrhythmia (bradycardia with heart rate !60 beats per minute, heart block, nonsinus tachyarrhythmia with heart rate O100 beats/min), with or without the use of reversal agents; colonic perforation; and death
Denis 2013	NRS	An event that prevented completion of the planned procedure and/or resulted in admission to hospital, prolongation of existing hospital stay, another procedure needing sedation/anaesthesia, or subsequent medical consultation was considered an AE [42]. Unplanned events that did not interfere with completion of the planned procedure or change the plan of care were considered incidents
Denis 2021	NRS	AEs were classified according to the American Society for Gastrointestinal Endoscopy (ASGE) lexicon. All events definitely, probably, and possibly related to colonoscopy occurring within 30 days of the colonoscopy were taken into account. whereas events unlikely related were not. An event that prevented completion of the planned procedure and/or resulted in admission to the hospital, prolongation of an existing hospital stay, another procedure needing sedation/anesthesia, or subsequent medical consultation was considered an AE. Unplanned events that did not interfere with completion of the planned procedure or change the plan of care were considered incidents
Din 2015	NRS	Major post-polypectomy adverse events were defined as: bleeding episodes requiring transfusion or colonic perforation within 30 days of the procedure
Dominitz 2019	RCT	30 day complication rate

Dyson 2014	NRS	An adverse event is defined by the NHSBCSP as an event that prevents the completion of the procedure (excluding technical failure or poor bowel preparation) and/or results in: (i) admission to hospital or prolonged hospital stay; (ii) another intervention (endoscopic, radiological, or surgical); or (iii) subsequent medical consultation.
Ellul 2010	NRS	An adverse event is defined by the NHSBCSP as an event that prevents the completion of the procedure (excluding technical failure or poor bowel preparation) and/or results in: (i) admission to hospital or prolonged hospital stay; (ii) another intervention (endoscopic, radiological, or surgical); or (iii) subsequent medical consultation.
Garcia 2012	NRS	severe complications during the diagnostic procedure (perforation, bleeding requiring transfusion, vagal syndrome or peritonitis-like syndrome).
Garcia-Albeniz 2017	NRS	We also identified all adverse events occurring within 30 days after baseline that were severe enough to require an emergency department visit or hospitalization. We classified adverse events as serious gastrointestinal (perforation, gastrointestinal bleeding requiring transfusion), other gastrointestinal (gastrointestinal bleeding not requiring transfusion, paralytic ileus, nausea, vomiting and dehydration, abdominal pain), or cardiovascular (myocardial infarction or angina, arrhythmias, congestive heart failure, cardiac or respiratory arrest, syncope, hypotension or shock) events
Ghanouni 2016	NRS	An adverse event is defined by the NHSBCSP as an event that prevents the completion of the procedure (excluding technical failure or poor bowel preparation) and/or results in: (i) admission to hospital or prolonged hospital stay; (ii) another intervention (endoscopic, radiological, or surgical); or (iii) subsequent medical consultation.
Hamdani 2013	NRS	The study outcome was the diagnosis of colonic perforationusing International Classification of Disease, 9th revision (ICD-9) codes 569.83 and 998.2, defined as perforation of intestine and accidental puncture or laceration during a procedure, 7 d after the day of colonoscopy
Holme 2014	RCT	defined as complications
Hsu 2020	NRS	severe colonoscopy-related complications - Severe complications were defined as significant bleeding, perforation, and cardiopulmonary events. Complications included any all-cause, unplanned hospital visit (emergency department visit and patient admission) within 14 days after the index colonoscopy.
Ibánez 2018	NRS	Colonoscopy complications can be classified as mild or severe. Mild complications include symptoms such as pain or bloating, diarrhoea, constipation, nausea, vomiting, blood in the stool, anal or rectal pain and headaches. Severe complication was defined according to the def- inition proposed in the European Guide for the quality of screening and diagnosis of colorectal cancer, ‘‘complication which requires hospital admission or causes death due to perforation or haemorrhage which requires transfusion or severe vasovagal syndrome or peritonitis, and occurs within a period of 0 to 30 days from completion of the colonoscopy’’.5<br>A severe complication was considered as an immedi- ate complication when it occurred on the same day as the colonoscopy and as a late complication when it occurred any time from the following day to 30 days after the colonoscopy.
Karlijn 2021	NRS	All adverse events occurring within 30 days after endoscopy are manually recorded by endoscopists in the DRCE. For colonoscopies, the following types of adverse events are registered: cardiovascular, pulmonary, thromboembolic, perforation, bleeding, infectious, allergy/intolerance, pain, and an option for recording adverse events other than aforementioned. The severity grading system for adverse events as proposed by the ASGE is used [139]. The likelihood of the adverse event being related to the endoscopy is recorded as “related,” “likely related,” “possibly related,” or “unlikely related” to the procedure. Thus, the all-cause adverse event rate can be monitored.

Ko 2010	NRS	We examined the incidence of serious events directly related to colonoscopy, as ascertained by subjects' self-report, including perforation, postpolypectomy syndrome, gastrointestinal bleeding requiring hospitalization and/or transfusion, and diverticulitis. The primary outcome was an aggregate measure of these 4 most serious complications requiring hospitalization within 30 days.
Kobiela 2020	RCT	AE reporting requirements were based on the review of International Classifica- tion of Diseases (ICD)-9 and ICD-10 coding, length of stay, and other available data.
Kooyker 2021	NRS	We estimated the colonoscopy-related mortality rate in a FIT-based CRC screening program in 3 independent ways using prospectively collected data from several sources:<br>1. The fatal complication rate among FIT-positives undergoing colonoscopy based on endoscopistreported complications from national endoscopy complication registries.<br>2. The 30-days excess death rate in FIT-positives undergoing colonoscopy compared with a reference population not undergoing colonoscopy (FITnegatives).<br>3. The rate of deaths among FIT-positives undergoing colonoscopy that were likely related to colonoscopy based on data on causes of death.
Ladabaum 2021	NRS	We defined adverse events as emergency department visits or hospital admissions within 180 days preceding or after the colonoscopy
Lee 2012	NRS	An adverse event is defined by the NHSBCSP as an event that prevents the completion of the procedure (excluding technical failure or poor bowel preparation) and/or results in: (i) admission to hospital or prolonged hospital stay; (ii) another intervention (endoscopic, radiological, or surgical); or (iii) subsequent medical consultation.
Levin 2002	NRS	Chart reviews included all cases in which the discharge diagnoses included 1 of the following: bowel perforation, complication of procedure, abdominal pain, lower gastrointestinal bleeding, fever, colitis of unclear etiology, diverticulitis, or anemia. Review also included all cases in which the electronic database indicated 1 or more of the following procedures: suture of laceration of large intestines, partial excision of large intestines, total intra-abdominal colectomy, laparotomy, colostomy, ileostomy, and abdominal resection of rectum
Lieberman 2000	NRS	serious complications during or immediately after colonoscopy
Mikkelsen 2018	NRS	any complication (n=34) including the following four categories: 1) perforation or lesion during colonoscopy (DT812G1), 2) post-colonoscopy bleeding that required treatment or prompted the patient to contact a hospital for medical evaluation (DT810J1), 3) other medical complications related to colonoscopy or sedation (eg, hypotension, pain, vomiting, and respiratory complications) that prevented completion of the colonoscopy or required medical treatment (DT888U1), and 4) post-polypectomy syndrome defined as fever and abdominal pain without symptoms of perforation or pneumoperitoneum (DT888L
Nelson 2002	NRS	Complications were considered major if they required a blood transfusion, hospitalization, surgery, or if they resulted in death. Data on all complications within 30 days were collected
Paszat 2020	NRS	AEs are defined as the rate of hospital ad- mission for colonoscopic perforation ≤ 7 days following the date of outpatient colonoscopy, and the frequency of hospital ad- mission for bleeding due to colonoscopic polypectomy ≤14 days following the colonoscopy, among CCC participants.
Pedersen 2020	NRS	AEs from readmission within eight days or death within 30 days of colonoscopy. AEs were investigated in electronic health records and categorised, attributed and graded according to the ASGE lexicon. Each AE was attributed to colonoscopy as unlikely, possible, probable or definite; severity was graded as mild, moderate, severe or fatal
Portillo 2018	NRS	hospital discharges and emergency care to detect any severe or minor complica- tions within 30 days. condi- tions were considered severe

		complications if they required the patient to remain in hospital after the procedure for at least 24 hours or, in patients discharged after of colonoscopy, requir- ed readmission within 30 days after the procedure.
Pox 2012	NRS	The following complications were defined as major complications: death related to colonoscopy, perforation, significant bleeding (requiring blood transfusion, hospitalization, or surgery), cardiopulmonary events requiring hospitalization, and any other complication requiring hospital admission. Less significant complications were defined as follows: bleeding controlled at endoscopy (no transfusion or hospitalization), cardiopulmonary events controlled at time of endoscopy not requiring hospitalization, and other complications not requiring hospitalization.
Quyn 2018	NRS	Adverse events were defined as those that prevented completion of the planned procedure (excluding technical failure or poor bowel preparation) or resulted in admission to hospital, prolongation of existing hospital stay, another interventional procedure or subsequent medical consultation
Randel 2021	RCT	Adverse events occuring during or within 30 days after the procedure or pain during the procedure
Regula 2006	NRS	Clinically significant complications requiring medical intervention (including complications from polypectomy)
Robertson 2019	RCT	adverse events are defined as non-serious and reported when deemed to be at least possibly resulting from the colonoscopy and serious adverse events are always reported.
Rutter 2014	NRS	An adverse event is defined by the NHSBCSP as an event that prevents the completion of the procedure (excluding technical failure or poor bowel preparation) and/or results in: (i) admission to hospital or prolonged hospital stay; (ii) another intervention (endoscopic, radiological, or surgical); or (iii) subsequent medical consultation.
Saraste 2016	NRS	All adverse events deviating from a normal postoperative course were registered after scrutinizing the hospital charts. In patients with multiple complications, the most severe of these was registered according to the Clavien–Dindo classification of surgical complications.
Segnan 2002	RCT	Immediate complications
Shroff 2015	NRS	Major complications were defi ned as any event related to the procedure and requiring admission within 30 days of the procedure. This includes perforations, post polypectomy bleeding requiring transfusion and/or hospital admission, cardiopulmonary events, and neurologic events
Sieg 2006	NRS	Acute complications were also recorded on the documentation sheet: cardiopulmonary complications, polypectomy bleeding and perforations. During the bleeding it could be indicated if an operation was necessary. In all patients with perforations or postoperative bleeding, follow-up examinations were the outcome of the complication.
Stock 2013	NRS	Serious AEs of interest were perforation, bleeding, myocardial infarction, stroke, splenic injury, and a number of adverse outcomes grouped as other AEs.
Taleban 2018	NRS	Acute adverse events during and immediately following the colonoscopy. Adverse events were stratified into three categories according to their level of severity. Major adverse events required hospitalization or blood transfusion. Minor adverse events, including changes in cardiovascular and pulmonary status, were further distinguished according to require- ment of an intervention (i.e., medication, increased oxy- genation). Cardiopulmonary changes included hypotension (systolic ≤ 90 mmHg), hypertension (systolic ≥ 180 mmHg), bradycardia (heart rate < 40), tachycardia (heart rate > 100), and desaturation (SpO2 < 90) episodes. Also, the use of medication reversal agents (e.g., atropine and metoprolol) and increased oxygen were noted. Otherwise, patients were categorized as having transient minor adverse events that did not require intervention.
Tomaszewski 2021	NRS	We defined unplanned events as serious adverse events if they resulted in death, hospital admission or important inter- vention, and we subclassified them (by consensus) as probably, possibly or unlikely related to the colonoscopy.

Vanaclocha-Espi 2018	NRS	we defined SC as any complication requiring hospital admission or causing death due to perforation, bleeding requiring transfusion, vagal syndrome or peritonitis, and occurring between 0 and 30 days after colonoscopy" under 2.2. Study design: cases and controls.
Wang 2018a	NRS	complications occurring within 30 days, defined as presenting to an emergency department (SEDD) or inpatient setting (SID).<br><br>For the primary analyses, we defined complications as emergency department visits or hospital admissions within 30 days following the initial procedure, based on the standard 30-day period in the post-surgical literature.
Wang 2018b	NRS	All-cause unplanned visits within 30 days after procedures
Xirasagar 2020	NRS	minimal adverse events
Zwink 2017	NRS	complications arising during or within four weeks of colonoscopies performed as part of the German screening colonoscopy program.

Appendix 15 – Risk of bias in studies with assessments of death

sTable 23. Risk of bias in studies with assessments of death

Direction of effect: Unpredictable (?), Underestimate (U), Overestimate (O). **Risk of bias: Critical, serious, moderate, low**

Procedure	Design	Study ID	Worst score	Outcome	Analysis category	Inception	Classification	Performance	Missing data	Measurement	Reporting
FS	RCT	Atkin 1998	Critical	Death	Death-NRFU	?	?	?	U	?	?
FS	RCT	Atkin 1998	Critical	Death	Death-NRFU	?	?	?	U	?	?
FS	RCT	Holme 2014	Critical	Death	Death-NRFU	?	?	?	U	U	U
FS	NRS	Jain 2002	Critical	Death	Death-NRFU	?	?	?	U	U	?
FS	NRS	Levin 2002	Critical	Death	Death-FUR	?	U	?	U	U	?
FS	RCT	Atkin 2002	Serious	Death	Death-FUR	?	?	?	U	U	?
FS	RCT	Randel 2021	Serious	Death	Death-FUR	?	U	?	?	U	?
TCfobt	NRS	Arana-Arri 2018	Critical	Death	Death-FUR	?	?	?	U	U	?
TCfobt	NRS	Cheng 2002	Critical	Death	Death-NRFU	?	?	?	?	U	?
TCfobt	NRS	Din 2017	Critical	Death	Death-NRFU	?	?	?	U	U	?
TCfobt	RCT	Robinson 1999	Critical	Death	Death-FUR	U	?	?	?	U	?
TCfobt	NRS	Steele 2009	Critical	Death	Death-NRFU	?	?	?	?	U	?
TCfobt	NRS	Quyn 2018	Critical	Death	Death-NRFU	?	?	?	?	U	?
TCfobt	NRS	Denis 2013	Moderate	Death	Death-FUR	?	?	?	U	U	?
TCfobt	NRS	Benazzato 2020	Moderate	Death	Death-FUR	?	?	?	?	?	U
TCfobt	NRS	Denis 2021	Serious	Death	Death-FUR	?	U	?	U	U	?
TCfobt	NRS	Ibánez 2018	Serious	Death	Death-FUR	?	?	?	U	U	?
TCfobt	NRS	Lee 2012	Serious	Death	Death-NRFU	?	?	U	U	U	?
TCfobt	NRS	Marino 2012	Serious	Death	Death-FUR	?	?	?	U	U	?
TCfobt	RCT	Randel 2021	Serious	Death	Death-FUR	?	U	?	?	U	?
TCfobt	NRS	Derbyshire 2018	Serious	Death	Death-FUR	?	?	?	U	U	U
TCfobt	NRS	Portillo 2018	Serious	Death	Death-FUR	?	?	?	?	U	?
TCfobt	NRS	Vanaclocha-Espi 2018	Serious	Death	Death-FUR	?	?	?	U	U	?
TCfobt	NRS	Tomaszewski 2021	Serious	Death	Death-FUR	?	U	?	U	U	?
TCfobt	NRS	Denis 2021	Serious	Death	Death-FUR	?	U	?	U	U	?
TCfobt	NRS	Kooyker 2021	Serious	Death	Death-FUR	?	?	?	U	?	?
TCfobt	NRS	Saraste 2016	Serious	Death	Death-FUR	?	U	?	U	U	?
TCfobt	NRS	Mikkelsen 2018	Serious	Death	Death-FUR	?	?	?	U	U	?
TCfobt	NRS	Gupta 2012	Serious	Death	Death-NRFU	U	?	?	U	U	?

Procedure	Design	Study ID	Worst score	Outcome	Analysis category	Inception	Classification	Performance	Missing data	Measurement	Reporting
TCfollowup	NRS	Castro 2013	Critical	Death	Death-FUR	?	?	?	U	U	?
TCfollowup	RCT	Holme 2014	Critical	Death	Death-NRFU	?	?	?	U	U	U
TCfollowup	NRS	Rutter 2012	Critical	Death	Death-FUR	?	?	?	U	U	?
TCfollowup	NRS	Shroff 2015	Critical	Death	Death-FUR	?	U	?	U	U	?
TCfollowup	RCT	Atkin 2002	Serious	Death	Death-FUR	?	?	?	U	U	?
TCfollowup	NRS	Dellon 2009	Serious	Death	Death-FUR	?	U	?	U	U	?
TCfollowup	RCT	Randel 2021	Serious	Death	Death-FUR	?	U	?	?	U	?
TConly	NRS	Berhane 2009	Critical	Death	Death-FUR	0	?	?	U	U	?
TConly	NRS	Imperiale 2000	Critical	Death	Death-NRFU	?	?	?	?	U	?
TConly	NRS	Lieberman 2000	Critical	Death	Death-FUR	?	?	U	U	U	?
TConly	NRS	Sieg 2006	Critical	Death	Death-FUR	?	?	?	U	U	?
TConly	NRS	Stock 2013	Critical	Death	Death-FUR	?	?	?	U	U	?
TConly	NRS	Strul 2006	Critical	Death	Death-NRFU	?	?	?	?	U	?
TConly	NRS	Waldmann 2016	Critical	Death	Death-NRFU	U	?	U	U	U	?
TConly	NRS	Zwink 2017	Critical	Death	Death-FUR	?	?	?	U	U	?
TConly	NRS	Pox 2012	Critical	Death	Death-NRFU	?	?	?	U	U	?
TConly	NRS	Rutter 2012	Critical	Death	Death-FUR	?	?	?	U	U	?
TConly	RCT	Stoop 2012	Critical	Death	Death-FUR	?	U	?	U	U	U
TConly	RCT	Bretthauer 2016	Moderate	Death	Death-FUR	?	?	U	U	U	?
TConly	NRS	Bokemeyer 2009	Serious	Death	Death-NRFU	U	?	?	U	U	?
TConly	NRS	Ferlitsch 2011	Serious	Death	Death-NRFU	U	?	?	?	U	?
TConly	NRS	Xirasagar 2020	Serious	Death	Death-NRFU	0	0	?	?	U	U
TConly	NRS	Wang 2018b	Serious	Death	Death-FUR	?	?	?	U	U	U
TConly	RCT	Kobiela 2020	Serious	Death	Death-FUR	?	?	U	?	?	?
TConly	NRS	Wang 2018a	Serious	Death	Death-FUR	?	?	?	U	U	?
TConly	NRS	Causada-Calo 2020	Serious	Death	Death-FUR	U	?	?	U	U	?
TConly	NRS	Pedersen 2020	Serious	Death	Death-FUR	U	?	?	U	U	?
TConly	NRS	Nelson 2002	Serious	Death	Death-FUR	?	?	?	U	U	?

Appendix 16 – Risk of bias in studies with assessments of CPEs

sTable 24. Risk of bias in studies with assessments of CPEs

Direction of effect: Unpredictable (?), Underestimate (U), Overestimate (O). **Risk of bias: Critical, serious, moderate, low**

Procedure	Design	Study ID	Worst score	Outcome	Analysis category	Inception	Classification	Performance	Missing data	Measurement	Reporting
FS	NRS	Levin 2002	Critical	Cardiopulmonary	ACS long-term	?	U	?	U	U	?
FS	RCT	Atkin 2002	Serious	Cardiopulmonary	ACS long-term	?	?	?	U	U	?
FS	NRS	Duku 2014	Critical	Cardiopulmonary	Follow-up time NR	?	?	?	U	U	?
FS	RCT	Segnan 2005	Critical	Cardiopulmonary	Follow-up time NR	?	?	?	U	U	?
FS	RCT	Gondal 2003	Critical	Cardiopulmonary	Follow-up time NR	?	?	?	U	U	?
FS	RCT	Atkin 1998	Serious	Cardiopulmonary	Follow-up time NR	?	?	?	U	?	?
FS	RCT	Atkin 1998	Serious	Cardiopulmonary	Follow-up time NR	?	?	?	U	?	?
FS	RCT	Atkin 2002	Serious	Cardiopulmonary	Follow-up time NR	?	?	?	U	U	?
FS	RCT	Senore 2011	Critical	Cardiopulmonary	NDCPE	?	?	?	?	U	?
FS	RCT	Atkin 2002	Serious	Cardiopulmonary	NDCPE	?	?	?	U	U	?
FS	RCT	Segnan 2005	Critical	Cardiopulmonary	other	?	?	?	U	U	?
FS	NRS	Pabby 2005	Serious	Cardiopulmonary	Vasovagal reaction short-term	U	U	?	U	U	?
FS	RCT	Segnan 2002	Serious	Cardiopulmonary	Vasovagal reaction short-term	?	?	?	U	U	?
TCfollowup	NRS	Castro 2013	Critical	Cardiopulmonary	arrhythmia long-term	?	?	?	U	U	?
TCfollowup	RCT	Segnan 2005	Critical	Cardiopulmonary	Follow-up time NR	?	?	?	U	U	?
TCfollowup	RCT	Gondal 2003	Critical	Cardiopulmonary	Follow-up time NR	?	?	?	U	U	?
TCfollowup	NRS	Shroff 2015	Critical	Cardiopulmonary	NDCPE	?	U	?	U	U	?
TCfollowup	NRS	Castro 2013	Critical	Cardiopulmonary	other	?	?	?	U	U	?
TCfollowup	RCT	Segnan 2002	Serious	Cardiopulmonary	Vasovagal reaction short-term	?	?	?	U	U	?

Procedure	Design	Study ID	Worst score	Outcome	Analysis category	Inception	Classification	Performance	Missing data	Measurement	Reporting
TCfobt	NRS	Denis 2013	Moderate	Cardiopulmonary	ACS long-term	?	?	?	U	U	?
TCfobt	NRS	Denis 2021	Serious	Cardiopulmonary	ACS long-term	?	U	?	U	U	?
TCfobt	NRS	Saraste 2016	Serious	Cardiopulmonary	ACS long-term	?	U	?	U	U	?
TCfobt	NRS	Tomaszewski 2021	Serious	Cardiopulmonary	ACS short-term	?	U	?	U	U	?
TCfobt	NRS	Denis 2013	Moderate	Cardiopulmonary	arrhythmia long-term	?	?	?	U	U	?
TCfobt	NRS	Denis 2021	Serious	Cardiopulmonary	arrhythmia long-term	?	U	?	U	U	?
TCfobt	NRS	Tomaszewski 2021	Serious	Cardiopulmonary	arrhythmia short-term	?	U	?	U	U	?
TCfobt	NRS	Lee 2012	Critical	Cardiopulmonary	Follow-up time NR	?	?	U	U	U	?
TCfobt	NRS	Gupta 2012	Serious	Cardiopulmonary	Follow-up time NR	U	?	?	U	U	?
TCfobt	NRS	Gupta 2012	Serious	Cardiopulmonary	Follow-up time NR	U	?	?	U	U	?
TCfobt	NRS	Hughes 2005	Serious	Cardiopulmonary	heart failure short-term	U	?	?	U	U	?
TCfobt	NRS	Benazzato 2020	Moderate	Cardiopulmonary	NDCPE	?	?	?	?	U	U
TCfobt	NRS	Tepes 2017	Serious	Cardiopulmonary	NDCPE	?	?	?	?	?	?
TCfobt	RCT	Robertson 2019	Serious	Cardiopulmonary	NDCPE	?	U	?	?	?	?
TCfobt	RCT	Dominitz 2019	Serious	Cardiopulmonary	NDCPE	?	?	?	?	?	?
TCfobt	NRS	Hsu 2020	Serious	Cardiopulmonary	NDCPE	?	?	?	U	U	?
TCfobt	NRS	Tomaszewski 2021	Serious	Cardiopulmonary	other	?	U	?	U	U	?
TCfobt	NRS	Denis 2021	Serious	Cardiopulmonary	Pulmonary event long-term	?	U	?	U	U	?
TCfobt	NRS	Tomaszewski 2021	Serious	Cardiopulmonary	Pulmonary event short-term	?	U	?	U	U	?
TCfobt	NRS	Saraste 2016	Serious	Cardiopulmonary	stroke long-term	?	U	?	U	U	?
TCfobt	NRS	Tomaszewski 2021	Serious	Cardiopulmonary	stroke short-term	?	U	?	U	U	?
TCfobt	NRS	Denis 2013	Moderate	Cardiopulmonary	TE long-term	?	?	?	U	U	?
TCfobt	NRS	Denis 2013	Moderate	Cardiopulmonary	TE long-term	?	?	?	U	U	?
TCfobt	NRS	Saraste 2016	Serious	Cardiopulmonary	TE long-term	?	U	?	U	U	?
TCfobt	NRS	Denis 2021	Serious	Cardiopulmonary	TE long-term	?	U	?	U	U	?
TCfobt	NRS	Tomaszewski 2021	Serious	Cardiopulmonary	TE short-term	?	U	?	U	U	?
TCfobt	NRS	Ibánez 2018	Serious	Cardiopulmonary	Vasovagal reaction long-term	?	?	?	U	U	?

Procedure	Design	Study ID	Worst score	Outcome	Analysis category	Inception	Classification	Performance	Missing data	Measurement	Reporting
TConly	NRS	Berhane 2009	Critical	Cardiopulmonary	ACS long-term	0	?	?	U	U	?
TConly	RCT	Stoop 2012	Critical	Cardiopulmonary	ACS long-term	?	U	?	U	U	U
TConly	NRS	Nelson 2002	Serious	Cardiopulmonary	ACS long-term	?	?	?	U	U	?
TConly	NRS	Stock 2013	Serious	Cardiopulmonary	ACS long-term	?	?	?	U	U	?
TConly	NRS	Wang 2018a	Serious	Cardiopulmonary	ACS long-term	?	?	?	U	U	?
TConly	NRS	Garcia-Albeniz 2017	Serious	Cardiopulmonary	ACS long-term	?	?	?	U	U	?
TConly	NRS	Causada-Calo 2020	Serious	Cardiopulmonary	ACS long-term	U	?	?	U	U	?
TConly	NRS	Garcia-Albeniz 2017	Serious	Cardiopulmonary	ACS long-term	?	?	?	U	U	?
TConly	NRS	Taleban 2018	Serious	Cardiopulmonary	ACS short-term	?	?	?	?	U	?
TConly	NRS	Lieberman 2000	Serious	Cardiopulmonary	ACS short-term	?	?	U	U	U	?
TConly	NRS	Pedersen 2020	Serious	Cardiopulmonary	ACS short-term	U	?	?	U	U	?
TConly	RCT	Stoop 2012	Critical	Cardiopulmonary	arrhythmia long-term	?	U	?	U	U	U
TConly	NRS	Garcia-Albeniz 2017	Serious	Cardiopulmonary	arrhythmia long-term	?	?	?	U	U	?
TConly	NRS	Causada-Calo 2020	Serious	Cardiopulmonary	arrhythmia long-term	U	?	?	U	U	?
TConly	NRS	Garcia-Albeniz 2017	Serious	Cardiopulmonary	arrhythmia long-term	?	?	?	U	U	?
TConly	NRS	Wang 2018a	Serious	Cardiopulmonary	arrhythmia long-term	?	?	?	U	U	?
TConly	NRS	Nelson 2002	Serious	Cardiopulmonary	arrhythmia long-term	?	?	?	U	U	?
TConly	NRS	Pedersen 2020	Serious	Cardiopulmonary	arrhythmia short-term	U	?	?	U	U	?
TConly	NRS	Taupin 2006	Critical	Cardiopulmonary	Follow-up time NR	?	?	?	?	U	?
TConly	RCT	Van Dam 2013	Critical	Cardiopulmonary	Follow-up time NR	U	?	?	U	U	U
TConly	NRS	Xirasagar 2020	Serious	Cardiopulmonary	Follow-up time NR	0	0	?	?	U	U
TConly	NRS	Garcia-Albeniz 2017	Serious	Cardiopulmonary	heart failure long-term	?	?	?	U	U	?
TConly	NRS	Garcia-Albeniz 2017	Serious	Cardiopulmonary	heart failure long-term	?	?	?	U	U	?
TConly	NRS	Causada-Calo 2020	Serious	Cardiopulmonary	heart failure long-term	U	?	?	U	U	?
TConly	NRS	Wang 2018a	Serious	Cardiopulmonary	heart failure long-term	?	?	?	U	U	?
TConly	NRS	Bokemeyer 2009	Critical	Cardiopulmonary	NDCPE	U	?	?	U	U	?
TConly	NRS	Crispin 2009	Critical	Cardiopulmonary	NDCPE	?	?	?	U	U	?
TConly	NRS	Sieg 2006	Critical	Cardiopulmonary	NDCPE	?	?	?	U	U	?
TConly	NRS	Pox 2012	Critical	Cardiopulmonary	NDCPE	?	?	?	U	U	?
TConly	NRS	Pox 2012	Critical	Cardiopulmonary	NDCPE	?	?	?	U	U	?
TConly	NRS	Kozbial 2015	Critical	Cardiopulmonary	NDCPE	U	?	?	U	U	U
TConly	RCT	Senore 2011	Critical	Cardiopulmonary	NDCPE	?	?	?	U	U	U
TConly	NRS	Khalid-de Bakker 2011	Serious	Cardiopulmonary	NDCPE	?	?	?	?	U	?
TConly	NRS	Khalid-de Bakker 2011b	Serious	Cardiopulmonary	NDCPE	?	?	?	U	U	?

Procedure	Design	Study ID	Worst score	Outcome	Analysis category	Inception	Classification	Performance	Missing data	Measurement	Reporting
TConly	NRS	Waldmann 2016	Serious	Cardiopulmonary	NDCPE	U	?	U	U	U	?
TConly	NRS	Ferlitsch 2011	Serious	Cardiopulmonary	NDCPE	U	?	?	?	U	?
TConly	NRS	Nelson 2002	Critical	Cardiopulmonary	other	?	?	?	U	?	?
TConly	NRS	Wang 2018a	Critical	Cardiopulmonary	other	?	?	?	U	?	?
TConly	NRS	Garcia-Albeniz 2017	Serious	Cardiopulmonary	other	?	?	?	U	U	?
TConly	NRS	Causada-Calo 2020	Serious	Cardiopulmonary	other	U	?	?	U	U	?
TConly	NRS	Garcia-Albeniz 2017	Serious	Cardiopulmonary	other	?	?	?	U	U	?
TConly	NRS	Causada-Calo 2020	Serious	Cardiopulmonary	other	U	?	?	U	U	?
TConly	NRS	Causada-Calo 2020	Serious	Cardiopulmonary	other	U	?	?	U	U	?
TConly	NRS	Pedersen 2020	Serious	Cardiopulmonary	other	U	?	?	U	U	?
TConly	NRS	Lieberman 2000	Serious	Cardiopulmonary	other	?	?	U	U	U	?
TConly	NRS	Causada-Calo 2020	Serious	Cardiopulmonary	other	U	?	?	U	U	?
TConly	RCT	Stoop 2012	Critical	Cardiopulmonary	Pulmonary event long-term	?	U	?	U	U	U
TConly	NRS	Wang 2018a	Serious	Cardiopulmonary	Pulmonary event long-term	?	?	?	U	U	?
TConly	NRS	Wang 2018b	Serious	Cardiopulmonary	Pulmonary event short-term	?	?	?	U	U	U
TConly	NRS	Wang 2018b	Serious	Cardiopulmonary	Pulmonary event short-term	?	?	?	U	U	U
TConly	NRS	Wang 2018b	Serious	Cardiopulmonary	Pulmonary event short-term	?	?	?	U	U	U
TConly	NRS	Pedersen 2020	Serious	Cardiopulmonary	Pulmonary event short-term	U	?	?	U	U	?
TConly	RCT	Stoop 2012	Critical	Cardiopulmonary	stroke long-term	?	U	?	U	U	U
TConly	NRS	Nelson 2002	Serious	Cardiopulmonary	stroke long-term	?	?	?	U	U	?
TConly	NRS	Wang 2018a	Serious	Cardiopulmonary	stroke long-term	?	?	?	U	U	?
TConly	NRS	Stock 2013	Serious	Cardiopulmonary	stroke long-term	?	?	?	U	U	?
TConly	NRS	Wang 2018a	Serious	Cardiopulmonary	stroke long-term	?	?	?	U	U	?
TConly	NRS	Lieberman 2000	Serious	Cardiopulmonary	stroke short-term	?	?	U	U	U	?
TConly	NRS	Pedersen 2020	Serious	Cardiopulmonary	stroke short-term	U	?	?	U	U	?
TConly	NRS	Causada-Calo 2020	Serious	Cardiopulmonary	TE long-term	U	?	?	U	U	?
TConly	NRS	Nelson 2002	Serious	Cardiopulmonary	TE long-term	?	?	?	U	U	?
TConly	NRS	Pedersen 2020	Serious	Cardiopulmonary	TE short-term	U	?	?	U	U	?
TConly	NRS	Garcia-Albeniz 2017	Serious	Cardiopulmonary	Vasovagal reaction long-term	?	?	?	U	U	?
TConly	NRS	Nelson 2002	Serious	Cardiopulmonary	Vasovagal reaction long-term	?	?	?	U	U	?
TConly	NRS	Garcia-Albeniz 2017	Serious	Cardiopulmonary	Vasovagal reaction long-term	?	?	?	U	U	?
TConly	NRS	Pedersen 2020	Critical	Cardiopulmonary	Vasovagal reaction short-term	U	?	?	U	U	?
TConly	RCT	Brethauer 2016	Moderate	Cardiopulmonary	Vasovagal reaction short-term	?	?	U	U	U	U

Appendix 17 – GRADE assessments of findings on deaths

sTable 25. GRADE assessment of the evidence on deaths associated with CRCSPs

	FS	TCfobt	TConly	TCfollowup
Death-AFU	-4	-2	-2	-5
Death FUR	-4	-5	-5	-5
Death NRFU	NA	-4	-3	NA

Abbreviations: Flexible sigmoidoscopy (FS), colonoscopy following FOBT/FIT (TCfobt), colposcopy without prior screening tests (TConly), colonoscopy following other types of screening tests than FOBT/FIT (TCfollowup), deaths with any follow-up time (Death-AFU), deaths with follow-up time reported (Death FUR), deaths without follow-up time reported (Death NRFU), Not Applicable due to lack of studies (NA).

sTable 26. GRADE rating of deaths-AFU during FS

Procedure	FS				
Outcome	Death				
Subcategory	Death-AFU				
No. Subpop	People	Events	Risk/100.000	Quality	Grading SUM
2	76739	6	8	very low	-4
Risk of bias	Inconsistency	Indirectness	Imprecision	Publications bias	Large effect
-2	0	NA	-1	-1	NA
Reasons for downgrading due to Inconsistency		0	OIS	12790	
Measurement tool					
Outcome assessor					
Country/setting					
Study year varies > 10 years					
Age distribution					
Expertise of endoscopists					
With/without anaesthesia					
Varying polypectomy rate					

sTable 27. GRADE rating of deaths-AFU during TConly

Procedure	TConly				
Outcome	Death				
Subcategory	Death-AFU				
No. Subpop	People	Events	Risk/100.000	Quality	Grading SUM
10	1548407	197	13	very low	-2
Risk of bias	Inconsistency	Indirectness	Imprecision	Publications bias	Large effect
-2	NA	NA	0	-1	1
Reasons for downgrading due to Inconsistency		0	OIS	7860	
Measurement tool					
Outcome assessor					
Country/setting					
Study year varies > 10 years					
Age distribution					
Expertise of endoscopists					
With/without anaesthesia					
Varying polypectomy rate					

sTable 28. GRADE rating of deaths-AFU during TCfobt

Procedure	TCfobt				
Outcome	Death				
Subcategory	Death-FUR				
No. Subpop	People	Events	Risk/100.000	Quality	Grading SUM
16	781533	82	10	very low	-2
Risk of bias	Inconsistency	Indirectness	Imprecision	Publications bias	Large effect
-2	NA	NA	0	-1	1
Reasons for downgrading due to Inconsistency		0	OIS	9531	
Measurement tool					
Outcome assessor					
Country/setting					
Study year varies > 10 years					
Age distribution					
Expertise of endoscopists					
With/without anaesthesia					
Varying polypectomy rate					

sTable 29. GRADE rating of deaths-AFU during TCfollowup

Procedure	TCfollowup				
Outcome	Death				
Subcategory	Death-AFU				
No. Subpop	People	Events	Risk/100.000	Quality	Grading SUM
3	8962	1	11	very low	-5
Risk of bias	Inconsistency	Indirectness	Imprecision	Publications bias	Large effect
-2	0	NA	-2	-1	NA
Reasons for downgrading due to Inconsistency		0	OIS	8962	
Measurement tool					
Outcome assessor					
Country/setting					
Study year varies > 10 years					
Age distribution					
Expertise of endoscopists					
With/without anaesthesia					
Varying polypectomy rate					

sTable 30. GRADE rating of deaths-FUR during FS

Procedure	FS				
Outcome	Death				
Subcategory	Death-FUR				
No. Subpop	People	Events	Risk/100.000	Quality	Grading SUM
2	76739	6	8	very low	-4
Risk of bias	Inconsistency	Indirectness	Imprecision	Publications bias	Large effect
-2	0	NA	-1	-1	NA
Reasons for downgrading due to Inconsistency		0	OIS	12790	
Measurement tool					
Outcome assessor					
Country/setting					
Study year varies > 10 years					
Age distribution					
Expertise of endoscopists					
With/without anaesthesia					
Varying polypectomy rate					

sTable 31. GRADE rating of deaths-FUR during TCfobt

Procedure	TCfobt				
Outcome	Death				
Subcategory	Death-FUR				
No. Subpop	People	Events	Risk/100.000	Quality	Grading SUM
14	748328	81	11	very low	-5
Risk of bias	Inconsistency	Indirectness	Imprecision	Publications bias	Large effect
-2	-1	NA	-1	-1	NA
Reasons for downgrading due to Inconsistency		2	OIS	9239	
Measurement tool	NO				
Outcome assessor	YES				
Country/setting	NO				
Study year varies > 10 years	NO				
Age distribution	NO				
Expertise of endoscopists	NO				
With/without anaesthesia	NO				
Varying polypectomy rate	YES				

sTable 32. GRADE rating of deaths-NRFU during TCfobt

Procedure	TCfobt				
Outcome	Death				
Subcategory	Death-NRFU				
No. Subpop	People	Events	Risk/100.000	Quality	Grading SUM
2	33205	1	3	very low	-4
Risk of bias	Inconsistency	Indirectness	Imprecision	Publications bias	Large effect
-2	0	NA	-1	-1	NA
Reasons for downgrading due to Inconsistency		0	OIS	33205	
Measurement tool					
Outcome assessor					
Country/setting					
Study year varies > 10 years					
Age distribution					
Expertise of endoscopists					
With/without anaesthesia					
Varying polypectomy rate					

sTable 33. GRADE rating of deaths-FUR during TCfollowup

Procedure	TCfollowup				
Outcome	Death				
Subcategory	Death-FUR				
No. Subpop	People	Events	Risk/100.000	Quality	Grading SUM
3	8962	1	11	very low	-5
Risk of bias	Inconsistency	Indirectness	Imprecision	Publications bias	Large effect
-2	0	NA	-2	-1	NA
Reasons for downgrading due to Inconsistency		0	OIS	8962	
Measurement tool					
Outcome assessor					
Country/setting					
Study year varies > 10 years					
Age distribution					
Expertise of endoscopists					
With/without anaesthesia					
Varying polypectomy rate					

sTable 34. GRADE rating of deaths-FUR during TConly

Procedure	TConly				
Outcome	Death				
Subcategory	Death-FUR				
No. Subpop	People	Events	Risk/100.000	Quality	Grading SUM
7	1211470	197	16	very low	-5
Risk of bias	Inconsistency	Indirectness	Imprecision	Publications bias	Large effect
-2	-2	NA	0	-1	NA
Reasons for downgrading due to Inconsistency		5	OIS	6150	
Measurement tool	NO				
Outcome assessor	YES				
Country/setting	YES				
Study year varies > 10 years	YES				
Age distribution	NO				
Expertise of endoscopists	YES				
With/without anaesthesia	NO				
Varying polypectomy rate	YES				

sTable 35. GRADE rating of deaths-NRFU during TConly

Procedure	TConly				
Outcome	Death				
Subcategory	Death-NRFU				
No. Subpop	People	Events	Risk/100.000	Quality	Grading SUM
3	336937	0	0	very low	-3
Risk of bias	Inconsistency	Indirectness	Imprecision	Publications bias	Large effect
-2	0	NA	0	-1	NA
Reasons for downgrading due to Inconsistency		0	OIS	NA	
Measurement tool					
Outcome assessor					
Country/setting					
Study year varies > 10 years					
Age distribution					
Expertise of endoscopists					
With/without anaesthesia					
Varying polypectomy rate					

Appendix 18 – GRADE assessments of findings on CPEs

sTable 36. GRADE assessment of the evidence on CPEs associated with CRCSPs

	FS	TCfobt	TConly	TCfollowup
ACS short-term	NA	-3	-5	NA
arrhythmia short-term	NA	-3	-3	NA
heart failure short-term	NA	-2	NA	NA
Pulmonary event short-term	NA	-3	-5	NA
stroke short-term	NA	-3	-4	NA
TE short-term	NA	-3	-3	NA
Vasovagal reaction short-term	-5	NA	-1	-2
ACS long-term	-3	-4	-6	NA
arrhythmia long-term	NA	-3	-6	NA
heart failure long-term	NA	NA	-6	NA
Pulmonary event long-term	NA	-3	-3	NA
stroke long-term	NA	-3	-4	NA
TE long-term	NA	-3	-3	NA
Vasovagal reaction long-term	NA	-3	-5	NA
NA: Not Applicable due to lack of studies				

sTable 37. Fulfilled downgrade criteria for inconsistency in GRADE assessments of CPEs

Downgrade criteria for inconsistency	Analyses with criterion, N
Measurement tool	5
Outcome assessor	7
Country/setting	2
Study year varies > 10 years	1
Age distribution	4
Expertise of endoscopists	5
With/without anaesthesia	5
Varying polypectomy rate	7

sTable 38. GRADE rating of CPE, vasovagal reaction short-term during FS

Procedure	FS				
Outcome	Cardiopulmonary				
Subcategory	Vasovagal reaction short-term				
No. Subpop	People	Events	Risk/100.000	Quality	Grading SUM
2	16879	140	829	very low	-5
Risk of bias	Inconsistency	Indirectness	Imprecision	Publications bias	Large effect
-2	-2	NA	-1	-1	1
Reasons for downgrading due to Inconsistency		4	OIS	2813	
Measurement tool	YES				
Outcome assessor	YES				
Country/setting	YES				
Study year varies > 10 years	NO				
Age distribution	NO				
Expertise of endoscopists	NO				
With/without anaesthesia	NO				
Varying polypectomy rate	YES				

sTable 39. GRADE rating of CPE, ACS long-term during FS

Procedure	FS				
Outcome	Cardiopulmonary				
Subcategory	ACS long-term				
No. Subpop	People	Events	Risk/100.000	Quality	Grading SUM
1	40674	2	5	very low	-3
Risk of bias	Inconsistency	Indirectness	Imprecision	Publications bias	Large effect
-2	NA	NA	0	-1	0
Reasons for downgrading due to Inconsistency		0	OIS	6779	
Measurement tool					
Outcome assessor					
Country/setting					
Study year varies > 10 years					
Age distribution					
Expertise of endoscopists					
With/without anaesthesia					
Varying polypectomy rate					

sTable 40. GRADE rating of CPE, ACS short-term during TCfobt

Procedure	TCfobt				
Outcome	Cardiopulmonary				
Subcategory	ACS short-term				
No. Subpop	People	Events	Risk/100.000	Quality	Grading SUM
1	78831	7	9	very low	-3
Risk of bias	Inconsistency	Indirectness	Imprecision	Publications bias	Large effect
-2	NA	NA	0	-1	0
Reasons for downgrading due to Inconsistency		0	OIS	11262	
Measurement tool					
Outcome assessor					
Country/setting					
Study year varies > 10 years					
Age distribution					
Expertise of endoscopists					
With/without anaesthesia					
Varying polypectomy rate					

sTable 41. GRADE rating of CPE, arrhythmia short-term during TCfobt

Procedure	TCfobt				
Outcome	Cardiopulmonary				
Subcategory	arrhythmia short-term				
No. Subpop	People	Events	Risk/100.000	Quality	Grading SUM
1	78831	4	5	very low	-3
Risk of bias	Inconsistency	Indirectness	Imprecision	Publications bias	Large effect
-2	NA	NA	0	-1	0
Reasons for downgrading due to Inconsistency		0	OIS	19708	
Measurement tool					
Outcome assessor					
Country/setting					
Study year varies > 10 years					
Age distribution					
Expertise of endoscopists					
With/without anaesthesia					
Varying polypectomy rate					

sTable 42. GRADE rating of CPE, heart failure short-term during TCfobt

Procedure	TCfobt				
Outcome	Cardiopulmonary				
Subcategory	heart failure short-term				
No. Subpop	People	Events	Risk/100.000	Quality	Grading SUM
1	92	1	1087	very low	-2
Risk of bias	Inconsistency	Indirectness	Imprecision	Publications bias	Large effect
-2	NA	NA	0	-1	1
Reasons for downgrading due to Inconsistency		0	OIS	92	
Measurement tool					
Outcome assessor					
Country/setting					
Study year varies > 10 years					
Age distribution					
Expertise of endoscopists					
With/without anaesthesia					
Varying polypectomy rate					

sTable 43. GRADE rating of CPE, pulmonary events short-term during TCfobt

Procedure	TCfobt				
Outcome	Cardiopulmonary				
Subcategory	Pulmonary event short-term				
No. Subpop	People	Events	Risk/100.000	Quality	Grading SUM
1	78831	1	1	very low	-3
Risk of bias	Inconsistency	Indirectness	Imprecision	Publications bias	Large effect
-2	NA	NA	0	-1	0
Reasons for downgrading due to Inconsistency		0	OIS	78831	
Measurement tool					
Outcome assessor					
Country/setting					
Study year varies > 10 years					
Age distribution					
Expertise of endoscopists					
With/without anaesthesia					
Varying polypectomy rate					

sTable 44. GRADE rating of CPE, stroke short-term during TCfobt

Procedure	TCfobt				
Outcome	Cardiopulmonary				
Subcategory	stroke short-term				
No. Subpop	People	Events	Risk/100.000	Quality	Grading SUM
1	78831	2	3	very low	-3
Risk of bias	Inconsistency	Indirectness	Imprecision	Publications bias	Large effect
-2	NA	NA	0	-1	0
Reasons for downgrading due to Inconsistency		0	OIS	39416	
Measurement tool					
Outcome assessor					
Country/setting					
Study year varies > 10 years					
Age distribution					
Expertise of endoscopists					
With/without anaesthesia					
Varying polypectomy rate					

sTable 45. GRADE rating of CPE, TE short-term during TCfobt

Procedure	TCfobt				
Outcome	Cardiopulmonary				
Subcategory	TE short-term				
No. Subpop	People	Events	Risk/100.000	Quality	Grading SUM
1	78831	2	3	very low	-3
Risk of bias	Inconsistency	Indirectness	Imprecision	Publications bias	Large effect
-2	NA	NA	0	-1	0
Reasons for downgrading due to Inconsistency		0	OIS	39416	
Measurement tool					
Outcome assessor					
Country/setting					
Study year varies > 10 years					
Age distribution					
Expertise of endoscopists					
With/without anaesthesia					
Varying polypectomy rate					

sTable 46. GRADE rating of CPE, ACS long-term during TCfobt

Procedure	TCfobt				
Outcome	Cardiopulmonary				
Subcategory	ACS long-term				
No. Subpop	People	Events	Risk/100.000	Quality	Grading SUM
3	22322	3	13	very low	-4
Risk of bias	Inconsistency	Indirectness	Imprecision	Publications bias	Large effect
-2	0	NA	-1	-1	0
Reasons for downgrading due to Inconsistency		0	OIS	7441	
Measurement tool					
Outcome assessor					
Country/setting					
Study year varies > 10 years					
Age distribution					
Expertise of endoscopists					
With/without anaesthesia					
Varying polypectomy rate					

sTable 47. GRADE rating of CPE, arrhythmia long-term during TCfobt

Procedure	TCfobt				
Outcome	Cardiopulmonary				
Subcategory	arrhythmia long-term				
No. Subpop	People	Events	Risk/100.000	Quality	Grading SUM
2	19338	4	21	very low	-3
Risk of bias	Inconsistency	Indirectness	Imprecision	Publications bias	Large effect
-1	0	NA	-1	-1	0
Reasons for downgrading due to Inconsistency		0	OIS	4835	
Measurement tool					
Outcome assessor					
Country/setting					
Study year varies > 10 years					
Age distribution					
Expertise of endoscopists					
With/without anaesthesia					
Varying polypectomy rate					

sTable 48. GRADE rating of CPE, pulmonary event long-term during TCfobt

Procedure	TCfobt				
Outcome	Cardiopulmonary				
Subcategory	Pulmonary event long-term				
No. Subpop	People	Events	Risk/100.000	Quality	Grading SUM
1	9061	1	11	very low	-3
Risk of bias	Inconsistency	Indirectness	Imprecision	Publications bias	Large effect
-2	NA	NA	0	-1	0
Reasons for downgrading due to Inconsistency		0	OIS	9061	
Measurement tool					
Outcome assessor					
Country/setting					
Study year varies > 10 years					
Age distribution					
Expertise of endoscopists					
With/without anaesthesia					
Varying polypectomy rate					

sTable 49. GRADE rating of CPE, stroke long-term during TCfobt

Procedure	TCfobt				
Outcome	Cardiopulmonary				
Subcategory	stroke long-term				
No. Subpop	People	Events	Risk/100.000	Quality	Grading SUM
1	2984	4	134	very low	-3
Risk of bias	Inconsistency	Indirectness	Imprecision	Publications bias	Large effect
-2	NA	NA	0	-1	0
Reasons for downgrading due to Inconsistency		0	OIS	746	
Measurement tool					
Outcome assessor					
Country/setting					
Study year varies > 10 years					
Age distribution					
Expertise of endoscopists					
With/without anaesthesia					
Varying polypectomy rate					

sTable 50. GRADE rating of CPE, TE long-term during TCfobt

Procedure	TCfobt				
Outcome	Cardiopulmonary				
Subcategory	TE long-term				
No. Subpop	People	Events	Risk/100.000	Quality	Grading SUM
4	32599	6	18	very low	-3
Risk of bias	Inconsistency	Indirectness	Imprecision	Publications bias	Large effect
-1	0	NA	-1	-1	0
Reasons for downgrading due to Inconsistency		0	OIS	5433	
Measurement tool					
Outcome assessor					
Country/setting					
Study year varies > 10 years					
Age distribution					
Expertise of endoscopists					
With/without anaesthesia					
Varying polypectomy rate					

sTable 51. GRADE rating of CPE, vasovagal reaction long-term during TCfobt

Procedure	TCfobt				
Outcome	Cardiopulmonary				
Subcategory	Vasovagal reaction long-term				
No. Subpop	People	Events	Risk/100.000	Quality	Grading SUM
1	7467	0	0	very low	-3
Risk of bias	Inconsistency	Indirectness	Imprecision	Publications bias	Large effect
-2	NA	NA	0	-1	0
Reasons for downgrading due to Inconsistency		0	OIS	NA	
Measurement tool					
Outcome assessor					
Country/setting					
Study year varies > 10 years					
Age distribution					
Expertise of endoscopists					
With/without anaesthesia					
Varying polypectomy rate					

sTable 52. GRADE rating of CPE, vasovagal reaction short-term during TCfollowup

Procedure	TCfollowup				
Outcome	Cardiopulmonary				
Subcategory	Vasovagal reaction short-term				
No. Subpop	People	Events	Risk/100.000	Quality	Grading SUM
1	775	7	903	very low	-2
Risk of bias	Inconsistency	Indirectness	Imprecision	Publications bias	Large effect
-2	NA	NA	0	-1	1
Reasons for downgrading due to Inconsistency		0	OIS	111	
Measurement tool					
Outcome assessor					
Country/setting					
Study year varies > 10 years					
Age distribution					
Expertise of endoscopists					
With/without anaesthesia					
Varying polypectomy rate					

sTable 53. GRADE rating of CPE, ACS short-term during TConly

Procedure	TConly				
Outcome	Cardiopulmonary				
Subcategory	ACS short-term				
No. Subpop	People	Events	Risk/100.000	Quality	Grading SUM
3	14383	2	14	very low	-5
Risk of bias	Inconsistency	Indirectness	Imprecision	Publications bias	Large effect
-2	0	NA	-2	-1	0
Reasons for downgrading due to Inconsistency		0	OIS	7192	
Measurement tool					
Outcome assessor					
Country/setting					
Study year varies > 10 years					
Age distribution					
Expertise of endoscopists					
With/without anaesthesia					
Varying polypectomy rate					

sTable 54. GRADE rating of CPE, arrhythmia short-term during TConly

Procedure	TConly				
Outcome	Cardiopulmonary				
Subcategory	arrhythmia short-term				
No. Subpop	People	Events	Risk/100.000	Quality	Grading SUM
1	11163	21	188	very low	-3
Risk of bias	Inconsistency	Indirectness	Imprecision	Publications bias	Large effect
-2	NA	NA	0	-1	0
Reasons for downgrading due to Inconsistency		0	OIS	532	
Measurement tool					
Outcome assessor					
Country/setting					
Study year varies > 10 years					
Age distribution					
Expertise of endoscopists					
With/without anaesthesia					
Varying polypectomy rate					

sTable 55. GRADE rating of CPE, pulmonary event short-term during TConly

Procedure	TConly				
Outcome	Cardiopulmonary				
Subcategory	Pulmonary event short-term				
No. Subpop	People	Events	Risk/100.000	Quality	Grading SUM
4	1373976	925	67	very low	-5
Risk of bias	Inconsistency	Indirectness	Imprecision	Publications bias	Large effect
-2	-2	NA	0	-1	0
Reasons for downgrading due to Inconsistency		5	OIS	1485	
Measurement tool	NO				
Outcome assessor	YES				
Country/setting	YES				
Study year varies > 10 years	NO				
Age distribution	YES				
Expertise of endoscopists	YES				
With/without anaesthesia	NO				
Varying polypectomy rate	YES				

sTable 56. GRADE rating of CPE, stroke short-term during TConly

Procedure	TConly				
Outcome	Cardiopulmonary				
Subcategory	stroke short-term				
No. Subpop	People	Events	Risk/100.000	Quality	Grading SUM
2	14284	6	42	very low	-4
Risk of bias	Inconsistency	Indirectness	Imprecision	Publications bias	Large effect
-2	0	NA	-1	-1	0
Reasons for downgrading due to Inconsistency		5	OIS	2381	
Measurement tool					
Outcome assessor					
Country/setting					
Study year varies > 10 years					
Age distribution					
Expertise of endoscopists					
With/without anaesthesia					
Varying polypectomy rate					

sTable 57. GRADE rating of CPE, TE short-term during TConly

Procedure	TConly				
Outcome	Cardiopulmonary				
Subcategory	TE short-term				
No. Subpop	People	Events	Risk/100.000	Quality	Grading SUM
1	11163	5	45	very low	-3
Risk of bias	Inconsistency	Indirectness	Imprecision	Publications bias	Large effect
-2	NA	NA	0	-1	0
Reasons for downgrading due to Inconsistency		0	OIS	2233	
Measurement tool					
Outcome assessor					
Country/setting					
Study year varies > 10 years					
Age distribution					
Expertise of endoscopists					
With/without anaesthesia					
Varying polypectomy rate					

sTable 58. GRADE rating of CPE, vasovagal reaction short-term during TConly

Procedure	TConly				
Outcome	Cardiopulmonary				
Subcategory	Vasovagal reaction short-term				
No. Subpop	People	Events	Risk/100.000	Quality	Grading SUM
1	11912	51	428	very low	-1
Risk of bias	Inconsistency	Indirectness	Imprecision	Publications bias	Large effect
0	NA	NA	0	-1	0
Reasons for downgrading due to Inconsistency		0	OIS	234	
Measurement tool					
Outcome assessor					
Country/setting					
Study year varies > 10 years					
Age distribution					
Expertise of endoscopists					
With/without anaesthesia					
Varying polypectomy rate					

sTable 59. GRADE rating of CPE, ACS long-term during TConly

Procedure	TConly				
Outcome	Cardiopulmonary				
Subcategory	ACS long-term				
No. Subpop	People	Events	Risk/100.000	Quality	Grading SUM
6	765457	346	45	very low	-6
Risk of bias	Inconsistency	Indirectness	Imprecision	Publications bias	Large effect
-2	-2	NA	-1	-1	0
Reasons for downgrading due to Inconsistency		6	OIS	2212	
Measurement tool	YES				
Outcome assessor	YES				
Country/setting	NO				
Study year varies > 10 years	NO				
Age distribution	YES				
Expertise of endoscopists	YES				
With/without anaesthesia	YES				
Varying polypectomy rate	YES				

sTable 60. GRADE rating of CPE, arrhythmia long-term during TConly

Procedure	TConly				
Outcome	Cardiopulmonary				
Subcategory	arrhythmia long-term				
No. Subpop	People	Events	Risk/100.000	Quality	Grading SUM
5	756799	2136	282	very low	-6
Risk of bias	Inconsistency	Indirectness	Imprecision	Publications bias	Large effect
-2	-2	NA	-1	-1	0
Reasons for downgrading due to Inconsistency		6	OIS	354	
Measurement tool	YES				
Outcome assessor	YES				
Country/setting	NO				
Study year varies > 10 years	NO				
Age distribution	YES				
Expertise of endoscopists	YES				
With/without anaesthesia	YES				
Varying polypectomy rate	YES				

sTable 61. GRADE rating of CPE, heart failure long-term during TConly

Procedure	TConly				
Outcome	Cardiopulmonary				
Subcategory	heart failure long-term				
No. Subpop	People	Events	Risk/100.000	Quality	Grading SUM
4	753603	946	126	very low	-6
Risk of bias	Inconsistency	Indirectness	Imprecision	Publications bias	Large effect
-2	-2	NA	-1	-1	0
Reasons for downgrading due to Inconsistency		6	OIS	797	
Measurement tool	YES				
Outcome assessor	YES				
Country/setting	NO				
Study year varies > 10 years	NO				
Age distribution	YES				
Expertise of endoscopists	YES				
With/without anaesthesia	YES				
Varying polypectomy rate	YES				

sTable 62. GRADE rating of CPE, pulmonary event long-term during TConly

Procedure	TConly				
Outcome	Cardiopulmonary				
Subcategory	Pulmonary event long-term				
No. Subpop	People	Events	Risk/100.000	Quality	Grading SUM
1	645095	1317	204	very low	-3
Risk of bias	Inconsistency	Indirectness	Imprecision	Publications bias	Large effect
-2	NA	NA	0	-1	0
Reasons for downgrading due to Inconsistency		0	OIS	490	
Measurement tool					
Outcome assessor					
Country/setting					
Study year varies > 10 years					
Age distribution					
Expertise of endoscopists					
With/without anaesthesia					
Varying polypectomy rate					

sTable 63. GRADE rating of CPE, stroke long-term during TConly

Procedure	TConly				
Outcome	Cardiopulmonary				
Subcategory	stroke long-term				
No. Subpop	People	Events	Risk/100.000	Quality	Grading SUM
4	1302044	764	59	very low	-4
Risk of bias	Inconsistency	Indirectness	Imprecision	Publications bias	Large effect
-2	-1	NA	0	-1	0
Reasons for downgrading due to Inconsistency		3	OIS	1704	
Measurement tool	NO				
Outcome assessor	YES				
Country/setting	NO				
Study year varies > 10 years	NO				
Age distribution	NO				
Expertise of endoscopists	NO				
With/without anaesthesia	YES				
Varying polypectomy rate	YES				

sTable 64. GRADE rating of CPE, TE long-term during TConly

Procedure	TConly				
Outcome	Cardiopulmonary				
Subcategory	TE long-term				
No. Subpop	People	Events	Risk/100.000	Quality	Grading SUM
2	33639	26	77	very low	-3
Risk of bias	Inconsistency	Indirectness	Imprecision	Publications bias	Large effect
-2	0	NA	0	-1	0
Reasons for downgrading due to Inconsistency		0	OIS	1294	
Measurement tool					
Outcome assessor					
Country/setting					
Study year varies > 10 years					
Age distribution					
Expertise of endoscopists					
With/without anaesthesia					
Varying polypectomy rate					

sTable 65. GRADE rating of CPE, vasovagal reaction long-term during TConly

Procedure	TConly				
Outcome	Cardiopulmonary				
Subcategory	Vasovagal reaction long-term				
No. Subpop	People	Events	Risk/100.000	Quality	Grading SUM
3	81261	423	521	very low	-5
Risk of bias	Inconsistency	Indirectness	Imprecision	Publications bias	Large effect
-2	-2	NA	-1	-1	1
Reasons for downgrading due to Inconsistency		6	OIS	192	
Measurement tool	YES				
Outcome assessor	YES				
Country/setting	NO				
Study year varies > 10 years	YES				
Age distribution	NO				
Expertise of endoscopists	YES				
With/without anaesthesia	YES				
Varying polypectomy rate	YES				

Appendix 19 – Studies identified and harms assessed in other systematic reviews

sTable 66. Harms assessed in other systematic reviews:

Study ID	RCTs	NRS	Studies, N	**N, %********	FS	TCfobt	TConly	TCfollowup	Death	CPE	Perforation	Major bleeding	Minor bleeding	Death < 30 days of surgery	Pain
Lin 2021	unclear		106	79%	YES	YES	YES	YES	NO	NO	YES	YES	NO	NO	NO
Lin 2016	13	22	35	26%	YES	YES	YES	YES	NO	NO	YES	YES	NO	NO	NO
Reumkens 2016***	0	7	7	5%	NO	NO	YES	NO	NO	NO	YES	YES	NO	NO	NO
Tinmouth 2016	13	3	16	12%	YES	YES	YES	NO	NO	NO	NO	NO	NO	NO	NO
Fitzpatrick-Lewis 2016	5	17	22	16%	YES	YES	YES	NO	YES	NO	YES	YES	YES	NO	NO
Hewitson 2007	3	0	3	2%	NO	YES	NO	NO	NO	NO	NO	NO	NO	NO	NO
Holme 2013	8	0	8	6%	YES	YES	NO	NO	YES	NO	YES	YES	NO	YES	NO
Vermeer 2017	7	20	27	20%	NO	YES	YES	NO	NO	NO	YES	YES	NO	YES	NO
Niv 2008**	0	3	3	2%	NO	NO	YES	NO	NO	NO	YES	YES	NO	NO	NO
Jodal 2019*	6	1	7	5%	YES	YES	YES	NO	YES	NO	NO	NO	NO	YES	YES

*Distinction between gFOBT per 2-5 screening rounds, gFOBT per screening test and FIT per screening test in quantitative analyses of harm
**Death described narratively in one of the 10 included studies
***Death rates "not applicable" for colonoscopy for screening purpose, not further explained
**** Compared to the 134 publications in this review

Types of harm assessed in other reviews:

1) Serious adverse events requiring unexpected or unwanted medical attention, and/or resulting in death. These events included, but were not limited to, perforation, major bleeding, severe abdominal symptoms, and cardiovascular events. We excluded studies whose reported harms were limited to minor adverse events that did not necessarily result in medical attention (e.g., patient dissatisfaction, worry, minor gastrointestinal complaints), physiologic outcomes only (e.g., hypoxia, renal or electrolyte disturbances), or harms of health certificate effect (i.e., persons with negative screening result engaging in risky health behaviors or not pursuing future screening).
2) Pooled prevalence of perforations, post-colonoscopy bleeding, post-polypectomy bleeding, and mortality.
3) Referring to Holme 2013 concerning complication rates
4) Harms: complications (bleeding or perforation) of the test or the follow-up test, false positives, false negatives, over-diagnosis, death
5) Narrative report of harm noted by trial investigators
6) Bleeding, perforation, death < 30 days after screening procedure, death < 30 days of surgery, major complications (not further defined), Miscellaneous Major adverse events narratively reported: Bleeding, perforation and death excluded 2 myocardial infarction, 1 pulmonary embolus Other types of harm narratively reported: Snare entrapment Minor events not requiring hospitalisation 5 cases of definite glutaraldehyde colitis and 8 probable cases 14 patients who had a laparotomy had complications which prolonged their hospital stay
7) Complications after colonoscopy, morbidity and mortality following surgery, psychological distress and inappropriate use of the screening test. Other types of harm narratively reported: Other complications from colonoscopy were reported in eighteen studies, including cardiovascular events, postpolypectomy syndrome, vasovagal reactions or abdominal pain or discomfort. None of the included studies reported any mortality after colonoscopy.
8) Complications (perforation, bleeding, and death).
9) Outcomes of interest were colorectal cancer incidence and mortality, all-cause mortality, harms (bleeding, perforation, screening-related death and other major and minor complications as reported by trial authors) and burdens (need for further diagnostic workup including colonoscopy, procedure-related pain, psychological impact of a positive test and absence from work to prepare, perform and recover after the screening procedure). Major adverse events narratively reported: Bleeding, perforation and death excluded. Sigmoidoscopy: two myocardial infarctions, one pulmonary embolus, one burnt serosa syndrome and one fever of unknown cause. FIT: two individuals with hypotension or bradycardia. Colonoscopy: 10 individuals with hypotension or bradycardia, one desaturation. Other types of harm narratively reported: Includes snare entrapment, vasovagal reactions, glutaraldehyde colitis and other events not requiring hospitalisation.

Appendix 20 - Deaths associated with CRCSPs compared to other reviews

sTable 67. Deaths associated with CRCSPs compared to former systematic reviews

Deaths associated with once-only sigmoidoscopy				
	Events*	People	Events/100.000 people	GRADE
Current review, Death FUR*	17	202.933	3, [0-87]	-4
Holme 2013	6	172.871	3	
Jodal 2019	7	40.674	17	
FL 2016**	6	40.332	15	
Deaths associated with colonoscopy following FOBT				
	Events	People	Events/100.000 people	GRADE
Current review, Death FUR*	83	830.233	4, [1-11]	-5
Holme 2013	1	37.560	3	
Jodal 2019	0	68.754	<1	
FL 2016**	10	39.561	25	
Deaths associated with once-only colonoscopy				
	Events	People	Events/100.000 people	GRADE
Current review, Death FUR*	220	4.658.351	23, [10-55]	-5
Jodal 2019	0	12.574	<1	
FL 2016**	14	109.076	13	
*All studies with follow-up time reported were compiled regardless of risk of bias and study design<br>**FL = Fitzpatrick-Lewis. Events/procedure converted to events/people and added together				

References

1. Zorzela L, Loke YK, Ioannidis JP, Golder S, Santaguida P, Altman DG, et al. PRISMA harms checklist: improving harms reporting in systematic reviews. BMJ. 2016;352:i157. Epub 2016/02/03. PubMed PMID: 26830668.
2. Harris RP, Sheridan SL, Lewis CL, Barclay C, Vu MB, Kistler CE, et al. The harms of screening: a proposed taxonomy and application to lung cancer screening. JAMA Intern Med. 2014;174(2):281-5. Epub 2013/12/11. doi: 10.1001/jamainternmed.2013.12745. PubMed PMID: 24322781.
3. Rajendran A, Rajaratnam R, Thomas-Gibson S, Humphries A, Wilson A, Vance M, et al. Sa1716 Post Polypectomy Bleeding (Ppb) Following Screening Colonoscopy: Experience at a Single Centre in the United Kingdom...Digestive Disease Week (DDW) 2017 American Society for Gastrointestinal Endoscopy (ASGE) Program and Abstracts, Chicago, Illinois, 6–9 May 2017. Gastrointestinal endoscopy. 2017;85:AB252-AB. doi: 10.1016/j.gie.2017.03.567.
4. Ahmed S, Naumann DN, Karandikar S. Differences in screening vs non-screening colonoscopy: scope for improvement? Colorectal Dis. 2016;18(9):903-9. Epub 2016/02/07. doi: 10.1111/codi.13291. PubMed PMID: 26850216.
5. Reumkens A, Rondagh EJ, Bakker CM, Winkens B, Masclee AA, Sanduleanu S. Post-Colonoscopy Complications: A Systematic Review, Time Trends, and Meta-Analysis of Population-Based Studies. The American journal of gastroenterology. 2016;111(8):1092-101. Epub 2016/06/15. doi: 10.1038/ajg.2016.234. PubMed PMID: 27296945.
6. Hunter JP, Saratzis A, Sutton AJ, Boucher RH, Sayers RD, Bown MJ. In meta-analyses of proportion studies, funnel plots were found to be an inaccurate method of assessing publication bias. J Clin Epidemiol. 2014;67(8):897-903. Epub 2014/05/06. doi: 10.1016/j.jclinepi.2014.03.003. PubMed PMID: 24794697.
7. The UK NSC recommendation on Bowel Cancer screening in adults 2018. Available from: https://legacyscreening.phe.org.uk/bowelcancer.
8. Atkin WS, Hart A, Edwards R, McIntyre P, Aubrey R, Wardle J, et al. Uptake, yield of neoplasia, and adverse effects of flexible sigmoidoscopy screening. Gut. 1998;42(4):560-5. Epub 1998/06/09. PubMed PMID: 9616321; PubMed Central PMCID: PMCPMC1727083.
9. Atkin WS, Cook CF, Cuzick J, Edwards R, Northover JMA, Wardle J. Single flexible sigmoidoscopy screening to prevent colorectal cancer: baseline findings of a UK multicentre randomised trial. Lancet. 2002;359:1291-300. Epub 2002/04/20. doi: 10.1016/s0140-6736(02)08268-5. PubMed PMID: 11965274.
10. Blom J, Lidén A, Nilsson J, Påhlman L, Nyrén O, Holmberg L. Colorectal cancer screening with flexible sigmoidoscopy-participants' experiences and technical feasibility. Eur J Surg Oncol. 2004;30(4):362-9. Epub 2004/04/06. doi: 10.1016/j.ejso.2004.01.005. PubMed PMID: 15063888.
11. Collett JA, Olynyk JK, Platell CF. Flexible sigmoidoscopy screening for colorectal cancer in average-risk people: update of a community-based project. Med J Aust. 2000;173(9):463-6. Epub 2001/01/10. PubMed PMID: 11149301.
12. Duku M, Ng D, George SD, Hughes T, Tierney K, Turnbull S, et al. PWE-014 Jesrey Flexible Sigmoidoscopy Bowel Cancer Programme: One Year’s Experience. Gut. 2014;63(Suppl 1):A127. doi: 10.1136/gutjnl-2014-307263.274.
13. Eloubeidi MA, Wallace MB, Desmond R, Farraye FA. Female gender and other factors predictive of a limited screening flexible sigmoidoscopy examination for colorectal cancer. The American journal of gastroenterology. 2003;98(7):1634-9. Epub 2003/07/23. doi: 10.1111/j.1572-0241.2003.07480.x. PubMed PMID: 12873591.
14. Forbes M, group M. A comparison of colorectal neoplasia screening tests: a multicentre community-based study of the impact of consumer choice. Med J Aust. 2006;184(11):546-50. Epub 2006/06/14. PubMed PMID: 16768659.

15. Gondal G, Grotmol T, Hofstad B, Bretthauer M, Eide TJ, Hoff G. The Norwegian Colorectal Cancer Prevention (NORCCAP) screening study: baseline findings and implementations for clinical work-up in age groups 50-64 years. Scand J Gastroenterol. 2003;38:635-42.
16. Hoff G, Grotmol T, Skovlund E, Bretthauer M. Risk of colorectal cancer seven years after flexible sigmoidoscopy screening: randomised controlled trial. BMJ. 2009;338:b1846. doi: 10.1136/bmj.b1846.
17. Hol L, van Leerdam ME, van Ballegooijen M, van Vuuren AJ, van Dekken H, Reijerink JC, et al. Screening for colorectal cancer: randomised trial comparing guaiac-based and immunochemical faecal occult blood testing and flexible sigmoidoscopy. Gut. 2010;59(1):62-8. Epub 2009/08/13. doi: 10.1136/gut.2009.177089. PubMed PMID: 19671542.
18. Holme Ø, Løberg M, Kalager M, Bretthauer M, Hernán MA, Aas E, et al. Effect of Flexible Sigmoidoscopy Screening on Colorectal Cancer Incidence and Mortality: A Randomized Clinical Trial. JAMA. 2014;312(6):606-15. doi: 10.1001/jama.2014.8266.
19. Jain A, Falzarano J, Jain A, Decker R, Okubo G, Fujiwara D. Outcome of 5,000 flexible sigmoidoscopies done by nurse endoscopists for colorectal screening in asymptomatic patients. Hawaii Med J. 2002;61:118-20. Epub 2002/08/01. PubMed PMID: 12148407.
20. Kewenter J, Brevinge H. Endoscopic and surgical complications of work-up in screening for colorectal cancer. Dis Colon Rectum. 1996;39(6):676-80. Epub 1996/06/01. doi: 10.1007/bf02056949. PubMed PMID: 8646956.
21. Larsen IK, Grotmol T, Bretthauer M, Gondal G, Huppertz-Hauss G, Hofstad B, et al. Continuous evaluation of patient satisfaction in endoscopy centres. Scand J Gastroenterol. 2002;37(7):850-5. Epub 2002/08/23. PubMed PMID: 12190102.
22. Levin TR, Conell C, Shapiro JA, Chazan SG, Nadel MR, Selby JV. Complications of screening flexible sigmoidoscopy. Gastroenterology. 2002;123(6):1786-92. Epub 2002/11/28. doi: 10.1053/gast.2002.37064. PubMed PMID: 12454834.
23. Olynyk JK, Aquilia S, Fletcher DR, Dickinson JA. Flexible sigmoidoscopy screening for colorectal cancer in average-risk subjects: a community-based pilot project. Med J Aust. 1996;165(2):74-6. Epub 1996/07/15. PubMed PMID: 8692065.
24. Pabby A, Suneja A, Heeren T, Farraye FA. Flexible sigmoidoscopy for colorectal cancer screening in the elderly. Dig Dis Sci. 2005;50(11):2147-52. Epub 2005/10/22. doi: 10.1007/s10620-005-3022-x. PubMed PMID: 16240230.
25. Randel KR, Botteri E, Natvig E, Berstad P, Jorgensen A, Darre-Naess O, et al. Colorectal Cancer Screening With Repeated Fecal Immunochemical Test Versus Sigmoidoscopy: Baseline Results From a Randomized Trial. Gastroenterology. 2021;160(4):1085-. doi: http://dx.doi.org/10.1053/j.gastro.2020.11.037.
26. Rasmussen M, Kronborg O, Fenger C, Jørgensen OD. Possible advantages and drawbacks of adding flexible sigmoidoscopy to hemoccult-II in screening for colorectal cancer. A randomized study. Scand J Gastroenterol. 1999;34(1):73-8. Epub 1999/02/27. doi: 10.1080/00365529950172862. PubMed PMID: 10048736.
27. Robb KA, Lo SH, Power E, Kralj-Hans I, Edwards R, Vance M, et al. Patient-reported outcomes following flexible sigmoidoscopy screening for colorectal cancer in a demonstration screening programme in the UK. J Med Screen. 2012;19(4):171-6. Epub 2012/01/01. doi: 10.1258/jms.2012.012129. PubMed PMID: 23486697.
28. Santavirta J. Screening sigmoidoscopy in persons aged 60 years. Colorectal Disease. 2002;4(3):184-8. doi: https://doi.org/10.1046/j.1463-1318.2002.00298.x.
29. Schoen RE, Weissfeld JL, Bowen NJ, Switzer G, Baum A. Patient satisfaction with screening flexible sigmoidoscopy. Arch Intern Med. 2000;160(12):1790-6. Epub 2000/06/29. doi: 10.1001/archinte.160.12.1790. PubMed PMID: 10871972.

30. Schoen RE, Pinsky PF, Weissfeld JL, Yokochi LA, Church T, Laiyemo AO, et al. Colorectal-cancer incidence and mortality with screening flexible sigmoidoscopy. N Engl J Med. 2012;366(25):2345-57. PubMed PMID: 22612596.
31. Segnan N, Senore C, Andreoni B, Aste H, Bonelli L, Crosta C, et al. Baseline findings of the Italian multicenter randomized controlled trial of "once-only sigmoidoscopy"--SCORE. J Natl Cancer Inst. 2002;94(23):1763-72. Epub 2002/12/05. PubMed PMID: 12464648.
32. Segnan N, Senore C, Andreoni B, Arrigoni A, Bisanti L, Cardelli A, et al. Randomized trial of different screening strategies for colorectal cancer: patient response and detection rates. J Natl Cancer Inst. 2005;97(5):347-57. Epub 2005/03/03. doi: 10.1093/jnci/dji050. PubMed PMID: 15741571.
33. Senore C, Ederle A, Fantin A, Andreoni B, Bisanti L, Grazzini G, et al. Acceptability and side-effects of colonoscopy and sigmoidoscopy in a screening setting. J Med Screen. 2011;18(3):128-34. Epub 2011/11/03. doi: 10.1258/jms.2011.010135. PubMed PMID: 22045821.
34. Viiala CH, Olynyk JK. Outcomes for women in a flexible sigmoidoscopy-based colorectal cancer screening programme. Intern Med J. 2008;38(2):90-4. Epub 2007/10/06. doi: 10.1111/j.1445-5994.2007.01468.x. PubMed PMID: 17916176.
35. Zubarik R, Ganguly E, Benway D, Ferrentino N, Moses P, Vecchio J. Procedure-related abdominal discomfort in patients undergoing colorectal cancer screening: a comparison of colonoscopy and flexible sigmoidoscopy. The American journal of gastroenterology. 2002;97(12):3056-61. Epub 2002/12/21. doi: 10.1111/j.1572-0241.2002.07101.x. PubMed PMID: 12492190.
36. Arana-Arri E, Imaz-Ayo N, Fernández MJ, Idigoras I, Bilbao I, Bujanda L, et al. Screening colonoscopy and risk of adverse events among individuals undergoing fecal immunochemical testing in a population-based program: A nested case-control study. United European Gastroenterol J. 2018;6(5):755-64. PubMed PMID: 30083338.
37. Benazzato L, Zorzi M, Guzzinati S, Antonelli G, Hassan C, Fantin A. Colonoscopy-related adverse events and mortality in an Italian organized colorectal cancer screening program. Endoscopy. 2021;53(5):501-8. doi: http://dx.doi.org/10.1055/a-1228-9225.
38. Binefa G, García M, Milà N, Rodríguez L, Rodríguez-Moranta F, Guardiola J, et al. Colonoscopy quality assessment in a mass population screening programme based on faecal occult blood test. Rev Esp Enferm Dig. 2013;105(7):400-8. Epub 2013/11/12. doi: 10.4321/s1130-01082013000700005. PubMed PMID: 24206550.
39. Cheng TI, Wong JM, Hong CF, Cheng SH, Cheng TJ, Shieh MJ, et al. Colorectal cancer screening in asymptomaic adults: comparison of colonoscopy, sigmoidoscopy and fecal occult blood tests. J Formos Med Assoc. 2002;101(10):685-90. Epub 2003/01/09. PubMed PMID: 12517041.
40. Dancourt V, Lejeune C, Lepage C, Gailliard MC, Meny B, Faivre J. Immunochemical faecal occult blood tests are superior to guaiac-based tests for the detection of colorectal neoplasms. European journal of cancer (Oxford, England : 1990). 2008;44(15):2254-8. Epub 2008/09/02. doi: 10.1016/j.ejca.2008.06.041. PubMed PMID: 18760592.
41. Denis B, Ruetsch M, Strentz P, Vogel JY, Guth F, Boyaval JM, et al. Short term outcomes of the first round of a pilot colorectal cancer screening programme with guaiac based faecal occult blood test. Gut. 2007;56(11):1579-84. PubMed PMID: 17616542.
42. Denis B, Gendre I, Sauleau EA, Lacroute J, Perrin P. Harms of colonoscopy in a colorectal cancer screening programme with faecal occult blood test: a population-based cohort study. Digestive and liver disease : official journal of the Italian Society of Gastroenterology and the Italian Association for the Study of the Liver. 2013;45(6):474-80. Epub 2013/02/19. doi: 10.1016/j.dld.2013.01.006. PubMed PMID: 23414583.
43. Denis B, Gendre I, Weber S, Perrin P. Adverse events of colonoscopy in a colorectal cancer screening program with fecal immunochemical testing: a population-based observational study. Endosc Int Open. 2021;9(2):E224-e32. Epub 2021/02/09. doi: 10.1055/a-1324-2763

10.1055/a-1324-2763. Epub 2021 Feb 3.

44. Denters MJ, Deutekom M, Bossuyt PM, Stroobants AK, Fockens P, Dekker E. Lower risk of advanced neoplasia among patients with a previous negative result from a fecal test for colorectal cancer. Gastroenterology. 2012;142(3):497-504. Epub 2011/11/24. doi: 10.1053/j.gastro.2011.11.024. PubMed PMID: 22108194.
45. Denters MJ, Deutekom M, Bossuyt PM, Fockens P, Dekker E. Patient burden of colonoscopy after positive fecal immunochemical testing for colorectal cancer screening. Endoscopy. 2013;45(5):342-9. Epub 2013/03/14. doi: 10.1055/s-0032-1326238. PubMed PMID: 23483433.
46. Derbyshire E, Hungin P, Nickerson C, Rutter MD. Colonoscopic perforations in the English National Health Service Bowel Cancer Screening Programme. Endoscopy. 2018;50(9):861-70. Epub 2018/03/29. doi: 10.1055/a-0584-7138

10.1055/a-0584-7138. Epub 2018 Mar 28.
47. Din S, Ball AJ, Taylor E, Rutter M, Riley SA, Johal S. Polypectomy practices of sub-centimeter polyps in the English Bowel Cancer Screening Programme. Surg Endosc. 2015;29(11):3224-30. Epub 2015/01/17. doi: 10.1007/s00464-015-4064-6. PubMed PMID: 25591413.
48. S DIN, Kerr K, Goddard A. PWE-008 The East Midlands polypectomy study. Gut. 2010;59(Suppl 1):A88. doi: 10.1136/gut.2009.208942d.
49. Dominitz J RD, Ahnen DJ, Beed A, Boardman K, Del Curto B, Guarino P, Imperiale T, Johnson G, Kyriakides T,LaCasse A, Larson M, Lieberman D, Provenzale D, Shaukat A, Sultan S, Planeta B. Screening colonoscopy findings and complications in a multi-center study in the United States. United European Gastroenterology Journal. 2019;7(8):258---. doi: 10.1177/205064061985467.
50. Dyson JK, Mason JM, Rutter MD. Prior hysterectomy and discomfort during colonoscopy: a retrospective cohort analysis. Endoscopy. 2014;46(6):493-8. Epub 2014/05/03. doi: 10.1055/s-0034-1365462. PubMed PMID: 24788540.
51. Ellul P, Fogden E, Simpson CL, Nickerson CL, McKaig BC, Swarbrick ET, et al. Downstaging of colorectal cancer by the National Bowel Cancer Screening programme in England: first round data from the first centre. Colorectal disease : the official journal of the Association of Coloproctology of Great Britain and Ireland. 2010;12(5):420-2. Epub 2009/10/22. doi: 10.1111/j.1463-1318.2009.02069.x. PubMed PMID: 19843116.
52. Faivre J, Dancourt V, Lejeune C, Tazi MA, Lamour J, Gerard D, et al. Reduction in colorectal cancer mortality by fecal occult blood screening in a French controlled study. Gastroenterology. 2004;126(7):1674-80. Epub 2004/06/10. doi: 10.1053/j.gastro.2004.02.018. PubMed PMID: 15188160.
53. Florido M, Ortega SP, Iyo EY, Reyes J, Novella M, Lucero JA, et al. Result of the first round of the colorectal cancer screening programme in the balearic islands (Spain). United European Gastroenterology Journal. 2017;5(5 Supplement 1):A558-A. doi: http://dx.doi.org/10.1177/2050640617725676.
54. Fritzell K, Forsberg A, Wangmar J, Wengström Y, Bottai M, Hultcrantz R. Gender, having a positive FIT and type of hospital are important factors for colonoscopy experience in colorectal cancer screening - findings from the SCREESCO study. Scand J Gastroenterol. 2020;55(11):1354-62. Epub 2020/09/19. doi: 10.1080/00365521.2020.1820568

10.1080/00365521.2020.1820568. Epub 2020 Sep 18.
55. Garcia M, Milà N, Binefa G, Borràs JM, Espinàs JA, Moreno V. False-positive results from colorectal cancer screening in Catalonia (Spain), 2000-2010. J Med Screen. 2012;19(2):77-82. Epub 2012/06/02. doi: 10.1258/jms.2012.012013. PubMed PMID: 22653571.
56. Ghanouni A, Plumb A, Hewitson P, Nickerson C, Rees CJ, von Wagner C. Patients' experience of colonoscopy in the English Bowel Cancer Screening Programme. Endoscopy. 2016;48(3):232-40. Epub 2016/02/04. doi: 10.1055/s-0042-100613. PubMed PMID: 26841268.
57. Graser A, Stieber P, Nagel D, Schäfer C, Horst D, Becker CR, et al. Comparison of CT colonography, colonoscopy, sigmoidoscopy and faecal occult blood tests for the detection of advanced

adenoma in an average risk population. Gut. 2009;58(2):241-8. Epub 2008/10/15. doi: 10.1136/gut.2008.156448. PubMed PMID: 18852257.
58. Gupta S, Saunders BP, Fraser C, Kennedy RH, Ignjatovic A, Sala S, et al. The first 3 years of national bowel cancer screening at a single UK tertiary centre. Colorectal disease : the official journal of the Association of Coloproctology of Great Britain and Ireland. 2012;14(2):166-73. Epub 2011/06/22. doi: 10.1111/j.1463-1318.2011.02567.x. PubMed PMID: 21689280.
59. Hsu WF, Chang CY, Chang CC, Chang LC, Chen CH, Lin CC, et al. Risk of colonoscopy-related complications in a fecal immunochemical test-based population colorectal cancer screening program. Endoscopy. 2020. Epub 2020/12/04. doi: 10.1055/a-1328-5126

10.1055/a-1328-5126.
60. Hughes K, Leggett B, Del Mar C, Croese J, Fairley S, Masson J, et al. Guaiac versus immunochemical tests: faecal occult blood test screening for colorectal cancer in a rural community. Australian & New Zealand Journal of Public Health. 2005;29(4):358-64. PubMed PMID: 106534378. Language: English. Entry Date: 20051104. Revision Date: 20150711. Publication Type: Journal Article.
61. Ibáñez J, Vanaclocha-Espí M, Pérez-Sanz E, Valverde MJ, Sáez-Lloret I, Molina-Barceló A, et al. Severe complications in colorectal cancer screening colonoscopies in the Valencian Community. Gastroenterol Hepatol. 2018;41(9):553-61. Epub 2018/07/30. doi: 10.1016/j.gastrohep.2018.06.007

10.1016/j.gastrohep.2018.06.007. Epub 2018 Jul 25.
62. Karlijn J. Nass; Peter J. van der Schaar; Manon van der Vlugt; Michiel LAAJvESvdBMML. Continuous monitoring of colonoscopy performance in

the Netherlands: first results of a nationwide registry. Endoscopy. 2021.
63. Kooyker AI, Toes-Zoutendijk E, Opstal-van Winden AWJ, Buskermolen M, van Vuuren HJ, Kuipers EJ, et al. Colonoscopy-Related Mortality in a Fecal Immunochemical Test-Based Colorectal Cancer Screening Program. Clin Gastroenterol Hepatol. 2021;19(7):1418-25. Epub 2020/08/11. doi: 10.1016/j.cgh.2020.07.066

10.1016/j.cgh.2020.07.066. Epub 2020 Aug 7.
64. Lee TJ, Rutter MD, Blanks RG, Moss SM, Goddard AF, Chilton A, et al. Colonoscopy quality measures: experience from the NHS Bowel Cancer Screening Programme. Gut. 2012;61(7):1050-7. Epub 2011/09/24. doi: 10.1136/gutjnl-2011-300651. PubMed PMID: 21940723.
65. Marino M, Zucchi E, Cimarosti R, Berretti D, Bulajic M, Lodolo I, et al. Adverse events during bowel cancer screening program in single tertiary centre in north-east Italy: A28 months preliminary report. Digestive and Liver Disease. 2012;44:S201.
66. Meulen LWT, van der Zander QEW, Bogie RMM, Keulen ETP, van Nunen AB, Winkens B, et al. Evaluation of polypectomy quality indicators of large nonpedunculated colorectal polyps in a nonexpert, bowel cancer screening cohort. Gastrointestinal endoscopy. 2021;94(6):1085-95.e2. Epub 2021/06/18. doi: 10.1016/j.gie.2021.06.008

10.1016/j.gie.2021.06.008. Epub 2021 Jun 15.
67. Mikkelsen EM, Thomsen MK, Tybjerg J, Friis-Hansen L, Andersen B, Jorgensen JCR, et al. Colonoscopy-related complications in a nationwide immunochemical fecal occult blood test-based colorectal cancer screening program. Clin Epidemiol. 2018;10:1649-55. PubMed PMID: 30519113.
68. Monteiro H, Tavares F, Reis J, Ferreira G, Campos MJ, Costa S, et al. Colorectal Screening Program in Northern Portugal: First Findings. Acta Med Port. 2021. Epub 2021/07/21. doi: 10.20344/amp.15904

10.20344/amp.15904.
69. Neely D, Campbell W, Davey P, Rodgers C, McCrory D. Colorectal cancer screening: The northern trust experience. Ulster Medical Journal. 2013;82(3):160-3.

70. Parente F, Boemo C, Ardizzoia A, Costa M, Carzaniga P, Ilardo A, et al. Outcomes and cost evaluation of the first two rounds of a colorectal cancer screening program based on immunochemical fecal occult blood test in northern Italy. Endoscopy. 2013;45(1):27-34. Epub 2012/12/21. doi: 10.1055/s-0032-1325800. PubMed PMID: 23254404.
71. Paszat LF, Sutradhar R, Luo J, Rabeneck L, Tinmouth J, Luo J. Perforation and post-polypectomy bleeding complicating colonoscopy in a population-based screening program. Endoscopy International Open. 2021;9(4):E637-e45. Epub 2021/04/22. doi: http://dx.doi.org/10.1055/a-1381-7149

10.1055/a-1381-7149. Epub 2021 Apr 15.
72. Portillo I, Idigoras I, Bilbao I, Arana-Arri E, Fernández-Landa MJ, Hurtado JL, et al. Colorectal cancer screening program using FIT: quality of colonoscopy varies according to hospital type. Endosc Int Open. 2018;6(9):E1149-e56. Epub 2018/09/14. doi: 10.1055/a-0655-1987

10.1055/a-0655-1987. Epub 2018 Sep 11.
73. Quintero E, Castells A, Bujanda L, Cubiella J, Salas D, Lanas Á, et al. Colonoscopy versus fecal immunochemical testing in colorectal-cancer screening. N Engl J Med. 2012;366(8):697-706. Epub 2012/02/24. doi: 10.1056/NEJMoa1108895. PubMed PMID: 22356323.
74. Quyn AJ, Fraser CG, Stanners G, Carey FA, Rees CJ, Moores B, et al. Scottish Bowel Screening Programme colonoscopy quality - scope for improvement? Colorectal Dis. 2018;20(9):O277-O83. Epub 2018/06/05. doi: 10.1111/codi.14281. PubMed PMID: 29863812.
75. Robertson DJ, Dominitz JA, Beed A, Ahnen D, Boardman KD, Curto BD, et al. COMPLICATIONS OF SCREENING COLONOSCOPY IN A MULTI-CENTER STUDY OF COLORECTAL CANCER SCREENING. Gastroenterology. 2019;156(6 Supplement 1):S-150. doi: http://dx.doi.org/10.1016/S0016-5085%2819%2937164-1.
76. Robinson MH, Hardcastle JD, Moss SM, Amar SS, Chamberlain JO, Armitage NC, et al. The risks of screening: data from the Nottingham randomised controlled trial of faecal occult blood screening for colorectal cancer. Gut. 1999;45(4):588-92. Epub 1999/09/16. PubMed PMID: 10486370; PubMed Central PMCID: PMCPMC1727686.
77. Rutter MD, Nickerson C, Rees CJ, Patnick J, Blanks RG. Risk factors for adverse events related to polypectomy in the English Bowel Cancer Screening Programme. Endoscopy. 2014;46(2):90-7. Epub 2014/01/31. doi: 10.1055/s-0033-1344987. PubMed PMID: 24477363.
78. Saraste D, Martling A, Nilsson PJ, Blom J, Tornberg S, Hultcrantz R, et al. Complications after colonoscopy and surgery in a population-based colorectal cancer screening programme. J Med Screen. 2016;23:135-40. Epub 2016/03/05. doi: 10.1177/0969141315625701. PubMed PMID: 26940962.
79. Steele R, Group UCCSP. Results of the first round of a demonstration pilot of screening for colorectal cancer in the United Kingdom. BMJ. 2004;329(7458):133. PubMed PMID: 15237087.
80. Steele RJ, McClements PL, Libby G, Black R, Morton C, Birrell J, et al. Results from the first three rounds of the Scottish demonstration pilot of FOBT screening for colorectal cancer. Gut. 2009;58(4):530-5. Epub 2008/11/28. doi: 10.1136/gut.2008.162883. PubMed PMID: 19036949.
81. Sung JJ, Chan FK, Leung WK, Wu JC, Lau JY, Ching J, et al. Screening for colorectal cancer in Chinese: comparison of fecal occult blood test, flexible sigmoidoscopy, and colonoscopy. Gastroenterology. 2003;124(3):608-14. Epub 2003/03/04. doi: 10.1053/gast.2003.50090. PubMed PMID: 12612899.
82. Tepeš B, Bracko M, Novak Mlakar D, Stefanovic M, Stabuc B, Frkovic Grazio S, et al. Results of the FIT-based National Colorectal Cancer Screening Program in Slovenia. Journal of clinical gastroenterology. 2017;51(6):e52-e9. Epub 2016/08/24. doi: 10.1097/mcg.0000000000000662. PubMed PMID: 27552327.
83. Tomaszewski M, Sanders D, Enns R, Gentile L, Cowie S, Nash C, et al. Risks associated with colonoscopy in a population-based colon screening program: an observational cohort study. CMAJ Open. 2021;9(4):E940-e7. Epub 2021/10/14. doi: 10.9778/cmajo.20200192

10.9778/cmajo.20200192. Print 2021 Oct-Dec.

84. Vanaclocha-Espi M, Molina-Barcelo A, Ibanez J, Valverde-Roig MJ, Perez E, Salas D, et al. Risk factors for severe complications of colonoscopy in screening programs. Preventive Medicine. 2019;118:304-8. doi: http://dx.doi.org/10.1016/j.ypmed.2018.11.010.
85. Yamada E, Watanabe S, Nakajima A. Associations of Mental Health and Physical Function with Colonoscopy-related Pain. Internal medicine (Tokyo, Japan). 2017;56(4):383-8. Epub 2017/02/17. doi: 10.2169/internalmedicine.56.7465. PubMed PMID: 28202858; PubMed Central PMCID: PMCPMC5364189.
86. Zorzi M, Fedato C, Naldoni C, Sassatelli R, Sassoli De' Bianchi P, Senore C, et al. Screening for colorectal cancer in Italy: 2007 survey. Epidemiologia e prevenzione. 2009;33(3 Suppl 2):57-74. Epub 2009/12/04. PubMed PMID: 19776487.
87. Castro G, Azrak MF, Seeff LC, Royalty J. Outpatient colonoscopy complications in the CDC's Colorectal Cancer Screening Demonstration Program: a prospective analysis. Cancer. 2013;119 Suppl 15:2849-54. Epub 2013/07/24. doi: 10.1002/cncr.28159. PubMed PMID: 23868479.
88. Dellon ES, Lippmann QK, Galanko JA, Sandler RS, Shaheen NJ. Effect of GI endoscopy nurse experience on screening colonoscopy outcomes. Gastrointestinal endoscopy. 2009;70(2):331-43. Epub 2009/06/09. doi: 10.1016/j.gie.2008.12.059. PubMed PMID: 19500788; PubMed Central PMCID: PMCPMC2753217.
89. Ko CW, Riffle S, Michaels L, Morris C, Holub J, Shapiro JA, et al. Serious complications within 30 days of screening and surveillance colonoscopy are uncommon. Clinical gastroenterology and hepatology : the official clinical practice journal of the American Gastroenterological Association. 2010;8(2):166-73. Epub 2009/10/24. doi: 10.1016/j.cgh.2009.10.007. PubMed PMID: 19850154; PubMed Central PMCID: PMCPMC2821994.
90. Mandel JS, Bond JH, Church TR, Snover DC, Bradley GM, Schuman LM, et al. Reducing mortality from colorectal cancer by screening for fecal occult blood. Minnesota Colon Cancer Control Study. N Engl J Med. 1993;328(19):1365-71. Epub 1993/05/13. doi: 10.1056/nejm199305133281901. PubMed PMID: 8474513.
91. Naumann DN, Kavanagh C, Hipkiss G, Potter-Concannon S, Budhoo M, Ahmed M, et al. Impact of cumulative experience on the quality of screening colonoscopy: A 13-year observational study. J Med Screen. 2021;28(4):433-8. Epub 2021/04/20. doi: 10.1177/09691413211009562

10.1177/09691413211009562. Epub 2021 Apr 17.
92. Polter DE. Risk of colon perforation during colonoscopy at Baylor University Medical Center. Proceedings (Baylor University Medical Center). 2015;28(1):3-6. Epub 2015/01/02. PubMed PMID: 25552784; PubMed Central PMCID: PMCPMC4264696.
93. Rutter CM, Johnson E, Miglioretti DL, Mandelson MT, Inadomi J, Buist DS. Adverse events after screening and follow-up colonoscopy. Cancer causes & control : CCC. 2012;23(2):289-96. Epub 2011/11/23. doi: 10.1007/s10552-011-9878-5. PubMed PMID: 22105578; PubMed Central PMCID: PMCPMC3406732.
94. Shroff S, Abdeljawad K, Qayed E. Incidence of major complications during outpatient colonoscopy for colon cancer screening. American Journal of Gastroenterology. 2015;110:S617.
95. Berhane C, Denning D. Incidental finding of colorectal cancer in screening colonoscopy and its cost effectiveness. The American surgeon. 2009;75(8):699-703; discussion -4. Epub 2009/09/04. PubMed PMID: 19725293.
96. Bielawska B, Day AG, Lieberman DA, Hookey LC. Risk factors for early colonoscopic perforation include non-gastroenterologist endoscopists: a multivariable analysis. Clinical gastroenterology and hepatology : the official clinical practice journal of the American Gastroenterological Association. 2014;12(1):85-92. Epub 2013/07/31. doi: 10.1016/j.cgh.2013.06.030. PubMed PMID: 23891916; PubMed Central PMCID: PMCPMC4050305.
97. Bokemeyer B, Bock H, Hüppe D, Düffelmeyer M, Rambow A, Tacke W, et al. Screening colonoscopy for colorectal cancer prevention: results from a German online registry on 269000 cases.

European journal of gastroenterology & hepatology. 2009;21(6):650-5. PubMed PMID: 105533266. Language: English. Entry Date: 20090619. Revision Date: 20150711. Publication Type: Journal Article.
98. Bretthauer M, Kaminski MF, Løberg M, Zauber AG, Regula J, Kuipers EJ, et al. Population-Based Colonoscopy Screening for Colorectal Cancer: A Randomized Clinical Trial. JAMA Intern Med. 2016;176(7):894-902. PubMed PMID: 27214731.
99. Bugajski M, Wieszczy P, Hoff G, Rupinski M, Regula J, Kaminski MF. Modifiable factors associated with patient-reported pain during and after screening colonoscopy. Gut. 2018;67(11):1958-64. Epub 2017/10/04. doi: 10.1136/gutjnl-2017-313905

10.1136/gutjnl-2017-313905. Epub 2017 Sep 28.
100. Causada-Calo N, Bishay K, Al Mazroui A, Albashir S, Armstrong D. Association between Age and Complications after Outpatient Colonoscopy. JAMA Network Open. 2020;3(6):8958-. doi: https://dx.doi.org/10.1001/jamanetworkopen.2020.8958.
101. Chiu H, Lee Y, Tu C, Chen C, Tseng P, Liang J, et al. Association between early stage colon neoplasms and false-negative results from the fecal immunochemical test. Clinical Gastroenterology and Hepatology. 2013;11(7):832-8.
102. Crispin A, Birkner B, Munte A, Nusko G, Mansmann U. Process quality and incidence of acute complications in a series of more than 230,000 outpatient colonoscopies. Endoscopy. 2009;41(12):1018-25. Epub 2009/10/27. doi: 10.1055/s-0029-1215214. PubMed PMID: 19856246.
103. Dae HK, Seok YL, Kui SC, Ho JL, Su CP, Kim J, et al. The usefulness of colonoscopy as a screening test for detecting colorectal polyps. Hepato-gastroenterology. 2007;54(80):2240-2.
104. Ferlitsch M, Reinhart K, Pramhas S, Wiener C, Gal O, Bannert C, et al. Sex-specific prevalence of adenomas, advanced adenomas, and colorectal cancer in individuals undergoing screening colonoscopy. JAMA. 2011;306(12):1352-8. Epub 2011/09/29. doi: 10.1001/jama.2011.1362. PubMed PMID: 21954479.
105. Garcia-Albeniz X, Hsu J, Bretthauer M, Hernan MA, García-Albéniz X, Hsu J, et al. Effectiveness of Screening Colonoscopy to Prevent Colorectal Cancer Among Medicare Beneficiaries Aged 70 to 79 Years: A Prospective Observational Study. Ann Intern Med. 2017;166:18-26. Epub 2016/09/27. doi: 10.7326/M16-0758. PubMed PMID: 27669524.
106. Hamdani U, Naeem R, Haider F, Bansal P, Komar M, Diehl DL, et al. Risk factors for colonoscopic perforation: a population-based study of 80118 cases. World journal of gastroenterology. 2013;19(23):3596-601. Epub 2013/06/27. doi: 10.3748/wjg.v19.i23.3596. PubMed PMID: 23801860; PubMed Central PMCID: PMCPMC3691036.
107. Huppe D, Lemberg L, Felten G. Effectiveness and patient tolerance of screening colonoscopy - First results. Zeitschrift fur Gastroenterologie. 2004;42(7):591-8.
108. Imperiale TF, Wagner DR, Lin CY, Larkin GN, Rogge JD, Ransohoff DF. Risk of Advanced Proximal Neoplasms in Asymptomatic Adults According to the Distal Colorectal Findings. New England Journal of Medicine. 2000;343(3):169-74. doi: 10.1056/NEJM200007203430302.
109. Ionescu EM, Nicolaie T, Gologan SI, Mocanu A, Ditescu C, Arbanas T, et al. Opportunistic colorectal cancer screening using colonoscopy. Comparative results between two historical cohorts in Bucharest, Romania. Journal of Gastrointestinal and Liver Diseases. 2015;24(2):171-6.
110. Kaminski MF, Wieszczy P, Rupinski M, Wojciechowska U, Didkowska J, Kraszewska E, et al. Increased Rate of Adenoma Detection Associates With Reduced Risk of Colorectal Cancer and Death. Gastroenterology. 2017;153(1):98-105. doi: https://doi.org/10.1053/j.gastro.2017.04.006.
111. Khalid-de Bakker CA, Jonkers DM, Hameeteman W, de Ridder RJ, Masclee AA, Stockbrugger RW. Cardiopulmonary events during primary colonoscopy screening in an average risk population. The Netherlands journal of medicine. 2011;69(4):186-91. Epub 2011/04/30. PubMed PMID: 21527807.
112. Khalid-de Bakker CA, Jonkers DM, Hameeteman W, de Ridder RJ, Masclee AA, Stockbrugger RW. Opportunistic screening of hospital staff using primary colonoscopy: participation, discomfort and willingness to repeat the procedure. Digestion. 2011;84(4):281-8. Epub 2011/11/02. doi: 10.1159/000327383. PubMed PMID: 22041853.

113. Kobiela J, Spychalski P, Wieszczy P, Pisera M, Pilonis N, Rupinski M, et al. Mortality and Rate of Hospitalization in a Colonoscopy Screening Program From a Randomized Health Services Study. Clin Gastroenterol Hepatol. 2019;18(7):1501-8.e3. Epub 2019/09/17. doi: 10.1016/j.cgh.2019.09.010

10.1016/j.cgh.2019.09.010. Epub 2019 Sep 13.
114. Kozbial K, Reinhart K, Heinze G, Zwatz C, Bannert C, Salzl P, et al. High quality of screening colonoscopy in Austria is not dependent on endoscopist specialty or setting. Endoscopy. 2015;47(3):207-16. Epub 2014/11/21. doi: 10.1055/s-0034-1390910. PubMed PMID: 25412094.
115. Levent EA, Genthner, F Straulino, S Kangalli, I Reiffenstein, N Tscherwinski, A Eickhoff. Postpolypectomy Syndrome - an Underrated Complication After Endoscopic Polypectomy?Which Risk Factors Lead to a Postpolypectomy Syndrome? Endoscopy. 2021.
116. Lieberman DA, Weiss DG, Bond JH, Ahnen DJ, Garewal H, Harford WV, et al. Use of Colonoscopy to Screen Asymptomatic Adults for Colorectal Cancer. New England Journal of Medicine. 2000;343(3):162-8. doi: 10.1056/NEJM200007203430301.
117. Nelson DB, McQuaid KR, Bond JH, Lieberman DA, Weiss DG, Johnston TK, et al. Procedural success and complications of large-scale screening colonoscopy. Gastrointestinal endoscopy. 2002;55:307-14.
118. Pedersen L, Sorensen N, Bernstein I, Lindorff-Larsen K, Carlsen CG, Wensel N, et al. Colonoscopy adverse events: are we getting the full picture? Scandinavian Journal of Gastroenterology. 2020:1-9. doi: http://dx.doi.org/10.1080/00365521.2020.1792541.
119. Pox CP, Altenhofen L, Brenner H, Theilmeier A, Von Stillfried D, Schmiegel W. Efficacy of a nationwide screening colonoscopy program for colorectal cancer. Gastroenterology. 2012;142(7):1460-7 e2. Epub 2012/03/27. doi: 10.1053/j.gastro.2012.03.022. PubMed PMID: 22446606.
120. Regula J, Rupinski M, Kraszewska E, Polkowski M, Pachlewski J, Orlowska J, et al. Colonoscopy in colorectal-cancer screening for detection of advanced neoplasia. The New England journal of medicine. 2006;355(18):1863-72. Epub 2006/11/03. doi: 10.1056/NEJMoa054967. PubMed PMID: 17079760.
121. Schoenfeld P, Cash B, Flood A, Dobhan R, Eastone J, Coyle W, et al. Colonoscopic screening of average-risk women for colorectal neoplasia. The New England journal of medicine. 2005;352(20):2061-8. Epub 2005/05/20. doi: 10.1056/NEJMoa042990. PubMed PMID: 15901859.
122. Sieg A, Theilmeier A. [Results of coloscopy screening in 2005--an Internet-based documentation]. Dtsch Med Wochenschr. 2006;131(8):379-83. Epub 2006/02/16. doi: 10.1055/s-2006-932528. PubMed PMID: 16479468.
123. Stock C, Ihle P, Sieg A, Schubert I, Hoffmeister M, Brenner H. Adverse events requiring hospitalization within 30 days after outpatient screening and nonscreening colonoscopies. Gastrointestinal endoscopy. 2013;77(3):419-29. Epub 2013/02/16. doi: 10.1016/j.gie.2012.10.028. PubMed PMID: 23410698.
124. Stoop EM, de Haan MC, de Wijkerslooth TR, Bossuyt PM, van Ballegooijen M, Nio CY, et al. Participation and yield of colonoscopy versus non-cathartic CT colonography in population-based screening for colorectal cancer: a randomised controlled trial. The Lancet Oncology. 2012;13(1):55-64. Epub 2011/11/18. doi: 10.1016/s1470-2045(11)70283-2. PubMed PMID: 22088831.
125. Strul H, Kariv R, Leshno M, Halak A, Jakubowicz M, Santo M, et al. The prevalence rate and anatomic location of colorectal adenoma and cancer detected by colonoscopy in average-risk individuals aged 40-80 years. The American journal of gastroenterology. 2006;101(2):255-62. Epub 2006/02/04. doi: 10.1111/j.1572-0241.2006.00430.x. PubMed PMID: 16454827.
126. Taleban S, Toosizadeh N, Junna S, Golden T, Ghazala S, Wadeea R, et al. Frailty assessment predicts acute outcomes in patients undergoing screening colonoscopy. Dig Dis Sci. 2018;63(12):3272-80. Epub 2018/05/26. doi: 10.1007/s10620-018-5129-x

10.1007/s10620-018-5129-x. Epub 2018 May 24.

127. Taupin D, Chambers SL, Corbett M, Shadbolt B. Colonoscopic screening for colorectal cancer improves quality of life measures: a population-based screening study. Health and quality of life outcomes. 2006;4:82. Epub 2006/10/19. doi: 10.1186/1477-7525-4-82. PubMed PMID: 17044941; PubMed Central PMCID: PMCPMC1626073.
128. van Dam L, de Wijkerslooth TR, de Haan MC, Stoop EM, Bossuyt PM, Fockens P, et al. Time requirements and health effects of participation in colorectal cancer screening with colonoscopy or computed tomography colonography in a randomized controlled trial. Endoscopy. 2013;45(3):182-8. Epub 2013/03/01. doi: 10.1055/s-0032-1326080. PubMed PMID: 23446667.
129. Waldmann E, Gessl I, Sallinger D, Jeschek P, Britto-Arias M, Heinze G, et al. Trends in quality of screening colonoscopy in Austria. Endoscopy. 2016;48(12):1102-9.
130. Wang L, Mannalithara A, Singh G, Ladabaum U. Low Rates of Gastrointestinal and Non-Gastrointestinal Complications for Screening or Surveillance Colonoscopies in a Population-Based Study. Gastroenterology. 2018;154(3):540-55.e8. Epub 2017/10/17. doi: 10.1053/j.gastro.2017.10.006

10.1053/j.gastro.2017.10.006. Epub 2017 Oct 12.
131. Wang P, Xu T, Ngamruengphong S, Makary MA, Kalloo A, Hutfless S. Rates of infection after colonoscopy and osophagogastroduodenoscopy in ambulatory surgery centres in the USA. Gut. 2018;67(9):1626-36. Epub 2018/05/20. doi: 10.1136/gutjnl-2017-315308

10.1136/gutjnl-2017-315308. Epub 2018 May 18.
132. Wijkerslooth TR, de Haan MC, Stoop EM, Bossuyt PM, Thomeer M, Essink-Bot ML, et al. Burden of colonoscopy compared to non-cathartic CT-colonography in a colorectal cancer screening programme: randomised controlled trial. Gut. 2012;61(11):1552-9. Epub 2011/12/27. doi: 10.1136/gutjnl-2011-301308. PubMed PMID: 22198714.
133. Wong RJ, Tran B, Le A, Ly M, Nguyen HA, Nguyen K, et al. Low rates of adverse events and low utilization of emergency department or outpatient clinics following screening and surveillance colonoscopy among average risk adults. Gastrointestinal endoscopy. 2017;85(5 Supplement 1):AB85-AB.
134. Xirasagar S, Wu Y, Tsai M-hH, Zhang J, Chiodini S, de Groen PC, et al. Colorectal cancer prevention by a CLEAR principles-based colonoscopy protocol: an observational study. Gastrointestinal endoscopy. 2020;91(4):905-. Epub 2019/12/10. doi: 10.1016/j.gie.2019.11.043

10.1016/j.gie.2019.11.043. Epub 2019 Dec 7. From Duplicate 3 (Colorectal cancer prevention by a CLEAR principles–based colonoscopy protocol: an observational study - Xirasagar, Sudha; Wu, Yuqi; Tsai, Meng-han; Zhang, Jiajia; Chiodini, Stephanie; de Groen, Piet C) research; tables/charts. Journal Subset: Biomedical; Double Blind Peer Reviewed; Editorial Board Reviewed; Expert Peer Reviewed; Peer Reviewed; USA. NLM UID: 0010505.
135. Zwink N, Holleczek B, Stegmaier C, Hoffmeister M, Brenner H, Holleczek B, et al. Complication Rates in Colonoscopy Screening for Cancer: A Prospective Cohort Study of Complications Arising During the Procedure and in the Ensuing Four Weeks. Deutsches Arzteblatt International. 2017;114(18):321-7. doi: http://dx.doi.org/10.3238/arztebl.2017.0321.
136. Adler A, Lieberman D, Aminalai A, Aschenbeck J, Drossel R, Mayr M, et al. Data quality of the German screening colonoscopy registry. Endoscopy. 2013;45(10):813-8. Epub 2013/09/11. doi: 10.1055/s-0033-1344583. PubMed PMID: 24019130.
137. Hol L, de Jonge V, van Leerdam ME, van Ballegooijen M, Looman CW, van Vuuren AJ, et al. Screening for colorectal cancer: comparison of perceived test burden of guaiac-based faecal occult blood test, faecal immunochemical test and flexible sigmoidoscopy. European journal of cancer (Oxford, England : 1990). 2010;46(11):2059-66. Epub 2010/07/14. doi: 10.1016/j.ejca.2010.03.022. PubMed PMID: 20621736.
138. Ladabaum U, Mannalithara A, Desai M, Sehgal M, Singh G. Age-Specific Rates and Time-Courses of Gastrointestinal and Nongastrointestinal Complications Associated With Screening/Surveillance Colonoscopy. The American journal of gastroenterology. 2021. Epub 2021/10/26. doi: 10.14309/ajg.0000000000001531.

139. Cotton PB, Eisen GM, Aabakken L, Baron TH, Hutter MM, Jacobson BC, et al. A lexicon for endoscopic adverse events: report of an ASGE workshop. Gastrointestinal endoscopy. 2010;71(3):446-54. doi: https://doi.org/10.1016/j.gie.2009.10.027.
