# Supplementary material for: Deaths and cardiopulmonary events following colorectal cancer screening—A systematic review with meta-analyses
Source: PLoS One. 2024 Mar 14;19(3):e0295900. doi: 10.1371/journal.pone.0295900 (PMC10939197; doi:10.1371/journal.pone.0295900)
Supplement: S2 File — (PDF) [file pone.0295900.s002.pdf]

S2 – Supplementary Figures

CONTENT

Fig. 3 Bias distribution for assessments of death 3

Fig. 4 Weighted bias distribution for assessments of death 4

Fig. 5 Bias distribution for assessments of CPEs 5

Fig. 6 Weighted bias distribution for assessments of CPEs 6

Fig. 7 Pooled estimate and forest plot of deaths with any follow up time associated with flexible sigmoidoscopy 7

Fig. 8 Pooled estimate and forest plot of deaths with reporting of follow up time associated with flexible sigmoidoscopy 8

Fig. 9 Pooled estimate and forest plot of deaths without reporting of follow up time associated with flexible sigmoidoscopy 9

Fig. 10 Pooled estimate and forest plot of deaths with any follow up time associated with colonoscopy following fobt 10

Fig. 11 Pooled estimate and forest plot of deaths with reporting of follow up time associated with colonoscopy following fobt 11

Fig. 12 Pooled estimate and forest plot of deaths without reporting of follow up time associated with colonoscopy following fobt 12

Fig. 13 Pooled estimate and forest plot of deaths with any follow up time associated with once-only colonoscopy 13

Fig. 14 Pooled estimate and forest plot of deaths with follow up time reported associated with once-only colonoscopy 14

Fig. 15 Pooled estimate and forest plot of deaths without reporting of follow up time associated with once-only colonoscopy 15

Fig. 16 Pooled estimate and forest plot of deaths with any follow up time associated with colonoscopy following various screening tests 16

Fig. 17 Pooled estimate and forest plot of deaths with reporting of follow up time associated with colonoscopy following various screening tests 17

Fig. 18 Pooled estimate and forest plot of vasovagal events with short-term follow up associated with flexible sigmoidoscopy 18

Fig. 19 Pooled estimate and forest plot of acute coronary syndrome with long-term follow up associated with flexible sigmoidoscopy 19

Fig. 20 Pooled estimate and forest plot of acute coronary syndrome with long-term follow up associated with colonoscopy following fobt 20

Fig. 21 Pooled estimate and forest plot of arrhythmia with long-term follow up associated with colonoscopy following fobt 21

Fig. 22 Pooled estimate and forest plot of thromboembolic events with long-term follow up associated with colonoscopy following fobt 22

Fig. 23 Pooled estimate and forest plot of acute coronary syndrome with short-term follow up associated with once-only colonoscopy 23

Fig. 24 Pooled estimate and forest plot of Pulmonary events with short-term follow up associated with once-only colonoscopy 24

Fig. 25 Pooled estimate and forest plot of Stroke events with short-term follow up associated with once-only colonoscopy 25

Fig. 26 Pooled estimate and forest plot of vasovagal events with short-term follow up associated with once-only colonoscopy 26

Fig. 27 Pooled estimate and forest plot of acute coronary syndrome with long-term follow up associated with once-only colonoscopy 27

Fig. 28 Pooled estimate and forest plot of arrhythmia with long-term follow up associated with once-only colonoscopy 28

Fig. 29 Pooled estimate and forest plot of heart failure with long-term follow up associated with once-only colonoscopy 29

Fig. 30 Pooled estimate and forest plot of pulmonary events with long-term follow up associated with once-only colonoscopy 30

Fig. 31 Pooled estimate and forest plot of stroke events with long-term follow up associated with once-only colonoscopy 31

Fig. 32 Pooled estimate and forest plot of thromboembolic events with long-term follow up associated with once-only colonoscopy 32

Fig. 33 Pooled estimate and forest plot of Vasovagal events with long-term follow up associated with once-only colonoscopy 33

FIG. 3 BIAS DISTRIBUTION FOR ASSESSMENTS OF DEATH

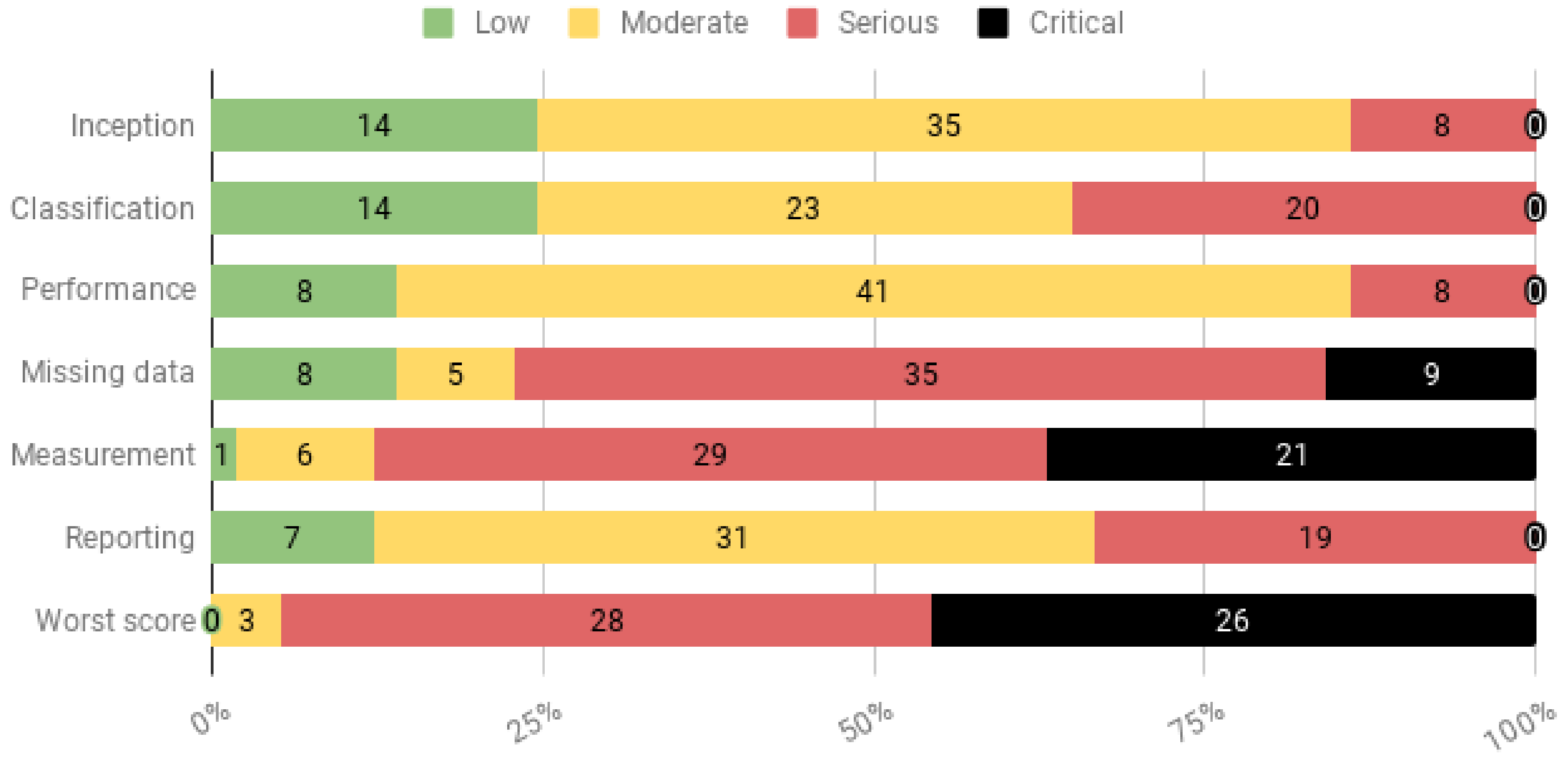

Figure 3. Green colour represents subpopulations with low risk of bias for the type of bias, yellow; moderate risk of bias, red; serious risk of bias and black; critical risk of bias.

FIG. 4 WEIGHTED BIAS DISTRIBUTION FOR ASSESSMENTS OF DEATH

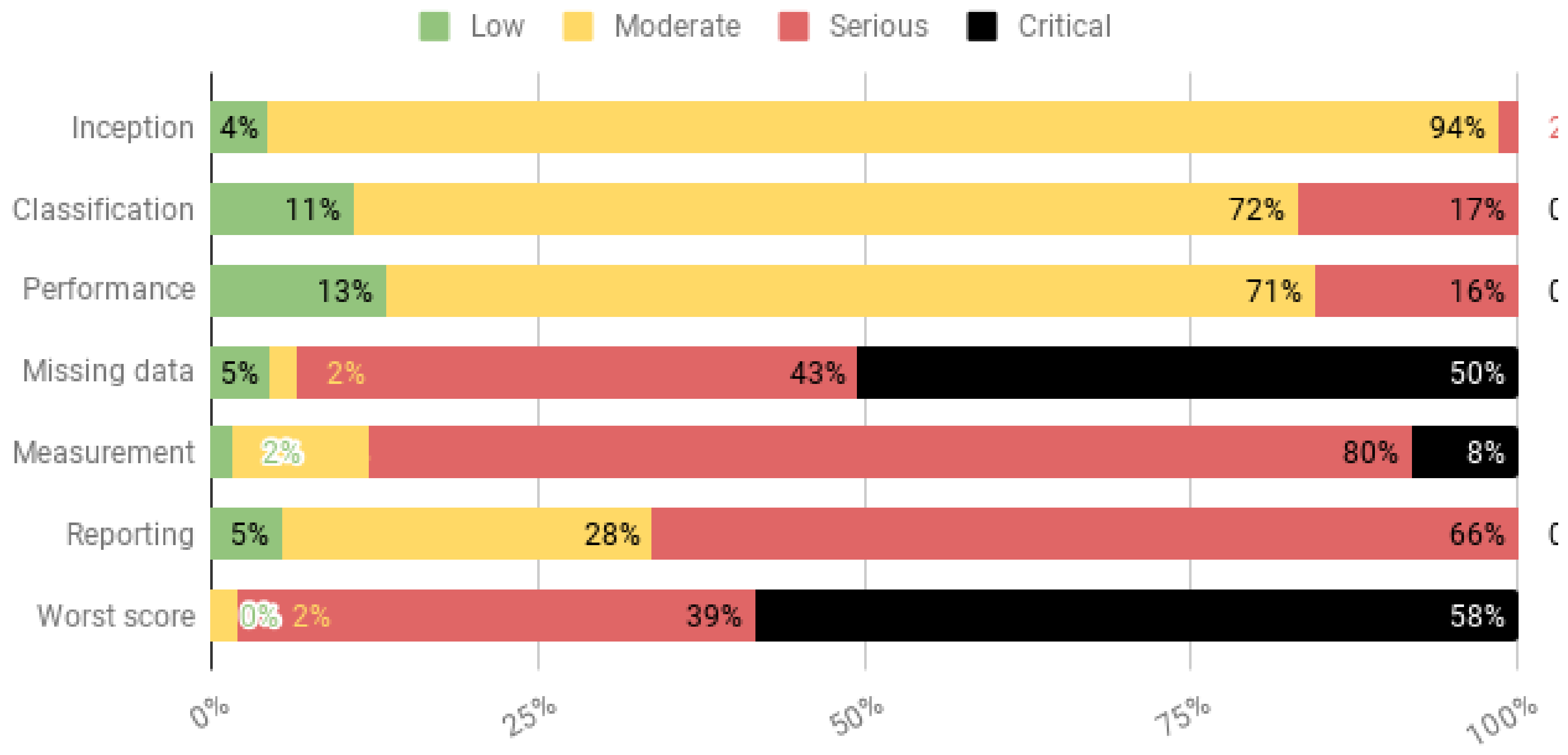

Figure 4. Green colour represents subpopulations with low risk of bias for the type of bias, yellow; moderate risk of bias, red; serious risk of bias and black; critical risk of bias. The distribution is weighted according to the size of each of the subpopulations.

FIG. 5 BIAS DISTRIBUTION FOR ASSESSMENTS OF CPES

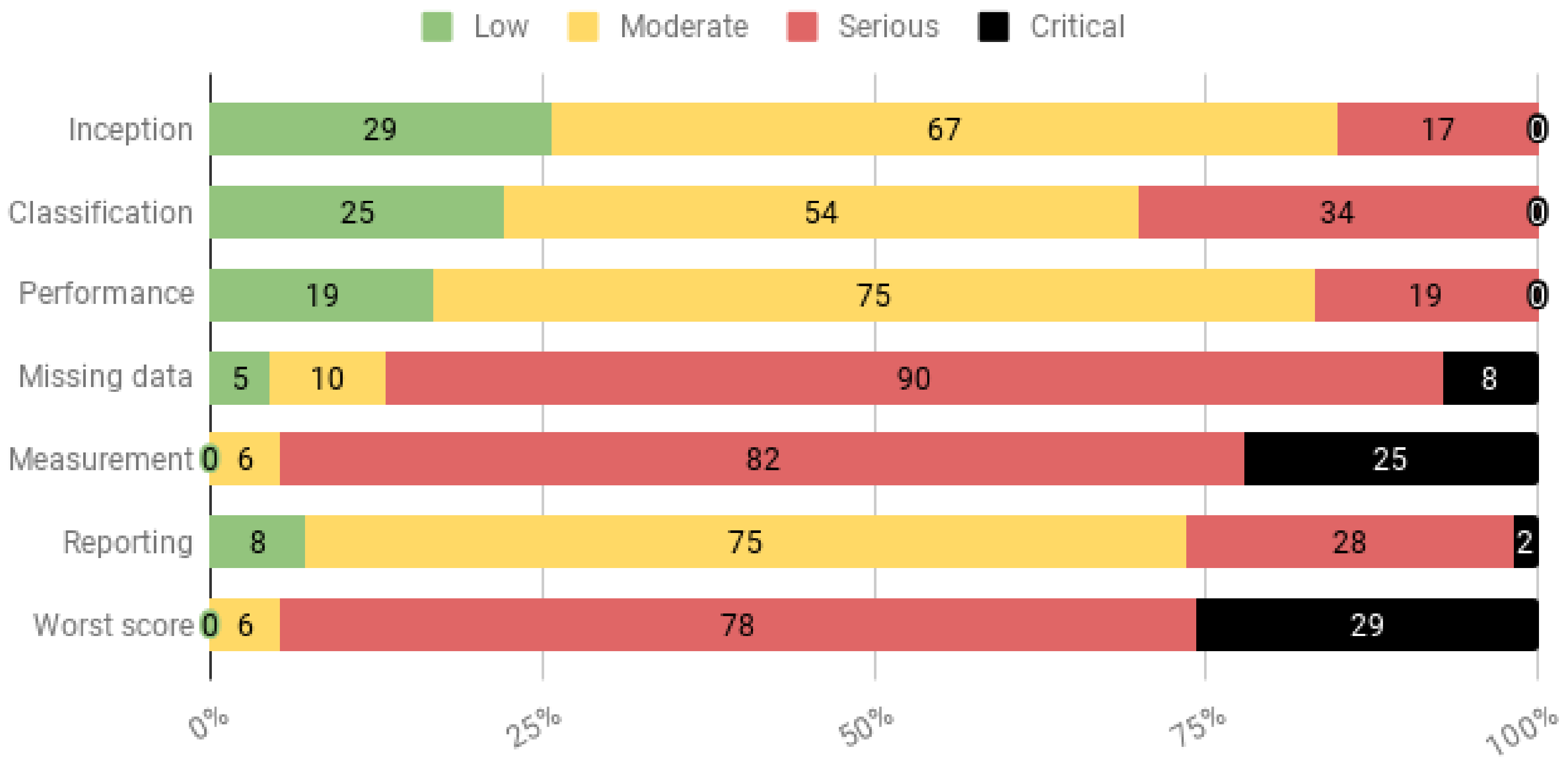

Figure 5. Green colour represents subpopulations with low risk of bias for the type of bias, yellow; moderate risk of bias, red; serious risk of bias and black; critical risk of bias. The distribution is weighted according to the size of each of the subpopulations.

FIG. 6 WEIGHTED BIAS DISTRIBUTION FOR ASSESSMENTS OF CPES

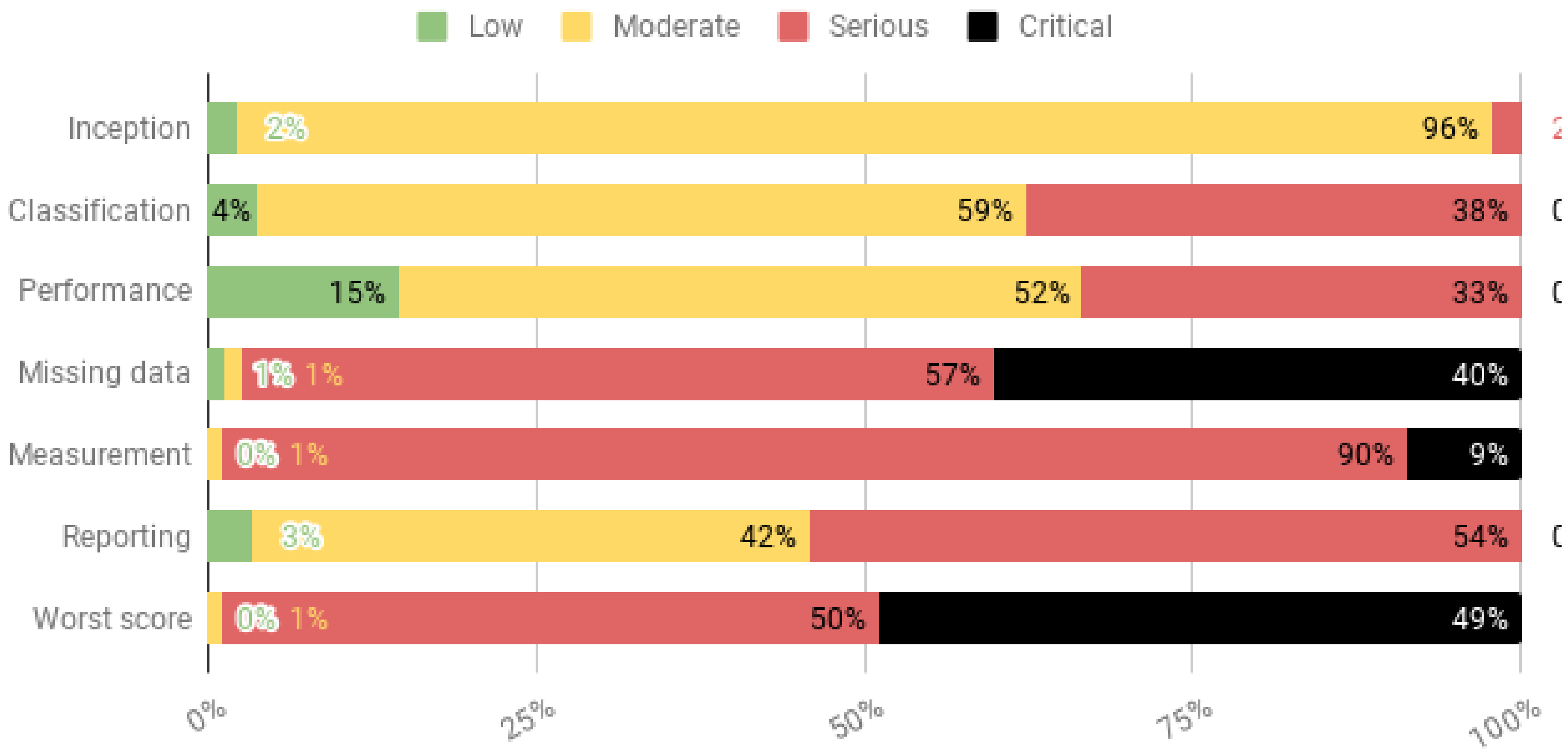

Figure 6. Green colour represents subpopulations with low risk of bias for the type of bias, yellow; moderate risk of bias, red; serious risk of bias and black; critical risk of bias. The distribution is weighted according to the size of each of the subpopulations.

FIG. 7 POOLED ESTIMATE AND FOREST PLOT OF DEATHS WITH ANY FOLLOW UP TIME ASSOCIATED WITH FLEXIBLE SIGMOIDOSCOPY

Study	Events	People	Weight	Point estimate per 100.000 people, 95% CI
Randel 2021	0	36065	8.93 %	0 [0 , 10]
Atkin 1998a	0	770	8.93 %	0 [0 , 478]
Holme 2014	0	12955	8.93 %	0 [0 , 28]
Jain 2002	0	4106	8.93 %	0 [0 , 90]
Atkin 1998b	1	536	13.67 %	187 [5 , 1035]
Atkin 2002	6	40674	24.48 %	15 [5 , 32]
Levin 2002	10	107704	26.13 %	9 [4 , 17]
1) Subtotal (not critical)	6	76739	33.41 %	3 [0 , 87]
2) Subtotal (critical)	11	126071	66.59 %	9 [5 , 16]
3) Total	17	202810	100 %	6 [1 , 45]

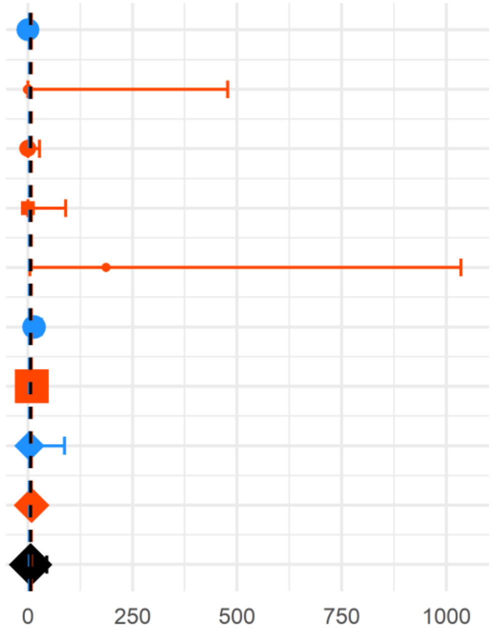

Heterogeneity:

1) $\tau^2 = 2.32$, $I^2 = 0$ %, $\chi^2 = 7.62$ (df = 1 , p-value = 0.0058)

2) $\tau^2 = 0$, $I^2 = 51.12$ %, $\chi^2 = 7.37$ (df = 4 , p-value = 0.1177)

3) $\tau^2 = 0.53$, $I^2 = 27.92$ %, $\chi^2 = 15.03$ (df = 6 , p-value = 0.02)

Figure 7. Blue-coloured object: Studies without critical risk of bias, Red-coloured object: Studies with critical risk of bias, Black-coloured object: All studies regardless of the risk of bias. Circles: Randomized Controlled Trials, Squares: Non-randomized studies, diamonds: Weighted summary measures. The blue diamond shows the weighted average for studies that do not have a critical risk of bias. The red diamond shows the weighted average for studies with critical risk of bias. The black diamond shows the weighted average for all studies regardless of their risk of bias.

FIG. 8 POOLED ESTIMATE AND FOREST PLOT OF DEATHS WITH REPORTING OF FOLLOW UP TIME ASSOCIATED WITH FLEXIBLE SIGMOIDOSCOPY

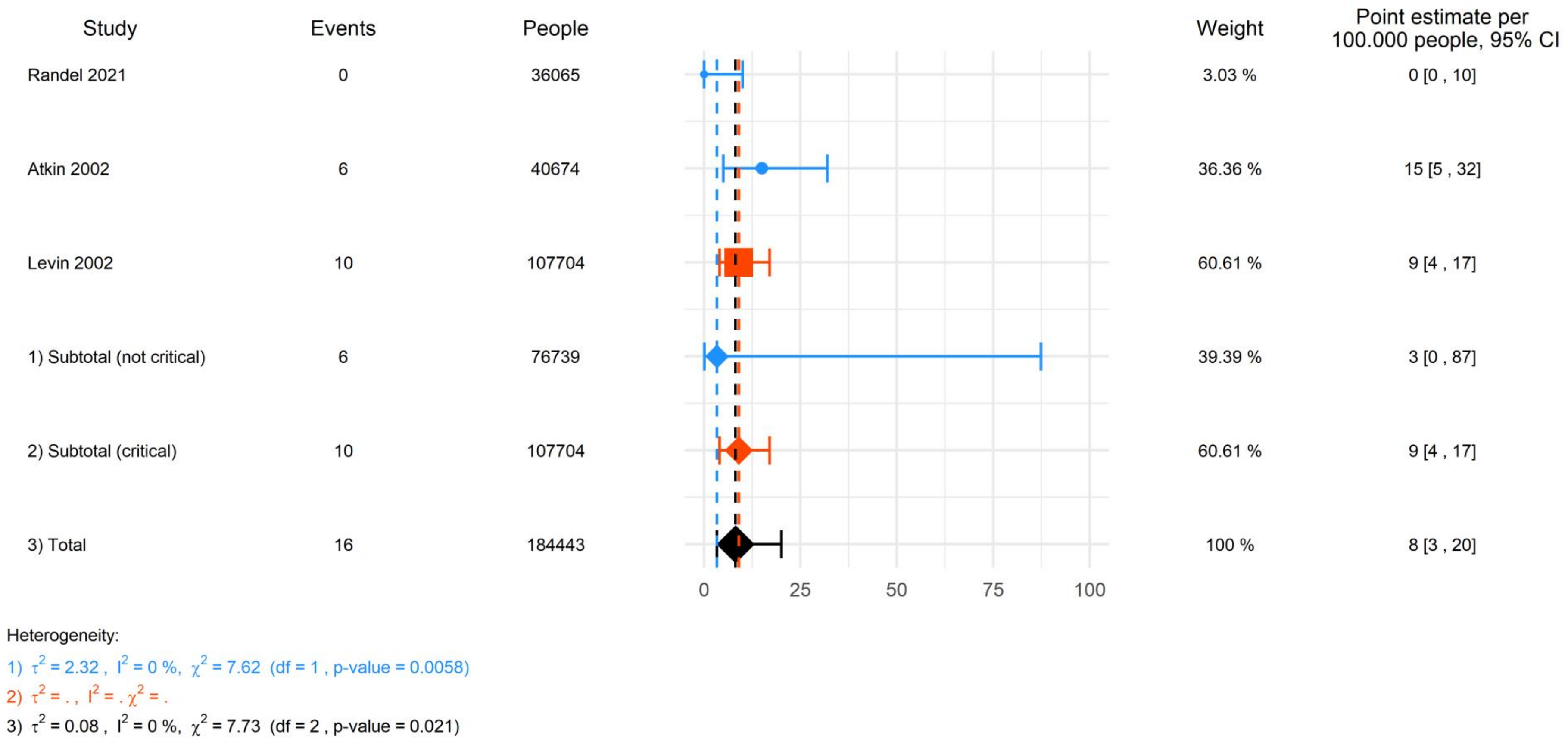

Figure 8. Blue-coloured object: Studies without critical risk of bias, Red-coloured object: Studies with critical risk of bias, Black-coloured object: All studies regardless of the risk of bias. Circles: Randomized Controlled Trials, Squares: Non-randomized studies, diamonds: Weighted summary measures. The blue diamond shows the weighted average for studies that do not have a critical risk of bias. The red diamond shows the weighted average for studies with critical risk of bias. The black diamond shows the weighted average for all studies regardless of their risk of bias.

FIG. 9 POOLED ESTIMATE AND FOREST PLOT OF DEATHS WITHOUT REPORTING OF FOLLOW UP TIME ASSOCIATED WITH FLEXIBLE SIGMOIDOSCOPY

Study	Events	People	Weight	Point estimate per 100.000 people, 95% CI
Holme 2014	0	12955	30.19 %	0 [0 , 28]
Jain 2002	0	4106	30.19 %	0 [0 , 90]
Atkin 1998a	0.01	770	1.4 %	1 [0 , 481]
Atkin 1998b	1	536	38.22 %	187 [5 , 1035]
1) Subtotal (critical)	1.01	18367	100 %	8 [1 , 81]
2) Total	1.01	18367	100 %	8 [1 , 81]

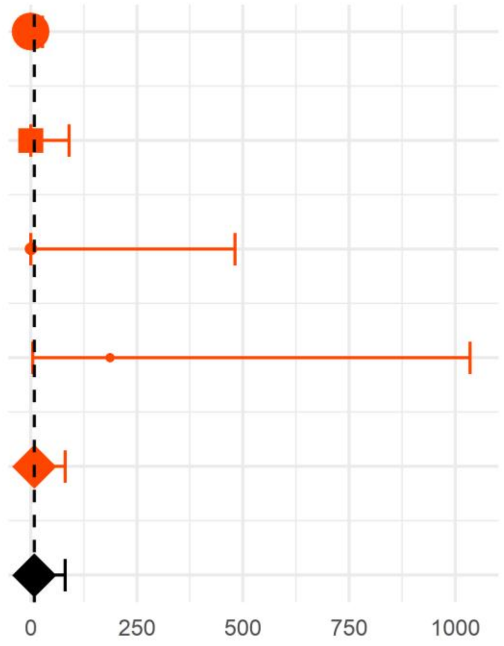

Heterogeneity:

1) $\tau^2 = 7.21$, $I^2 = 0$ %, χ^2 = NaN (df = 3 , p-value = NaN)

2) $\tau^2 = 7.21$, $I^2 = 0$ %, χ^2 = NaN (df = 3 , p-value = NaN)

Figure 9. Blue-coloured object: Studies without critical risk of bias, Red-coloured object: Studies with critical risk of bias, Black-coloured object: All studies regardless of the risk of bias. Circles: Randomized Controlled Trials, Squares: Non-randomized studies, diamonds: Weighted summary measures. The blue diamond shows the weighted average for studies that do not have a critical risk of bias. The red diamond shows the weighted average for studies with critical risk of bias. The black diamond shows the weighted average for all studies regardless of their risk of bias.

FIG. 10 POOLED ESTIMATE AND FOREST PLOT OF DEATHS WITH ANY FOLLOW UP TIME ASSOCIATED WITH COLONOSCOPY FOLLOWING FOBT

Study	Events	People	Weight	Point estimate per 100.000 people, 95% CI
Arana-Arri 2018	0	36346	3.35 %	0 [0 , 10]
Denis 2013	0	10024	3.35 %	0 [0 , 37]
Denis 2021b	0	9061	3.35 %	0 [0 , 41]
Ibanez 2018	0	7467	3.35 %	0 [0 , 49]
Marino 2012	0	1000	3.35 %	0 [0 , 368]
Robinson 1999	0	1300	3.35 %	0 [0 , 283]
Cheng 2002	0	6534	3.35 %	0 [0 , 56]
Din 2017	0	780	3.35 %	0 [0 , 472]
Lee 2012	0	32148	3.35 %	0 [0 , 11]
Steele 2009	0	8631	3.35 %	0 [0 , 43]
Denis 2021a	1	17152	4.71 %	6 [0 , 32]
Derbyshire 2018	1	222488	4.71 %	0 [0 , 3]
Portillo 2018	1	48759	4.71 %	2 [0 , 11]
Randel 2021	1	6945	4.71 %	14 [0 , 80]
Saraste 2016	1	2984	4.71 %	34 [1 , 187]
Vanaclocha-Espi 2018	1	41203	4.71 %	2 [0 , 14]
Quyn 2018	1	53332	4.71 %	2 [0 , 10]
Gupta 2012	1	1057	4.71 %	95 [2 , 526]
Tomaszewski 2021	2	78831	5.91 %	3 [0 , 9]
Mikkelsen 2018	11	14671	7.47 %	75 [37 , 134]
Benazzato 2020	15	99674	7.59 %	15 [8 , 25]
Kooyker 2021	48	158949	7.82 %	30 [22 , 40]
1) Subtotal (not critical)	83	752413	78.51 %	5 [2 , 13]
2) Subtotal (critical)	1	106923	21.46 %	1 [0 , 7]
3) Total	84	859336	100 %	3 [1 , 9]

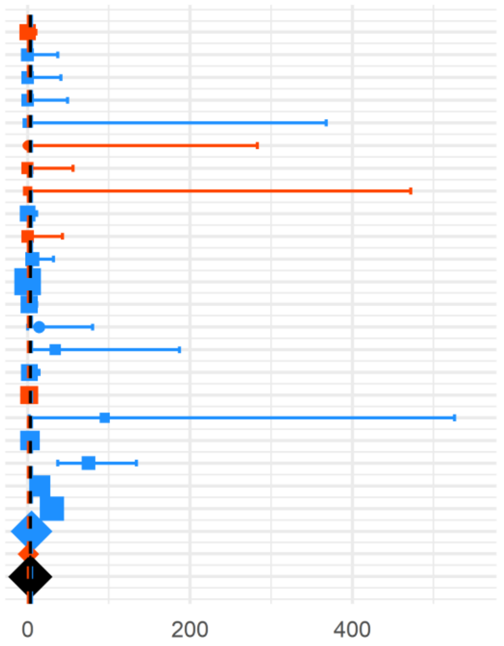

Heterogeneity:

1) $\tau^2 = 2.42$, $I^2 = 75.58$ %, $\chi^2 = 135.26$ (df = 15 , p-value = 0)

2) $\tau^2 = 0$, $I^2 = 0$ %, $\chi^2 = 1.39$ (df = 5 , p-value = 0.9253)

3) $\tau^2 = 2.54$, $I^2 = 69.14$ %, $\chi^2 = 152.03$ (df = 21 , p-value = 0)

Figure 10. Blue-coloured object: Studies without critical risk of bias, Red-coloured object: Studies with critical risk of bias, Black-coloured object: All studies regardless of the risk of bias. Circles: Randomized Controlled Trials, Squares: Non-randomized studies, diamonds: Weighted summary measures. The blue diamond shows the weighted average for studies that do not have a critical risk of bias. The red diamond shows the weighted average for studies with critical risk of bias. The black diamond shows the weighted average for all studies regardless of their risk of bias.

FIG. 11 POOLED ESTIMATE AND FOREST PLOT OF DEATHS WITH REPORTING OF FOLLOW UP TIME ASSOCIATED WITH COLONOSCOPY FOLLOWING FOBT

Study	Events	People	Weight	Point estimate per 100.000 people, 95% CI
Arana-Arri 2018	0	36346	4.37 %	0 [0 , 10]
Denis 2013	0	10024	4.37 %	0 [0 , 37]
Denis 2021b	0	9061	4.37 %	0 [0 , 41]
Ibanez 2018	0	7467	4.37 %	0 [0 , 49]
Marino 2012	0	1000	4.37 %	0 [0 , 368]
Robinson 1999	0	1300	4.37 %	0 [0 , 283]
Denis 2021a	1	17152	6.12 %	6 [0 , 32]
Derbyshire 2018	1	222488	6.12 %	0 [0 , 3]
Portillo 2018	1	48759	6.12 %	2 [0 , 11]
Randel 2021	1	6945	6.12 %	14 [0 , 80]
Saraste 2016	1	2984	6.12 %	34 [1 , 187]
Vanaclocha-Espi 2018	1	41203	6.12 %	2 [0 , 14]
Tomaszewski 2021	2	78831	7.65 %	3 [0 , 9]
Mikkelsen 2018	11	14671	9.62 %	75 [37 , 134]
Benazzato 2020	15	99674	9.77 %	15 [8 , 25]
Kooyker 2021	48	158949	10.07 %	30 [22 , 40]
1) Subtotal (not critical)	82	719208	91.31 %	5 [2 , 13]
2) Subtotal (critical)	0	37646	8.74 %	0 [0 , 10]
3) Total	82	756854	100 %	4 [1 , 11]

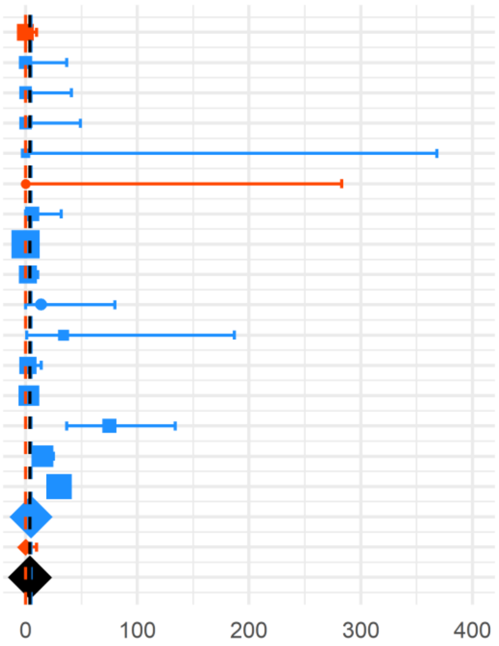

Heterogeneity:

1) $\tau^2 = 2.15$, $I^2 = 78.2$ %, $\chi^2 = 125.55$ (df = 13 , p-value = 0)

2) $\tau^2 = 0$, $I^2 = 0$ %, $\chi^2 = 0$ (df = 0 , p-value = 1)

3) $\tau^2 = 2.38$, $I^2 = 74.85$ %, $\chi^2 = 133.92$ (df = 15 , p-value = 0)

Figure 11. Blue-coloured object: Studies without critical risk of bias, Red-coloured object: Studies with critical risk of bias, Black-coloured object: All studies regardless of the risk of bias. Circles: Randomized Controlled Trials, Squares: Non-randomized studies, diamonds: Weighted summary measures. The blue diamond shows the weighted average for studies that do not have a critical risk of bias. The red diamond shows the weighted average for studies with critical risk of bias. The black diamond shows the weighted average for all studies regardless of their risk of bias.

FIG. 12 POOLED ESTIMATE AND FOREST PLOT OF DEATHS WITHOUT REPORTING OF FOLLOW UP TIME ASSOCIATED WITH COLONOSCOPY FOLLOWING FOBT

Study	Events	People	Weight	Point estimate per 100.000 people, 95% CI
Cheng 2002	0	6534	17.41 %	0 [0 , 56]
Din 2017	0	780	17.41 %	0 [0 , 472]
Steele 2009	0	8631	17.41 %	0 [0 , 43]
Lee 2012	0.01	32148	0.66 %	0 [0 , 12]
Gupta 2012	1	1057	23.55 %	95 [2 , 526]
Quyn 2018	1	53332	23.55 %	2 [0 , 10]
1) Subtotal (not critical)	1.01	33205	24.21 %	6 [0 , 79]
2) Subtotal (critical)	1	69277	75.78 %	1 [0 , 10]
3) Total	2.01	102482	100 %	2 [0 , 14]

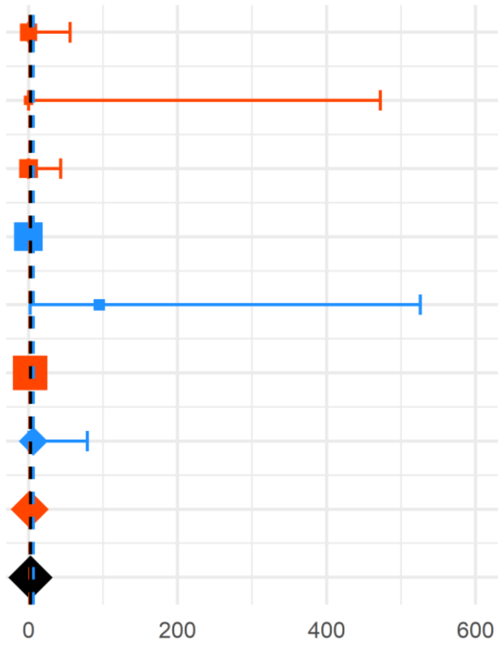

Heterogeneity:

1) $\tau^2 = 7.9$, $I^2 = 0$ %, $\chi^2 =$ NaN (df = 1 , p-value = NaN)

2) $\tau^2 = 0$, $I^2 = 0$ %, $\chi^2 = 0.52$ (df = 3 , p-value = 0.9138)

3) $\tau^2 = 0$, $I^2 = 37.91$ %, $\chi^2 =$ NaN (df = 5 , p-value = NaN)

Figure 12. Blue-coloured object: Studies without critical risk of bias, Red-coloured object: Studies with critical risk of bias, Black-coloured object: All studies regardless of the risk of bias. Circles: Randomized Controlled Trials, Squares: Non-randomized studies, diamonds: Weighted summary measures. The blue diamond shows the weighted average for studies that do not have a critical risk of bias. The red diamond shows the weighted average for studies with critical risk of bias. The black diamond shows the weighted average for all studies regardless of their risk of bias.

FIG. 13 POOLED ESTIMATE AND FOREST PLOT OF DEATHS WITH ANY FOLLOW UP TIME ASSOCIATED WITH ONCE-ONLY COLONOSCOPY

Study	Events	People	Weight	Point estimate per 100.000 people, 95% CI
Berhane 2009	0	11808	3.89 %	0 [0 , 31]
Bretthauer 2016	0	11912	3.89 %	0 [0 , 31]
Lieberman 2000	0	3121	3.89 %	0 [0 , 118]
Sieg 2006	0	109349	3.89 %	0 [0 , 3]
Stock 2013	0	8658	3.89 %	0 [0 , 43]
Zwink 2017	0	5252	3.89 %	0 [0 , 70]
Bokemeyer 2009	0	267579	3.89 %	0 [0 , 1]
Ferlitsch 2011	0	44350	3.89 %	0 [0 , 8]
Imperiale 2000	0	1994	3.89 %	0 [0 , 185]
Strul 2006	0	1177	3.89 %	0 [0 , 313]
Waldmann 2016	0	159246	3.89 %	0 [0 , 2]
Xirasagar 2020	0	25008	3.89 %	0 [0 , 15]
Stoop 2012	1	1276	4.8 %	78 [2 , 436]
Nelson 2002	3	3196	5.68 %	94 [19 , 274]
Pox 2012	7	2804983	6 %	0 [0 , 1]
Pedersen 2020	10	11163	6.07 %	90 [43 , 165]
Kobiela 2020	11	55390	6.09 %	20 [10 , 36]
Rutter 2012	15	38472	6.13 %	39 [22 , 64]
Causada-Calo 2020	19	30443	6.16 %	62 [38 , 97]
Wang 2018b	21	454271	6.17 %	5 [3 , 7]
Wang2018a	133	645095	6.25 %	21 [17 , 24]
1) Subtotal (not critical)	197	1548407	51.98 %	5 [1 , 32]
2) Subtotal (critical)	23	3145336	48.05 %	0 [0 , 18]
3) Total	220	4693743	100 %	1 [0 , 10]

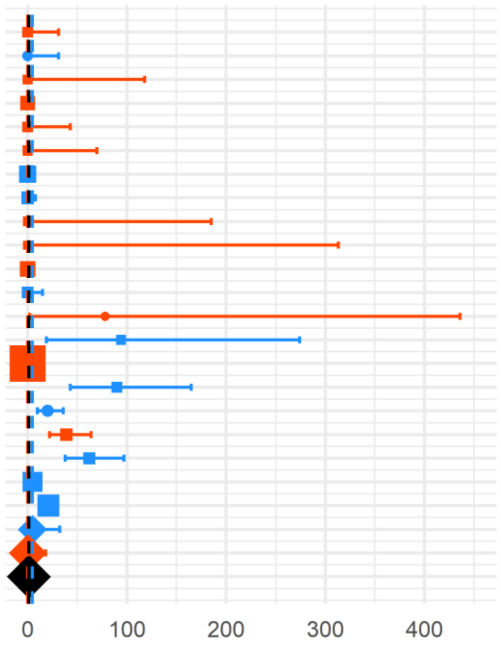

Heterogeneity:

1) $\tau^2 = 6.42$, $I^2 = 90.85$ %, $\chi^2 = 207.14$ (df = 9 , p-value = 0)

2) $\tau^2 = 9.02$, $I^2 = 92.12$ %, $\chi^2 = 113.59$ (df = 10 , p-value = 0)

3) $\tau^2 = 8.42$, $I^2 = 91.75$ %, $\chi^2 = 628.71$ (df = 20 , p-value = 0)

Figure 13. Blue-coloured object: Studies without critical risk of bias, Red-coloured object: Studies with critical risk of bias, Black-coloured object: All studies regardless of the risk of bias. Circles: Randomized Controlled Trials, Squares: Non-randomized studies, diamonds: Weighted summary measures. The blue diamond shows the weighted average for studies that do not have a critical risk of bias. The red diamond shows the weighted average for studies with critical risk of bias. The black diamond shows the weighted average for all studies regardless of their risk of bias.

FIG. 14 POOLED ESTIMATE AND FOREST PLOT OF DEATHS WITH FOLLOW UP TIME REPORTED ASSOCIATED WITH ONCE-ONLY COLONOSCOPY

Study	Events	People	Weight	Point estimate per 100.000 people, 95% CI
Berhane 2009	0	11808	4.08 %	0 [0 , 31]
Bretthauer 2016	0	11912	4.08 %	0 [0 , 31]
Lieberman 2000	0	3121	4.08 %	0 [0 , 118]
Sieg 2006	0	109349	4.08 %	0 [0 , 3]
Stock 2013	0	8658	4.08 %	0 [0 , 43]
Zwink 2017	0	5252	4.08 %	0 [0 , 70]
Stoop 2012	1	1276	5.91 %	78 [2 , 436]
Nelson 2002	3	3196	8.42 %	94 [19 , 274]
Pedersen 2020	10	11163	9.89 %	90 [43 , 165]
Kobiela 2020	11	55390	9.96 %	20 [10 , 36]
Rutter 2012	15	38472	10.15 %	39 [22 , 64]
Causada-Calo 2020	19	30443	10.26 %	62 [38 , 97]
Wang 2018b	21	454271	10.3 %	5 [3 , 7]
Wang2018a	133	645095	10.63 %	21 [17 , 24]
1) Subtotal (not critical)	197	1211470	63.54 %	23 [10 , 55]
2) Subtotal (critical)	16	177936	36.46 %	0 [0 , 1486]
3) Total	213	1389406	100 %	9 [3 , 31]

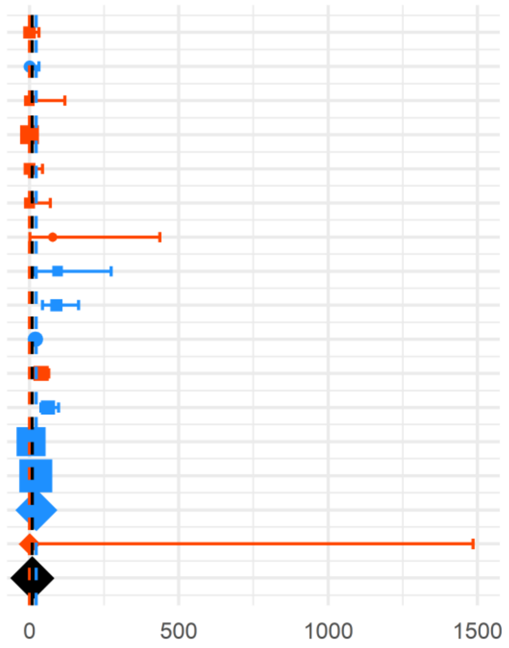

Heterogeneity:

1) $\tau^2 = 1.21$, $I^2 = 93.9$ %, $\chi^2 = 110.46$ (df = 6 , p-value = 0)

2) $\tau^2 = 8.71$, $I^2 = 0$ %, $\chi^2 = 48.34$ (df = 6 , p-value = 0)

3) $\tau^2 = 3.05$, $I^2 = 87.6$ %, $\chi^2 = 164.95$ (df = 13 , p-value = 0)

Figure 14. Blue-coloured object: Studies without critical risk of bias, Red-coloured object: Studies with critical risk of bias, Black-coloured object: All studies regardless of the risk of bias. Circles: Randomized Controlled Trials, Squares: Non-randomized studies, diamonds: Weighted summary measures. The blue diamond shows the weighted average for studies that do not have a critical risk of bias. The red diamond shows the weighted average for studies with critical risk of bias. The black diamond shows the weighted average for all studies regardless of their risk of bias.

FIG. 15 POOLED ESTIMATE AND FOREST PLOT OF DEATHS WITHOUT REPORTING OF FOLLOW UP TIME ASSOCIATED WITH ONCE-ONLY COLONOSCOPY

Study	Events	People	Weight	Point estimate per 100.000 people, 95% CI
Bokemeyer 2009	0	267579	13.24 %	0 [0 , 1]
Ferlitsch 2011	0	44350	13.24 %	0 [0 , 8]
Imperiale 2000	0	1994	13.24 %	0 [0 , 185]
Strul 2006	0	1177	13.24 %	0 [0 , 313]
Waldmann 2016	0	159246	13.24 %	0 [0 , 2]
Xirasagar 2020	0	25008	13.24 %	0 [0 , 15]
Pox 2012	7	2804983	20.53 %	0 [0 , 1]
1) Subtotal (not critical)	0	336937	39.72 %	0 [0 , 1]
2) Subtotal (critical)	7	2967400	60.25 %	0 [0 , 0]
3) Total	7	3304337	100 %	0 [0 , 0]

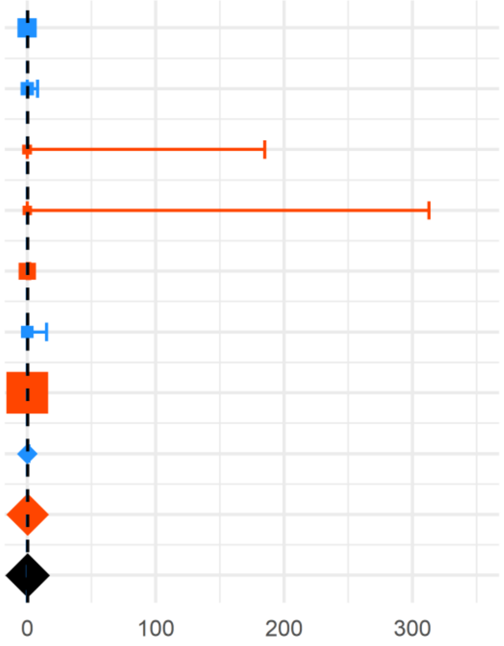

Heterogeneity:

1) $\tau^2 = 0$, $I^2 = 0$ %, $\chi^2 = 0$ (df = 0 , p-value = 1)

2) $\tau^2 = 0$, $I^2 = 0$ %, $\chi^2 = 0.79$ (df = 3 , p-value = 0.8523)

3) $\tau^2 = 0$, $I^2 = 0$ %, $\chi^2 = 2.29$ (df = 6 , p-value = 0.8908)

Figure 15. Blue-coloured object: Studies without critical risk of bias, Red-coloured object: Studies with critical risk of bias, Black-coloured object: All studies regardless of the risk of bias. Circles: Randomized Controlled Trials, Squares: Non-randomized studies, diamonds: Weighted summary measures. The blue diamond shows the weighted average for studies that do not have a critical risk of bias. The red diamond shows the weighted average for studies with critical risk of bias. The black diamond shows the weighted average for all studies regardless of their risk of bias.

FIG. 16 POOLED ESTIMATE AND FOREST PLOT OF DEATHS WITH ANY FOLLOW UP TIME ASSOCIATED WITH COLONOSCOPY FOLLOWING VARIOUS SCREENING TESTS

Study	Events	People	Weight	Point estimate per 100.000 people, 95% CI
Castro 2013	0	3215	7.69 %	0 [0 , 115]
Dellon 2009	0	3614	7.69 %	0 [0 , 102]
Randel 2021	0	3297	7.69 %	0 [0 , 112]
Shroff 2015	0	6152	7.69 %	0 [0 , 60]
Holme 2014	0	2746	7.69 %	0 [0 , 134]
Atkin 2002	1	2051	15.38 %	49 [1 , 271]
Rutter 2012	3	4984	46.15 %	60 [12 , 176]
1) Subtotal (not critical)	1	8962	30.76 %	11 [2 , 79]
2) Subtotal (critical)	3	17097	69.22 %	3 [0 , 3000]
3) Total	4	26059	100 %	7 [0 , 95]

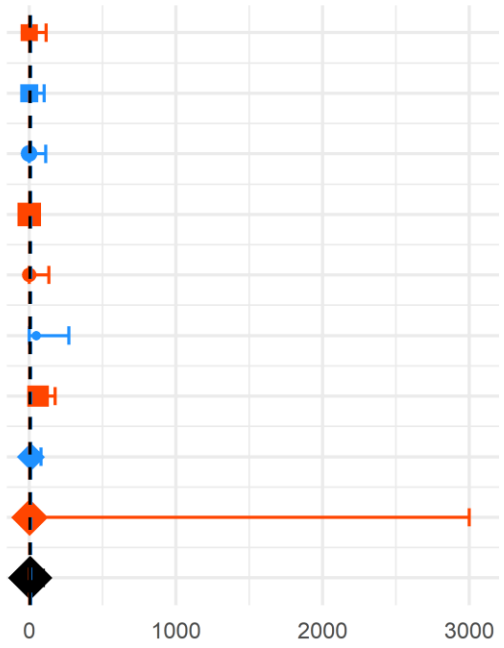

Heterogeneity:

1) $\tau^2 = 0$, $I^2 = 0$ %, $\chi^2 = 2.95$ (df = 2 , p-value = 0.2289)

2) $\tau^2 = 2.98$, $I^2 = 0$ %, $\chi^2 = 7.4$ (df = 3 , p-value = 0.0603)

3) $\tau^2 = 1.54$, $I^2 = 0$ %, $\chi^2 = 10.51$ (df = 6 , p-value = 0.1047)

Figure 16. Blue-coloured object: Studies without critical risk of bias, Red-coloured object: Studies with critical risk of bias, Black-coloured object: All studies regardless of the risk of bias. Circles: Randomized Controlled Trials, Squares: Non-randomized studies, diamonds: Weighted summary measures. The blue diamond shows the weighted average for studies that do not have a critical risk of bias. The red diamond shows the weighted average for studies with critical risk of bias. The black diamond shows the weighted average for all studies regardless of their risk of bias.

FIG. 17 POOLED ESTIMATE AND FOREST PLOT OF DEATHS WITH REPORTING OF FOLLOW UP TIME ASSOCIATED WITH COLONOSCOPY FOLLOWING VARIOUS SCREENING TESTS

Study	Events	People	Weight	Point estimate per 100.000 people, 95% CI
Castro 2013	0	3215	8.33 %	0 [0 , 115]
Dellon 2009	0	3614	8.33 %	0 [0 , 102]
Randel 2021	0	3297	8.33 %	0 [0 , 112]
Shroff 2015	0	6152	8.33 %	0 [0 , 60]
Atkin 2002	1	2051	16.67 %	49 [1 , 271]
Rutter 2012	3	4984	50 %	60 [12 , 176]
1) Subtotal (not critical)	1	8962	33.33 %	11 [2 , 79]
2) Subtotal (critical)	3	14351	66.66 %	9 [0 , 421]
3) Total	4	23313	100 %	9 [1 , 93]

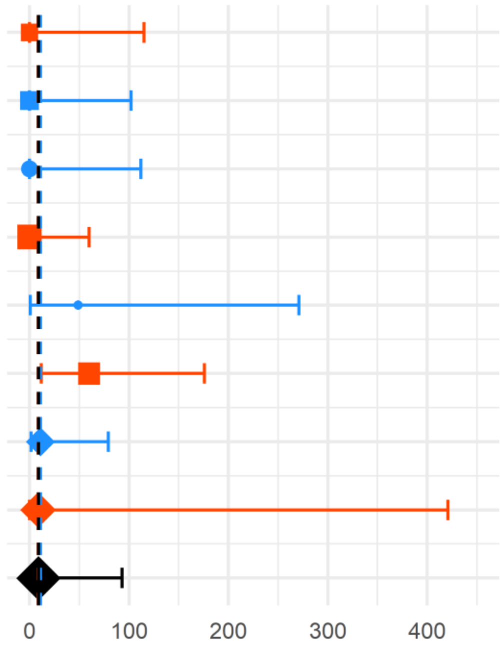

Heterogeneity:

1) $\tau^2 = 0$, $I^2 = 0$ %, $\chi^2 = 2.95$ (df = 2 , p-value = 0.2289)

2) $\tau^2 = 2.04$, $I^2 = 0$ %, $\chi^2 = 6.35$ (df = 2 , p-value = 0.0419)

3) $\tau^2 = 1.28$, $I^2 = 0$ %, $\chi^2 = 9.62$ (df = 5 , p-value = 0.0868)

Figure 17. Blue-coloured object: Studies without critical risk of bias, Red-coloured object: Studies with critical risk of bias, Black-coloured object: All studies regardless of the risk of bias. Circles: Randomized Controlled Trials, Squares: Non-randomized studies, diamonds: Weighted summary measures. The blue diamond shows the weighted average for studies that do not have a critical risk of bias. The red diamond shows the weighted average for studies with critical risk of bias. The black diamond shows the weighted average for all studies regardless of their risk of bias.

FIG. 18 POOLED ESTIMATE AND FOREST PLOT OF VASOVAGAL EVENTS WITH SHORT-TERM FOLLOW UP ASSOCIATED WITH FLEXIBLE SIGMOIDOSCOPY

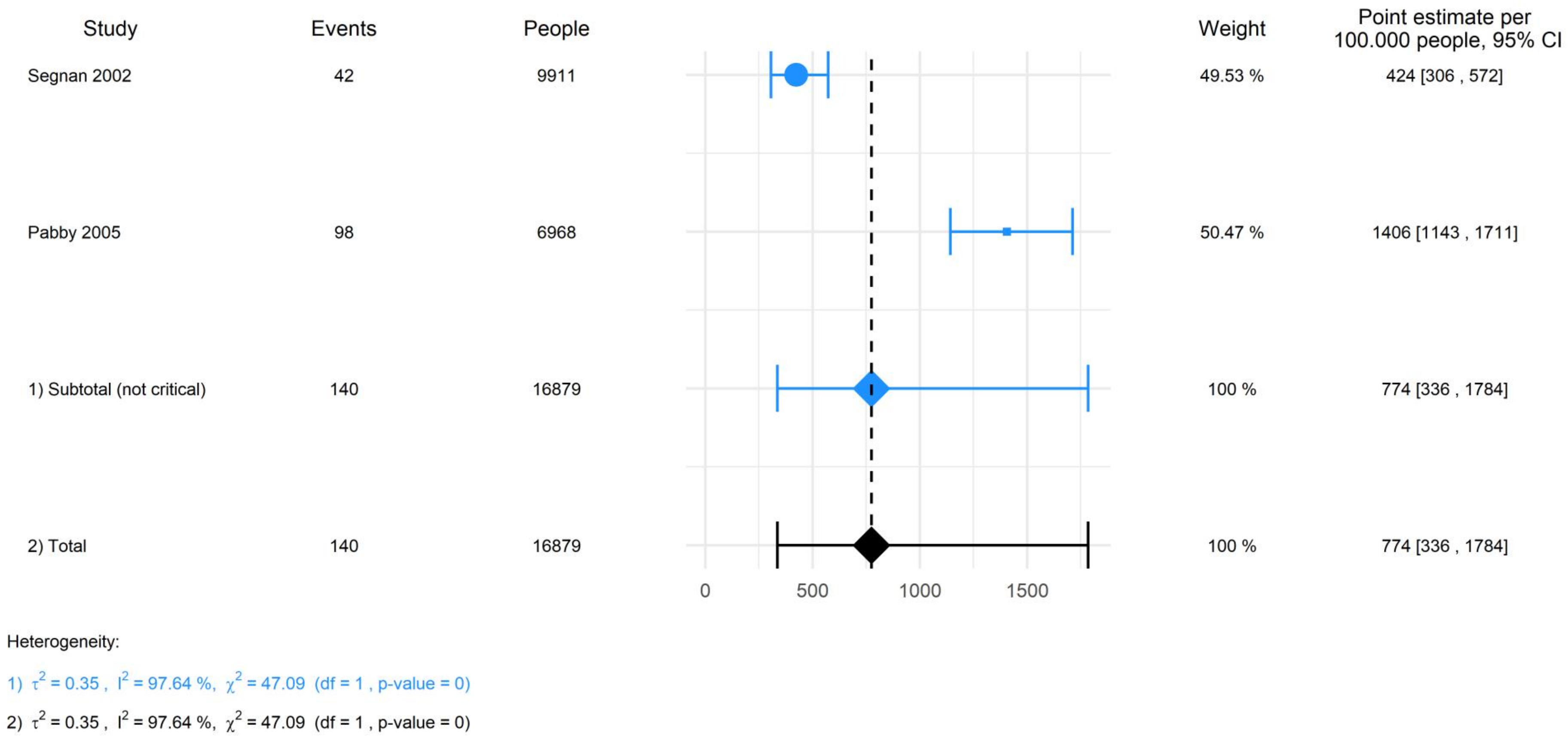

Figure 18. Blue-coloured object: Studies without critical risk of bias, Red-coloured object: Studies with critical risk of bias, Black-coloured object: All studies regardless of the risk of bias. Circles: Randomized Controlled Trials, Squares: Non-randomized studies, diamonds: Weighted summary measures. The blue diamond shows the weighted average for studies that do not have a critical risk of bias. The red diamond shows the weighted average for studies with critical risk of bias. The black diamond shows the weighted average for all studies regardless of their risk of bias.

FIG. 19 POOLED ESTIMATE AND FOREST PLOT OF ACUTE CORONARY SYNDROME WITH LONG-TERM FOLLOW UP ASSOCIATED WITH FLEXIBLE SIGMOIDOSCOPY

Study	Events	People	Weight	Point estimate per 100.000 people, 95% CI
Atkin 2002	2	40674	42.98 %	5 [1 , 18]
Levin 2002	33	107704	57.02 %	31 [21 , 43]
1) Subtotal (not critical)	2	40674	42.98 %	5 [1 , 18]
2) Subtotal (critical)	33	107704	57.02 %	31 [21 , 43]
3) Total	35	148378	100 %	14 [4 , 52]

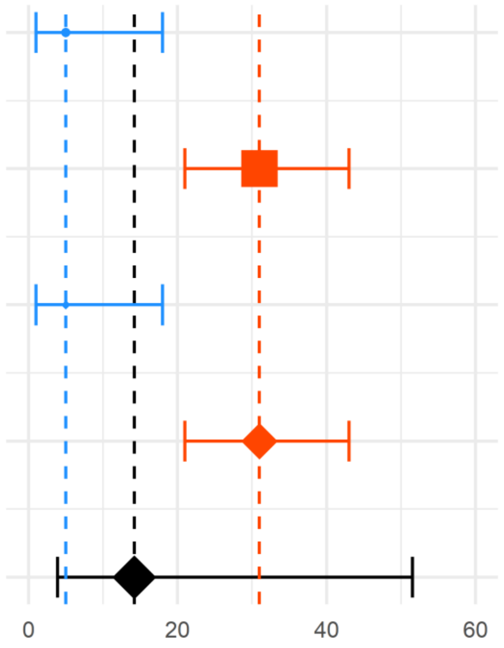

Heterogeneity:

1) τ^2 = . , I^2 = . , χ^2 = .

2) τ^2 = . , I^2 = . , χ^2 = .

3) $\tau^2 = 0.65$, $I^2 = 84.16$ %, $\chi^2 = 10.99$ (df = 1 , p-value = 9e-04)

Figure 19. Blue-coloured object: Studies without critical risk of bias, Red-coloured object: Studies with critical risk of bias, Black-coloured object: All studies regardless of the risk of bias. Circles: Randomized Controlled Trials, Squares: Non-randomized studies, diamonds: Weighted summary measures. The blue diamond shows the weighted average for studies that do not have a critical risk of bias. The red diamond shows the weighted average for studies with critical risk of bias. The black diamond shows the weighted average for all studies regardless of their risk of bias.

FIG. 20 POOLED ESTIMATE AND FOREST PLOT OF ACUTE CORONARY SYNDROME WITH LONG-TERM FOLLOW UP ASSOCIATED WITH COLONOSCOPY FOLLOWING FOBT

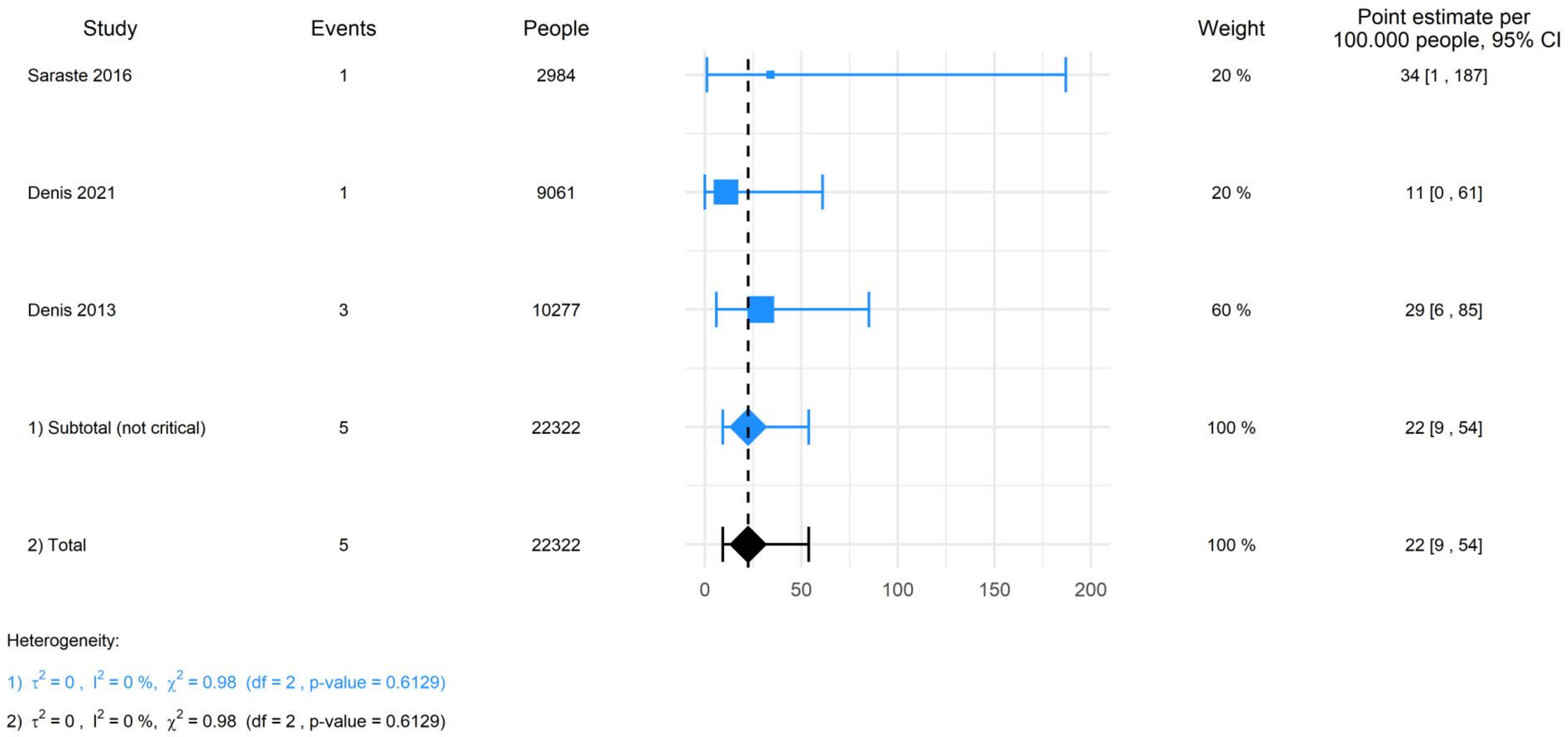

Figure 20. Blue-coloured object: Studies without critical risk of bias, Red-coloured object: Studies with critical risk of bias, Black-coloured object: All studies regardless of the risk of bias. Circles: Randomized Controlled Trials, Squares: Non-randomized studies, diamonds: Weighted summary measures. The blue diamond shows the weighted average for studies that do not have a critical risk of bias. The red diamond shows the weighted average for studies with critical risk of bias. The black diamond shows the weighted average for all studies regardless of their risk of bias.

FIG. 21 POOLED ESTIMATE AND FOREST PLOT OF ARRHYTHMIA WITH LONG-TERM FOLLOW UP ASSOCIATED WITH COLONOSCOPY FOLLOWING FOBT

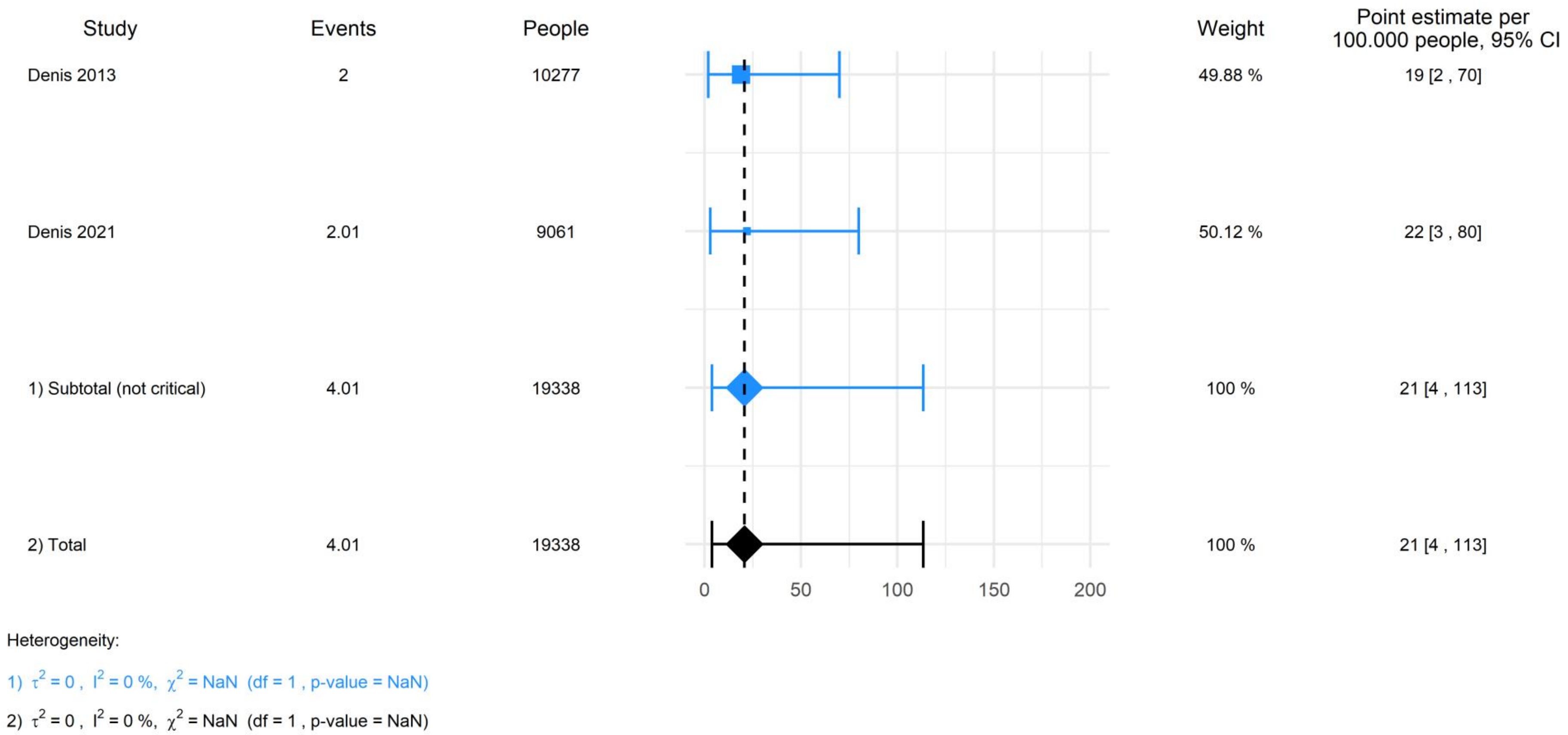

Figure 21. Blue-coloured object: Studies without critical risk of bias, Red-coloured object: Studies with critical risk of bias, Black-coloured object: All studies regardless of the risk of bias. Circles: Randomized Controlled Trials, Squares: Non-randomized studies, diamonds: Weighted summary measures. The blue diamond shows the weighted average for studies that do not have a critical risk of bias. The red diamond shows the weighted average for studies with critical risk of bias. The black diamond shows the weighted average for all studies regardless of their risk of bias.

FIG. 22 POOLED ESTIMATE AND FOREST PLOT OF THROMBOEMBOLIC EVENTS WITH LONG-TERM FOLLOW UP ASSOCIATED WITH COLONOSCOPY FOLLOWING FOBT

Study	Events	People	Weight	Point estimate per 100.000 people, 95% CI
Saraste 2016	1	2984	16.67 %	34 [1 , 187]
Denis 2013b	1	10277	16.67 %	10 [0 , 54]
Denis 2021	1	9061	16.67 %	11 [0 , 61]
Denis 2013a	3	10277	50 %	29 [6 , 85]
1) Subtotal (not critical)	6	32599	100 %	18 [8 , 41]
2) Total	6	32599	100 %	18 [8 , 41]

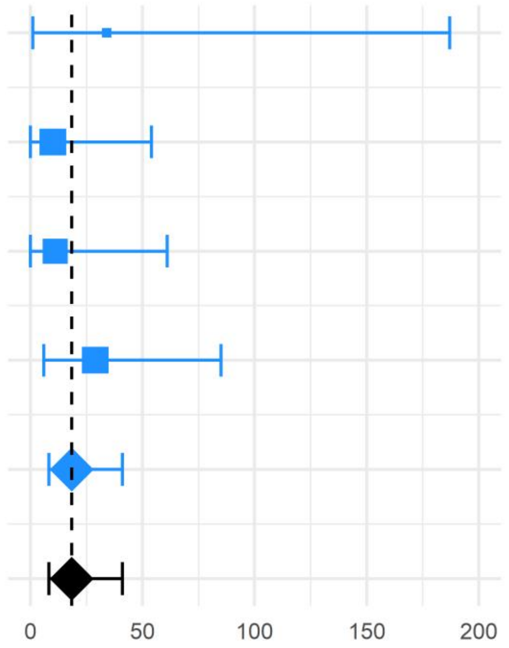

Heterogeneity:

1) $\tau^2 = 0$, $I^2 = 0$ %, $\chi^2 = 1.67$ (df = 3 , p-value = 0.644)

2) $\tau^2 = 0$, $I^2 = 0$ %, $\chi^2 = 1.67$ (df = 3 , p-value = 0.644)

Figure 22. Blue-coloured object: Studies without critical risk of bias, Red-coloured object: Studies with critical risk of bias, Black-coloured object: All studies regardless of the risk of bias. Circles: Randomized Controlled Trials, Squares: Non-randomized studies, diamonds: Weighted summary measures. The blue diamond shows the weighted average for studies that do not have a critical risk of bias. The red diamond shows the weighted average for studies with critical risk of bias. The black diamond shows the weighted average for all studies regardless of their risk of bias.

FIG. 23 POOLED ESTIMATE AND FOREST PLOT OF ACUTE CORONARY SYNDROME WITH SHORT-TERM FOLLOW UP ASSOCIATED WITH ONCE-ONLY COLONOSCOPY

Study	Events	People	Weight	Point estimate per 100.000 people, 95% CI
Pedersen 2020	0	11163	30.46 %	0 [0 , 33]
Taleban 2018	1	99	34.77 %	1010 [26 , 5500]
Liebermann 2000	1	3121	34.77 %	32 [1 , 178]
1) Subtotal (not critical)	2	14383	100 %	21 [1 , 514]
2) Total	2	14383	100 %	21 [1 , 514]

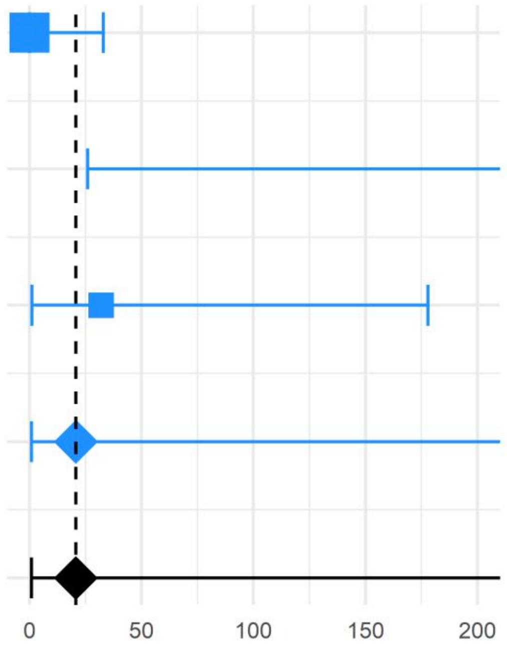

Heterogeneity:

1) $\tau^2 = 6.28$, $I^2 = 66.41$ %, $\chi^2 = 10.24$ (df = 2 , p-value = 0.006)

2) $\tau^2 = 6.28$, $I^2 = 66.41$ %, $\chi^2 = 10.24$ (df = 2 , p-value = 0.006)

Figure 23. Blue-coloured object: Studies without critical risk of bias, Red-coloured object: Studies with critical risk of bias, Black-coloured object: All studies regardless of the risk of bias. Circles: Randomized Controlled Trials, Squares: Non-randomized studies, diamonds: Weighted summary measures. The blue diamond shows the weighted average for studies that do not have a critical risk of bias. The red diamond shows the weighted average for studies with critical risk of bias. The black diamond shows the weighted average for all studies regardless of their risk of bias.

FIG. 24 POOLED ESTIMATE AND FOREST PLOT OF PULMONARY EVENTS WITH SHORT-TERM FOLLOW UP ASSOCIATED WITH ONCE-ONLY COLONOSCOPY

Study	Events	People	Weight	Point estimate per 100.000 people, 95% CI
Pedersen 2020	15	11163	23.59 %	134 [75 , 222]
Wang2018b1	87	454271	25.27 %	19 [15 , 24]
Wang2018b3	299	454271	25.54 %	66 [59 , 74]
Wang2018b2	524	454271	25.59 %	115 [106 , 126]
1) Subtotal (not critical)	925	1373976	100 %	65 [31 , 137]
2) Total	925	1373976	100 %	65 [31 , 137]

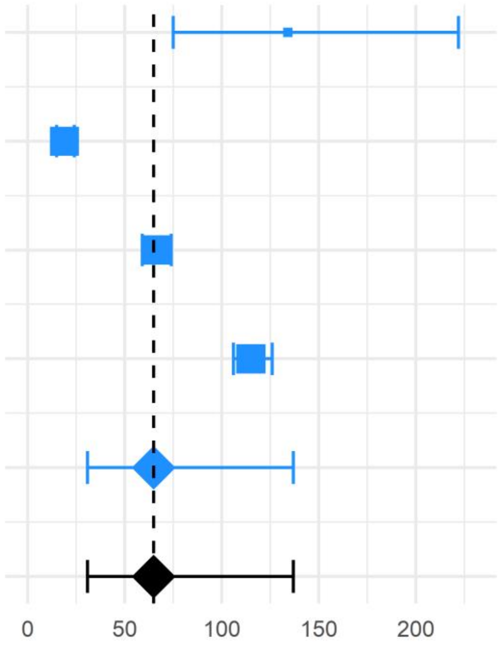

Heterogeneity:

1) $\tau^2 = 0.56$, $I^2 = 98.86$ %, $\chi^2 = 352.81$ (df = 3 , p-value = 0)

2) $\tau^2 = 0.56$, $I^2 = 98.86$ %, $\chi^2 = 352.81$ (df = 3 , p-value = 0)

Figure 24. Blue-coloured object: Studies without critical risk of bias, Red-coloured object: Studies with critical risk of bias, Black-coloured object: All studies regardless of the risk of bias. Circles: Randomized Controlled Trials, Squares: Non-randomized studies, diamonds: Weighted summary measures. The blue diamond shows the weighted average for studies that do not have a critical risk of bias. The red diamond shows the weighted average for studies with critical risk of bias. The black diamond shows the weighted average for all studies regardless of their risk of bias.

FIG. 25 POOLED ESTIMATE AND FOREST PLOT OF STROKE EVENTS WITH SHORT-TERM FOLLOW UP ASSOCIATED WITH ONCE-ONLY COLONOSCOPY

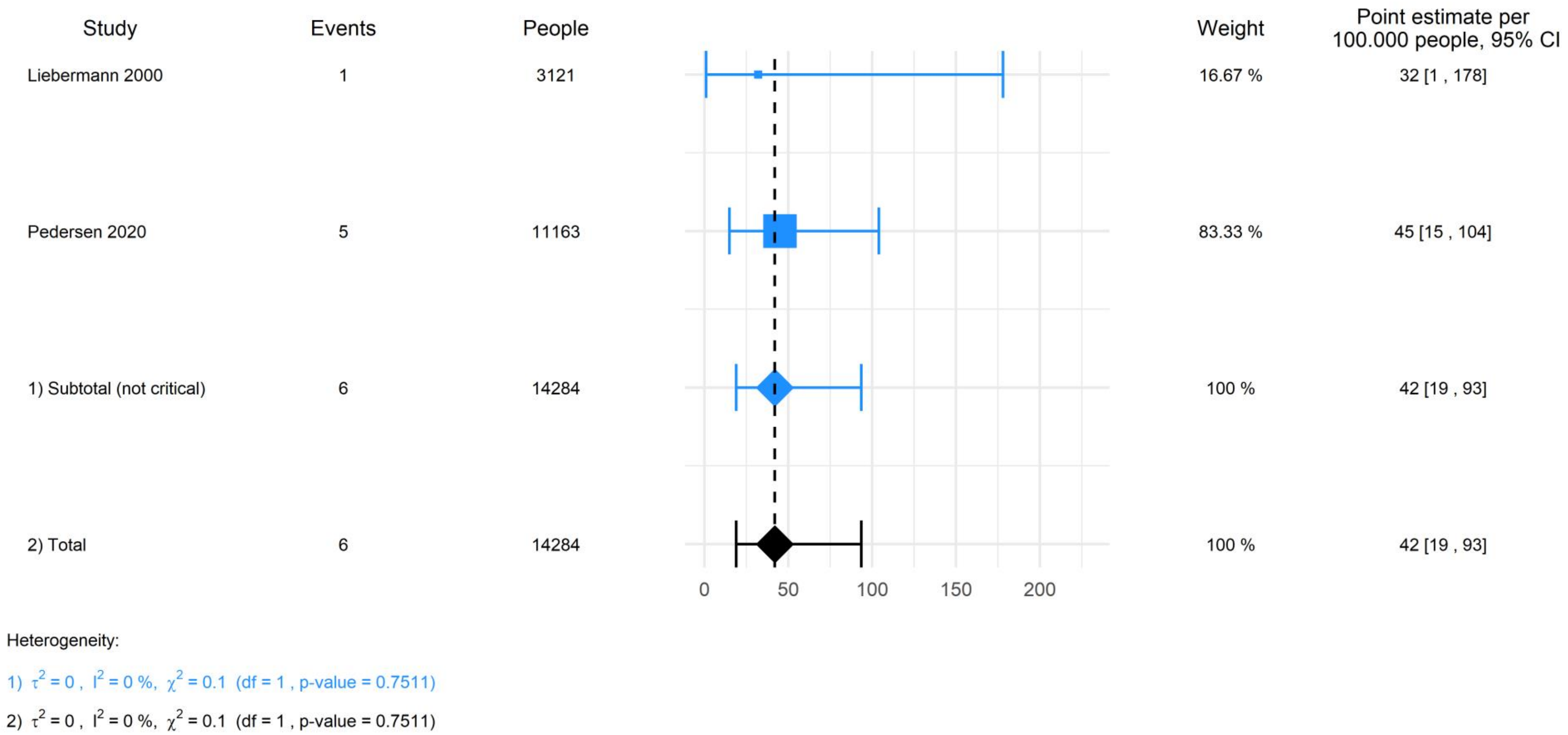

Figure 25. Blue-coloured object: Studies without critical risk of bias, Red-coloured object: Studies with critical risk of bias, Black-coloured object: All studies regardless of the risk of bias. Circles: Randomized Controlled Trials, Squares: Non-randomized studies, diamonds: Weighted summary measures. The blue diamond shows the weighted average for studies that do not have a critical risk of bias. The red diamond shows the weighted average for studies with critical risk of bias. The black diamond shows the weighted average for all studies regardless of their risk of bias.

FIG. 26 POOLED ESTIMATE AND FOREST PLOT OF VASOVAGAL EVENTS WITH SHORT-TERM FOLLOW UP ASSOCIATED WITH ONCE-ONLY COLONOSCOPY

Study	Events	People	Weight	Point estimate per 100.000 people, 95% CI
Pedersen 2020	8	11163	48.35 %	72 [31 , 141]
Bretthauer 2016	51	11912	51.65 %	428 [319 , 563]
1) Subtotal (not critical)	51	11912	51.65 %	428 [319 , 563]
2) Subtotal (critical)	8	11163	48.35 %	72 [31 , 141]
3) Total	59	23075	100 %	180 [51 , 633]

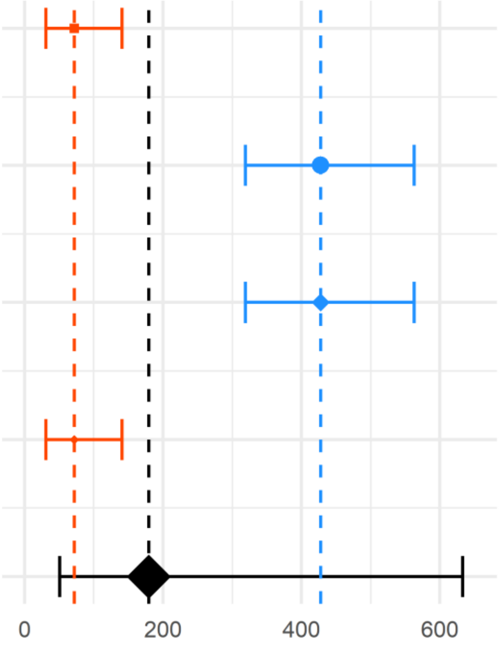

Heterogeneity:

1) τ^2 = . , I^2 = . , χ^2 = .

2) τ^2 = . , I^2 = . , χ^2 = .

3) τ^2 = 0.76 , I^2 = 95.47 %, χ^2 = 32.23 (df = 1 , p-value = 0)

Figure 26. Blue-coloured object: Studies without critical risk of bias, Red-coloured object: Studies with critical risk of bias, Black-coloured object: All studies regardless of the risk of bias. Circles: Randomized Controlled Trials, Squares: Non-randomized studies, diamonds: Weighted summary measures. The blue diamond shows the weighted average for studies that do not have a critical risk of bias. The red diamond shows the weighted average for studies with critical risk of bias. The black diamond shows the weighted average for all studies regardless of their risk of bias.

FIG. 27 POOLED ESTIMATE AND FOREST PLOT OF ACUTE CORONARY SYNDROME WITH LONG-TERM FOLLOW UP ASSOCIATED WITH ONCE-ONLY COLONOSCOPY

Study	Events	People	Weight	Point estimate per 100.000 people, 95% CI
Stoop 2012	0	1276	5.85 %	0 [0 , 289]
Nelson 2002	1	3196	8.65 %	31 [1 , 174]
Berhane 2009	1	11808	8.65 %	8 [0 , 47]
Stock 2013	2	8658	11.38 %	23 [3 , 83]
Causada-Calo 2020	35	30443	16.17 %	115 [80 , 160]
Garcia-Albeniz 2017a	66	46872	16.37 %	141 [109 , 179]
Garcia-Albeniz 2017c	82	31193	16.41 %	263 [209 , 326]
Wang2018a	160	645095	16.5 %	25 [21 , 29]
1) Subtotal (not critical)	346	765457	85.48 %	71 [32 , 157]
2) Subtotal (critical)	1	13084	14.5 %	8 [1 , 54]
3) Total	347	778541	100 %	49 [20 , 118]

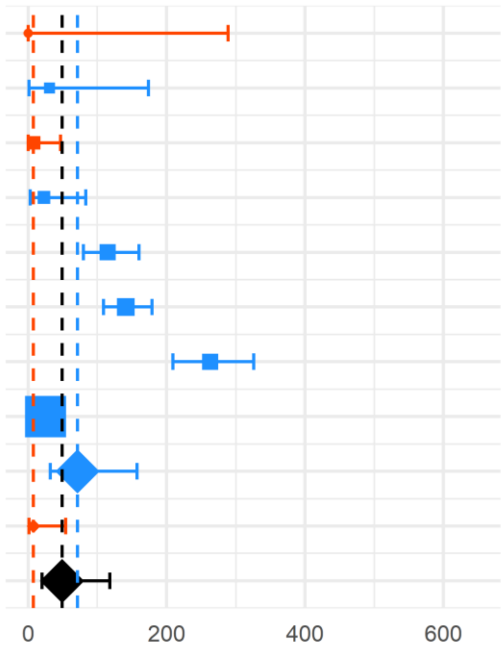

Heterogeneity:

1) $\tau^2 = 0.84$, $I^2 = 98.61$ %, $\chi^2 = 308.58$ (df = 5 , p-value = 0)

2) $\tau^2 = 0$, $I^2 = 0$ %, $\chi^2 = 0.21$ (df = 1 , p-value = 0.6505)

3) $\tau^2 = 1.17$, $I^2 = 98.08$ %, $\chi^2 = 314.99$ (df = 7 , p-value = 0)

Figure 27. Blue-coloured object: Studies without critical risk of bias, Red-coloured object: Studies with critical risk of bias, Black-coloured object: All studies regardless of the risk of bias. Circles: Randomized Controlled Trials, Squares: Non-randomized studies, diamonds: Weighted summary measures. The blue diamond shows the weighted average for studies that do not have a critical risk of bias. The red diamond shows the weighted average for studies with critical risk of bias. The black diamond shows the weighted average for all studies regardless of their risk of bias.

FIG. 28 POOLED ESTIMATE AND FOREST PLOT OF ARRHYTHMIA WITH LONG-TERM FOLLOW UP ASSOCIATED WITH ONCE-ONLY COLONOSCOPY

Study	Events	People	Weight	Point estimate per 100.000 people, 95% CI
Stoop 2012	1	1276	12.41 %	78 [2 , 436]
Nelson 2002	1	3196	12.41 %	31 [1 , 174]
Causada-Calo 2020	14	30443	18.29 %	46 [25 , 77]
Garcia-Albeniz 2017a	297	46872	18.95 %	634 [564 , 710]
Garcia-Albeniz 2017c	351	31193	18.96 %	1125 [1011 , 1249]
Wang2018a	1473	645095	18.98 %	228 [217 , 240]
1) Subtotal (not critical)	2136	756799	87.59 %	204 [60 , 689]
2) Subtotal (critical)	1	1276	12.41 %	78 [2 , 436]
3) Total	2137	758075	100 %	176 [58 , 533]

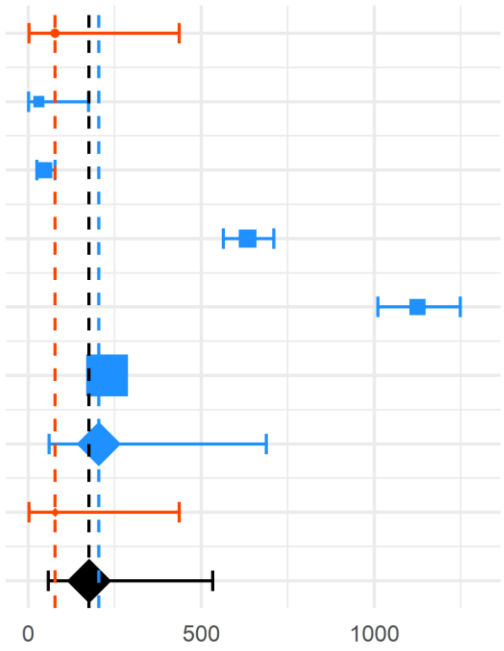

Heterogeneity:

1) τ^2 = 1.82 , I^2 = 99.56 %, χ^2 = 771.69 (df = 4 , p-value = 0)

2) τ^2 = . , I^2 = . χ^2 = .

3) τ^2 = 1.72 , I^2 = 99.45 %, χ^2 = 774.32 (df = 5 , p-value = 0)

Figure 28. Blue-coloured object: Studies without critical risk of bias, Red-coloured object: Studies with critical risk of bias, Black-coloured object: All studies regardless of the risk of bias. Circles: Randomized Controlled Trials, Squares: Non-randomized studies, diamonds: Weighted summary measures. The blue diamond shows the weighted average for studies that do not have a critical risk of bias. The red diamond shows the weighted average for studies with critical risk of bias. The black diamond shows the weighted average for all studies regardless of their risk of bias.

FIG. 29 POOLED ESTIMATE AND FOREST PLOT OF HEART FAILURE WITH LONG-TERM FOLLOW UP ASSOCIATED WITH ONCE-ONLY COLONOSCOPY

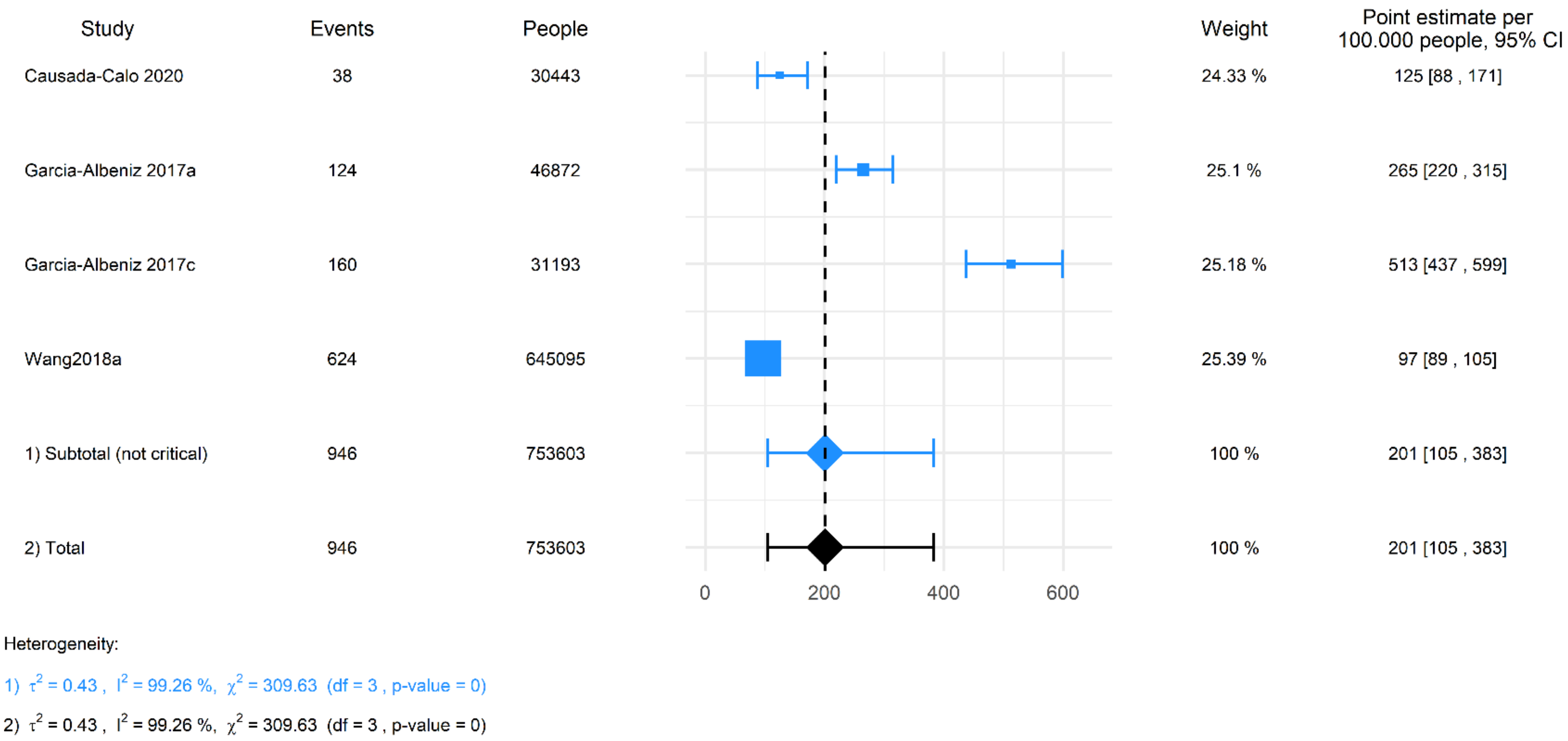

Figure 29. Blue-coloured object: Studies without critical risk of bias, Red-coloured object: Studies with critical risk of bias, Black-coloured object: All studies regardless of the risk of bias. Circles: Randomized Controlled Trials, Squares: Non-randomized studies, diamonds: Weighted summary measures. The blue diamond shows the weighted average for studies that do not have a critical risk of bias. The red diamond shows the weighted average for studies with critical risk of bias. The black diamond shows the weighted average for all studies regardless of their risk of bias.

FIG. 30 POOLED ESTIMATE AND FOREST PLOT OF PULMONARY EVENTS WITH LONG-TERM FOLLOW UP ASSOCIATED WITH ONCE-ONLY COLONOSCOPY

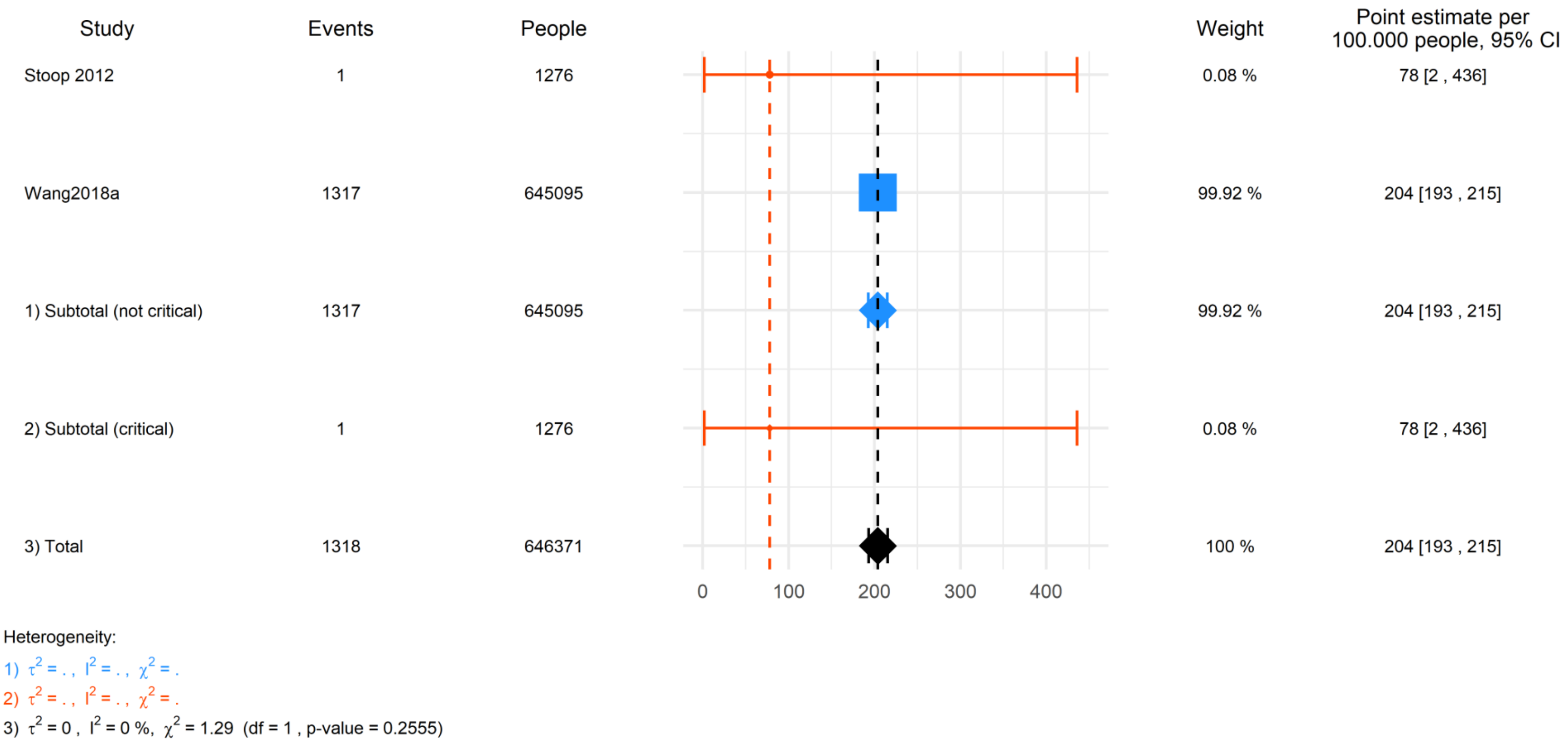

Figure 30. Blue-coloured object: Studies without critical risk of bias, Red-coloured object: Studies with critical risk of bias, Black-coloured object: All studies regardless of the risk of bias. Circles: Randomized Controlled Trials, Squares: Non-randomized studies, diamonds: Weighted summary measures. The blue diamond shows the weighted average for studies that do not have a critical risk of bias. The red diamond shows the weighted average for studies with critical risk of bias. The black diamond shows the weighted average for all studies regardless of their risk of bias.

FIG. 31 POOLED ESTIMATE AND FOREST PLOT OF STROKE EVENTS WITH LONG-TERM FOLLOW UP ASSOCIATED WITH ONCE-ONLY COLONOSCOPY

Study	Events	People	Weight	Point estimate per 100.000 people, 95% CI
Stoop 2012	0	1276	0.79 %	0 [0 , 289]
Stock 2013	3	8658	4.39 %	35 [7 , 101]
Nelson 2002	4	3196	5.69 %	125 [34 , 320]
Wang2018a1	328	645095	44.12 %	51 [45 , 57]
Wang2018a2	429	645095	45.01 %	67 [60 , 73]
1) Subtotal (not critical)	764	1302044	99.21 %	58 [49 , 70]
2) Subtotal (critical)	0	1276	0.79 %	0 [0 , 289]
3) Total	764	1303320	100 %	58 [48 , 69]

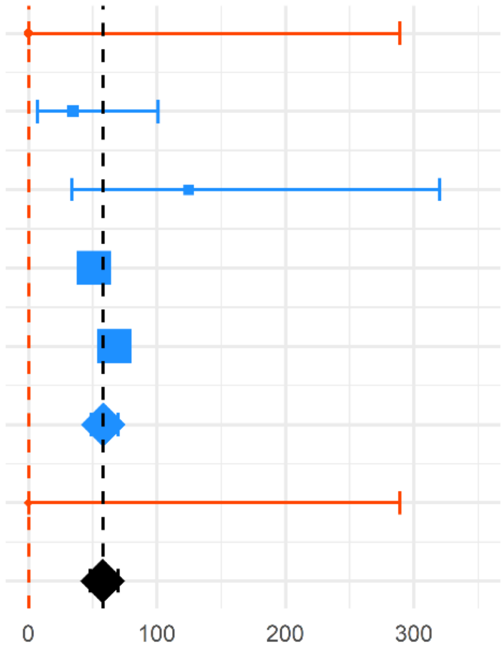

Heterogeneity:

1) τ^2 = 0.02 , I^2 = 81.81 %, χ^2 = 16.33 (df = 3 , p-value = 0.001

2) τ^2 = . , I^2 = . χ^2 = .

3) τ^2 = 0.02 , I^2 = 75.75 %, χ^2 = 17.82 (df = 4 , p-value = 0.001

Figure 31. Blue-coloured object: Studies without critical risk of bias, Red-coloured object: Studies with critical risk of bias, Black-coloured object: All studies regardless of the risk of bias. Circles: Randomized Controlled Trials, Squares: Non-randomized studies, diamonds: Weighted summary measures. The blue diamond shows the weighted average for studies that do not have a critical risk of bias. The red diamond shows the weighted average for studies with critical risk of bias. The black diamond shows the weighted average for all studies regardless of their risk of bias.

FIG. 32 POOLED ESTIMATE AND FOREST PLOT OF THROMBOEMBOLIC EVENTS WITH LONG-TERM FOLLOW UP ASSOCIATED WITH ONCE-ONLY COLONOSCOPY

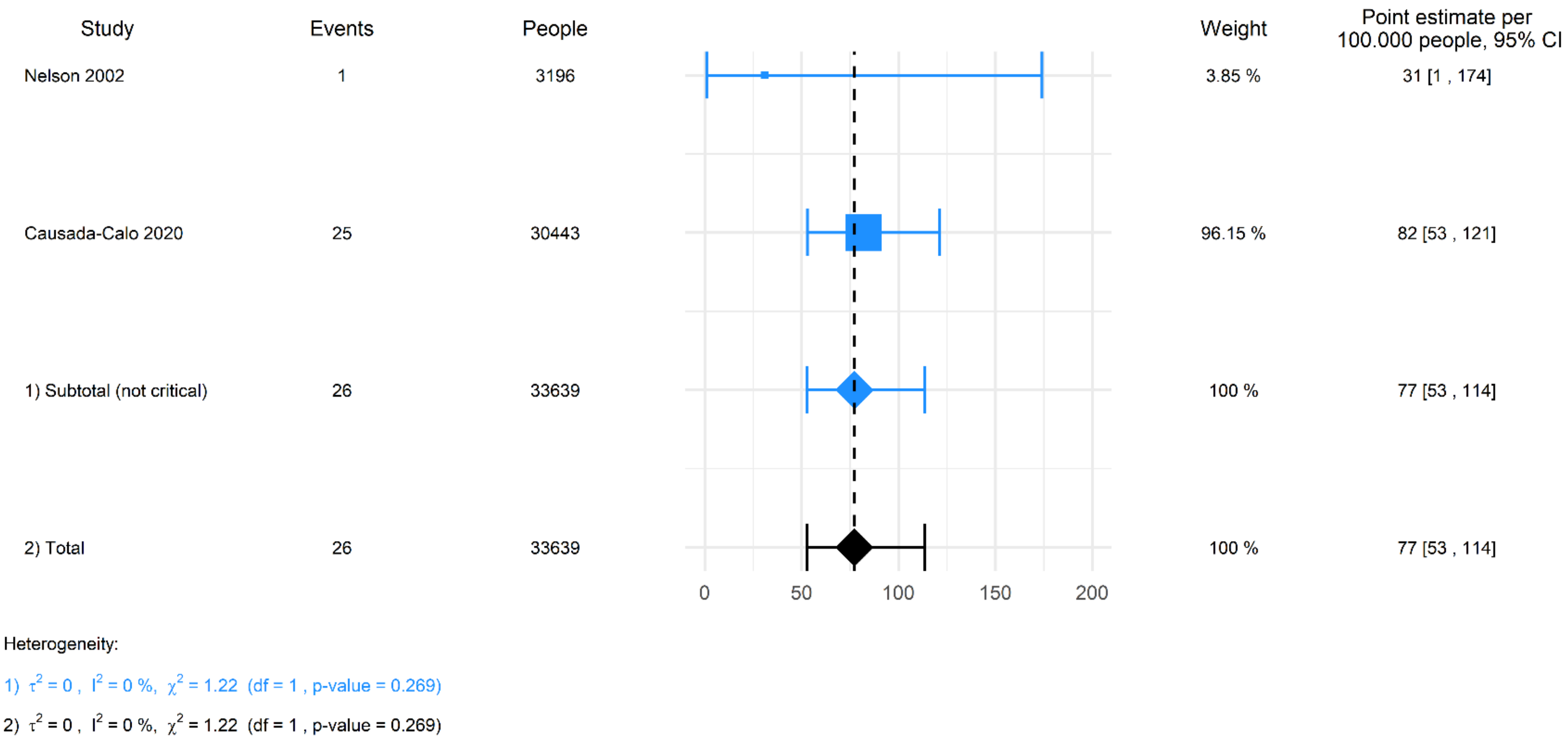

Figure 32. Blue-coloured object: Studies without critical risk of bias, Red-coloured object: Studies with critical risk of bias, Black-coloured object: All studies regardless of the risk of bias. Circles: Randomized Controlled Trials, Squares: Non-randomized studies, diamonds: Weighted summary measures. The blue diamond shows the weighted average for studies that do not have a critical risk of bias. The red diamond shows the weighted average for studies with critical risk of bias. The black diamond shows the weighted average for all studies regardless of their risk of bias.

FIG. 33 POOLED ESTIMATE AND FOREST PLOT OF VASOVAGAL EVENTS WITH LONG-TERM FOLLOW UP ASSOCIATED WITH ONCE-ONLY COLONOSCOPY

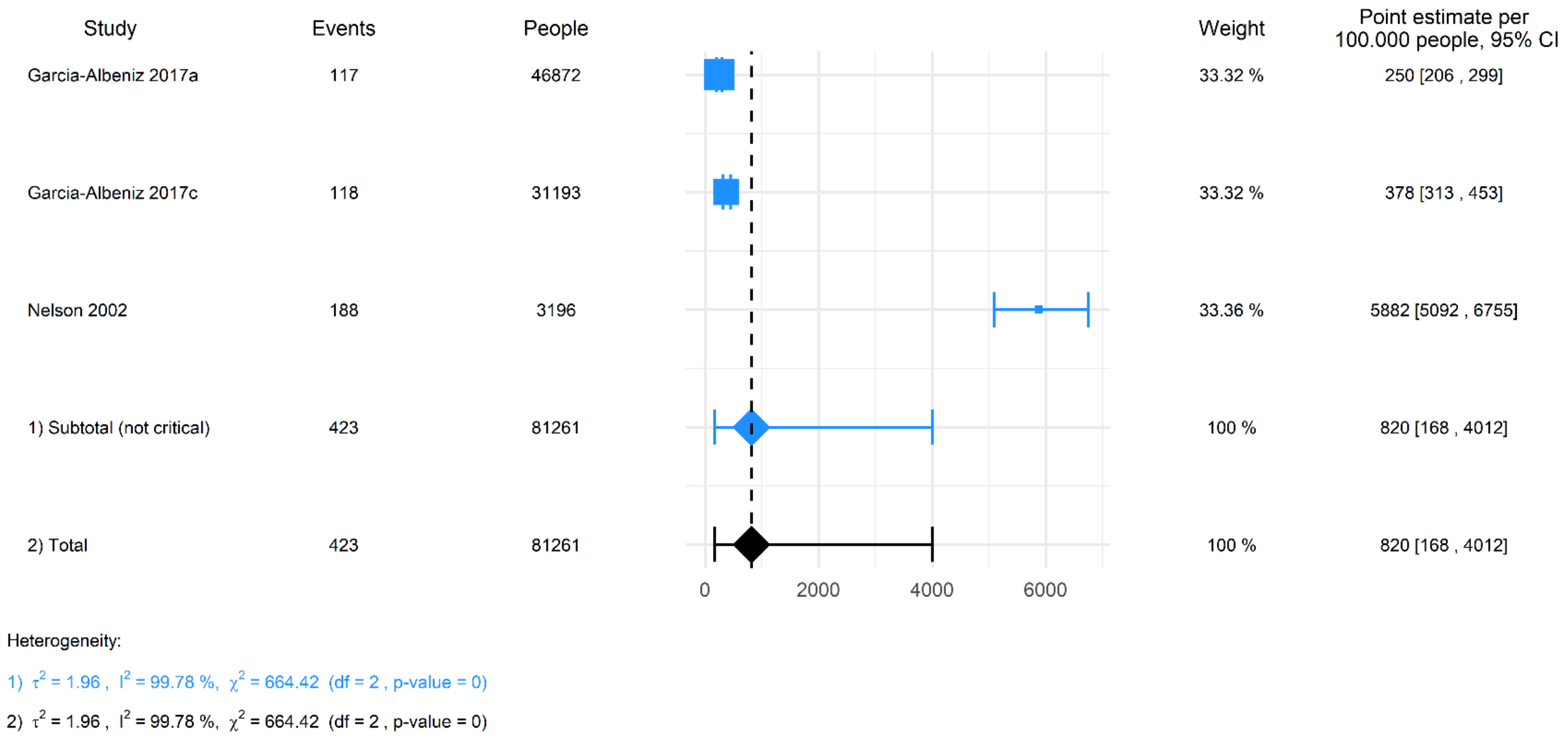

Figure 33. Blue-coloured object: Studies without critical risk of bias, Red-coloured object: Studies with critical risk of bias, Black-coloured object: All studies regardless of the risk of bias. Circles: Randomized Controlled Trials, Squares: Non-randomized studies, diamonds: Weighted summary measures. The blue diamond shows the weighted average for studies that do not have a critical risk of bias. The red diamond shows the weighted average for studies with critical risk of bias. The black diamond shows the weighted average for all studies regardless of their risk of bias.
