# Supplementary material for: Deaths and cardiopulmonary events following colorectal cancer screening—A systematic review with meta-analyses
Source: PLoS One. 2024 Mar 14;19(3):e0295900. doi: 10.1371/journal.pone.0295900 (PMC10939197; doi:10.1371/journal.pone.0295900)
Supplement: Attachment — Submitted filename: Response to reviewers FINAL, 1.docx [file pone.0295900.s006.docx]

Dear Ms. Divya Bhandari,

Thank you for considering our manuscript for publication in PLOS One.

Below, we respond to your comments about the journal requirement and to the comments from the peer reviewers. We have replied to the peer reviewers’ comments in a tabular format below.

We sincerely appreciate the thoughtful comments and valuable questions regarding our work from the reviewers. The feedback has been very helpful in guiding our revisions and addressing any areas that required clarification. We are grateful for the reviewer’s recognition of the hard work and effort we have invested in extracting and presenting the main findings of our review. We hope that you will find that we have taken the comments and questions raised by you and the reviewers adequately into account and that we have made the necessary changes to ensure a more coherent and reader-friendly article.

When we went through our manuscript, we found some minor errors and shortcomings that we have changed. These are (in the order they appear in the manuscript):

1) We have added an author affiliation to John Brodersen
2) We have added the following references:
   a) Updated CONSORT-harm reference to the recently published updated version since this does not change out methods or findings while referring the reader to the most recent version of the reporting guideline.
   b) Reference to the recently updated CONSORT-harms checklist where the older version is referenced in the discussion, section 5.1 summary of main findings.
   c) Reference to a recent systematic review that highlights the need for better harm assessment of screening programmes in a systematic review in the sentence where we advise that clinical trials do the same, section 5.4 future research.
   d) The reference list has been updated with correction of erroneous citations and we have added reference to all studies included for review.
3) Minor spelling mistakes have been corrected

Journal Requirements:

When submitting your revision, we need you to address these additional requirements.

1. Please ensure that your manuscript meets PLOS ONE's style requirements, including those for file naming. The PLOS ONE style templates can be found at [https://journals.plos.org/plosone/s/file? id=wjVg/PLOSOne_formatting_sample_main_body.pdf](https://journals.plos.org/plosone/s/file?%20id=wjVg/PLOSOne_formatting_sample_main_body.pdf) and [https://journals.plos.org/plosone/s/file?id=ba62/PLOSOne_formatting_sample_title_authors_affiliations.pdf](https://journals.plos.org/plosone/s/file?id=ba62/PLOSOne_formatting_sample_title_authors_affiliations.pdf%20%20)

**We have carefully followed PLOS ONE's style requirements for our manuscript, including adhering to the recommended file naming conventions. Our manuscript aligns with the provided PLOS ONE style templates, ensuring compliance with their guidelines**

2. We note that you have referenced (ie. Bewick et al. [5]) which has currently not yet been accepted for publication. Please remove this from your References and amend this to state in the body of your manuscript: (ie “Bewick et al. [Unpublished]”) as detailed online in our guide for authors [http://journals.plos.org/plosone/s/submission-guidelines#loc-reference-style](http://journals.plos.org/plosone/s/submission-guidelines%23loc-reference-style)

**We have looked through our reference list. Here, reference number 5 is not “Bewick et al.” but “Shaukat A, Levin TR. Current and future colorectal cancer screening strategies. Nature Reviews Gastroenterology & Hepatology. 2022. doi: 10.1038/s41575-022-00612-y”, which is a published article. Also, the author "Bewick et al." does not appear in our reference list.**

# Response to reviewers

## Reviewer 1

The authors presented a methodologically well-executed meta-analysis to quantify the risk of the most serious types of physical harm during CRCSPs, i.e., deaths and cardiopulmonary events (CPEs). However, I believe that the manuscript can be improved and I present my suggestions for each section:

| **Comments from reviewer** | **Response to reviewer** |
| --- | --- |
| Abstract:  I suggest removing in line 30 "Design Systematic review with descriptive statistics and random-effects meta-analyses" putting it in Methods: Systematic review with descriptive statistics and random-effects meta-analyses carried out according to PRISMA. Systematic search in the literature for studies investigating physical harms associated...  On lines 35 and 36 I suggest that you exchange "We used the ROBINS-I tool and the GRADE approach to assess the internal and external validity of findings, respectively." para: We used the ROBINS-I tool and the GRADE approach to assess the risk of bias and certainty of evidence, respectively.  In lines 46 and 47 I suggest the change to "We found a tendency towards lower estimates of deaths and CPEs in studies with very low certainty of evidence and a high risk of bias compared to non-critical studies. | We have revised the manuscript according to the suggestion to the following: *“Systematic review with descriptive statistics and random-effects meta-analyses of studies investigating physical harms following CRCSPs”*  Due to the word limit of 300 characters in the abstract, we have made a more condensed sentence about the risk of bias and the certainty of the evidence.  Our results did not show a dose-response correlation between the GRADE rating and the estimate of deaths or cardiopulmonary events. However, we did find a trend towards lower estimates of harm in studies with a critical risk of bias. Therefore, we have changed the wording to: *“We found a tendency towards lower estimates of deaths and CPEs in studies with a critical risk of bias.”* |
| Methods  3.1 Study eligibility  The main inclusion and exclusion criteria defined by the authors for the selection of studies must be clearly described here as text and not indicating that they are in Appendix 1.  3.6 The external validity of findings across studies included for review I suggest switching to:  3.6 The certainty of evidence of findings across studies included for review | We have amended the text in section 3.1 to comply with the suggestion with details about how screening and harms were operationalized and criteria for in- and exclusion in Appendix 1.  We have followed this comment and changed the wording from internal/external validity to risk of bias and certainty of the evidence throughout the manuscript. |
| Results  PRISMA 2020 recommends that in systematic reviews, authors present a main table, usually table 1, that shows the characteristics of the studies included in the review, containing authors, year, population, outcomes, main results and columns of risk of bias and GRADE certainty of evidence. Due to the large number of studies included, I believe that the authors can replace the confusing tables in the submitted manuscript with two tables, one with the characteristics of the studies in which the death outcome was evaluated and the other in which the CPEs outcome was verified.  On line 310 I suggest changing:  4.7 Characteristics of the external validity of findings (A5) for:   4.7 GRADE certainty of evidence to outcomes (A5)   Table 3 in line 314 and table 4 in line 321 can be excluded and in the tables previously suggested by me for each study the authors present in the column GRADE certainty of evidence if the study was evaluated as very low, low, moderate and high, being able to represent by the circle with a positive sign according to GRADE handbook. | This comment is in line with comment number 5 from reviewer 7. Please view our response to this comment regarding Table 1.  However, we do not find that the newly added table about study characteristics, referred to as Table 1 by the reviewer, (table 7a-c in Appendix 8) makes the remaining tables superfluous. We have removed tables 3, 4, 7, and 8, leaving 4 remaining tables, which we find present important and distinct information.  We cannot comply with the suggestion to include GRADE and bias ratings in the study characteristics table since these assessments are made on the outcome level where we have also distinguished between different subtypes of harm, e.g., arrhythmias as one type of cardiopulmonary event. Therefore one study contributes with data about multiple outcomes, and for each outcome assessment, i.e., the 151 subpopulations, we have made a bias rating, and for each type of outcome across subpopulations, we have made a GRADE rating. Both are presented in separate tables in the supplementary files.  We have changed the title to the following, which we think is more in line with the wording suggested above in the abstract section: *“4.7 Characteristics of the certainty of the evidence (A5)”.*  This comment aligns with comment number 18 and 19 by reviewer 7, please view our response here. We cannot comply with this comment since the GRADE rating is on the outcome level, not the study level. Therefore, we have kept tables 3 and 4 but moved them to the supplementary material. We have also added the GRADE rating to tables 5 and 6, which we hope aligns with the rationale behind this comment that the GRADE rating is presented alongside the findings. |
| Discussion  In lines 581 to 583 of the conclusion the authors state "Also, many types of CPEs occur during CRCSPs, including arrhythmia, vasovagal events, heart failure, and acute coronary syndrome, especially for older people and if polypectomy is performed."  However, there is not at least one paragraph in the discussion that discusses hypotheses that explain why CPEs are more frequent in the elderly and in patients undergoing polypectomy, or whether these results are similar to those reported in other systematic reviews. I suggest you add a paragraph or two about this, as these are important results for clinical decision-making in screening the elderly population.  Conclusion  On lines 580 and 581 I suggest changing to:  Based on our findings, we can conclude that deaths during CRCSPs are rare, yet they do occur, but with very low certainty of evidence. | We agree that these findings deserve to be contextualized better and linked to similar findings in other studies. We have added the following sentence to the end of section 5.1 summary of key findings: “*However, we found that arrhythmia, vasovagal events, heart failure, and acute coronary syndrome, occurred at higher rates among older people and if polypectomy is performed, which is consistent with findings in other studies [Gornick, 2022 #142] and in other reviews [Day, 2011 #143], potentially caused by the greater comorbidity in this age group, which have led to debate about the appropriate cut-off age for when to stop screening [Guittet, 2023 #144][Patel, 2022 #145].”*  We have added the suggested text to the sentence in the discussion. |

## Reviewer 2

| **Comments from reviewer** | **Response to reviewer** |
| --- | --- |
| Reviewer #2: This manuscript is a very meaningful systematic review research about colorectal cancer screening and physical harms such as deaths and cardiopulmonary events. I agree with their conclusion that deaths during CRCSPs are rare, yet they do occur.  But I think that such serious harms were complications of endoscopic interventions like polypectomy and that endoscopic intervention should be excluded like surgical intervention. | We thank reviewer 2 for these reflections and positive feedback. Our results support other findings that polypectomies likely increase the risk of harm. However, our results also show that harms do occur during endoscopic procedures without polypectomies. Ideally, we could divide analyses according to studies with and without polypectomies and we could calculate the added risk of specific types of polypectomy techniques. However, as stated in many places in the manuscript, reporting the rate of polypectomies and the specific technique used was so inadequate that the best we could do was to report a weighted average rate of polypectomies and highlight the issue of poor reporting in many studies. |

## Reviewer 3

Reviewer #3: Review Report

Title: Physical harms associated with sigmoidoscopy and colonoscopy during colorectal cancer screening - a systematic review with meta-analyses of deaths and cardiopulmonary events

| **Comments from reviewer** | **Response to reviewer** |
| --- | --- |
| 1) The title should be very refined and shortened. E.g., time and design. | Thank you for the suggestion. A shorter title might be more “catchy” for the reader. However, it also comes at the cost of being less precise than the current title, which outlines the subject studied (physical harm), the intervention (sigmoidoscopy and colonoscopy), the setting (colorectal cancer screening), the type of study (systematic review with meta-analysis), and the outcomes (deaths and cardiopulmonary events).  We would like to suggest the following title (also changed in the manuscript):  **Deaths and cardiovascular events following colorectal cancer screening – a systematic review with meta-analyses**  We leave the final decision to the editor. |
| 2) The type of the systematic review and meta-analysis should be specified. | We have conducted a classical systematic review (type) and a random effects meta-analysis. The type of review is a systematic review, which we have written in the title and the abstract. We have written that the type of meta-analysis is random effects in the abstract also. Therefore we think we have fulfilled the requested description. |
| 3) Still the knowledge gap in this section is not too detail and the reasonings are weak, | Thank you for this comment – however, it is not clear to us which section that needs to be clearer. Therefore we are not able to reply or make any changes. If the comment pertains to the introduction, this has been revised according to comments from other peer reviewers. |
| 4) Still the methods need some framing like    a) The particular timing of the harm.    b) The setting, types of specific diagnosis, who diagnosed and how, the sources of data and its similarity and difference, the test of heterogeneity, the study period and the criteria of deciding that specific time, is that only general hospital or referral and specialized hospital, who verified and how? Needs to be assessed and presented well. in addition, the strength and limitation of the included studies should be also well assessed.    c) The philosophical stance of the researchers.    d) Results should be still logical, concise and short.    e) The discussion, conclusion and recommendation should be entirely drawn from the study objectives. | a) This comment aligns with comment number 2 from reviewer 1. Please view our response to that comment. We have amended the text in section 3.1 of study eligibility to explain the timing of the harm. b) The requested information about studies and how outcomes were measured are now outlined in Tables 7a-c in Appendix 8. The tests for heterogeneity are described in the methods, section 3.9. Regarding the strengths and limitations of studies, we have conducted a thorough bias assessment, a GRADE rating, and we have written a detailed account of the strength and limitations of the evidence in this area in the discussion section. c) Thank you for noting the absence of this. To our knowledge, it is not customary within the quantitative sciences to explicitly state one’s philosophical stance. Instead, we have tried to elaborate the reasons for conducting a systematic review in this area in the introduction. d) We agree. We have followed the best-available guidelines for conduct and reporting in the area of harms of medical interventions. We acknowledge that the results section is lengthy. However, the evidence in this area is complex, heterogeneous and in general of poor quality, which has made it difficult to present it in a denser format. If the reviewer has any specific recommendations for improving clarity, we welcome these. e) We agree. We have provided a summary of findings that corresponds to the seven aims of the review and it is based on our results section. We go on to discuss the strengths and limitations of the evidence in the area and of our methods, followed by a comparison to similar types of studies, which is followed by recommendations for practice and future research, which we find is based on our findings. We conclude with our review questions. Again, if the reviewer has any specific recommendations for improving clarity, we welcome these. |

## Reviewer 4

Reviewer #4: Thanks for this opportunity to review this submission. The author of this study tried to systematically review and conduct a meta-analysis on the physical harms associated with colorectal cancer (CRC) screening interventions. The study is very well designed and conducted to investigate the interesting notion which appears also to be a crucial complication of CRC screenings. The manuscript is also well drafted. Just some revisions might help to enhance the draft. My comments and suggestions are provided below.

| **Comments from reviewer** | **Response to reviewer** |
| --- | --- |
| 1. Lines 22-51: the abstract needs to be formatted according to the PLOS One guidelines for submission. | We have structured the abstract according to the PRISMA-harms guideline, which is meant to guide reporting of systematic reviews that investigate the harmful effect of medical interventions. The PLOS One guideline for abstracts is less specific about the content of the abstract and we believe that we have adhered to both guidelines, summarizing the most important results and their significance, keeping the abstract within 300 words etc. (<https://journals.plos.org/plosone/s/submission-guidelines>) |
| 2. Lines 50-51: the final conclusion is somehow vague indicating “Physical harm during CRCSPs likely occurs more often than previously recognized”. A revision on the final conclusion might be better for abstract. | We agree. We have revised the abstract substantially to comply with comments from reviewers while keeping it within the 300-word limit. |
| 3. Lines 91-92: if any previous publication is online, it is suggested to cite it here. I found out a presentation published with the PROSPERO registration number ([https://ebm.bmj.com/content/24/Suppl_1/A29.1](https://eur02.safelinks.protection.outlook.com/?url=https%3A%2F%2Febm.bmj.com%2Fcontent%2F24%2FSuppl_1%2FA29.1&data=05%7C01%7Cfhm%40sund.ku.dk%7Caa5bf378f3794ee62de308db252a7c6e%7Ca3927f91cda14696af898c9f1ceffa91%7C0%7C0%7C638144637444345305%7CUnknown%7CTWFpbGZsb3d8eyJWIjoiMC4wLjAwMDAiLCJQIjoiV2luMzIiLCJBTiI6Ik1haWwiLCJXVCI6Mn0%3D%7C3000%7C%7C%7C&sdata=Lp15RfqG4SKvHQkKtIYa2qSosyXqMlolhYZDBwULFpc%3D&reserved=0)). | Thank you for bringing our attention to the online publication of an abstract before the oral presentation of the systematic review during a scientific conference. We have referenced the online material as requested. |
| 4. Line 114: as the latest update on literature search was made almost a year ago (march 4th, 2022), it is suggested to update the search for the revision to include any newly published papers in the field. | We agree that an updated literature search is key for systematic reviews. However, AMSTAR recommends that the literature search does not exceed 24 months, which the current date for the search strategy is well within. In addition, when we originally submitted the manuscript we were asked to update our search strategy. We updated the search from the 12^th^ of April 2017 to the 4^th^ of March 2022, which yielded 37 new studies eligible for review. This involved a significant workload extracting data from studies, assessing the risk of bias, the certainty of the evidence and rerunning all analyses, amounting to 9 months of work. Of note, the inclusion of the newly updated evidence did not change the results significantly. On these grounds, we do not consider it would add substantial new insights in this area that we updated the search strategy again. Conversely, we fear it would further postpone the publication of our results. |
| 5. Results: this section is lengthy and could be boring for readers. It is highly suggested to summarize the findings and move some of the numerous tables and figures to the appendix to make reading the results easier. | We have substantially revised the results section focusing on improving the readability of tables, moving supplementary content and most of the figures to the supplementary materials. Of note, the reviews aim to comply with the best-available guidelines for conducting and reporting of systematic reviews in the area, which sets a quite high standard for the required detail in the reporting of results. Despite this, we have tried to simplify as much as we could. |
| 6. Lines 443-462: it is not routine to evaluate previous systematic reviews in a review study and the studies might be removed and the comparison could be moved to the discussion section of the manuscript. | We agree that it is not within the scope of systematic reviews of original studies to evaluate other systematic reviews in the area. Therefore, we have removed the 7^th^ aim of the review (to compare our findings with findings in existing systematic reviews in the area) and we have moved the paragraph (section 4.10) to the discussion under the subheading *Comparison to other systematic reviews in the area.* |
| 7. Discussion: this section lacks the appropriate comparison with similar studies which is provided in the last part of the results section. | Please view the response above. |
| 8. Conclusion: this section also could be briefer and only reflect the final message of the manuscript and the rest could be stated in the discussion. | We agree that the conclusion should be as concise and accurate as possible. However, we have also tried to provide a comprehensive account of the most important findings from the review. We have rewritten the conclusion to reflect the final messages of the review. |

## Reviewer 5

| **Comments from reviewer** | **Response to reviewer** |
| --- | --- |
| The authors have conducted a systematic review and meta-analyses of complications dou to endoscopic screening or follow-up after a positive screening test. The review is comprehensive, contains data from 134 papers and must have been extremely time- consuming to conduct. The data is of obvious importance as screening for colorectal cancer is implemented in more and more countries.  The amount of data may be the authors’s greatest challenge, also for accuracy. For example, I know that 1 patient died after TC f-up of a positive FOBT in the 2021 Randel trial, but I did not find that death in the figures.  Otherwise, I have some suggestions which may improve readability. | We are thankful for the reviewer’s comments about the importance of the findings in the context of the global implementation of colorectal cancer screening programmes. Indeed, it has been extensive work to complete this review.  It is correct that we made a mistake during data extraction. We have corrected the error and amended the numbers in the data extraction tables and in Figures 6 and 7 where the study by Randel and colleagues contribute with data.  Data has been extracted by two reviewers independently to ensure that we do not make such mistakes. However, no system is perfect. Therefore, to ensure that there are no similar errors in data extraction, we have gone through our numbers once again to ensure that other harmful events have not been missed. (We did not find other missed events).  When we looked through our numbers we found that we had concluded on the number of deaths across all types of studies regardless of the risk of bias and the follow-up time. However, in the methods section, we defined our primary outcomes as assessments from studies without critical risk of bias with reported follow-up time. This has been corrected in the summary of findings section (section 5.1) and the abstract. |
| First of all - the figures and tables contains abbreviations that should generally be written in full. Table 3 may serve as an example that requires significant changes. Additionally – titles and figure legends should not contain abbreviations. As a rule of thumb – all tables and figures should be stand-alone parts of the manuscript. The colors of the Forest-plot should be explained in footnotes. | Table 3 has been revised and moved to the supplementary files.  The comment concerning the layout of tables in general overlaps with comments 17-19 from reviewer 7. Please view our response here. We have made substantial revisions to all tables in the manuscript and in the supplementary file, including the request to exclude abbreviations and write all concepts in full text.  Concerning the figure and explanation of colors in footnotes, this has been added as image text boxes throughout Supplementary file 2 – Figures. |
| When quantifying adverse events, a comparator group should be included, to account for events that would have occurred without screening. In the present manuscript, no comparator was used, and accordingly, quantification of adverse events (apart from obvious endoscopic complications like GI bleeding and perforation) is susceptible to the baseline risk of those events (e.g. cardiopulmonary, death). Because the included papers are derived from different age-groups and different populations, and without a comparator, the results are very difficult to interpret. The authors should elaborate more about this limitation in the discussion. | We agree that this limitation was not sufficiently described in our discussion. Therefore we have added the following to the manuscript file: *“Another limitation within the best-available evidence and hence our findings, is that only 5 studies (4%) had an unscreened control group with measurement of the outcomes of interest, physical harm. In effect, findings about physical harms, e.g., deaths and cardiopulmonary events, is susceptible to bias from confounding, i.e. events happening during or in the 30-day period following diagnostic procedures but not because of these procedures. This acts to bias our findings towards the overestimation of harms related to diagnostic procedures. However, more mechanisms likely act to cause underestimation of harms related to screening (section 5.4 Implications of findings).”* |
| Figures are not mentioned by occurrence in the text. Please re-assess. | The editing office at PLOS One asked that Figure 1 was the PRISMA flow chart even though it comes after Figure 2. We have no preference regarding what is Figures 1 and 2. The other figures have been moved from the manuscript file to the S2 – Figures, and the order has been revised so that figures are now mentioned by occurrence in the manuscript file (with reference to S2 – Figures). |
| Why was downgrading due to publication bias executed? What was the rationale?  4.3 belongs to methods section.  In addition, how did the authors distinguish between similar subcategories, e.g. pain, discomfort, colorectal symptoms. Maybe it would be better to collapse some categories to improve readability. It should not be necessary for the reader to scrutinize the appendix to understand definitions in detail. | Publication bias is a standard part of the GRADE assessment (<https://gdt.gradepro.org/app/handbook/handbook.html>). This comment overlaps with comment number 5 from reviewer 7. Please view our response here also: In appendix 5 under the 5^th^ domain of GRADE, publication bias, we have written about our considerations for the likelihood that there is publication bias regarding the evidence included for review and the likely magnitude of the impact of this on the results of the review. To comply with the AMSTAR checklist, we have added the key points from this paragraph in the appendix to the discussion, under section 5.3 (implications of findings).  Section 4.3 in the results section answers research question number 1: “to identify all physical harms associated with CRCSPs via the scientific literature”. Because it answers a research question and presents data from studies, we find it most suitable to place this section within the results section.  We acknowledge that it is difficult to categorize the harms of screening. We have explained our rationale and methods for subcategorizing outcomes into distinct categories in section 3.7.3. with further details for the interested reader in Appendix 6. We are willing to move some of the content from Appendix 6 into the manuscript file. However, the manuscript is already a long read, as noted by many of the reviewers. Therefore, we would prefer to keep the current structure.  Noting the concern about how harms were categorized, we have added the following sentence to the limitations section of the discussion: *“The heterogeneity of the evidence made the task of dividing outcomes into outcome types, and further to subcategorize each type of harm according to the severity and follow up time to make quantifiable groups of outcomes, a difficult task. It might be argued that our 17 types of harm, and how these were subcategorized, are to some extent arbitrary. However, we used the best-available guidance in the area to inform our categorizations, which were also thoroughly discussed in the author group. Still, our list of outcomes, and methods for subcategorization, should be seen as a work-in-progress that future studies might improve upon.”* |
| 4.5 belongs to methods section | Section 4.5 in the results section answers research question number 4: “to determine whether characteristics of the screening intervention or the screening population affect the risk for physical harm or the consequences thereof”. Because the section answers a research question and presents data from studies, we find it most suitable to place this section within the results section. |
| 4.7 Occurrence of death. The authors have categorized the studies in two subpopulations depending on follow-up time was reported or not. It would make more sense if the category with f-up time reported was split into categories depending on f-up time because complications must per definition be more frequent as the f-up time increases from e.g. 7 days to 3 months. | This is a fair point. However, some degree of heterogeneity between studies must be accepted to allow the compilation of findings from studies that will always be a little different from each other. We have argued for the legitimacy of compiling the evidence in meta-analyses in the last paragraph of section 5.2. Here, we have added the following text to existing arguments to comply with the reviewer’s comments: *“Of note, to allow compilation of the evidence we had to accept some heterogeneity between study characteristics, e.g., the age span of study populations, and within the categories of harm, to avoid ending up with as many categories as there were studies included for review. For example, in the outcome category deaths with follow-up time (Death-FUR) we accepted that the follow-up time varied from during the procedures to 3 months after the procedure also because the large majority of assessments (82%) had a follow-up time of 30 days. These spans of heterogeneity for a certain variable, here follow-up time, was allowed when we did not find significant variation in the occurrence of the outcome, i.e., despite different follow-up times between assessments we did not observe any systematic differences.”* |
| 4.8.1-4 Number of deaths per 100 000 screened should be reported. Tables should include follow-up time in categories. | The number of deaths per 100,000 people screened is reported in Table 5 and the text within sections 4.8.1-4. The follow-up time included in the categories of harm, e.g., death-FUR is available in Appendix 8, tables 7a-c. |
| 4.8 What is NDCPE? Please write in full first time. | We have written the definition in full the first time it is used in the manuscript file. |
| Line 395: up time not reported”, “other”, and “Non-defined cardiopulmonary events (NDCPE)” | We have written the definition in full the first time it is used in the manuscript file. |
| 4.9 Belongs to discussion section | Section 4.9 in the results section answers research question number 6: “to quantify the risk and the consequences of physical harm when possible”. Because it answers a research question and presents data from included studies, we find it most suitable to place this section within the results section. |
| Consequences of CPE: This section is a pure description of the studies that reported these events, but no results. Either results should be reported, or this section should be omitted. | As above, this section describes our findings pertaining to research question number 6: “to quantify the risk **and the consequences** of physical harm when possible”. Due to the poor reporting of the consequences of CPEs in studies included for review and taking the length of the manuscript into account, we find that the current description of consequences is as good as it gets. |
| 4.10 belongs to discussion | This comment is in line with comment number 6 by reviewer 4. Here, we have responded: “We agree that it is not within the scope of systematic reviews of original studies to evaluate other systematic reviews in the area. Therefore, we have removed the 7^th^ aim of the review (to compare our findings with findings in existing systematic reviews in the area) and we have moved the paragraph (section 4.10) to the discussion under the subheading *Comparison to other systematic reviews in the area.*” |
| The authors claim that severity of harm was included in their review. This is not clear to me after reading tables in section 4.9. Please clarify. | We have aimed to assess the severity of harm in line with the recommendation from ASGE (Cotton PB, Eisen GM, Aabakken L, Baron TH, Hutter MM, Jacobson BC, et al. A lexicon for endoscopic adverse events: report of an ASGE workshop. Gastrointestinal endoscopy. 2010;71(3):446-54. doi: <https://doi.org/10.1016/j.gie.2009.10.027>.).  We have reported what we find are the two most severe types of physical harm from the list of 17 potential types of physical harm associated with CRCSPs that we found. The severity of deaths cannot be subcategorized/described further. In terms of cardiopulmonary events, in section 4.9 we have reported that despite assessment of CPEs for 104 subpopulations, the severity of the CPE assessed was only reported for 10 subpopulations. |
| Strengths and limitation: In addition to the point I addressed previously, the authors should also mention no age categories as reasons for heterogeneity. CPE are clearly more prevalent in 75-years old compared to 45 year old individuals. | This is a fair point. We have added this to the discussion in the limitation section (please view our response above to the reviewer’s comment about 4.7 Occurrences of death. |
| The authors should also include that most studies does not include complications from surgery due to screening findings. Especially, surgery for benign polyps that would never have caused any harm to the screenee (and could have been removed endoscopically in specialized centres) is problematic, and mortality rates of about 1%, and serious complication rate of about 15% has been reported, and may tip the harm-benefit balance in the wrong direction. Of interest, the Funen gFOBT study included death due to surgery in their analyses with the consequence that the CRC mortality reduction from screening disappeared. | We agree that this is an important limitation to acknowledge. We have revised the first paragraph in section 5.2 to the following to comply with the reviewer’s comment: *“One of the review’s main limitations is that it exclusively accounts for physical harms resulting from invasive diagnostic procedures, e.g. sigmoidoscopy and colonoscopy, and not physical harms associated with procedures or treatment before or after these procedures. In effect, the true extent of physical harm associated with CRCSPs requires analyses of the other steps of the screening cascade, e.g. surgical treatment following diagnostic workup. In addition, we must acknowledge that this publication only provides an account of the two major types of physical harm, namely deaths and CPEs, related to the diagnostic procedures during screening.”* |

## Reviewer 6

In this study, which is a very difficult systematic review to understand and read, the authors aimed to review the evidence on physical harms associated with colorectal cancer screening programs and, when possible, quantify the risk of the most serious types of physical harm during colorectal cancer screening programs i.e., deaths and cardiopulmonary events.

| **Comments from reviewer** | **Response to reviewer** |
| --- | --- |
| The authors state that they classify the study's results and do not present them all in this article. Generating Multiple Publications from One Research Study in Academic Writing: An Ethical Problem or not. Although this is also a topic of discussion, it should be mentioned in the study's limitations section. Besides, my own opinion is that it is very difficult to understand a study that includes so much data at once. Despite the fact that this circumstance is mentioned in the article, it must also be mentioned in the limitation section. | We acknowledge there are ethical implications of writing multiple publications from one study. However, as discussed in the referenced article, it might be equally problematic to write too lengthy and complex papers, which are at the cost of readability and thus scientific impact.  Ethical concerns arise when there is a lack of transparency, misrepresentation, or when duplicate publication occurs without appropriate acknowledgement or justification. Therefore, we have tried to be transparent about the justification for our choices.  In the introduction section we have described how we encountered a much larger, more complex, and very heterogeneous evidence base compared to our expectations. We have included 134 studies for review that report on 17 different types of harm, which we have further subcategorized, e.g., cardiopulmonary events had to be divided into 17 distinct subcategories to allow meaningful quantification. To allow adequate attention to findings, and to avoid writing a paper that no one would have the time or energy to read, we decided it was best to divide reporting of the review into more than one publication.  In the discussion, we have amended the wording around the issue to the following: *“In addition, we must acknowledge that this publication only provides an account of the two major types of physical harm, namely deaths and CPEs, related to the diagnostic procedures during screening. Analyses of other types of physical harm associated with CRCSPs are planned for separate publications. Based on the literature in the area, we judged that the benefits of separating reporting of the review’s findings into more than one publication outweigh the caveats and ethical concerns of this practice [Altay, 2021 #147;Sathyanarayana Rao, 2016 #146].”*  We would like to quote one of the referenced articles: “There is nothing unethical in conducting a study and presenting part of the results in one paper and the rest of the results in another paper as long as each paper is based on the full sample and each paper contains sufficient information for a standalone publication”, <https://www.ncbi.nlm.nih.gov/pmc/articles/PMC5270256/> |
| While the physical injuries are classified, they are divided into 17 subgroups, but there is considerable overlap between the groups: perforation, bleeding, colorectal complications and postpolypectomy syndrome are examples of classifications that overlap.  In addition, complications related to bowel preparation are intertwined with numerous subgroups. This method of classification is extremely confusing. | We agree that the classification of harms is a difficult task. In section 3.7.3 we have described how harms were categorized and subcategorized. We have used the best-available guidance in the area to guide our categorization (ASGE review of adverse events in colonoscopy, the ASGE lexicon, and the Canadian Association of Gastroenterology consensus guidelines on safety and quality indicators in endoscopy). Further, harms were categorized following full data extraction by multiple reviewers to promote objectivity and reliability of categorizations. Still, no categorization is perfect and we consider our categorization as a starting point for ongoing discussion about how the harms of colorectal cancer screening should be defined and measured. This latter point is raised in the discussion section, section 5.3. *“Our findings highlight the need for consensus on how harms should be measured and reported in clinical studies, e.g. a core outcome set [62]. Currently, a taxonomy is lacking that clearly defines and conceptualises all effects of screening, e.g. benefits, harm, costs, and legal and ethical implications for people and society. Our taxonomy distinguishes between 17 categories of physical harm, which are further subcategorised, resulting from screening. Here, we define harm as any negative effect from screening participation perceived by the screening participant or their significant others [63, 64].”* |

## Reviewer 7

I would like to start by thanking you for sharing interesting information through this meta- analysis on physical harms associated with CCS. However, I have some comments and queries that readers may have while perusing this article. Please feel free to accept or reject the comments with rationale. The volume of comments may seem too much, but many of the comments are focused on formatting issues and clarity also.

General: I understand the hard work and effort put into extracting and presenting all the main findings identified during your review. Most authors would not have reported results with all the breakdowns and sub-categories. I appreciate the effort. However, if possible, I request that you go through your manuscript and streamline or make it concise, clear, and present it systematically. The following comments might also clarify what changes or updates can be made.

| **Comments from reviewer** | **Response to reviewer** |
| --- | --- |
| 1) Keywords: There are too many keywords right now. Limit the number of keywords to around 8-10. Remove "Grade" and "Robins" keywords. The PubMed Mesh database can be used to generate proper keywords if necessary. | We have limited the number of keywords to nine. We have removed GRADE and ROBINS-I. These are the current keywords:   1. Colorectal Neoplasms / diagnosis* 2. Colorectal Neoplasms / mortality 3. Early Detection of Cancer* / adverse effects 4. Risk 5. Sigmoidoscopy 6. Colonoscopy 7. Mass screening 8. Meta-Analysis as Topic 9. Systematic Review [Publication Type] |
| 2) Remove the "design" subheading in the abstract and move the sentence under it to the "Methods" heading. | We have followed this recommendation. |
| 3) On the title page, please write down the city and country too in the affiliations where missing. Otherwise, in the future, those missed out will only trigger one more round of review/revisions. Fit the title page on one page, if possible. | We have revised it as requested. |
| 4) Section 3.2: a) If there were no differences (other than dates) in the two searches (original and updated search), I suggest ignoring reporting two searches. Instead, authors may report that the search was done from inception to March 2022 (Suggestion). b) In this paragraph, clarify whether grey literature, trial registries, and references of identified papers were searched or not, and explicitly mention what type of study design was included here. | We have revised section 3.2 Information sources and search strategy as requested. |
| 5) These days, meta-analysis papers are evaluated using the Amstar-2 checklist (Refer: [https://amstar.ca/Amstar_Checklist.php](https://eur02.safelinks.protection.outlook.com/?url=https%3A%2F%2Famstar.ca%2FAmstar_Checklist.php&data=05%7C01%7Cfhm%40sund.ku.dk%7Caa5bf378f3794ee62de308db252a7c6e%7Ca3927f91cda14696af898c9f1ceffa91%7C0%7C0%7C638144637444345305%7CUnknown%7CTWFpbGZsb3d8eyJWIjoiMC4wLjAwMDAiLCJQIjoiV2luMzIiLCJBTiI6Ik1haWwiLCJXVCI6Mn0%3D%7C3000%7C%7C%7C&sdata=Q2CnkWBMdy7Q3P0bPwJNhc7vHQTwHcCR4WxW9ax8k10%3D&reserved=0)) to consider them as high quality or not, in order to include them as high-quality evidence for policy recommendation, etc. Therefore, if possible, I suggest that you go through the 16 criteria and address any missing criteria in your manuscript. To be specific, item 10: reporting on the sources of funding for the studies selected in your review, 14: explanation and investigation of sources of any heterogeneity in the results, 15: detail of publication bias assessment seems to be missing. If addressed or provided rational reasons, this can be considered high-quality work by future researchers. | We have followed this suggestion, read through the AMSTAR checklist, and amended the manuscript to comply with the recommendations within the AMSTAR checklist. This has led to the following changes to the manuscript file:   1) Reference to the AMSTAR checklist has been added in the first paragraph of the Methods section. 2) We have added information about the literature search strategy in section *3.2 Information sources and search strategy* 3) We have added three tables to the supplementary file in Appendix 8, a table with study characteristics of included studies (sTable7a), a table with information about subpopulations in studies with and without assessments of death and follow-up time (sTable7b), and a table with information about subpopulations in studies with and without assessments of cardiopulmonary events and follow-up time (sTable7c).   Comments to the points raised about specific items in the AMSTAR checklist   1) Item10: Funding.    a) At the beginning of the review process, we looked for sources of funding. However, it was rarely reported and when it was reported it was without sufficient detail to make use of the information, e.g. often only a grant number was referenced without any more information, which made us judge that the information was not relevant to readers in view of the abundance of information already in the review. We have added a sentence about the inadequate reporting of funding sources in the discussion, *section 5.4 Future research*. 2) Item 14: Explanations for observed heterogeneity.    a) Throughout the results section we have emphasized the heterogeneity of definitions, assessment and reporting of harms, which we also illustrate in Appendix 6 with the need to subcategorize cardiopulmonary events into 17 distinct subcategories, the very different conceptualizations of harms across studies compiled in Appendix 11, and in the newly added Tables 7a-c in appendix 8. Further, in the discussion we emphasize the heterogeneity in the summary of the results section (section 5.1.), we note that it is a limitation regarding the legitimacy of conducting meta-analyses, and in the conclusion we write *“The evidence on physical harms associated with CRCSPs was very low quality, and the heterogeneity was high”.* Noting the length of the current manuscript we do not find that it would benefit the paper to write more about heterogeneity. 3) Item 15: Publication bias.    a) In Appendix 5 under the 5^th^ domain of GRADE, publication bias, we have written about our considerations for the likelihood that there is publication bias regarding the evidence included for review and the likely magnitude of the impact of this on the results of the review. To comply with the AMSTAR checklist, we have added the key points from this paragraph in the appendix to the discussion, under section 5.3 (implications of findings)   We judge that the other items in the AMSTAR checklist not noted above were already sufficiently described in the submitted manuscript file. We have included a completed version of the AMSTAR checklist with the re-submission (S5 – AMSTAR checklist (completed)) |
| 6) Line 130: Authors mention "In case we could not retrieve full-text articles, we included the abstracts." Please clarify how MA was done with the abstract only or remove this sentence if incorrect. | It is correct that we included 8 abstracts of the total 134 publications included for review, which we could not retrieve as full-text articles, likely because these were never published as full-text publications. We judged that it was better to include these studies as they provide important information. We have added a sentence in the discussion section 5.3 Implications of findings about the role of publications bias and how these 8 abstracts illustrate that publication bias is likely.  We have revised the sentence in the Methods section to: “*In case we could not retrieve full-text articles, we included the abstracts and assessed these by the same methods as full-text articles.*”  Also, we have added the following sentence to the beginning of the discussion, section 5.1 summary of main findings: “*We included 134 publications for review, of these 8 (6%) were only available in abstract form.”* |
| 7) Please note that all appendices are included in S1 (supplementary information file 1). Therefore, please change your citation to inform that it is inside S1. For instance, "are listed in Appendix 3" to be written as "are listed in S1 Appendix 3". The same goes for figures that will not be in the main manuscript. For example, Fig 33 should be written as S2 Fig 33. | We have revised the wording, so that references to appendices reads (S1 – Appendix Y) and for figures (S5 – Figure X) |
| 8) Authors have around 33 figures. Only a few of these figures are planned to be in the main manuscript. Other figures will be in a supplementary info file (suppose S2 List of additional figures). S2 will be a PDF consisting of a table of content (with a list of figures) and shall consist of all additional figures in it. This additional S2 file will not be formatted by the journal and will be kept as it is. Therefore, all figures inside the S2 file shall have captions, legends, and footnotes together. Do not submit additional figures as each individual figure (in TIFF format). For the figures to be in the main manuscript, it shall be submitted individually in TIFF format, while its captions and footnotes shall be in the manuscript itself. | We have compiled figures that should be within supplementary file in S2 – Figures with captions and legends and footnotes. We will revise so that only figures that are intended within the manuscript file are uploaded as TIFF-format. |
| 9) Line 160-163: Avoid using bullets or lists in the main manuscript as much as possible and convert those to paragraph format. For example, it could be written as – “…excluding the following items: 1 baseline risk; 2 relative risk; 3 absolute risk; and 4 study design.”. Similar for line 258 to 273, etc. | We have revised the bullet point format to text format in the following places of the manuscript:  1. At the end of the introduction, the 7 aims of the review  2. Section 3.6 3.6 The external validity of findings across studies included for review  We thought it was best to keep the bullet point format in section 4.3, where we list the 17 types of physical harm. |
| 10) Line 209: The authors mention combining RCT and NRS as they were one-armed. Please clarify for readers unfamiliar with the term “one-armed”. | We have added the following sentence to section 3.8.1: *“i.e., the entire study population receives the intervention, there is no control group,”* |
| 11) Line 214: The authors mention calculating weights as the study size divided by the total population size across studies. Provide a rationale or reference for such usage. | We have added a reference to the Cochrane Collaboration’s Handbook for systematic review, chapter 10 about meta-analysis. |
| 12) Line 219: Author mentions “and related statistics”. Please mention what other statistics (chi-2, Q, p-value?) or remove vague terms. | We have changed to: Heterogeneity was quantified using I^2^ , τ^2^, and χ^2^ |
| 13) Line 220: For calculation of CI using the Clopper-Pearson method, provide references. | We have added a reference to the method and related methods. |
| 14) Line 223: “These are illustrated via the orange and blue lines in the Forrest plot”. Please note that figures and table shall be stand alone, therefore, add such info in the footnotes of all relevant figure itself. Footnotes for fig in main manuscript shall be below the fig caption in the main text or footnotes can be in the figure itself. | We have removed this sentence and amended figures as stated in our response to comment 8. |
| 15) The authors did not provide information on publication bias assessment in the main text method section. Please include the rationale for not conducting the assessment and why a -1 scoring was given for all. | We have commented on this in our response to comment number 5. We have added the following sentence to the manuscript, section 3.5 Risk of bias: *“Of note, publication bias, i.e. the selective publication of research studies based on their results, is assessed via the GRADE approach (Appendix 5).”* Here, we have also referenced Appendix 5 I S1 which describes the requested information. |
| 16) There is a discrepancy between line 277, which states that 57 of the 151 subpopulations had death assessment, and the third row of table 1, which shows 39 subpopulations. Please clarify or correct this inconsistency. | We have rephrased the sentence to make it clearer that the outcomes were assessed for 57 subpopulations, with reporting of the follow-up time for 39 of the subpopulations: *“Across the four screening interventions, the outcome death was explicitly assessed for 57 of the 151 (38%) subpopulations. For 39 of these 57 subpopulations, the follow-up time was reported (68%).”* |
| 17) Table 1 (also applicable to most of the tables and figures): Overall the quality, information, labels, footnotes etc. of tables and figures needs major attention and improvements. Tables and figures shall be stand alone. All the abbreviation used should be clarified in footnotes of table too (although clarified in the manuscript already). The captions of the figures and tables should be detailed enough to understand what is being presented without having to review the main text. Replace "outcome" with "death" in table 1 as it is for death assessment. Clarify that the values presented in the second and third rows of data are N and %, respectively. Clarify the difference in data presented in the "subpop with outcome assessed" and "outcome assessor" rows. Where is the asterisk symbol in the table1 data itself? Probably, no need to use boldface for all row heading and footnotes. | We have revised all tables in the manuscript to comply with PLOS One guidelines, which includes the requested information about abbreviations in footnotes, revised captions to make the tables understandable and to make overall improvements to readability.  The specific comments for Table 1 have been followed:  1. Replace "outcome" with "death" in Table 1 as it is for the death assessment  2. Clarify that the values presented in the second and third rows of data are N and %, respectively  3. We have clarified each of the rows and added the missing *  4. Boldface was removed from the table to reserve this for the table caption. |
| 18) Table 3 should provide the full form of abbreviations such as AFU, FUR, NRFU, FS, etc. in the footnotes. | This has been revised as part of the amendment in our response to comment number 17.  We found that the wording was a bit unclear and we have revised a sentence in section 4.8 about harm categories: “Deaths were analyzed as the total number of deaths, i.e. deaths with any follow-up time (Death-AFU), which was further subcategorized into two subcategories: Deaths with follow-up time reported (Death FUR) and deaths without follow-up time reported (Death NRFU) (Table 5).” |
| 19) Table 3 and Table 4 can be combined into one as there are already too many tables and figures in the main manuscript. | We have moved Tables 3 and 4 to the supplementary file but kept them separate as they concern two different outcome categories. |
| 20) Line 324's sentence is not grammatically clear. Please check and revise it. “We downgraded the evidence with -2 due to serious risk of bias in > 50% of studies for 25 analyses (89%) and   ….”. | We have changed the sentence to: *“We downgraded the evidence with -2 due to serious risk of bias in 25 analyses (89%) and with -1 in two analyses 7%).”* |
| 21) In Table 5, it would be helpful to include the number of subpopulations and sample size for each pooled estimate. For instance: “6 [1-45] (28%) 9 Subpop, 2123 people”. The meanings of "critical" and "non-critical" should be explained. The full form of missing abbreviations should be included in the footnotes. Instead of having "N per 100000 [95% CI] (I2)" in the title, it can be written in the first cell and clarified in the footnotes. Additionally, if available, GRADE for each estimate can be shown using symbols such as * and ** (for low grade, medium grade etc) to provide readers with information on the pooled estimate and its certainty. | We have changed the table to comply with the suggested adding of the number of subpopulations and how many people were screened in total across the subpopulations.  We have added the following footnote for the table: *“Each cell should be read as follows: The number, e.g., 6, is the weighted average of deaths per 100,000 people screened, followed by a 95% confidence interval, e.g., [1-45], the I^2^ measure of heterogeneity, e.g., (28%), the number of subpopulations in the analysis, e.g., 7 subpopulations, and how many people that corresponds to, e.g., 202,810 people, and finally the GRADE score for the pool of subpopulations that contribute with data for the outcome, e.g. GS: -4. Critical: Subpopulations with a critical risk of bias of the outcome Non-critical: Subpopulations with low to serious risk of bias of the outcome”*  We chose to add the GRADE score instead of the suggested ** for low/medium/high GRADE since none of the analyses had a GRADE rating above very low quality. |
| 22) The captions for figures 3-8 should be revised to be more detailed and clearer as standalone explanations. For example, "Fig 3 Pooled estimate and forest plot of deaths with any follow-up time associated with sigmoidoscopy only." In the footnotes, explain that blue represents xxx while red represents xxx and clarify what "critical" means. | We have revised the title for the figures to the following format: Fig XX Pooled estimate and forest plot of OUTCOME with FOLLOW-UP TIME associated with PROCEDURE GROUP.  We have also added a footnote for each figure that explains the colors in S2 - Figures. |
| 23) Caption of fig 5 should be moved to line 360, and adjustments should be made to other figures' caption placements accordingly. | Figures 3-29 have been moved to the supplementary file S2 as requested above in comment number 8. |
| 24) The authors mention that follow-up time is categorized as short-term for less than 14 days and long-term for 0 to 30 days on line 387. Please clarify why there is overlap. | We have revised the sentence to: *“We subcategorised according to the type of CPE, e.g. arrhythmia or heart failure, and according to the included follow-up time, dichotomised into a) short-term (< 14 days) and b) long-term (30 days).”* |
| 25) Table 6 needs the same improvements as Table 5, and the full form of ACS and TE should be included in the footnotes. | We have revised it like Table 5, comment number 21. |
| 26) The section "4.9.1 Sigmoidoscopy" should be detailed as "4.9.1 Sigmoidoscopy and CPEs" to avoid confusion and provide clarity to readers skimming through the manuscript.. Similar approaches for this kind of section title. | We have revised the subtitle both regarding deaths and cardiopulmonary events as suggested, e.g., 4.9.2 Colonoscopy following FOBT and cardiopulmonary events |
| 27) Table 7 and 8 repeats the information provided in table 6 and respective forest plots, therefore, these two tables can be safely dropped. | Due to the changes to Tables 5 and 6 we agree that Tables 7 and 8 can be deleted. |
| 28) Line 476 mentions that estimates range from 0 to 134 across meta-analyses. Please clarify whether this is across meta-analyses or across studies, as this range is not seen in result Table 5. | We have revised the sentence to: “*Yet, confidence intervals span from 0 to 91 deaths per 100,000 people screened in analyses of non-critical studies across the three groups of analyses, stratified according to follow-up time, illustrating uncertainty about the true effect*” |
| 29) The supplementary file does not include a list of the 134 studies with brief details that meet Amstar criteria. Please provide this information. | This has now been included, please view our response regarding comment number 5. |
| 30) To avoid potential concerns from publication teams, please convert the table in the acknowledgement section to a paragraph. | The table has been converted to plain text. |
| 31) Check references and add missing information for e.g. for 1(missing publisher, organization), 39, 45, 50 (delete text in line 749) , 59, 61 (missing journal name) etc. | We have looked through the entire reference list and added information when missing. |
| 32) Please delete the supplementary file "S2 General information about the systematic review" as the information is already included in the main manuscript or submission system i.e., funding, data availability, competing interest etc. Additionally, please remove the file "changes from 2021 to 2022 version" from the next submission | We have deleted "S2 General information about the systematic review". We will remove the file "changes from 2021 to 2022 version" from the next submission. |
| 33) The format of listing supplementary files in section 9 is unclear and too lengthy. Please consider listing them as follows: [S1 Appendices (includes all appendices from 1 to 22); S2 Additional Supplementary Figures (contains Fig 14 to Fig 33); S3 Prospero Protocol; S4 Prisma-harms Checklist]. | We have revised, updated and simplified the listing of supplementary files in the manuscript. |
| 34) The contents of section 10 (lines 862 to 917) are unnecessary and should be deleted as it provides a table of content for the main manuscript. | We agree, it has been deleted. |
| 35) Once again, Please review the format of the supplementary file carefully as it will be published as is. Especially font size, style, headings size and style, heading numbering etc are all not uniform. | We have reviewed all supplementary files and checked for spelling errors, formats etc. and that they comply with PLOS One guidance. |
| 36) S1 is the main supplementary file and should be a bookmarked PDF. To achieve this, simply save the file as a PDF with bookmarks in Word. | Noted. We will upload a pdf file on re-submission for each of the supplementary files. |
| 37) In S1, please address the following issues: 1) Resolve the two errors on page 2. 2) Include Fig 2 on page 3 directly in the PDF file rather than submitting it separately. 3) For Appendix 3 on page 31, rather than sharing the list of excluded papers in a Google Drive link, consider including the list in the PDF or using a permanent link such as the "figshare" repository. 4) Please present numerical values in decimal format as standard practice, rather than using a comma (e.g., 0.842 instead of 0,842). | We would like to include Figures 1 and 2 in the manuscript. We are of course willing to move them to the supplementary files instead if the editors wish it so.  We have resolved the two errors on page 2  We have changed the link from Google Drive to a link to an openly accessible project on the Open Science Framework repository.  We have changed the format of numerical values throughout. |
