# Supplementary material for: Deaths and cardiopulmonary events following colorectal cancer screening—A systematic review with meta-analyses
Source: PLoS One. 2024 Mar 14;19(3):e0295900. doi: 10.1371/journal.pone.0295900 (PMC10939197; doi:10.1371/journal.pone.0295900)
Supplement: Attachment — Submitted filename: Response to reviewers, nov23, 1.docx [file pone.0295900.s007.docx]

Dear Ms. Divya Bhandari,

Thank you for considering our responses to peer review and our revised manuscript for publication in PLOS One. Below, we respond to your comments about the journal requirement and to the comments from the peer reviewers. We have replied to each of the peer reviewers’ comments in **bold font**. Additionally, we have looked through the manuscript and the additional files for any remaining typos or grammatical errors, and we have improved the language throughout.

Apologies for not having explicitly noted that you served as reviewer #7. We were conscious of this, but now I see that we did not explicitly address this. Thank you for your helpful comments.

We have corrected minor spelling mistakes and re-read the entire manuscript, having made minor changes of wording to improve the language. Also, we have re-formatted the reference to *“CONSORT Harms 2022 statement, explanation, and elaboration: updated guideline for the reporting of harms in randomised trials”* by Daniela and colleagues. All changes are marked in tracked changes.

# Response to reviewers

## Reviewer #1

The authors complied with most of the recommendations made by the reviewers and justified with good arguments when they decided to keep the original text. Congratulations, as the manuscript is better for publication.

**Response:**

**Thank you for acknowledging our efforts to address your recommendations and for recognizing the improvements made to the manuscript. We appreciate your congratulations and are grateful for your positive assessment.**

## Reviewer #3

Dear authors,you have failed to present;

1. Single standing title.

2. Establishing strong niche

3. Flawed methods for the eligibility criteria, language description, consistency of tools, third reviewer, self selecting as 'author',not ise of terms,how the studies selected...etc.

54. Wide and non objective based lacking logical flow of results

5.The discussion and conclusion are also less strong to the title

Regards,

**Response:**

**Dear colleague, we believe we have made major changes to the manuscript according corrections and recommendations from all peer reviewers that are not recognized here. In some cases, we have not been able to fulfil all suggestions. In such cases, we have explained our reasons for keeping the original wording as recognized reviewer#1. Concerning the points raised here, we would like to highlight the following:**

1) **To comply with the previous suggestion and the current comment by reviewer #7, we have now changed the title to: “Deaths and cardiopulmonary events following colorectal cancer screening – a systematic review with meta-analyses”. We believe that this is a single standing title in line with the title of similar reviews that adequately describes the main goal of the review.**
2) **We have revised the introduction in the abstract to clarify the niche that we address: *“Therefore, we aimed to review the evidence on physical harms associated with endoscopic diagnostic procedures during CRCSPs and, when possible, to quantify the risk of the most serious types of physical harm during CRCSPs, i.e. deaths and cardiopulmonary events (CPEs).”* In addition, the introduction establishes the rationale for the review from lines 78-94.**
3) **The methods section follows recommendations from the Cochrane Handbook [34], the PRISMA-harms extension and the PRISMA 2020 reporting guideline [30, 35], the AMSTAR checklist [36] and scientific literature concerning the methodological challenges of reviewing the harms of interventions [23]. We would like to highlight that we have addressed all comments regarding methods from reviewer 3 in the last round of peer review. These new points raised here are difficult to address because they are unspecific and without examples and explanations. Shortly, we do not find that the eligibility criteria are flawed, the language has been revised throughout the manuscript and is quite standard for reviews, and we consistently use the same tools in the review, which are also used in similar reviews. It is unclear to us what is meant with the following: *“third reviewer, self selecting as 'author',not ise of terms,how the studies selected...etc.”***
4) **The results section was substantially revised following the last round of peer review with revision of tables and wording throughout following helpful suggestions from many of the reviewers. However, it is a difficult task to present the results in a simple manner due to the poor quality and the heterogeneity of the evidence as noted in our response to a similar comment form the peer reviewer in the last round of peer review: “*We agree. We have followed the best-available guidelines for conduct and reporting in the area of harms of medical interventions. We acknowledge that the results section is lengthy. However, the evidence in this area is complex, heterogeneous and in general of poor quality, which has made it difficult to present it in a denser format. If the reviewer has any specific recommendations for improving clarity, we welcome these.”***
5) **We would like to refer to our earlier response concerning the conclusion and the discussion, which has also been revised according to recommendations from other peer reviewers: *“We agree. We have provided a summary of findings that corresponds to the seven aims of the review and it is based on our results section. We go on to discuss the strengths and limitations of the evidence in the area and of our methods, followed by a comparison to similar types of studies, which is followed by recommendations for practice and future research, which we find is based on our findings. We conclude with our review questions. Again, if the reviewer has any specific recommendations for improving clarity, we welcome these.”***

## Reviewer #4

With many thanks for the efforts made to revise the manuscript based on the comments and the responses to the raised queries, I have no further comments and suggestions and I endorse publication of this submission.

**Response: Thank you very much for endorsing the publication of the manuscript and your acknowledgment of our efforts to address comments and respond to queries.**

Reviewer #5: (No Response)

Reviewer #6: (No Response)

## Reviewer #7

Thank you for addressing all of my 37 comments provided in the earlier version.

**Response: Thank you for the very helpful comments, we find that the manuscript has been significantly improved due to your suggestions.**

Thanks for updating keywords with Mesh as per my comment C1. However, please remove any unnecessary sub-headings and sub-tags followed by “/”, so that the final version of keywords appears as follows:

Colorectal Neoplasms, Early Detection of Cancer, Risk, Sigmoidoscopy, Colonoscopy, Mass screening, Meta-Analysis, Systematic Review

**Response: Thank you. We have changed the MeSH terms according to your suggestion.**

The footnotes in the tables are currently formatted in boldface. It may be advisable to consider removing the bold formatting for improved readability.

**Response: Thank you for this suggestion. We have reformatted so it is without bold face.**

I acknowledge that, based on reviewer's comment, the authors have suggested a more concise title, namely i.e., “Deaths and cardiovascular events following colorectal cancer screening – a systematic review with meta-analyses”. However, I have a reservation about the term "cardiovascular" not aligning entirely with the content of the manuscript, as the authors have exclusively utilized the term "cardiopulmonary" within the text. I kindly request that you revisit this aspect. I am open to retaining the original title or any other title that authors believe accurately represents your research. Therefore, please give due attention to the manuscript's title during the final technical review or proofreading stages.

**Response: Thank you for noting that we wrote cardiovascular and not cardiopulmonary as used in the main text. We have revised the title to: “Deaths and cardiopulmonary events following colorectal cancer screening – a systematic review with meta-analyses”.**
